# A Retrospective Analysis of Hepatic Disease Burden and Progression in a Hospital-Based Romanian Cohort Using Integrated Cross-Sectional and Longitudinal Data (2019–2023)

**DOI:** 10.3390/jcm15020454

**Published:** 2026-01-07

**Authors:** Alina Dumitrache (Păunescu), Nicoleta Anca Șuțan, Diana Ionela Popescu (Stegarus), Liliana Cristina Soare, Maria Cristina Ponepal, Cristina Florina Mihăescu, Maria Daniela Bondoc, Muhammed Atamanalp, Ana Cătălina Țânțu, Cătălina Gabriela Pisoschi, Ileana Monica Baniță, Monica Marilena Țânțu

**Affiliations:** 1 Doctoral School of University of Medicine and Pharmacy of Craiova, Petru-Rareș Street no. 2, 200349 Craiova, Romaniacatalina.tantu8@gmail.com (A.C.Ț.); c_pisoschi@yahoo.com (C.G.P.); monica.banita@yahoo.com (I.M.B.); 2 Department of Natural Sciences, National University of Science and Technology POLITEHNICA Bucharest, Piteşti University Centre, 1st Targu din Vale Str., 110040 Pitesti, Romania; liliana.soare@upb.ro (L.C.S.); maria.ponepal@upb.ro (M.C.P.); florina.mihaescu@upb.ro (C.F.M.); 3 National Research and Development Institute for Cryogenic and Isotopic Technologies—ICSI Ramnicu Valcea, 240050 Ramnicu Valcea, Romania; diana.stegarus@icsi.ro; 4 Department of Finance, Accounting and Economics, National University of Science and Technology POLITEHNICA Bucharest, Piteşti University Centre, 1st Targu din Vale Str., 110040 Pitesti, Romania; daniela.bondoc@onmicrosoft.upb.ro; 5 Department of Aquaculture, Faculty of Fisheries, Ataturk University, 25240 Erzurum, Turkey; mataman@atauni.edu.tr; 6 Department of Medical Assistance and Physical Therapy, National University of Science and Technology POLITEHNICA Bucharest, Piteşti University Centre, 1st Targu din Vale Str., 110040 Pitesti, Romania; marilena.tantu@upb.ro

**Keywords:** chronic liver disease, disease progression, hospitalization burden, clinical profiles, Argeș County, Romania

## Abstract

**Objective:** To analyze demographic traits, clinical complications, and healthcare use in patients with chronic liver disease across major etiologies in a large Romanian cohort. **Methods:** A retrospective study (2019–2023) of 2359 patients with chronic hepatitis C (CHC), hepatitis associated with alcohol (ALH), cirrhosis associated with alcohol (ALC), or non-alcoholic cirrhosis (NALC). Data on demographics, clinical outcomes, and hospitalizations were analyzed using descriptive statistics, regression modeling, and clustering in IBM SPSS 27.0.1. **Results:** CHC patients were oldest (mean 67.5 ± 12.3 years), while ALH patients were youngest (56.0 ± 11.0 years). CHC prevalence increased with age (10.0% in ≤30-year-olds to 87.1% in ≥81-year-olds; γ = 0.535, *p* < 0.001). Females comprised 60–70% of CHC cases, males > 85% of ALH and >78% of ALC. Mean hospitalization duration decreased from 13.80 days (2019) to 9.10 days (2023), yet cirrhotic patients had the longest stays (NALC: 16.37 ± 14.34; ALC: 17.66 ± 12.96) versus CHC (10.38 ± 10.14). Etiology was the strongest predictor of hospitalization length. Portal hypertension (PH) was the most common complication (54.3%), with males bearing more severe hepatic complications (ascites—38.3%; PH—66.8%). **Conclusions:** Hospital-based Romanian cohort analysis revealed that patient presentation and outcomes are fundamentally shaped by the interplay of etiology, sex, and age. We found a distinct female predominance in CHC, a pronounced male predominance in alcohol-related diseases, and evolving trends in non-alcoholic cirrhosis. These determinants dictate specific epidemiological patterns, hospitalization burdens, and complication risks, underscoring the critical need for a paradigm shift toward personalized, etiology-driven, and sex-tailored clinical management.

## 1. Introduction

Chronic liver diseases (CLD), characterized by the presence of harmful environmental factors and lifestyles populated by unhealthy habits from various perspectives such as smoking, diet, stress, unjustified and inadequate consumption of medications, sedentary lifestyle, alcohol consumption, have acquired the characteristics of a true public health problem [1]. From this category of diseases, hepatitis of various etiologies stands out, acute, but especially chronic ones, which have a significant impact both on the quality of life of individuals and on health systems [2]. These are characterized by a high prevalence, an important mortality rate and a significant socio-economic influence through significant costs, the impact on the socio-professional life of individuals and high consumption of resources [3,4].

The global burden of CLD and cirrhosis is substantial [1]. Although vaccination, screening and antiviral treatment campaigns for hepatitis B and C have reduced the burden of CLD in some parts of the world, concomitant increases in injection drug use, alcohol abuse and metabolic syndrome threaten these trends [5]. Thus, viral hepatitis continues to pose a significant challenge to global health, being one of the leading causes of death after COVID-19 in 2022 [6]. Continued efforts to address CLD-related morbidity and mortality require accurate contemporary estimates of epidemiology and outcomes.

Recent epidemiological analyses show that liver cirrhosis remains among the top 15 causes of global mortality, accounting for more than 1.3 million deaths annually [7]. Importantly, while the incidence of viral hepatitis-related cirrhosis has declined in regions with widespread access to antiviral therapies, metabolic dysfunction-associated steatotic liver disease (MASLD) and alcohol-related liver disease have become the dominant drivers of cirrhosis and HCC in Europe and North America [8,9,10]. MASLD alone affects more than 30% of the global adult population and is projected to increase concomitantly with obesity and type 2 diabetes [11,12,13].

Alcohol consumption remains a critical determinant of liver disease progression. High-risk drinking patterns have risen in many European countries over the past decade, leading to increased rates of hepatitis associated with alcohol (ALH) and cirrhosis associated with alcohol (ALC), particularly in younger adults [3]. Moreover, recent studies show that ALH is associated with extremely poor short-term prognosis, with 28-day mortality surpassing 25% in severe cases [14]. These alarming trends emphasize the need for both preventive and clinical strategies targeted at alcohol-related liver disease.

In parallel, the global rise in metabolic syndrome has transformed MASLD into the leading cause of chronic liver disease, with projections indicating a doubling in MASLD-related cirrhosis and hepatocellular carcinoma (HCC) by 2040 [15,16]. The natural history of MASLD shows strong associations with cardiovascular disease, which remains the primary cause of death in these patients [17]. Thus, integrating metabolic, cardiovascular, and hepatic risk management is critical for improving long-term outcomes.

Furthermore, contemporary data highlight the importance of demographic determinants, particularly age and sex, in shaping disease distribution and prognosis. Women tend to exhibit higher prevalence of chronic hepatitis C (CHC) in some populations, potentially due to historical patterns of iatrogenic transmission [18,19,20]. Furthermore, females develop liver cirrhosis at significantly lower levels of alcohol consumption than males, showing a markedly higher dose-dependent risk for both morbidity and mortality [21].

Despite the established global burden of chronic liver disease, a critical scarcity persists in Eastern Europe regarding large-scale, longitudinal data that integrates cross-sectional prevalence with temporal progression and healthcare utilization. To address this gap, we conducted a retrospective analysis of a large hospital-based cohort from the Pitești County Emergency Hospital, Argeș County, spanning 2019–2023.

The primary objective of this study was to examine the association between liver disease etiology and clinical course in a large hospital-based cohort, using both cross-sectional and longitudinal approaches. Specifically, we aimed to characterize demographic profiles, comorbidity patterns, complication burden, and disease severity across major etiological categories of chronic liver disease.

Secondary objectives included the quantification of hospitalization burden and its temporal dynamics, the identification of independent predictors of advanced liver disease as assessed by Child–Pugh classification, and the evaluation of longitudinal changes in disease severity among repeatedly hospitalized patients. In addition, cluster analysis was applied to explore clinically meaningful patient subgroups based on disease manifestations and severity.

By situating these analyses within a five-year regional cohort, this study seeks to address the current lack of longitudinal epidemiological data on chronic liver disease in Romania and Eastern Europe, delivering much-needed evidence to optimize patient care pathways within the regional healthcare context.

## 2. Materials and Methods

### 2.1. Study Design, Setting and Patient Population

The retrospective analysis in the hospital-based Romanian cohort included 2359 patients diagnosed with hepatic diseases, who were admitted to the Pitești County Emergency Hospital, Argeș County between 1 January 2019, through 31 December 2023.

The hospital serves a catchment area for an average of 630,000 inhabitants, representing a significant demographic segment of southern Romania (https://www.citypopulation.de/en/romania/admin/sud_muntenia_/RO311__arge%C8%99/, accessed on 20 November 2025).

Eligible cases included patients with at least one confirmed diagnosis of CHC (diagnostic code B18.2), ALH (diagnostic code K70.1), NALC (diagnostic code K74.6), and ALC (diagnostic code K70.3), as defined by the International Classification of Diseases. Tenth Revision, Clinical Modification—ICD-10-CM [22]. In this manuscript, the term non-alcoholic cirrhosis is used to refer to cirrhosis related to MASLD, in accordance with previously published work [23].

Each patient was recorded based on the unique identifier (ID) and was stratified by primary diagnosis, sex (female/male), age group (≤30, 31–40, 41–50, 51–60, 61–70, 71–80, ≥80) and by the earliest year of diagnosis.

The study was approved by the Scientific Ethics and Deontology Commission of the University of Medicine and Pharmacy of Craiova (no. 48/29 January 2024).

### 2.2. Data Source and Extraction

Data was retrieved from the hospital’s integrated medical services management information system (Hippocrates) and were subsequently coded into an anonymized electronic dataset. The extracted variables included patient ID, year of hospitalization, age, sex, length of stay, primary diagnosis, comorbidities [arterial hypertension (AH), cerebrovascular accident (CVA), heart failure (HF), type 2 diabetes mellitus (DM), obesity (Ob)], disease severity (Child–Pugh A, B, C), and major complications [ascites, esophageal varices (EV), digestive bleeding (DB), hepatic encephalopathy (HE), hepatocellular carcinoma (HCC), hepatorenal syndrome (HRS), and portal hypertension (PH)].

Treatment status for CHC patients and abstinence data for alcohol-related liver disease were not consistently available in the electronic records and were therefore not included in the analyses.

Additionally, liver disease severity was assessed using the Child–Pugh classification, which combines objective laboratory parameters (bilirubin, INR, albumin) with clinically relevant manifestations (ascites and hepatic encephalopathy) that are directly related to hospitalization burden and disease decompensation.

Although the laboratory variables required to calculate the MELD score were available, MELD was not selected as a primary severity measure because it is primarily intended for short-term mortality prediction and transplant prioritization, rather than for population-level characterization of disease severity in hospitalized cohorts. In contrast, Child–Pugh remains widely used in epidemiological and clinical studies to describe functional hepatic reserve and patterns of decompensation across etiological groups.

Given the epidemiological focus of the present study and its emphasis on longitudinal hospitalization patterns and complication burden, Child–Pugh was considered more appropriate for addressing the study objectives. Future studies may integrate both scores.

### 2.3. Data Preprocessing and Cohort Construction

Given that many patients experienced multiple admissions, a two-step preprocessing strategy was applied to generate complementary datasets.

A cross-sectional (unique-ID) dataset was created using the AGGREGATE procedure in IBM SPSS Statistics 27.0.1 (IBM Corp., Armonk, NY, USA), with ID specified as the break variable. Aggregation rules were defined as follows: age and year of hospitalization—minimum value (first admission); days of hospitalization—sum across all admissions (total hospital burden); diagnosis—first recorded diagnostic code (CHC, ALH, NALC, or ALC); comorbidities and complications—maximum value (presence at any hospitalization); Child–Pugh score—first recorded value (baseline disease severity). No missing values were found among the 2359 unique patients, confirming dataset completeness and internal consistency.

A second longitudinal (multiple-ID) dataset was constructed to evaluate the temporal evolution of hepatic disease, including those patients with ≥2 hospitalizations (*n* = 815) along with unique-ID cohort, totalizing 4341 records, with complete data for all cases. Using an additional AGGREGATE procedure, the number of admissions per patient and the mean hospitalization days were calculated. This structure enabled the assessment of disease progression, complication onset, and cumulative hospital utilization over time.

For analyses of baseline demographic, clinical, and hospitalization characteristics, the full unique-ID cohort (*N* = 2359) was used. Longitudinal analyses of disease progression were restricted to the sub-cohort of patients with ≥2 hospitalizations.

Descriptive analyses were conducted separately for the unique-ID cohort and the multiple-ID cohort.

### 2.4. Statistical Analysis

All analyses were performed using IBM SPSS Statistics (versions 27.0.1 and 29.0). A two-tailed *p* < 0.05 was considered statistically significant. Bootstrapping with 1000 resamples was employed to generate robust 95% confidence intervals (CIs) where indicated.

### 2.5. Descriptive Statistics and Distributional Analyses

Continuous variables were presented as mean with standard deviation (SD) or median with interquartile range (IQR), based on distribution normality assessed by the Kolmogorov–Smirnov test. Categorical variables were expressed as frequencies and percentages (n, %). Age was categorized into seven groups for specific analyses. Associations between categorical variables (e.g., diagnosis and sex) were evaluated using Pearson’s Chi-square tests, with Cramer’s V reported as the effect size.

### 2.6. Hospitalization Burden and Temporal Dynamics

Hospitalization days (length of stay) were described using descriptive statistics (mean, median, IQR) stratified by diagnosis, sex, and age group. To model the determinants of hospitalization duration, a Generalized Linear Model (GLM) with a Gamma distribution and log-link function was fitted, with diagnosis, year, and their interaction as fixed effects. The Gamma coefficient was used to assess the strength and direction of associations between ordinal variables. It was chosen because it is appropriate for ordered categories, robust to ties, and provides a directional measure of association, allowing the detection of monotonic trends in disease prevalence across consecutive years.

Results are reported as Estimated Marginal Means (EMMs) with 95% CIs. Type III Wald Chi-square tests were used to assess significance. The One-way ANOVA with Levene’s test for homogeneity of variances and Tukey’s HSD post hoc analysis was used to compare mean hospitalization days across diagnostic categories.

### 2.7. Analysis of Comorbidities and Complications

The prevalence of comorbidities and major complications was expressed as frequencies and percentages.

Associations between comorbidities or complications and demographic variables, respectively, age group, sex and year of admission were tested using Chi-square (χ^2^) tests for independence, Bootstrapping (1000 samples, percentile 95% CI) was used to enhance robustness. Crosstabulations provided cell distributions and percentage estimates. Cramer’s V was used as an effect size measure (small = 0.1, medium = 0.3, large = 0.5).

### 2.8. Analysis of Liver Disease Severity

The distribution of Child–Pugh classes (A, B, C) was analyzed in relation to etiology and demographic factors using cross-tabulation and Chi-square tests. To identify independent predictors of advanced disease (e.g., Child–Pugh class C), we performed binary logistic regression. Predictor variables included sex, age group, year of first hospitalization, cumulative hospital days, key comorbidities, and complications. Model fit was evaluated using the Omnibus test, −2 Log Likelihood, Cox and Snell R^2^, Nagelkerke R^2^, and the Hosmer-Lemeshow test. Results are presented as odds ratios (ORs) with 95% CIs.

### 2.9. Correlation and Longitudinal Analyses

Spearman’s rank correlation was applied to explore monotonic associations between hospitalization burden and clinical/demographic variables, including age, sex, etiology, comorbidities, and complications. According to the binary nature of the data, Spearman’s coefficient was performed. Bootstrapping (1000 samples, 95% CI) ensured robustness.

To evaluate longitudinal changes in hepatic function, Wilcoxon matched-pairs signed-rank tests were performed on paired Child–Pugh scores (baseline vs. final evaluation) for 2358 observations. This nonparametric approach accounted for the ordinal scale and potential ties in the data. Missing values were treated pairwise.

### 2.10. Cluster Analysis

To identify clinically meaningful patient subgroups, K-means clustering was performed. The algorithm was set to evaluate 5 clusters (K = 5) using a maximum of 10 iterations and a convergence criterion of 0, with distances saved for subsequent analysis. The decision to use K = 5 was based on iterative testing and evaluation of cluster stability, interpretability, and clinical relevance. Preliminary testing with K ranging from 3 to 7 indicated that 5 clusters achieved optimal separation with minimal within-cluster variance and maximal clinical interpretability, balancing overfitting and underfitting. The K-means algorithm was executed without updating initial cluster centers iteratively, and initial ANOVA and cluster distance matrices were examined to confirm separation between clusters. Convergence was achieved after 6 iterations, with minimal changes in cluster centers (maximum absolute coordinate change = 0.000), confirming stability. Cluster assignments were evaluated using distances from cluster centers, ensuring appropriate membership. Cluster profiles were characterized by the presence or absence of maximum clinical manifestations across all variables.

## 3. Results

### 3.1. Cohort Characteristics and Age Distribution by Liver Disease Etiology

Descriptive statistics for age distribution were calculated for the full cohort (*N* = 2359), stratified by primary diagnosis: CHC (*n* = 969), ALH (*n* = 929), NALC (*n* = 388), and ALC (*n* = 73).

**Figure 1:**
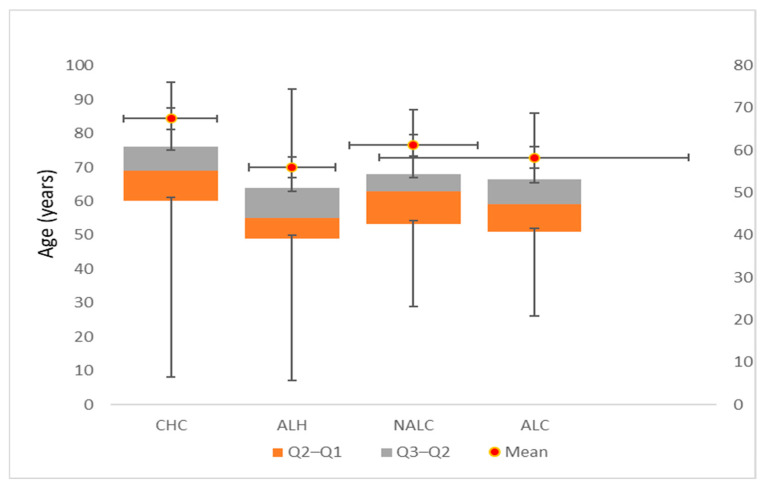
Age distribution by diagnosis with median, mean, and 95% confidence intervals. Box—interquartile range (25th–75th percentile); Line inside box—median; Whiskers—minimum and maximum values; Orange rectangles—Q2-Q1, Gray rectangles—Q3-Q2; Orange dot—mean; Error bar on orange dot—95% CI of the mean. CHC = chronic hepatitis C; ALH = hepatitis associated with alcohol; NALC = non-alcoholic cirrhosis; ALC = cirrhosis associated with alcohol.

Mean age was highest among CHC patients (67.48 ± 12.32 years) and lowest among ALH patients (56.01 ± 10.99 years). Median age ranged from 55 years in ALH to 69 years in CHC. Interquartile ranges (IQR) indicated moderate variability across diagnostic categories (CHC 60–76 years; ALH 49–64 years; NALC 53.25–68 years; ALC 51–66.5 years). Minimum and maximum age spanned from 27 years in ALH to 95 years in CHC. Bootstrap-derived 95% CIs confirmed the robustness of the mean age estimates for each group (Figure 1). Age distributions for CHC, ALH, and NALC significantly deviated from normality (Kolmogorov–Smirnov and Shapiro–Wilk tests, *p* < 0.05), while the ALC distribution did not (Shapiro–Wilk *p* = 0.435).

### 3.2. Age-Related Epidemiological Patterns of Liver Disease Etiologies

**Figure 2:**
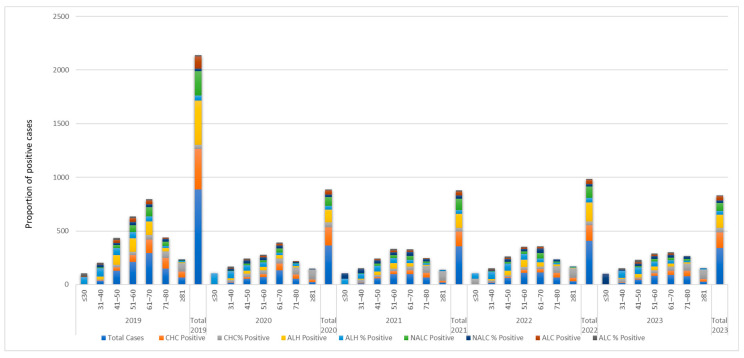
Distribution of primary diagnosis by age group and year (2019–2023); CHC = chronic hepatitis C; ALH = hepatitis associated with alcohol; NALC = non-alcoholic cirrhosis; ALC = cirrhosis associated with alcohol.

Analysis of disease distribution across age groups and study years revealed distinct epidemiological patterns for each etiology (Figure 2, Table 1).

The distribution of CHC positivity varied markedly across age groups and years. Across the pooled dataset (2019–2023) CHC prevalence increased steadily with age: only 10% of patients in the ≤30 stratum was CHC positive whereas the proportion reached 87.1% among patients ≥81 years.

This ascending pattern was consistent within each calendar year. Younger patients (≤30 years) showed very low CHC proportions, frequently null or close to zero, while the highest rates were repeatedly observed in the 71–80 and ≥81 categories. During 2019, 65.1% of patients were included in the age group 71–80 and 81.8% belongs to the age group ≥81, respectively. Year-specific pooled prevalence ranged from 35% (2022) to 46% (2020).

Pearson’s chi-square tests showed a strong association between age group and CHC status across all years (*p* < 0.001). The ordinal Gamma coefficient was consistently positive (overall γ = 0.535, *p* < 0.001), indicating a strong monotonic increase in CHC probability with age. A few cells in the youngest age categories contained expected counts <5; however, given the large total sample size, these did not affect the overall interpretation (Table 1 and Tables S1–S3).

Analysis of ALH distribution across the five consecutive years (2019–2023) revealed a distinct age-related trend. In 2019, the highest proportion of ALH cases was observed in the 31–50 years age groups (51.8–79.4%), while prevalence progressively decreased in older age groups, reaching only 10.6% in patients ≥ 81 years (Figure 2). Similar trends persisted across subsequent years, with a notable concentration of cases in middle-aged adults (41–60 years) and reduced prevalence among the very young (≤30 years) and elderly (≥81 years). The total ALH prevalence over the study period was 40.4%, with significant differences among age groups each year (Pearson χ^2^ = 40.15–132.35, *p* < 0.001). Negative Gamma values (−0.473 to −0.571) indicate a negative ordinal association between age and probability of ALH, confirming that older age groups are progressively less affected (Table 1 and Tables S4–S6).

The distribution of NALC over the same five years showed relatively stable prevalence across age groups, with moderate peaks in middle-aged adults (51–70 years, 22–42.6%) and lower occurrence in the youngest and elderly (≥81 years, 3.7–7.6%). Total NALC prevalence over the study period was 25.6% (Figure 2). The Pearson χ^2^ test revealed significant age-related differences in most years (2019, 2020, 2022, 2023; *p* < 0.05), but not in 2021 (*p* = 0.099). Gamma coefficients were close to zero (overall γ = −0.080), indicating minimal ordinal association between age and NALC prevalence (Table 1 and Tables S7–S9).

The distribution of ALC across 2019–2023 revealed lower overall prevalence compared to NALC, with 11% of total cases (Figure 2). Prevalence was highest in younger adults ≤ 30 years in 2019 (33.3%) and generally concentrated in the age group of 41–60 years across most years (10–22.9%). The Pearson χ^2^ analysis showed significant differences in 2019, 2022, and 2023 (*p* < 0.05), while other years did not reach significance. Gamma values were consistently negative (−0.337 overall), indicating a moderate inverse ordinal association between age and ALC prevalence, suggesting lower risk in older age groups (Table 1 and Tables S7–S9).

### 3.3. Temporal Trends in Hospitalization Duration (2019–2023)

**Figure 3:**
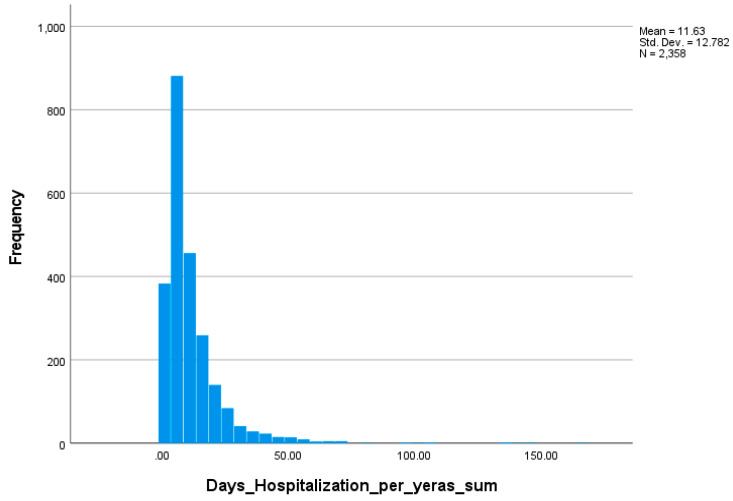
Distribution of annual hospitalization days (2019–2023) showed extreme positive skew and high kurtosis.

The distribution of annual hospitalization days was markedly right-skewed and leptokurtic across all study years (Shapiro–Wilk *p* < 0.001; Skewness: 2.33–6.18; Kurtosis: 7.28–66.00), indicating that most patients had short stays while a small number accounted for disproportionately long hospitalizations (Figure 3). The persistent right-skewed distribution across all years supports the use of a GLM with Gamma distribution and log-link function in subsequent analyses of temporal analysis of hospitalization (Table 2).

A significant temporal trend was observed in mean hospitalization duration. The mean length of hospitalization days decreased steadily from 13.80 ± 15.71 days in 2019 to 9.10 ± 7.95 days in 2023. This decline occurred while the median length of stay remained relatively stable (7–9 days), and the range of hospitalization days narrowed considerably from 1 to 147 days in 2019 to 1–62 days in 2023, respectively. Distributional parameters indicated strong right-skewness (skewness = 2.33–6.18) and leptokurtosis (kurtosis = 7.28–66.00), confirming the presence of infrequent yet extreme hospitalization episodes. The 95% CIs progressively shifted downward (12.76–14.83 in 2019 vs. 8.25–9.95 in 2023), supporting a statistically meaningful decline in mean hospitalization days (Table S13).

### 3.4. Hospitalization Burden Stratified by Diagnosis and Temporal Trends

#### 3.4.1. Diagnosis-Specific Hospitalization Duration

Analysis of annual hospitalization days revealed significant differences in healthcare burden across liver disease etiologies (Table 2).

Patients with liver cirrhosis, whether ALC or NALC, experienced the longest hospitalizations. Specifically, those with ALC had the highest mean hospitalization duration (17.66 ± 12.96 days, median = 14 days, IQR = 14 days), followed by patient with NALC (16.37 ± 14.34 days, median = 11 days, IQR = 14 days). In contrast, patients with ALH or CHC had shorter hospitalizations, with ALH mean of 13.04 ± 12.02 days (median = 9, IQR = 11), and CHC mean of 10.38 ± 10.14 days (median = 7, IQR = 9). Distributions were positively skewed in all categories, reflecting subsets of patients with extended hospital stays, and were leptokurtic, especially in non-cirrhotic groups (Tables S14–S17).

This gradient was further illustrated by percentile analysis. The 75th percentile for hospitalization days was 13 for CHC, 16 for ALH, 21 for NALC, and 23 for ALC, demonstrating a clear escalation in hospitalization burden with disease severity, from non-cirrhotic to cirrhotic stages.

#### 3.4.2. Temporal Trends and Stabilization of Hospital Stays

A significant downward trend in mean hospitalization days was observed from 2019 to 2023 across all diagnostic categories (Table 2). Across categories, the mean decreased from 13.25 ± 12.82 days in 2019 to 9.10 ± 7.92 days in 2023. IQR gradually narrowed over time, from 11 days in 2019 to 6 days in 2023, indicating a decrease in both the variability and the frequency of extreme, prolonged hospitalizations over time. Notably, the reduction in variance and SD values toward 2023 indicates an overall temporal stabilization of hospitalization burden, with fewer extreme cases observed in recent years.

Despite this overall trend, the relative burden between diagnoses persisted. The GLM revealed a statistically significant Year × Diagnosis interaction (*p* < 0.05), confirming that temporal changes in hospitalization duration was not uniform across etiologies. For instance, while all groups showed a decline, ALC patients maintained the longest stays throughout the study period.

#### 3.4.3. Statistical Modeling and Distributional Analysis

The distributions of hospitalization days were strongly right-skewed (Skewness > 2) and leptokurtic (Kurtosis > 7) for all categories and years (Table 2), validating the use of a Gamma-distributed models for analyses.

The largest year-on-year increase occurred among ALC patients (+3.7 days, 2021–2023), whereas CHC and ALH remained relatively constant over time. NALC showed intermediate but highly variable hospitalization profiles, reflecting heterogeneity in disease severity and comorbidity burden.

**Figure 4:**
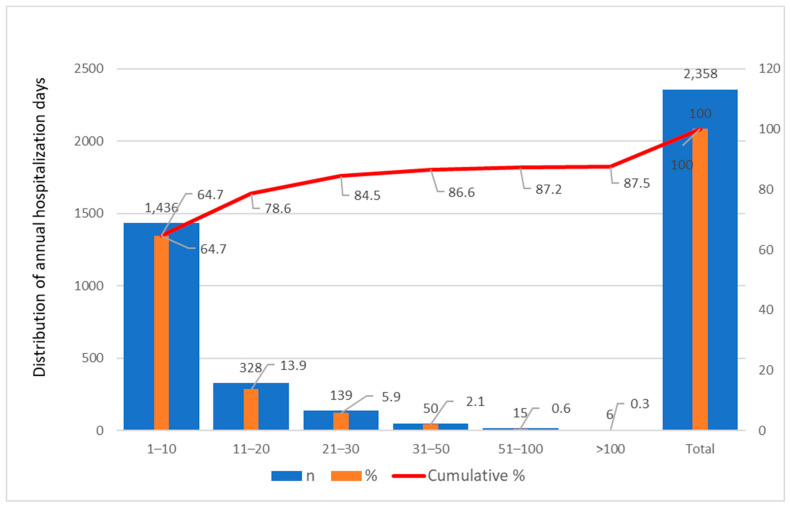
Distribution and cumulative frequency of total hospitalization days per age group.

The total number of hospitalization days per patient ranged from 1 to 60.82 days, with a mean of 11.35 ± 10.75 days. The frequency distribution for the entire cohort confirmed that most hospitalizations were short, with 64.7% of hospitalizations lasting between 1 and 10 days, while extreme hospitalization durations above 50 days were rare, representing less than 1.5% of cases (Figure 4). The 95th percentile was 33 days, and the 99th percentile was 60.82 days, justifying the capping of extreme values to improve model stability (Table S18).

### 3.5. Predictors of Hospitalization Duration: Generalized Linear Model Analysis

To identify independent predictors of hospitalization duration, a GLM with a Gamma distribution and log-link function was fitted. The model included primary diagnosis, Child–Pugh score, sex, and age as fixed effects. The full model was statistically significant (Likelihood Ratio χ^2^ = 559.37, df = 7, *p* < 0.001), indicating that these variables jointly explained a significant portion of the variance in hospitalization days. Goodness-of-fit statistics (Deviance/df = 0.601, Pearson χ^2^/df = 0.712) confirmed the model was appropriate for the positively skewed outcome data.

Parameter estimates from the GLM are presented in Table 3. CHC was set as the reference diagnostic category. The analysis revealed that all other liver disease diagnoses were associated with a significant increase in hospitalization duration compared to CHC. The strongest effect was observed for NALC (*B* = 0.940, *p* < 0.001), followed by ALH (*B* = 0.750, *p* < 0.001) and ALC (*B* = 0.626, *p* < 0.001). This indicates that patients with cirrhotic and alcoholic etiologies experienced substantially longer hospital stays than those with CHC.

Among other covariates, older age was a significant, though modest, predictor of longer hospitalization (*B* = 0.004, *p* = 0.003), while neither sex (*B* = 0.040, *p* = 0.312) nor Child–Pugh score (*B* = 0.007, *p* = 0.738) demonstrated a statistically significant association with hospitalization duration in this model.

### 3.6. Distinct and Persistent Sex-Based Etiological Patterns

**Figure 5:**
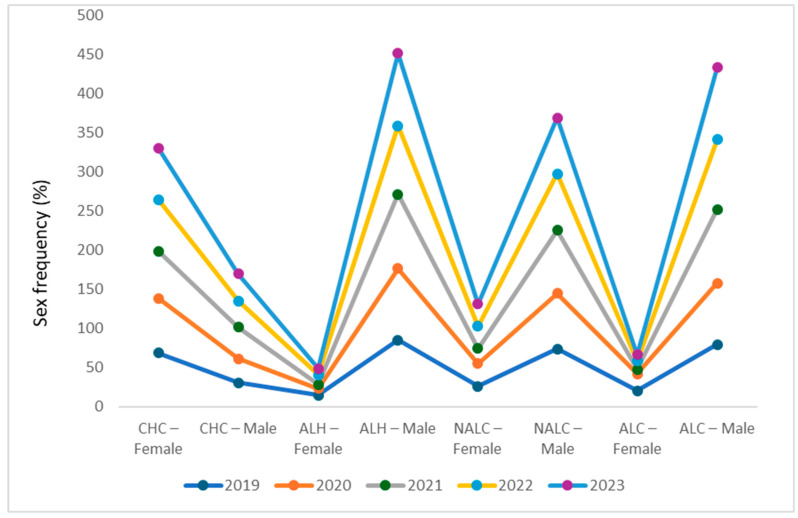
Sex distribution by diagnostic category in the unique-ID cohort over the five consecutive years 2019–2023. All gender differences were statistically significant (χ^2^ test, *p* < 0.05), except 2022–2023 for non-alcoholic cirrhosis.

The study cohort comprising 2359 patients, showed a male predominance (64%, *n* = 1510) over females (36%, *n* = 849). To investigate sex-based epidemiological patterns, we analyzed the association between sex and primary diagnosis for each year from 2019 to 2023 using Pearson’s chi-square tests. All statistical assumptions were met, with no expected cell counts below 5. In addition, the linear-by-linear association statistic was computed where appropriate to assess ordered associations across sex categories (Figure 5).

Across the five years the association between sex and disease occurrence was highly significant for the major comparisons (all overall *p* < 0.001). Two contrasting and persistent patterns were revealed. A female predominance was observed for CHC. In every year, females constituted 60% to 70% of CHC cases, with annual chi-square statistics ranging from χ^2^ = 91.3 to 268.7 (all *p* < 0.001), indicating that females were substantially over-represented among cases coded as CHC relative to males.

Conversely, a pronounced male predominance was evident for alcohol-related liver diseases. Males accounted for more than 85% of ALH cases (annual χ^2^ range: 53.5 to 169.2; all *p* < 0.001) and over 78% of ALC cases (overall χ^2^ = 53.85, *p* < 0.001).

NALC showed a weaker but often significant male predominance in the earlier years (2019–2021; χ^2^ = 17.44, 5.87 and 7.08, respectively; *p* ≤ 0.015). However, the association attenuated in 2022–2023 (χ^2^ = 2.06, 2.80; *p* = 0.151 and 0.094), indicating that the sex difference for NALC was not consistently sustained in the most recent years (Tables S19–S22).

### 3.7. Child–Pugh Classification and Predictors

#### 3.7.1. Univariate Associations Between Etiology and Disease Severity

In the cohort of CHC patients, Child–Pugh class distribution showed that the majority of patients were classified as Class A (67.6%, *n* = 1594), followed by Class B (17.2%, *n* = 405) and Class C (15.2%, *n* = 358). Statistical analysis revealed that 41.1% of patients had CHC and the association between Child–Pugh class and CHC status was strong (Pearson χ^2^ = 426.813, *p* < 0.001). Class A included 55.6% CHC patients, whereas Classes B and C had 10.4% and 11.5%, respectively (Table 4).

Among the cohort, 40.4% had a history of ALH. There was a significant association between Child–Pugh class and ALH (Pearson χ^2^ = 191.932, *p* < 0.001). Class A patients were predominantly alcohol-free, whereas higher proportions of ALH were observed in Classes B and C.

In the studied cohort, 25.6% had documented NALC, with a significant association between Child–Pugh class and cirrhosis etiology (χ^2^ = 82.691, *p* < 0.001). Class A included 19.9% NALC, while Classes B and C showed 37.8% and 36.9%, respectively (Table 4).

ALC was also significantly associated with Child–Pugh class (χ^2^ = 38.981, *p* < 0.001). Among patients without ALC, 91.7% were in class A, 84.2% in class B, and 82.2% in class C. Conversely, patients with ALC were more frequently observed in advanced stages, representing 15.8% and 17.9% of classes B and C, respectively. This trend underscores the impact of alcohol-related liver injury on the progression of hepatic dysfunction (Tables S23–S26).

#### 3.7.2. Multivariate Predictors of Child–Pugh Class

To identify independent predictors of disease severity, we constructed binary logistic regression models for each Child–Pugh class (Table 5).

For Child–Pugh A, the model was significant (Omnibus χ^2^(26) = 634.11, *p* < 0.001) and explained 23.6–32.9% of the variance. CHC was a strong negative predictor of Class A (OR = 0.17, *p* < 0.001), meaning CHC patients were much less likely to be in severe classes. The presence of PH (OR = 3.08, *p* < 0.001), HE (OR = 1.62, *p* = 0.001), and EV (OR = 1.48, *p* = 0.002) were significant positive predictors, confirming their association with worse baseline disease severity.

For Child–Pugh B and C, the models for advanced disease were statistically significant but had more limited predictive power (Nagelkerke R^2^ ≈ 0.19). CHC remained a significant positive predictor for both Class B (OR = 6.03, *p* < 0.001) and Class C (OR = 3.05, *p* < 0.001), indicating that among patients with advanced disease, CHC etiology was common. The presence of PH was a strong negative predictor for both Class B (OR = 0.46, *p* < 0.001) and Class C (OR = 0.35, *p* < 0.001), likely due to its near-universal presence in severe cirrhosis, reducing its power to discriminate between classes B and C. HRS was a specific negative predictor for Class C (OR = 0.50, *p* < 0.001).

The logistic regression model for Child–Pugh A was significant with Omnibus χ^2^ (26) = 634.11, *p* < 0.001, indicating that the predictors collectively distinguished Child–Pugh A from non-A patients. The model explained 23.6–32.9% of variance (Cox and Snell R^2^ = 0.236; Nagelkerke R^2^ = 0.329). The Hosmer-Lemeshow test indicated some misfit (χ^2^ = 54.843, df = 8, *p* < 0.001), suggesting that certain subgroups were not well predicted, possibly due to rare categories or complex interactions (Table 6).

Overall, the model correctly classified 73.6% of cases. Child–Pugh B prevalence was 17.2% (*n* = 405). The initial null model correctly classified 82.8% of cases, driven by most non-B patients. The final model correctly classified 82.7% of cases (Table 6), showing high specificity for non-B patients. Sensitivity for Child–Pugh B cases was limited, reflecting the imbalance in group sizes (Tables S27–S29).

Child–Pugh C prevalence was 15.2% (*n* = 358). The initial null model correctly classified 84.8% of cases, primarily reflecting non-C patients. The final model correctly classified 85.2% of cases. Sensitivity for Child–Pugh C was 3.1%, indicating limited predictive ability for this subgroup, while specificity for non-C cases was 99.9% (Table 6).

#### 3.7.3. Demographic and Temporal Distributions of Disease Severity

Analysis of demographic factors revealed that male sex was significantly associated with more severe disease (χ^2^ = 137.76, *p* < 0.001), with males comprising a larger proportion of Classes B (81.7%) and C (79.6%) compared to Class A (56.0%) (Table 7). Disease severity also varied by age (χ^2^ = 329.47, *p* < 0.001), with the highest prevalence of advanced classes (B and C) observed in patients aged 51–70 years. A significant temporal shift was noted (χ^2^ = 25.55, *p* = 0.012), with the proportion of Class A being highest in 2019 and a more even distribution of advanced classes across subsequent years.

The bootstrap analyses confirmed the stability of these proportion estimates, with 95% CIs indicating robust representation of disease severity in this cohort.

### 3.8. Prevalence of Comorbidities and Complications

**Figure 6:**
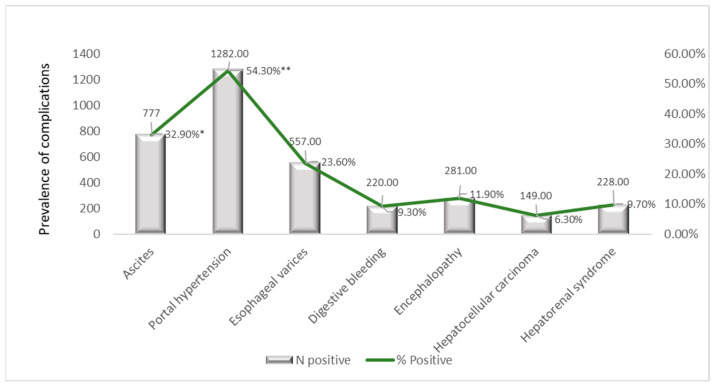
Prevalence of complications in the unique ID cohort; * Prevalence ≥ 25%—high epidemiological significance; ** Prevalence > 50%—clinically central to disease pathophysiology.

In the unique ID cohort, the prevalence of comorbidities and complications varied markedly across conditions. As presented in Figure 6, PH was the most prevalent complication, affecting over half of the patients (54.3%), followed by ascites, present in 32.9% of the cohort, consistent with the pathophysiological centrality of PH in advanced liver disease. EV were observed in 23.6% of patients, reflecting sequelae of elevated portal pressure.

Complication’s indicative of hepatic decompensation was less common, HE being presents in 11.9% and DB in 9.3% of patients. HRS and HCC were rare, affecting 9.7% and 6.3% of patients, respectively. Ob was documented in only 6.1% of patients and CVAs were the least frequent comorbidity, occurring in 5.8% of the cohort.

**Figure 7:**
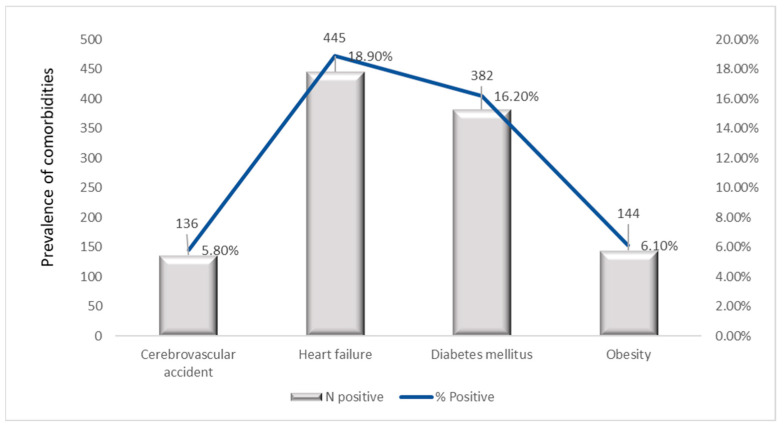
Prevalence of comorbidities and complications in the unique ID cohort.

Cardiometabolic comorbidities were less frequent, with HF present in 18.9% and DM in 16.2% of patients, highlighting a moderate metabolic burden within this population (Figure 7).

The overall prevalence of CVA was 5.8%, with a mean frequency of 0.07 ± 0.53 per patient (Table 8). The prevalence was marginally higher in patients with CHC (9%) compared to those with alcohol-related liver disease (ALH: 4%; ALC: 5%).

A univariate analysis of covariance (UNIANOVA) was performed to assess the effects of liver disease etiology on CVA frequency, with year of diagnosis as a covariate. While the overall model was statistically significant (F(14, 2344) = 2.27, *p* = 0.005), its explanatory power was minimal (adjusted R^2^ = 0.007). Critically, none of the individual liver disease diagnoses, CHC (*p* = 0.829), ALH (*p* = 0.097), NALC (*p* = 0.838), or ALC (*p* = 0.607), were significant independent predictors of CVA occurrence. No significant interaction effects were observed. This indicates that the etiology of the underlying liver disease is not a major determinant of CVA risk in this cohort.

Levene’s test indicated a significant violation of homogeneity (F(12, 2346) = 10.294, *p* < 0.001), suggesting unequal error variances across groups. Despite this, the UNIANOVA model was retained due to the large and balanced sample structure, with age group weighting applied to correct potential variance bias.

The full factorial model was statistically significant with F(14, 2344) = 2.266, *p* = 0.005, *η*^2^ = 0.013 (Adjusted R^2^ = 0.007). This indicates that approximately 1.3% of the variance in CVAs was explained by the combined effect of diagnostic categories and covariates (Table S30).

#### 3.8.1. Stratification of Comorbidities per Years

Analysis of non-liver comorbidities over the five-year study period revealed that most conditions remained stable, with the notable exception of HF. The overall and year-specific prevalences are detailed in Table 9.

The prevalences of CVA (3.9–7.6%), DM (14.2–18.4%), and Ob (4.2–7.5%) showed no statistically significant variation over time (CVA: χ^2^ = 8.79, *p* = 0.066; Diabetes: χ^2^ = 4.05, *p* = 0.400; Ob: χ^2^ = 9.14, *p* = 0.058). This stability suggests a consistent background level of these metabolic and vascular conditions within the hospitalized liver disease population.

In contrast, the prevalence of HF demonstrated significant temporal dynamics (χ^2^ = 20.71, *p* < 0.001). It was highest at the beginning of the study period in 2019 (21.7%) and declined to its lowest point in 2023 (11.7%). This marked decrease highlights HF as the most dynamic comorbidity in this cohort (Table S31).

Collectively, approximately 25–30% of patients exhibited at least one major cardiovascular or metabolic comorbidity, underscoring the need for integrated multidisciplinary management in patients with chronic liver disease.

#### 3.8.2. Age-Stratified Comorbidities Prevalence

A strong age-related pattern was observed for all analyzed comorbidities (Table 10). CVA prevalence increased markedly with age, from 0% in patients aged ≤30 years to 33.8% among those aged 61–70 years. HF exhibited a similar trend, with the highest prevalence of 31.2% in the 61–70-year group and 52.4% among patients aged ≥81 years. DM showed a progressive age-dependent rise, peaking at 35.1% in the 61–70-year group. Obesity reached its maximum prevalence among middle-aged and older-aged adults, with 27.5% in individuals aged 71–80 years. Chi-square analyses were statistically significant for all comorbidities (*p* < 0.001), confirming robust associations between age and comorbidity prevalence. These findings indicate that cerebrovascular, cardiovascular, and metabolic complications increase with advancing age, underscoring the cumulative burden of multimorbidity in elderly patients with chronic liver disease (Table S32).

Although the analyses primarily focused on age-related patterns, temporal assessment revealed consistent trends across the five-year period. The prevalence of HF and DM displayed gradual increases over time, in line with the progressive nature of chronic metabolic and cardiovascular disease. The aging of the hospitalized population contributed to the observed upward trends in overall comorbidity burden.

#### 3.8.3. Sex-Based Distribution of Comorbidities

Sex-stratified analyses revealed statistically significant differences in the prevalence of all four comorbidities (Table 11). The prevalence of CVA was significantly higher among females (8.4%) compared to males (4.3%), χ^2^ = 16.474, *p* < 0.001, Cramér’s V = 0.083 (small effect). Similarly, HF was markedly more frequent in females (28.2%) than in males (13.6%), χ^2^ = 74.739, *p* < 0.001, Cramér’s V = 0.178 (small-to-moderate effect). For DM, prevalence was also higher in females (20.0%) compared to males (14.0%), χ^2^ = 14.338, *p* < 0.001, Cramér’s V = 0.078 (small effect). Likewise, obesity was more prevalent among females (9.0%) than males (4.5%), χ^2^ = 18.762, *p* < 0.001, Cramér’s V = 0.089 (small effect). Among all comorbidities, HF demonstrated the strongest association with sex (Cramér’s V ≈ 0.18), while the remaining conditions showed smaller but statistically significant associations (all *p* < 0.001). These findings indicate that female patients exhibit a greater overall burden of cardiovascular and metabolic comorbidities, potentially impacting both the clinical trajectory and prognosis of chronic liver disease (Table S33).

#### 3.8.4. Temporal Trends in Liver-Related Complications (2019–2023)

The analysis of major liver-related complications revealed distinct temporal trends over the five-year study period (Table 12). These trends can be broadly categorized into improving, fluctuating, and stable patterns.

Two complications showed substantial and sustained improvements. The prevalence of DB fell markedly from 9.9% in 2019 to 3.8% in 2023 (χ^2^ = 38.86, *p* < 0.001). Similarly, the prevalence of HCC decreased from 8.8% to 5.0% (χ^2^ = 16.47, *p* = 0.002).

Several complications demonstrated non-linear trajectories, peaking in the middle of the study period. The prevalence of ascites peaked at 38.8% in 2021 before declining to 32.6% in 2023 (χ^2^ = 12.25, *p* = 0.016). HE rose notably to 19.1% in 2022 before partially receding to 12.6% in 2023 (χ^2^ = 30.74, *p* < 0.001). Both PH and EV reached their highest prevalence in 2022 (61.1% and 32.3%, respectively) before a pronounced decrease in 2023. In contrast, the prevalence of HRS remained stable throughout the study period, ranging from 8.7% to 10.8% (χ^2^ = 0.99, *p* = 0.912).

#### 3.8.5. Prevalence of Liver-Related Complications by Age Group

The burden of liver-related complications demonstrated a clear and significant relationship with patient age, revealing two distinct patterns, complications that peaked in mid-life and those that were age-invariant, respectively (Table 13).

Complications associated with PH and progressive fibrosis showed a strong, inverted U-shaped relationship with age. The prevalence of PH, ascites, and EV increased through middle age, peaking in the 51–70 years groups, and subsequently declined in the oldest patients (≥71 years). Similarly, DB and HRS, while less prevalent overall (<10%), also demonstrated significant variation with age (both *p* < 0.05), with the highest occurrence in middle-aged cohorts.

In contrast, the prevalence of HE and HCC was not significantly associated with age (*p* = 0.182 and *p* = 0.516, respectively). This suggests that the risk for these specific complications is driven more by factors such as metabolic status, liver disease etiology, and comorbidities than by chronological age itself (Table S34).

#### 3.8.6. Sex-Stratified Prevalence of Major Hepatic Complications

A pronounced sex-based disparity was observed, with male patients demonstrating a significantly higher prevalence of nearly all major hepatic complications compared to females (Table 14).

The most substantial difference was identified for PH, which affected 66.8% of males versus 32.2% of females (χ^2^ = 263.21, *p* < 0.001; Cramér’s V = 0.334), representing a strong effect size. Males also exhibited a significantly higher prevalence of ascites (38.3% vs. 23.4%; V = 0.152), HE (14.6% vs. 7.1%; V = 0.112), HRS (11.7% vs. 6.0%; V = 0.093), and EV (26.6% vs. 18.4%; V = 0.093). In contrast, the prevalence of DB did not differ significantly between sexes, and HCC showed only a non-significant trend toward higher prevalence in males.

Bootstrap analyses confirmed the robustness of these associations (Table S35). The consistent pattern across multiple complications indicates a systematically higher burden of advanced liver disease and its sequelae in male patients.

Bootstrap estimates confirmed the stability of the Chi-square results, with narrow 95% CIs and no significant sampling instability detected.

#### 3.8.7. K-Means Clustering of Patient Profiles Based on Complications

**Figure 8:**
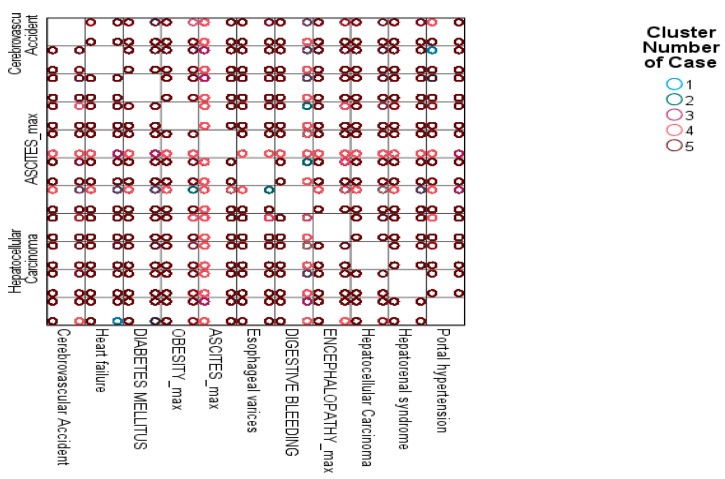
Clinical Phenotype Identification Using K-means Clustering (K = 5).

K-means clustering was applied to 2359 patients using 11 key clinical variables represented by to identify distinct comorbidity patterns. Five discrete clusters were identified, each exhibiting unique clinical characteristics (Figure 8).

Cluster 1 comprised patients with a high prevalence of CVAs, HF, DM, and Ob. This cluster represents a population with multiple metabolic and cardiovascular comorbidities, indicative of high cardiometabolic risk.

Cluster 2 included patients primarily presenting with ascites, EV, DB, HE, HRS, and PH, reflecting severe decompensated liver disease.

Cluster 3 PH was characterized by HE, PH, and EV, but with minimal or absent metabolic comorbidities, representing advanced liver dysfunction without significant cardiovascular or metabolic involvement.

Cluster 4 was defined by the presence of ascites and HRS with sporadic cardiovascular comorbidities, indicative of intermediate liver decompensation with renal involvement.

Cluster 5 consisted of patients with HCC in addition to ascites, EV, and PH, representing advanced liver malignancy accompanied by portal hypertensive complications (Table S36).

### 3.9. Hospitalization Patterns in the Multiple ID Cohort

#### 3.9.1. Hospitalization Frequency Stratification by Diagnostic

Analysis of hospitalization patterns revealed distinct healthcare utilization profiles across different liver disease etiologies (Table 15 and Table S37). Patients with CHC and NALC had the highest mean number of hospitalizations (3.99 and 4.17, respectively). Both groups exhibited highly right-skewed distributions (Skewness > 3.0), indicating that a small subset of patients within these etiologies accounted for a disproportionately high number of readmissions, with maximum hospitalization counts reaching 19–31.

In contrast, patients with alcohol-related liver diseases demonstrated lower healthcare utilization. Those with ALH and ALC had lower mean hospitalization frequencies, reaching 2.86 and 3.10, respectively. While their distributions were also right skewed, the maximum number of admissions per patient was lower (max 17 for ALH, 15 for ALC) compared to the CHC and NALC groups.

#### 3.9.2. Patient-Level Hospitalization Stratification by Age Group

Analysis of hospitalization patterns across age groups revealed a complex, non-linear relationship between age and healthcare utilization in this liver disease cohort (Table 16 and Table S38).

Therefore, patients aged ≤30 years experienced a mean of 2.12 hospitalizations (median = 2; range 1–4), while the 31–40 and 41–50 age groups had mean hospitalizations of 2.77 and 3.24, respectively. Hospitalization frequency peaked in the 51–60 years group (mean = 4.38; median = 3; range 1–19) before decreasing slightly in the 61–70 (mean = 3.66) and 71–80 age groups (mean = 3.33). Notably, patients aged ≥81 years exhibited the highest mean hospitalization frequency (6.04), with substantial variability (SD = 9.95; range 1–31), reflecting a small subset of individuals with frequent admissions. Interquartile ranges were generally moderate (3–5 hospitalizations), but skewness values indicated consistently right-skewed distributions, particularly among older patients.

#### 3.9.3. Descriptive Statistics of Hospitalization Frequency by Sex and Diagnostic Group

A total of 4 341 hospitalization records were analyzed, including 1582 females (36.4%) and 2759 males (63.6%). The mean number of hospitalizations was higher in females (4.19 ± 0.13) than in males (3.63 ± 0.07), with 95% bootstrap CIs of 3.92–4.47 and 3.49–3.77, respectively, indicating a statistically meaningful difference. Despite similar median hospitalization counts (median = 2 for both sexes), the higher mean in females reflects a subset of patients with multiple admissions (Table 17 and Table S39).

Females exhibited greater variance (28.55) and SD = 5.34 than males with a variance of 13.90 and SD = 3.73, respectively, as well as a slightly larger interquartile range (4 vs. 3), confirming higher heterogeneity in hospitalization frequency. Both distributions were positively skewed (3.18 for females, 2.08 for males) and leptokurtic (11.72 for females, 4.27 for males), reflecting right-tailed distributions with extreme hospitalization values more prevalent among females. Percentile analysis further highlighted sex-specific differences: the 75th percentile was 5 hospitalizations in females versus 4 in males, and the 95th percentile was 14 versus 12, respectively. Extreme values also supported these findings, with the maximum number of hospitalizations reaching 31 in females and 19 in males. Overall, females demonstrated both higher average hospitalization frequency and greater variability, indicating a small subset of high-utilization patients.

#### 3.9.4. Hospitalizations Across Liver Disease Subtypes

The mean number of hospitalizations per patient differed substantially among the groups. NALC patients exhibited the highest mean number of hospitalizations (4.92 ± 4.48), followed by CHC (3.99 ± 5.39), ALC (3.10 ± 2.36), and ALH (2.86 ± 2.72) (Table 18).

Levene’s test indicated that variances were significantly heterogeneous among groups (F = 78.02, *p* < 0.001), confirming that the assumption of homogeneity of variance was violated. Therefore, interpretation of the ANOVA results considers this variance heterogeneity.

Despite the heterogeneity of variances, a one-way ANOVA revealed highly significant differences in hospitalization frequency across diagnostic groups (F(3, 4336) = 49.42, *p* < 0.001). This indicates that the type of liver disease is a strong determinant of the frequency of hospital admissions.

**Figure 9:**
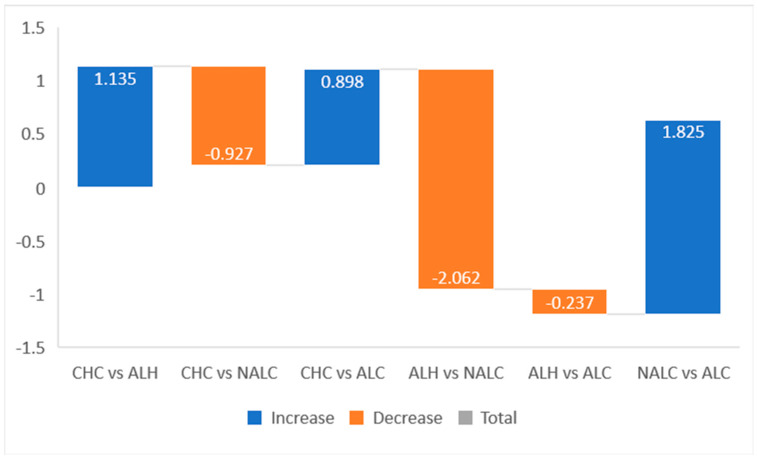
Pairwise comparisons of the number of hospitalizations between diagnostic groups using Tukey’s HSD post hoc test. CHC—chronic hepatitis C; ALH—hepatitis associated with alcohol; NALC—non-alcoholic cirrhosis; ALC—cirrhosis associated with alcohol.

Tukey’s HSD post hoc tests were conducted to examine pairwise differences between diagnostic groups. Significant differences (*p* < 0.05) were observed for most comparisons, except ALH versus ALC (Figure 9).

**Figure 10:**
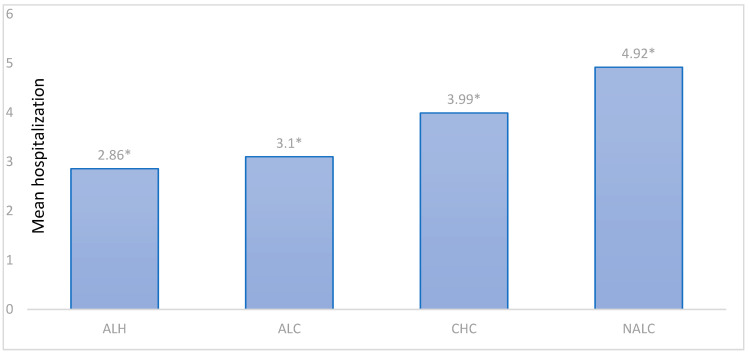
Homogeneous subsets of diagnostic groups based on the number of hospitalizations (Tukey HSD, α = 0.05). CHC—chronic hepatitis C; ALH—hepatitis associated with alcohol; NALC—non-alcoholic cirrhosis; ALC—cirrhosis associated with alcohol. * Groups marked differ significantly from other groups in homogeneous subsets (Tukey HSD, α = 0.05).

These results highlight that NALC patients were hospitalized significantly more frequently than patients in all other groups, reflecting a higher clinical burden. CHC patients also exhibited significantly more hospitalizations than ALH patients but fewer than NALC patients. No statistically significant difference was found between ALH and ALC (*p* = 0.830), suggesting similar hospitalization patterns in these alcohol-related liver diseases. Based on Tukey HSD, homogeneous subsets for the number of hospitalizations were identified (Figure 10). ALH patients constituted the group with the lowest hospitalization rates, while NALC formed the highest subset. CHC and ALC occupied intermediate positions, consistent with their mean values and post hoc significance.

#### 3.9.5. Correlation Analysis

Significant correlations (*p* < 0.05, two-tailed) were observed between days of hospitalizations and several clinical predictors. Spearman correlation analysis demonstrated significant associations between hospitalization frequency and clinical variables. Positive correlations were observed with PH (r = 0.176, *p* < 0.01), ascites (r = 0.206, *p* < 0.01), and NALC (r = 0.206, *p* < 0.01), indicating that patients with advanced or decompensated liver disease experienced more frequent admissions. Negative correlations were observed with ALH (r = −0.133, *p* < 0.01), AH (r = −0.122, *p* < 0.01), and HF (r = −0.072, *p* < 0.01), suggesting that hospitalizations were primarily driven by hepatic rather than cardiovascular conditions. Age correlated positively with comorbidities such as hypertension and HF but negatively with Child–Pugh score (r = −0.172, *p* < 0.01), indicating younger patients had more severe hepatic dysfunction. Sex was strongly associated with disease type (CHC r = −0.521, ALH r = 0.331, both *p* < 0.01), reflecting sex-specific etiologies of liver disease (Table 19 and Table S40).

### 3.10. Longitudinal Child–Pugh Score Analysis

The Related-Samples Wilcoxon Signed-Rank Test assessed changes in Child–Pugh scores between two time points. A statistically significant difference was observed (test statistic = 40,110; standardized Z = 2.818; *p* = 0.005), indicating a shift in the distribution of scores over time. Among 2358 patients (one missing), 215 exhibited positive differences, 156 negative differences, and 1987 ties, consistent with the limited variability inherent in the three-category ordinal scale (Table S41).

**Figure 11:**
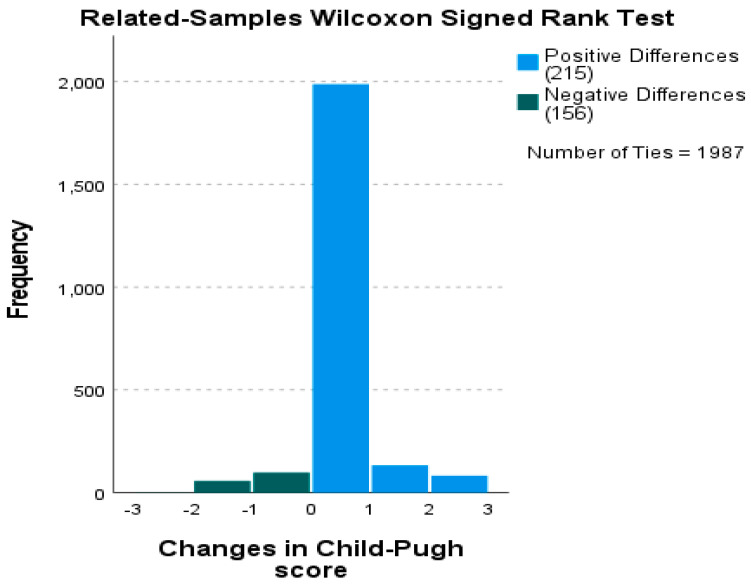
Histogram of paired differences in Child–Pugh scores between two time points.

A detailed descriptive analysis of the paired differences is crucial for interpreting this result (Figure 11). The histogram of differences revealed a total of 215 positive differences (where the second measurement was higher than the first), 156 negative differences (where the second measurement was lower than the first), and a substantial 1987 ties (where there was no change between the two measurements). This high number of ties is consistent with the limited variability inherent in the three-category ordinal scale.

**Figure 12:**
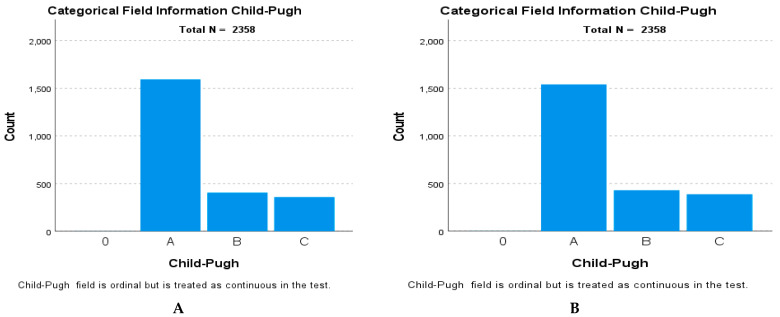
Baseline Characteristics and Primary Statistical Outcome. (**A**) Baseline distribution of the study cohort (*N* = 2358) by Child–Pugh classification. (**B**) Hypothesis test summary for the related samples Wilcoxon signed rank test.

The baseline distribution of the Child–Pugh score categories for the entire cohort at one of the time points is presented in Figure 12A. This figure provides the essential context for the sample’s initial disease severity profile. The test results indicated a significant difference between the two measurements. The test statistic was 40,110.000, resulting in a standardized test statistic of 2.818 and an asymptotic significance (*p*-value) of 0.005. Given that the *p*-value was less than the predetermined α = 0.05, the null hypothesis, which stated that the median of the differences equals zero, was rejected (Figure 12B).

### 3.11. Pandemic vs. Non-Pandemic Periods and Hospitalization Burden

Analysis of hospitalization days from 2019 to 2023 demonstrates a clear downward trend across the entire cohort (Table 2). Mean hospitalization duration decreased from 13.80 ± 15.71 days in 2019 to 9.10 ± 7.95 days in 2023, while median stays remained relatively stable (7–9 days). The reduction in both the mean and the interquartile range (IQR: 11 days in 2019 vs. 6 days in 2023) reflects a decrease in the frequency of extreme, prolonged hospitalizations, as further confirmed by a progressive narrowing of 95% confidence intervals (CI: 12.76–14.83 in 2019 vs. 8.25–9.95 in 2023). The right-skewed and leptokurtic distribution of hospitalization days persisted across all years (Skewness: 2.33–6.18; Kurtosis: 7.28–66.00), indicating that extreme values remained rare but influential.

Temporal patterns were consistent across diagnostic categories (CHC, ALH, NALC, ALC), with all showing declining mean hospitalization days. The GLM revealed a statistically significant Year × Diagnosis interaction (*p* < 0.05), confirming that temporal reductions were not uniform: cirrhotic patients (ALC, NALC) consistently experienced longer stays, whereas CHC and ALH patients showed smaller absolute decreases (Table 2, Figure 3 and Figure 4). The variance and SD for hospitalization days decreased toward 2023 across all groups, indicating stabilization of hospitalization burden and fewer extreme cases, independent of pandemic-related fluctuations.

These findings suggest that the observed decline in hospitalization duration was a sustained structural trend rather than a short-term effect of pandemic-related healthcare disruptions. No abrupt discontinuities or rebounds are apparent; the temporal decrease is gradual, with consistent directional patterns across etiologies. Extreme hospitalizations, although present in all years, became less frequent over time, as reflected in percentile analyses (75th percentile: CHC = 13 days, ALH = 16, NALC = 21, ALC = 23). Therefore, the pandemic period (2020–2021) does not appear to have introduced confounding spikes or reductions in hospitalization burden, supporting the robustness of the observed temporal trend.

## 4. Discussion

### 4.1. Analytical Framework: Integrating Cross-Sectional and Longitudinal Perspectives

A key methodological strength of this study is the creation of two complementary analytical datasets, which together provide a multidimensional view of chronic liver disease. The unique-ID cohort (*N* = 2359) offered a robust cross-sectional snapshot, enabling precise estimation of the baseline prevalence of comorbidities, complications, and the initial healthcare burden across different disease etiologies. This design was crucial for minimizing duplication bias and establishing reliable associations between demographic factors and disease manifestations at presentation.

Simultaneously, the longitudinal sub-cohort of patients with multiple admissions provided critical insights into the dynamic progression of liver disease. This dataset allowed us to track the evolution of disease severity, the emergence of new complications, and patterns of healthcare utilization over time. The integration of these two perspectives reveals that while cross-sectional analysis identifies baseline risk profiles, longitudinal follow-up is essential to understand the trajectory of patients, particularly those prone to recurrent decompensation and high hospital resource use.

The longitudinal analysis was necessarily restricted to patients with at least two hospital admissions, as repeated measurements are required to evaluate within-patient change over time. Consequently, this sub-cohort represents individuals with recurrent hospital contact, who are more likely to have unstable disease courses, recurrent decompensation, or higher overall healthcare utilization.

This design introduced an inherent selection bias toward patients with more advanced or clinically active liver disease. As a result, the observed changes should be interpreted as reflecting disease evolution among patients prone to rehospitalization rather than the natural history of the entire chronic liver disease population.

In order to mitigate this limitation, cross-sectional analyses based on the unique-ID cohort were conducted in parallel, providing an unbiased estimate of baseline disease severity, comorbidity burden, and complication prevalence at first admission.

This dual-cohort framework not only strengthens the epidemiological validity of our prevalence estimates but also illuminates the natural history of chronic liver disease, offering a powerful model for identifying high-risk patient phenotypes for targeted clinical management and resource allocation.

### 4.2. Demographics and Hospitalization Patterns

In the unique ID cohort, patient age and annual hospitalization burden differed across the four primary liver disease categories. CHC patients were the oldest (mean 67.5 years) with moderate median annual hospitalizations (7 days), reflecting long-standing disease and accumulated comorbidities. Similarly, Genowska et al. [24] highlight a decrease in hospitalizations in young CHC patients associated with increased access to HVC screening and treatment. The study conducted by Talić Drlje et al. [25] in Bosnia and Herzegovina shows that anti-HCV-positive individuals are predominantly young men with lower levels of education, more prone to unemployment, unmarried and coming from urban environments.

ALH patients were younger (mean 56.0 years) but experienced the highest hospitalization rates (median 9 days), suggesting disease activity or management intensity drives inpatient care. ALC patients were relatively young (mean 58.3 years) with the lowest hospitalization rates (median 6 days), potentially reflecting cohort size, early mortality, or healthcare-seeking behavior.

The study by Julien et al. [26] indicates an increase in the incidence of alcohol-related liver disease, especially in the young and female population. The authors also predict an increase in the age-standardized incidence of alcoholic cirrhosis from 9.9 cases per 100,000 patients/year in 2019 to 17.5 cases per 100,000 patients/year in 2040 unless current trends are controlled and new policies emerge worldwide.

In Europe, North America and Latin America, alcohol consumption remains the main cause of cirrhosis (over 60% of cirrhosis cases), while in Southeast Asia, Africa and the eastern Mediterranean regions, hepatitis C virus infection is the main cause [3].

NALC patients had intermediate age (mean 61.2 years) with median hospitalizations of 8 days, consistent with chronic disease progression requiring periodic interventions [27].

The growth of the global population, especially the increase in the elderly population with risk factors for NALC (Ob and DM) has contributed to the acceleration of disease progression for this age segment [28].

Age-stratified analyses revealed distinct patterns by etiology. CHC positivity increased with age, peaking in the oldest cohorts, consistent with historical exposure and delayed diagnosis, while ALH showed a negative association with age, reflecting peak incidence in productive-age population of 15–44 years. NALC exhibited a more uniform age distribution, suggesting metabolic and lifestyle factors beyond age alone influence disease occurrence. In contrast, ALC was concentrated on younger and middle-aged adults, highlighting the cumulative effect of alcohol consumption. These results are in accordance with other results presented [29,30,31,32,33,34]. However, these patterns should not underscore the importance of age-tailored screening and preventive strategies according to etiology.

Metabolic dysfunction-associated steatotic liver disease (MASLD) is the most common chronic liver disease, affecting more than 30% of adults and 7–14% of youths globally. MASLD and its advanced form of metabolic dysfunction-associated steatohepatitis (MASH) can progress to liver cirrhosis and hepatocellular carcinoma [35]. Despite its growing burden, effective therapies for MASLD and MASH remain limited. For HCC patients with high tumor burden, HAIC demonstrates comparable efficacy to TACE-HAIC both in combination with TKIs and ICIs. Therefore, HAIC should be the preferred local therapeutic strategy over TACE-HAIC in HCC patients with high tumor burden [36].

Hospitalization dynamics in the multiple ID cohort mirrored these findings. The study by Hasjim et al. [37] indicates a higher hospitalization rate in patients with decompensated cirrhosis (77.3 hospitalizations per 100 patients per year) compared to patients with compensated cirrhosis. However, regardless of the etiology of cirrhosis, frailty plays a vital role in shaping hospitalization rates, extending to compensated cirrhosis as well.

A potential concern is whether the observed changes in hospitalization burden may reflect healthcare disruptions during the COVID-19 pandemic. However, the temporal patterns observed do not indicate a transient pandemic-driven artifact. Changes in hospitalization metrics evolved gradually across the study period and persisted beyond the pandemic years, without evidence of a systematic rebound following the resumption of routine healthcare services. Importantly, trends were directionally consistent across etiological groups, arguing against diagnosis-specific access bias. These observations suggest that the reported temporal changes more likely reflect structural shifts in disease management and hospitalization practices rather than short-term pandemic-related effects. 

Sex differences were evident across liver disease etiologies. CHC was more prevalent in females (60–70%), probably since they undergo medical examinations more frequently, whereas ALH and ALC were predominantly male also associated with alcohol consumption. However, in Europe, there is a higher prevalence of HCV infections in men compared to women, indicating a male-female ratio of 1.6:1 in 2020, with a slight downward trend in recent years [38,39].

Analyzing the gender distribution of patients diagnosed with CHC in Bosnia and Herzegovina, Talić Drlje et al. [25] indicated a higher frequency in male patients (85.5%) compared to female patients (14.5%).

Similar results of gender distribution in CHC patients were observed in India by Sharma et al. [40], while in China the study by Niu et al. [41] indicates an almost equal ratio between men and women. Zhou et al. [42] attribute an increase in the incidence rate of HCV infection to low educational level, which constitutes a major risk factor.

The increased incidence of ALH and ALC in men is associated with increased alcohol consumption [23]. However, the biological and hormonal vulnerability of women to alcohol-induced liver damage should be emphasized [35]. Thus, the lower activity of the gastric enzyme alcohol dehydrogenase leads to higher blood alcohol levels in women compared to men even after an equivalent alcohol consumption. Also, the activity of estrogen is considered, which increases liver inflammation, which accelerates the progression of liver fibrosis. After menopause, when plasma estrogen levels decrease, the liver is more vulnerable to the cumulative effects of alcohol consumption [23,35,43].

NALC initially showed male predominance, which diminished in later years, potentially reflecting evolving metabolic risk in females. In a comparative study of clinico-socio-demographic characteristics among two diverse Indian population, Sahoo et al. [44] was observed that the gender distribution MASLD is more prevalent among males (71.6%) than the females (28.4%). Arora et al. [45] found that most of the MASLD patients were married males residing in nuclear families in semi-urban to urban areas, reflecting their accessibility to resources.

Globally, Teng et al. [11] estimated a prevalence of non-alcoholic fatty liver disease in adults of 32%, higher in men (40%) compared to women (26%). In Europe, Riazi et al. [46] indicated an incidence of NALC of 32.6%, like the estimate by Le et al. [47] of approximately 30.9%.

Sex-specific patterns extended to comorbidities, with females exhibiting higher prevalence of HF, CVAs, DM, and Ob, while males had higher burden of PH, ascites, and HRS. These observations highlight the necessity of sex-stratified preventive and management strategies.

### 4.3. Child–Pugh Classification

In this study, liver disease severity was evaluated using the Child–Pugh score, which incorporates both laboratory and clinical parameters. While MELD could have been calculated from the available laboratory data, it was not used as the primary measure because it is mainly intended for short-term mortality prediction and transplant prioritization [48]. The use of Child–Pugh allowed a more comprehensive assessment of functional hepatic reserve and clinical status, providing meaningful context for interpreting hospitalization trends, complications, and overall disease burden in this cohort [49].

Child–Pugh analysis demonstrated that Class A predominated (67.6%), consistent with early-stage liver disease being most common in tertiary care registries, while Classes B and C represented more advanced dysfunction. This evolution demonstrates a deterioration in liver function and an increased severity of liver disease [50].

Logistic regression identified CHC as a strong predictor of progression to Classes B and C, emphasizing viral etiology as a key determinant of disease severity. PH, EV, HE, and HRS were also associated with Child–Pugh classification, reflecting the pathophysiological interplay of decompensated liver disease. However, the ability of this score to predict these types of complications is questionable and still under research [51]. The disadvantage of the Child–Pugh score is the subjectivity of the classification of ascites and HE [52]. However, the ability of the Child–Pugh score to predict the evolution of ascites (the most common complication in decompensated cirrhosis) is superior to other scores used (MELD—the model for end-stage liver disease or MELD-Na—sodium-adjusted variant) [51].

Age and sex further influenced disease severity, with older males more likely to present with advanced stages, reinforcing the need for demographic- and etiology-specific management protocols. Similar results were obtained by Baker et al. [53] which highlight the increased incidence of Child–Pugh class C in male patients aged 46–55 (82%).

The Wilcoxon signed-rank test revealed a statistically significant change in Child–Pugh scores over the monitored period, primarily driven by a minority (~15.7%) of patients whose scores worsened. This finding underscore both the relative stability of liver function in most patients and the value of Child–Pugh as a sensitive metric for detecting progression in high-risk subgroups.

### 4.4. Comorbidities and Complications

Comorbidities showed clear age-dependent patterns: CVAs and HF increased markedly in older adults (≥61 years), diabetes was most prevalent in middle-aged patients (51–70 years), and Ob accumulated in older adults.

The heart-liver axis is well known in medical practice because the liver disease is a significant risk factor for cardiovascular outcomes [54]. Knowledge of these interactions is very important in the effective management of patients with heart or liver diseases to improve overall prognosis and therapy [55].

Also, people with chronic liver disease, especially those with cirrhosis, are at increased risk of ischemic and hemorrhagic stroke. Therefore, the management of acute stroke includes the selection of antithrombotic agents, antihypertensive agents, the use of statins, and the management of coagulopathy [56].

The complex relationship between CLD, diabetes and inflammation is essential for understanding the pathophysiological mechanisms that trigger it to develop more effective prevention and treatment approaches to improve quality of life and reduce the burden on the healthcare system [57].

Sex-specific disparities were also noted, with females exhibiting higher cardiovascular and metabolic comorbidity burden. For the effective management of these disorders, lifestyle modification is recommended, combining sustained physical activity with smoking cessation, a balanced diet and weight loss [55]. Temporal trends in complications revealed peaks in ascites and HE in 2021–2022, while HCC prevalence decreased, likely to reflect improved surveillance and preventive care. Correlations between PH and ascites, as well as between varices and DB, reinforced known pathophysiological relationships.

The Spearman correlation analysis highlighted that advanced hepatic dysfunction, particularly NALC, ascites, and PH, was the main driver of repeated hospitalizations, whereas ALH, ALC, and CHC showed negative or weaker correlations with hospitalization frequency, likely reflecting acute presentation, mortality, or lower follow-up duration. These findings underscore that PH and its complications constitute the predominant disease burden in this cohort, whereas metabolic and vascular comorbidities are present but less prevalent. The aggregation method (maximum function across hospitalizations) ensures that these prevalences reflect ever-occurrence, providing a cumulative estimate of lifetime disease burden per patient.

### 4.5. Cluster-Defined Phenotypes and Their Clinical Significance

The application of K-means clustering to a large cohort of 2359 patients with chronic liver disease enabled the identification of five clinically distinct phenotypes that capture different trajectories of disease progression and systemic involvement. Rather than classifying patients through conventional single-parameter assessments, the multidimensional profiling incorporated eleven major clinical complications, allowing differentiation of patient subgroups according to metabolic burden, liver decompensation, and cancer-related manifestations. This approach highlights the heterogeneity of chronic liver disease and underscores the need for personalized care strategies aligned with individual risk patterns.

Cluster 1 included patients with a high cumulative prevalence of Ob, DM, CVA, and HF. This phenotype reflects a patient population dominated by cardiometabolic disease, consistent with the growing epidemiological link between metabolic syndrome and chronic liver pathology [58,59]. The clustering results support existing evidence indicating that metabolic dysfunction not only accelerates the development of nonalcoholic fatty liver disease and nonalcoholic steatohepatitis [60,61], but also increases cardiovascular mortality, often exceeding liver-related mortality in these patients [62]. Thus, management of this cluster should expand beyond hepatic parameters, integrating intensive control of metabolic risk factors, cardiovascular monitoring, and early interventional strategies such as weight reduction, glycemic control, and lipid management.

Cluster 2, characterized by high rates of ascites, EV, DB, HE, HRS, and PH, represented severely decompensated liver disease [63]. This subgroup reflects the classic natural history of advanced PH in cirrhosis, progressing from fluid retention to multi-organ dysfunction [4,64]. The internal consistency of these co-occurring complications confirms the ability of unsupervised clustering to capture real biological interactions. Importantly, this cluster corresponds to patients with the highest short-term mortality risk [65], requiring aggressive liver-directed management, continuous hemodynamic assessment, and prioritization for transplantation evaluation

Cluster 3 presented an intermediate phenotype in which PH and HE were dominant, but metabolic comorbidities were relatively absent. These findings suggest patients with advanced hepatic insufficiency emerging in the absence of significant systemic metabolic disease. Such a profile may correspond to patients whose disease stems from viral hepatitis, alcohol-related liver injury, or autoimmune etiologies rather than metabolic dysfunction. Management in this group should focus on stabilizing hepatic performance, reducing ammonia burden, preventing variceal bleeding, and evaluating candidacy for disease-modifying therapies [66,67].

The profile of Cluster 4 indicated liver decompensation primarily defined by ascites and hepatorenal involvement, with only sporadic cardiovascular comorbidities. This subgroup may represent a transitional clinical state between compensated cirrhosis and fulminant failure, in which renal dysfunction becomes a key inflection point. This phenotype emphasizes the prognostic significance of renal impairment in cirrhosis, which is independently associated with increased mortality, reduced functional reserve, and poorer eligibility for interventional therapies. Early detection, sodium restriction, vasoconstrictive pharmacotherapy, and timely evaluation for transplantation may improve outcomes in this group [68,69].

Finally, Cluster 5 consisted of patients with HCC alongside PH, EV, and ascites. Unlike the previous clusters, this subgroup reflects malignancy-driven progression superimposed on an already compromised hepatic environment. This phenotype aligns with contemporary clinical observations showing that tumor development in cirrhosis frequently overlaps with portal hypertensive complications, worsening tolerance to both surgical and systemic anticancer therapies. Management strategies for patients in this cluster must therefore balance oncological benefit against hepatic reserve, incorporating transplant eligibility, locoregional therapies, and individualized multidisciplinary decision-making [70,71,72].

Taken together, these five clusters illustrate a progressive continuum of liver disease phenotypes, from metabolically driven systemic pathology to portal hypertensive deterioration and malignancy-associated terminal decline. Importantly, the unsupervised learning model captured clinically meaningful groupings without prior assumptions, reinforcing the validity of machine learning approaches in stratifying complex chronic diseases. These phenotypes enable improved prognostic stratification, support dynamic patient monitoring, and highlight opportunities for precision medicine. Incorporating such data-driven classifications into clinical workflow may facilitate better treatment prioritization, resource allocation, and individualized care pathways that improve both survival and quality of life in patients with advanced liver disease.

### 4.6. Clinical Implications

This study provides robust evidence of diagnosis-, age-, and sex-specific patterns in hospitalization, comorbidities, complications, and liver disease severity. CHC emerged as a major driver of advanced disease, while NALC patients exhibited the highest hospitalization burden and metabolic comorbidity load.

NALC, characterized by excessive fat accumulation in the liver, without significant alcohol consumption, is considered the most well-known form of liver damage in the world [16].

Alcohol-related etiologies (ALH and ALC) presented lower hospitalization frequencies but significant risk for early decompensation in males. These findings support targeted screening, early intervention, and resource allocation strategies tailored to etiology, demographic characteristics, and risk profiles. Multidisciplinary management, age- and sex-specific monitoring, and proactive outpatient care are crucial to reduce disease progression and healthcare utilization [73].

The dual-cohort design, rigorous statistical analyses, and large sample size strengthen the validity of these conclusions. Limitations include reliance on hospital-reported cases, potential underrepresentation of asymptomatic patients, and absence of longitudinal laboratory or imaging data for predictive modeling. Detailed clinical data and complete treatment history were not consistently available for all patients, which may limit certain analyses. Nonetheless, the study provides a comprehensive framework for understanding chronic liver disease dynamics and guiding clinical and public health interventions.

## 5. Conclusions

The large, comprehensive hospital-based Romanian cohort provides a detailed epidemiological and clinical portrait of chronic liver disease, revealing critical, interdependent determinants of patient presentation and outcomes that demand a paradigm shift in clinical management.

Our findings expose fundamental demographic divergences. CHC is a disease of older females (60–70% of cases), while alcohol-related liver diseases (ALH, ALC) exhibit a pronounced male predominance (>80%). NALC, carrying the highest hospitalization burden, presents a more uniform age distribution, with its sex bias attenuating in recent years, suggesting evolving epidemiological trends.

These etiologies dictate distinct healthcare trajectories. Cirrhotic patients, particularly those with NALC, endure the longest and most frequent hospitalizations. A significant decline in hospitalization over the study period signals improved management, yet the persistent disparity between disease groups underscores the need for etiology-specific care pathways.

Crucially, a profound sex-based disparity extends beyond etiology to disease manifestation. Females bear a heavier burden of cardiometabolic comorbidities (e.g., HF, DM), whereas males develop more severe hepatic complications (e.g., PH, ascites). This divergence necessitates sex-tailored prevention and monitoring strategies.

The progression to advanced disease, predicted by CHC, PH, and HRS, is further influenced by age and sex. Our cluster analysis validates the disease’s heterogeneity, moving beyond traditional classifications to define five distinct clinical phenotypes, from high cardiometabolic risk to severe decompensation, providing a robust framework for personalized, complication-driven care.

The management of chronic liver disease must evolve to reflect the complex interplay of etiology and demographic profile. Future strategies must prioritize etiology-specific screening, sex-tailored intervention, and phenotype-based risk stratification to enable early, multidisciplinary management, ultimately mitigating disease progression and reducing the significant associated healthcare burden.

## Figures and Tables

**Table 1:** Annual and overall association measures (Chi-Square and Gamma) for CHC, ALH, NALC, and ALC, across 2019–2023 (*N* = 2359).

Primary Diagnosis	Indicator	2019	2020	2021	2022	2023	Overall (2019–2023)
CHC	Pearson χ^2^	124.91	48.25	61.81	98.13	71.04	382.24
	*p*-value	<0.001	<0.001	<0.001	<0.001	<0.001	<0.001
	Gamma	0.518	0.484	0.521	0.571	0.596	0.535
ALH	Pearson χ^2^	132.35	47.34	40.15	70.41	51.00	323.04
	*p*-value	<0.001	<0.001	<0.001	<0.001	<0.001	<0.001
	Gamma	−0.537	−0.508	−0.473	−0.571	−0.545	−0.525
NALC	Pearson χ^2^	18.96	14.75	10.68	29.02	12.95	56.98
	*p*-value	0.004	0.022	0.099	0.000	0.044	0.000
	Gamma	−0.040	−0.213	−0.111	0.016	−0.117	−0.080
ALC	Pearson χ^2^	27.40	5.85	11.18	15.03	14.88	60.58
	*p*-value	<0.001	0.440	0.083	0.020	0.021	<0.001
	Gamma	−0.353	−0.232	−0.242	−0.420	−0.422	−0.337
	Valid *N*	888	365	356	409	341	2359

**Table 2:** Temporal dynamics (2019–2023) of annual hospitalization days by diagnostic category (*N* = 2359).

Primary Diagnosis	Year	Mean ± SD	Median	Range	Skewness	Kurtosis	95% CI (Lower–Upper)
CHC	2019	13.80 ± 15.71	9	1–147	3.58	19.40	12.76–14.83
	2020	10.55 ± 11.51	7	1–137	5.28	47.06	9.37–11.74
	2021	10.47 ± 9.17	8	1–62	2.33	7.28	9.52–11.43
	2022	10.97 ± 12.05	8	1–164	6.18	66.00	9.80–12.14
	2023	9.10 ± 7.95	7	1–62	2.73	11.37	8.25–9.95
ALH	2019	14.22 ± 15.88	10	1–147	3.70	18.95	13.18–15.27
	2020	11.87 ± 11.82	8	1–137	5.01	46.10	10.57–13.17
	2021	11.46 ± 9.65	8	1–62	2.61	8.54	10.45–12.47
	2022	12.13 ± 12.47	8	1–164	6.25	65.84	10.93–13.33
	2023	10.11 ± 8.22	7	1–62	2.85	12.15	9.21–11.01
NALC	2019	15.26 ± 16.12	10	1–147	3.75	21.14	14.10–16.42
	2020	12.02 ± 12.10	8	1–137	5.14	48.32	10.70–13.34
	2021	11.73 ± 9.74	8	1–62	2.59	9.22	10.70–12.76
	2022	12.84 ± 12.52	9	1–164	6.34	67.01	11.63–14.05
	2023	10.56 ± 8.31	7	1–62	2.91	12.40	9.66–11.46
ALC	2019	16.81 ± 17.04	11	1–147	3.93	22.68	15.60–18.02
	2020	13.24 ± 13.12	9	1–137	5.22	49.15	11.90–14.58
	2021	12.68 ± 10.11	9	1–62	2.75	9.86	11.61–13.75
	2022	13.97 ± 12.94	9	1–164	6.40	68.12	12.74–15.20
	2023	11.24 ± 8.54	8	1–62	3.02	12.93	10.33–12.15

**Table 3:** Generalized Linear Model (Gamma distribution, Log-Link) predicting annual hospitalization days (*N* = 2359).

Predictor	B *	Std. Error	95% CI	Wald χ^2^	*p*-Value
Intercept	4.245	0.141	3.970–4.521	912.37	<0.001
CHC (yes vs. no)	Reference	-	-	-	-
CHC (no vs. yes)	−0.784	0.062	−0.904–−0.663	162.39	<0.001
ALH (no vs. yes)	−0.750	0.051	−0.850–−0.651	220.10	<0.001
NALC (no vs. yes)	−0.940	0.048	−1.034–−0.846	381.24	<0.001
ALC (no vs. yes)	−0.626	0.052	−0.728–−0.524	144.33	<0.001
Child–Pugh	0.007	0.022	−0.036–0.051	0.112	0.738
Sex (female vs. male)	0.040	0.040	−0.038–0.118	1.021	0.312
Age	0.004	0.001	0.001–0.007	8.776	0.003

* A positive coefficient B indicates an increase in the log of expected hospitalization days compared to the reference category; CHC = chronic hepatitis C; ALH = hepatitis associated with alcohol; NALC = non-alcoholic cirrhosis; ALC = cirrhosis associated with alcohol.

**Table 4:** Distribution of Child–Pugh score by diagnostic (*N* = 2359).

Diagnostic		Child–Pugh A	Child–Pugh B	Child–Pugh C	Total
CHC	No	708 (30.0%)	363 (15.4%)	317 (13.6%)	1389 (59%)
	Yes	886 (37.6%)	42 (1.8%)	41 (1.7%)	969 (41.1%)
ALH	No	1104 (46.8%)	165 (7.0%)	136 (5.8%)	1405 (59.6%)
	Yes	491 (20.8%)	240 (10.2%)	222 (9.4%)	954 (40.4%)
NALC	No	1277 (54.1%)	252 (10.7%)	226 (9.6%)	1756 (74.4%)
	Yes	318 (13.5%)	153 (6.5%)	132 (5.6%)	603 (25.6%)
ALC	No	1463 (91.7%)	341 (84.2%)	294 (82.2%)	2099 (89.1%)
	Yes	132 (8.3%)	64 (15.8%)	64 (17.9%)	260 (11.0%)

CHC = chronic hepatitis C; ALH = hepatitis associated with alcohol; NALC = non-alcoholic cirrhosis; ALC = cirrhosis associated with alcohol.

**Table 5:** Significant logistic regression predictors for Child–Pugh class (*N* = 2359).

Child–Pugh	Predictor	B	SE	Wald	df	*p*	Exp(B)	95% CI (Lower-Upper)
A	CHC	−1.753	0.218	64.834	1	0.000	0.173	0.113–0.266
	EV	0.393	0.124	10.066	1	0.002	1.481	1.162–1.888
	HE	0.483	0.149	10.509	1	0.001	1.620	1.210–2.169
	HRS	0.327	0.160	4.202	1	0.040	1.387	1.014–1.897
	PH	1.124	0.133	71.805	1	0.000	3.078	2.373–3.993
B	Sex	0.192	0.164	1.376	1	0.241	1.211	0.879–1.669
	Year	reference	-	-	-	-	-	-
	CHC	1.797	0.273	43.286	1	0.000	6.031	3.531–10.301
	ALH	0.319	0.194	2.721	1	0.099	1.376	0.942–2.011
	NALC	0.163	0.191	0.730	1	0.393	1.177	0.810–1.710
	ALC	0.172	0.177	0.951	1	0.329	1.188	0.840–1.680
	CVA	0.357	0.328	1.189	1	0.276	1.429	0.752–2.716
	HF	0.146	0.208	0.497	1	0.481	1.158	0.771–1.739
	DM	−0.302	0.174	3.017	1	0.082	0.739	0.525–1.040
	Ob	0.345	0.326	1.124	1	0.289	1.412	0.746–2.673
	EV	−0.484	0.137	12.563	1	0.000	0.616	0.471–0.805
	DB	−0.028	0.197	0.020	1	0.887	0.972	0.660–1.431
	HE	−0.184	0.163	1.269	1	0.260	0.832	0.605–1.146
	HCC	0.076	0.248	0.093	1	0.760	1.079	0.663–1.754
	HRS	0.328	0.191	2.948	1	0.086	1.388	0.955–2.019
	PH	−0.772	0.164	22.106	1	0.000	0.462	0.335–0.638
	Age groups	-	-	5.210	6	0.517	-	-
	Constant	−3.023	0.799	14.332	1	0.000	0.049	-
C	Sex	−0.115	0.170	0.454	1	0.500	0.892	0.639–1.244
	CHC	1.115	0.274	16.607	1	0.000	3.050	1.784–5.215
	ALH	−0.045	0.199	0.051	1	0.822	0.956	0.647–1.413
	NALC	0.042	0.194	0.047	1	0.829	1.043	0.713–1.526
	ALC	−0.128	0.179	0.509	1	0.476	0.880	0.619–1.251
	CVA	0.071	0.335	0.046	1	0.831	1.074	0.558–2.069
	HF	0.331	0.230	2.072	1	0.150	1.392	0.887–2.185
	DM	−0.076	0.188	0.165	1	0.685	0.926	0.641–1.340
	Ob	−0.199	0.313	0.404	1	0.525	0.819	0.443–1.514
	EV	−0.010	0.147	0.005	1	0.943	0.990	0.742–1.319
	DB	−0.302	0.201	2.264	1	0.132	0.739	0.499–1.096
	HE	−0.426	0.165	6.666	1	0.010	0.653	0.473–0.903
	HCC	−0.090	0.263	0.117	1	0.733	0.914	0.546–1.530
	HRS	−0.691	0.170	16.480	1	0.000	0.501	0.359–0.699
	PH	−1.055	0.183	33.325	1	0.000	0.348	0.244–0.498
	Age groups	-	-	7.727	6	0.259	-	-
	Constant	−1.465	0.906	2.617	1	0.106	0.231	-

Non-significant predictors for Child–Pugh A: sex, year of hospitalization, ALH, cirrhosis types, CVA, HF, DM, Ob, DB, HCC, age groups. B—regression coefficients, SE—standard errors, Wald chi-square values, df—degrees of freedom, *p*—significance levels, Exp(B)—odds ratios and CI—95% confidence intervals. Positive coefficients indicate an increased likelihood of severe complications, whereas negative coefficients indicate a protective association. Reference categories are indicated where applicable. Exp(B) values > 1 indicate increased odds of Child–Pugh C, <1 indicate decreased odds.

**Table 6:** Logistic regression classification—Step1.

Observed		Predicted		Corectly Classified (%)
		0.00	1.00	
Child–Pugh A	0.00	451	312	59.1
	1.00	311	1285	80.5
Overall percentage				73.6
Child–Pugh B	0.00	1954	3	99.8
	1.00	405	0	0
Overall percentage				82.7
Child–Pugh B	0.00	1998	3	99.9
	1.00	347	11	3.1
Overall percentage				85.2

**Table 7:** Distribution of Child–Pugh score by sex, year, age group (*N* = 2359).

Variable	Category	Child–Pugh A	Child–Pugh B	Child–Pugh C	Total
Sex	Female	701 (29.7%)	74 (3.1%)	73 (3.1%)	848 (36.0%)
	Male	893 (37.9%)	331 (14.0%)	285 (12.1%)	1510 (64.0%)
Year	2019	638 (27.1%)	141 (6.0%)	107 (4.6%)	887 (37.7%)
	2020	251 (10.6%)	55 (2.3%)	59 (2.5%)	365 (15.5%)
	2021	226 (9.6%)	63 (2.7%)	67 (2.8%)	356 (15.1%)
	2022	249 (10.6%)	90 (3.8%)	70 (3.0%)	409 (17.3%)
	2023	230 (9.8%)	56 (2.4%)	55 (2.3%)	341 (14.5%)
Age group	≤30	4 (0.2%)	3 (0.1%)	2 (0.2%)	10 (0.5%)
	31–40	61 (2.6%)	18 (0.8%)	20 (0.8%)	99 (4.2%)
	41–50	208 (8.8%)	83 (3.5%)	67 (2.8%)	358 (15.2%)
	51–60	352 (14.9%)	106 (4.5%)	118 (5.0%)	576 (24.4%)
	61–70	494 (20.9%)	135 (5.7%)	103 (4.4%)	732 (31.0%)
	71–80	324 (13.7%)	45 (1.9%)	44 (1.9%)	413 (17.5%)
	≥81	151 (6.4%)	15 (0.6%)	4 (0.2%)	170 (7.2%)

**Table 8:** ANOVA Summary for the effects of CHC, ALC and NALC, and year on CVA values.

Source	df	F	Sig.	Partial η^2^	Interpretation
Corrected Model	14	2.266	0.005	0.013	Significant overall model
Intercept	1	9.926	0.002	0.004	Constant effect significant
CHC	1	0.047	0.829	0.000	n.s. *
ALH	1	2.756	0.097	0.001	Trend-level effect
NALC	1	0.042	0.838	0.000	n.s.
ALC	1	0.265	0.607	0.000	n.s.
YEAR_min	1	0.000	0.987	0.000	n.s.
CHC × YEAR	1	0.608	0.436	0.000	n.s.
ALH × YEAR	1	1.675	0.196	0.001	n.s.
ALH × NALC	1	1.810	0.179	0.001	n.s.
ALH × NALC × ALC × YEAR	6	0.541	0.777	0.001	n.s.

* n.s. = not significant (*p* > 0.05); df—degrees of freedom, F-values, Sig.—significance levels, Partial η^2^—partial eta squared. CHC = chronic hepatitis C; ALH = hepatitis associated with alcohol; NALC = non-alcoholic cirrhosis; ALC = cirrhosis associated with alcohol.

**Table 9:** Annual prevalence of comorbidities across 2019–2023 (*N* = 2359).

Comorbidity	Year	Negative *n* (%)	Positive *n* (%)	Total *n*	χ^2^ (df)	*p*-Value
CVA	2019	827 (93.1%)	61 (6.9%)	888	8.793 (4)	0.066
	2020	347 (95.1%)	18 (4.9%)	365		
	2021	341 (95.8%)	15 (4.2%)	356		
	2022	393 (96.1%)	16 (3.9%)	409		
	2023	315 (92.4%)	26 (7.6%)	341		
HF	2019	695 (78.3%)	193 (21.7%)	888	20.710 (4)	<0.001
	2020	296 (81.1%)	69 (18.9%)	365		
	2021	301 (84.6%)	55 (15.4%)	356		
	2022	321 (78.5%)	88 (21.5%)	409		
	2023	301 (88.3%)	40 (11.7%)	341		
DM	2019	735 (82.8%)	153 (17.2%)	888	4.046 (4)	0.400
	2020	298 (81.6%)	67 (18.4%)	365		
	2021	302 (84.8%)	54 (15.2%)	356		
	2022	351 (85.8%)	58 (14.2%)	409		
	2023	291 (85.3%)	50 (14.7%)	341		
Ob	2019	821 (92.5%)	67 (7.5%)	888	9.135 (4)	0.058
	2020	342 (93.7%)	23 (6.3%)	365		
	2021	341 (95.8%)	15 (4.2%)	356		
	2022	393 (96.1%)	16 (3.9%)	409		
	2023	318 (93.3%)	23 (6.7%)	341		

**Table 10:** Summary of key age-stratified statistics (*N* = 2359).

Comorbidity	Age (Years)							Total Prevalence	Chi-Square (*p*-Value)
	≤30	31–40	41–50	51–60	61–70	71–80	≥81		
CVA	0%	1.0%	1.4%	4.9%	6.3%	8.0%	13.5%	5.8%	41.224 (<0.001)
HF	0%	2.0%	3.9%	13.5%	19.0%	29.8%	52.4%	18.9%	240.610 (<0.001)
DM	10.0%	3.0%	10.3%	12.0%	18.3%	25.4%	19.4%	16.2%	59.333 (<0.001)
Ob	12.0%	14.0%	16.0%	20.0%	22.0%	27.5%	15.0%	19.0%	38.500 (<0.001)

Note: Total prevalence represents the proportion of positive cases in the entire cohort.

**Table 11:** Prevalence of comorbidities by sex, χ^2^ tests, and effect sizes (*N* = 2359).

Complication	Group	Count	% (Group N/Subgroup)	χ^2^ (df = 1)	*p*-Value (Two-Sided)	Cramér’s V (√(χ^2^/N))
CVA	Total (*N* = 2359)	136	5.77%	16.474	<0.001	0.083
	Female (*n* = 849)	71	8.36%			
	Male (*n* = 1510)	65	4.31%			
HF	Total (*N* = 2359)	445	18.86%	74.739	<0.001	0.178
	Female (*n* = 849)	239	28.15%			
	Male (*n* = 1510)	206	13.64%			
DM	Total (*N* = 2359)	382	16.19%	14.338	<0.001	0.078
	Female (*n* = 849)	170	20.02%			
	Male (*n* = 1510)	212	14.04%			
Ob	Total (*N* = 2359)	144	6.10%	18.762	<0.001	0.089
	Female (*n* = 849)	76	8.95%			
	Male (*n* = 1510)	68	4.50%			

**Table 12:** Temporal trends in liver-related comorbidities across 2019–2023 (*N* = 2359).

Comorbidity	Year	Negative n (%)	Positive n (%)	Total n	Chi-Square (df)	*p*-Value
Ascites	2019	626 (70.5)	262 (29.5)	888	12.247 (4)	0.016
	2020	247 (67.7)	118 (32.3)	365		
	2021	218 (61.2)	138 (38.8)	356		
	2022	261 (63.8)	148 (36.2)	409		
	2023	230 (67.4)	111 (32.6)	341		
EV	2019	680 (76.6)	208 (23.4)	888	40.050 (4)	<0.001
	2020	276 (75.6)	89 (24.4)	365		
	2021	271 (76.1)	85 (23.9)	356		
	2022	277 (67.7)	132 (32.3)	409		
	2023	298 (87.4)	43 (12.6)	341		
DB	2019	800 (90.1)	88 (9.9)	888	38.860 (4)	<0.001
	2020	326 (89.3)	39 (10.7)	365		
	2021	299 (84.0)	57 (16.0)	356		
	2022	386 (94.4)	23 (5.6)	409		
	2023	328 (96.2)	13 (3.8)	341		
HE	2019	809 (91.1)	79 (8.9)	888	30.742 (4)	<0.001
	2020	331 (90.7)	34 (9.3)	365		
	2021	309 (86.8)	47 (13.2)	356		
	2022	331 (80.9)	78 (19.1)	409		
	2023	298 (87.4)	43 (12.6)	341		
HCC	2019	810 (91.2)	78 (8.8)	888	16.470 (4)	0.002
	2020	346 (94.8)	19 (5.2)	365		
	2021	344 (96.6)	12 (3.4)	356		
	2022	386 (94.4)	23 (5.6)	409		
	2023	324 (95.0)	17 (5.0)	341		
HRS	2019	802 (90.3)	86 (9.7)	888	0.985 (4)	0.912
	2020	331 (90.7)	34 (9.3)	365		
	2021	325 (91.3)	31 (8.7)	356		
	2022	365 (89.2)	44 (10.8)	409		
	2023	308 (90.3)	33 (9.7)	341		
PH	2019	412 (46.4)	476 (53.6)	888	16.075 (4)	0.003
	2020	178 (48.8)	187 (51.2)	365		
	2021	151 (42.4)	205 (57.6)	356		
	2022	159 (38.9)	250 (61.1)	409		
	2023	177 (51.9)	164 (48.1)	341		

**Table 13:** Age-stratified prevalence of major liver-related complications in the study cohort (*N* = 2359).

Complication	Age (Years)							Total Positive (%)	*p*-Value
	≤30	31–40	41–50	51–60	61–70	71–80	≥81		
Ascites	30.0%	26.0%	39.9%	38.7%	36.9%	22.5%	11.2%	777 (32.9%)	<0.001
EV	30.0%	26.0%	31.3%	26.7%	23.1%	18.6%	9.4%	557 (23.6%)	<0.001
DB	0.0%	7.0%	12.8%	9.4%	10.0%	8.0%	4.1%	220 (9.3%)	0.034
HE	0.0%	14.0%	12.6%	13.2%	11.6%	12.3%	5.9%	281 (11.9%)	0.182
HCC	0.0%	3.0%	5.6%	6.4%	6.7%	7.7%	4.7%	149 (6.3%)	0.516
HRS	10.0%	12.0%	8.7%	12.2%	10.1%	8.7%	2.4%	228 (9.7%)	0.013
PH	80.0%	69.0%	72.1%	62.7%	55.1%	37.0%	17.6%	1282 (54.3%)	<0.001

**Table 14:** Prevalence of major hepatic complications by sex and Chi-square association tests (*N* = 2359).

Complication	Female (%)	Male (%)	Total Prevalence (%)	χ^2^ (df = 1)	*p*-Value	Effect Size (Cramer’s V)	Interpretation
Ascites	23.4	38.3	32.9	54.17	<0.001	0.152	Moderate, males > females
EV	18.4	26.6	23.6	20.17	<0.001	0.093	Weak, males > females
DB	8.2	9.9	9.3	1.83	0.176	0.028	n.s.
HE	7.1	14.6	11.9	29.67	<0.001	0.112	Weak, males > females
HCC	5.2	7.0	6.3	2.88	0.090	0.035	n.s. (trend)
HRS	6.0	11.7	9.7	20.33	<0.001	0.093	Weak, males > females
PH	32.2	66.8	54.3	263.21	<0.001	0.334	Strong, males > females

Note: n.s. = not significant (*p* ≥ 0.05); χ^2^ from Pearson test; effect size computed as √(χ^2^/*N*).

**Table 15:** Hospitalization frequency by liver disease etiology (*N* = 4341).

Diagnosis Group	Status	*N*	Mean	Median	SD	Variance	Range	IQR	Skewness	Kurtosis	Min	Max
CHC	No	2718	3.74	2	3.67	13.47	18	4	1.99	3.91	1	19
CHC	Yes	1622	3.99	2	5.39	29.03	30	4	3.20	11.57	1	31
ALH	No	3045	4.25	2	4.88	23.77	30	4	2.75	9.56	1	31
ALH	Yes	1295	2.86	2	2.72	7.41	16	2	2.16	5.08	1	17
NALC	No	3211	3.74	2	4.50	20.25	30	4	2.91	11.11	1	31
NALC	Yes	1129	4.17	3	5.39	29.03	30	6.5	3.20	11.57	1	19
ALC	No	4046	3.89	2	4.50	20.25	30	4	2.91	11.11	1	31
ALC	Yes	294	3.10	2	2.36	5.59	14	2	2.03	4.99	1	15

Abbreviations: SD—Standard Deviation; IQR—Interquartile Range; CHC—chronic hepatitis C; ALH—hepatitis associated with alcohol; NALC—non-alcoholic cirrhosis; ALC—cirrhosis associated with alcohol.

**Table 16:** Summary of hospitalization frequency per patient, by age group (*N* = 4341).

Age Group (Years)	*N* Patients	Mean ± SE	Median	SD	Min–Max	IQR	Skewness	95% CI Mean
≤30	17	2.12 ± 0.26	2	1.05	1–4	2	0.47	1.67–2.63
31–40	158	2.77 ± 0.18	2	2.31	1–9	3	1.40	2.43–3.15
41–50	644	3.24 ± 0.10	2	2.65	1–15	3	1.41	3.04–3.45
51–60	1139	4.38 ± 0.14	3	4.55	1–19	5	1.72	4.14–4.66
61–70	1386	3.66 ± 0.09	2	3.47	1–19	4	1.84	3.47–3.85
71–80	732	3.33 ± 0.14	2	3.65	1–22	3	2.90	3.05–3.59
≥81	265	6.04 ± 0.61	1	9.95	1–31	3	1.93	4.83–7.28

Note: SE = Standard Error; SD = Standard Deviation; IQR = Interquartile Range; 95% CI = 95% Confidence Interval (bootstrap, percentile method).

**Table 17:** Descriptive statistics of hospitalization frequency by sex (*N* = 4341).

Sex	*N*	Mean ± SE	95% CI Mean	Median	5% Trimmed Mean	SD	Variance	Min– Max	Range	IQR	Skewness	Kurtosis
Female	1582	4.19 ± 0.13	3.92–4.47	2	3.31	5.34	28.55	1–31	30	4	3.18	11.72
Male	2759	3.63 ± 0.07	3.49–3.77	2	3.11	3.73	13.90	1–19	18	3	2.08	4.27

**Table 18:** Descriptive statistics of the number of hospitalizations per patient across diagnostic groups (*N* = 2359).

Primary Diagnostic	*N*	Mean * (±SE)	95% CI	Min–Max
CHC	1622	3.99 ± 0.13	3.73–4.26	1–31
ALH	1295	2.86 ± 0.08	2.71–3.01	1–17
NALC	1129	4.92 ± 0.13	4.66–5.18	1–19
ALC	294	3.10 ± 0.14	2.82–3.37	1–15
Total	4340	3.83 ± 0.07	3.70–3.97	1–31

Note: * Means are expressed as mean value ± standard error; CHC—chronic hepatitis C; ALH—hepatitis associated with alcohol; NALC—non-alcoholic cirrhosis; ALC—cirrhosis associated with alcohol.

**Table 19:** Correlation Heatmap of Clinical and Demographic Variables with Statistical Significance Indicators.

Variables	Number_ Hospitalizations	Age_Group	Sex	CHC	ALH	NALC	ALC	Arterial Hypertension	Cerebrovascular Accident	Heart Failure	Diabetes Mellitus	Obesity	Child-Pugh	Ascites	Esophageal Varices	Digestive Bleeding	Encephalopathy	Hepatocellular Carcinoma	Hepatorenal Syndrome	Portal Hypertension
Number_ Hospitalizations	1	−0.048 **	−0.02	−0.066 **	−0.133 **	0.206 **	0.01	−0.122 **	−0.092 **	−0.072 **	−0.038 *	−0.034 *	0.079 **	0.206 **	0.091 **	0.00	0.00	−0.01	0.03	0.176 **
Age_Group	−0.048 **	1	−0.316 **	0.347 **	−0.299 **	−0.01	−0.115 **	0.286 **	0.102 **	0.268 **	0.128 **	0.036 *	−0.172 **	−0.109 **	−0.072 **	−0.039 *	−0.01	0.02	−0.02	−0.226 **
Sex	−0.02	−0.316 **	1	−0.521 **	0.331 **	0.160 **	0.120 **	−0.210 **	−0.063 **	−0.164 **	−0.088 **	−0.061 **	0.220 **	0.160 **	0.048 **	0.01	0.100 **	0.042 **	0.076 **	0.303 **
CHC	−0.066 **	0.347 **	−0.521 **	1	−0.504 **	−0.458 **	−0.208 **	0.369 **	0.072 **	0.281 **	0.160 **	0.100 **	−0.404 **	−0.339 **	−0.113 **	−0.01	−0.132 **	0.01	−0.150 **	−0.543 **
ALH	−0.133 **	−0.299 **	0.331 **	−0.504 **	1	−0.387 **	−0.176 **	−0.160 **	−0.041 **	−0.168 **	−0.087 **	−0.069 **	0.185 **	−0.131 **	0.075 **	0.00	0.066 **	−0.01	0.073 **	0.275 **
NALC	0.206 **	−0.01	0.160 **	−0.458 **	−0.387 **	1	−0.160 **	−0.188 **	−0.03	−0.101 **	−0.057 **	−0.02	0.202 **	0.420 **	0.061 **	0.01	0.081 **	0.03	0.105 **	0.321 **
ALC	0.01	−0.115 **	0.120 **	−0.208 **	−0.176 **	−0.160 **	1	−0.092 **	−0.02	−0.059 **	−0.051 **	−0.040 **	0.088 **	0.159 **	−0.02	−0.02	−0.01	−0.049 **	−0.03	−0.02
Arterial Hypertension	−0.122 **	0.286 **	−0.210 **	0.369 **	−0.160 **	−0.188 **	−0.092 **	1	0.186 **	0.560 **	0.250 **	0.230 **	−0.212 **	−0.268 **	−0.098 **	−0.047 **	−0.031 *	−0.01	−0.079 **	−0.309 **
Cerebrovascular Accident	−0.092 **	0.102 **	−0.063 **	0.072 **	−0.041 **	−0.03	−0.02	0.186 **	1	0.208 **	0.086 **	0.039 *	−0.065 **	−0.087 **	−0.044 **	0.00	0.03	0.00	0.00	−0.089 **
Heart Failure	−0.072 **	0.268 **	−0.164 **	0.281 **	−0.168 **	−0.101 **	−0.059 **	0.560 **	0.208 **	1	0.223 **	0.160 **	−0.154 **	−0.187 **	−0.076 **	−0.03	−0.01	0.00	−0.054 **	−0.238 **
Diabetes Mellitus	−0.038 *	0.128 **	−0.088 **	0.160 **	−0.087 **	−0.057 **	−0.051 **	0.250 **	0.086 **	0.223 **	1	0.125 **	−0.059 **	−0.110 **	−0.052 **	−0.030 *	0.00	0.01	−0.01	−0.126 **
Obesity	−0.034 *	0.036 *	−0.061 **	0.100 **	−0.069 **	−0.02	−0.040 **	0.230 **	0.039 *	0.160 **	0.125 **	1	−0.044 **	−0.074 **	0.00	−0.01	0.02	−0.02	0.00	−0.087 **
Child-Pugh	0.079 **	−0.172 **	0.220 **	−0.404 **	0.185 **	0.202 **	0.088 **	−0.212 **	−0.065 **	−0.154 **	−0.059 **	−0.044 **	1	0.294 **	0.143 **	0.03	0.122 **	−0.01	0.135 **	0.394 **
Ascites	0.206 **	−0.109 **	0.160 **	−0.339 **	−0.131 **	0.420 **	0.159 **	−0.268 **	−0.087 **	−0.187 **	−0.110 **	−0.074 **	0.294 **	1	0.084 **	0.050 **	0.03	0.031 *	0.122 **	0.380 **
Esophageal Varices	0.091 **	−0.072 **	0.048 **	−0.113 **	0.075 **	0.061 **	−0.02	−0.098 **	−0.044 **	−0.076 **	−0.052 **	0.00	0.143 **	0.084 **	1	0.258 **	0.126 **	0.02	−0.030 *	0.208 **
Digestive Bleeding	0.00	−0.039 *	0.01	−0.01	0.00	0.01	−0.02	−0.047 **	0.00	−0.03	−0.030 *	−0.01	0.03	0.050 **	0.258 **	1	0.02	−0.02	−0.01	0.045 **
Encephalopathy	0.00	−0.01	0.100 **	−0.132 **	0.066 **	0.081 **	−0.01	−0.031 *	0.03	−0.01	0.00	0.02	0.122 **	0.03	0.126 **	0.02	1	−0.02	0.00	0.112 **
Hepatocellular Carcinoma	−0.01	0.02	0.042 **	0.01	−0.01	0.03	−0.049 **	−0.01	0.00	0.00	0.01	−0.02	−0.01	0.031 *	0.02	−0.02	−0.02	1	−0.02	−0.01
Hepatorenal Syndrome	0.03	−0.02	0.076 **	−0.150 **	0.073 **	0.105 **	−0.03	−0.079 **	0.00	−0.054 **	−0.01	0.00	0.135 **	0.122 **	−0.030 *	−0.01	0.00	−0.02	1	0.111 **
Portal Hypertension	0.176 **	−0.226 **	0.303 **	−0.543 **	0.275 **	0.321 **	−0.02	−0.309 **	−0.089 **	−0.238 **	−0.126 **	−0.087 **	0.394 **	0.380 **	0.208 **	0.045 **	0.112 **	−0.01	0.111 **	1

Note: Values represent correlation coefficients. Positive values indicate positive correlation, negative values indicate negative correlation. * Correlation is significant at the 0.05 level (*p* < 0.05); ** Correlation is significant at the 0.01 level (*p* < 0.01).

## Data Availability

The original contributions presented in this study are included in the article. Further inquiries can be directed to the corresponding author.

## Supplementary Materials

The following supporting information can be downloaded at https://www.mdpi.com/article/10.3390/jcm15020454/s1: Table S1. Age Groups * Chronic hepatitis C (1 = yes, 0 = no) * YEAR_min Crosstabulation; Table S2. Pearson’s chi-square tests—age group and year; Table S3. Symmetric Measures—age group and year; Table S4. Age Groups * Alcoholic hepatitis (1 = yes, 0 = no) * YEAR_min Crosstabulation; Table S5. Chi-Square Tests—age group and CHC; Table S6. Symmetric Measures—age group and CHC; Table S7. Age Groups * Non-alcoholic cirrhosis * YEAR_min Crosstabulation; Table S8. Chi-Square Tests—NALC and age group; Table S9. Symmetric Measures—NALC and age group; Table S10. Age Groups * Alcoholic cirrhosis (1 = yes, 0 = no) * YEAR_min Crosstabulation; Table S11. Chi-Square Tests—ALC and age group; Table S12. Symmetric Measures—ALC and age group; Table S13. Distribution of annual hospitalization days (2019–2023) showed extreme positive skew and high kurtosis; Table S14. Days of hospitalization per CHC per year; Table S15. Days of hospitalization per ALH per year; Table S16. Days of hospitalization per NALC per year; Table S17. Days of hospitalization per ALC per year; Table S18. Generalized Linear Model (Gamma distribution, log link); Table S19. Sex-Based CHC Patterns; Table S20. Sex-Based ALH Patterns; Table S21. Sex-Based NALC Patterns; Table S22. Sex-Based ALC Patterns; Table S23. Distribution of Child–Pugh score by CHC; Table S24. Distribution of Child–Pugh score by diagnostic ALH; Table S25. Distribution of Child–Pugh score by diagnostic NALC; Table S26. Distribution of Child–Pugh score by ALC; Table S27. Logistic regression predictors for Child–Pugh A; Table S28. Logistic regression predictors for Child–Pugh B; Table S29. Logistic regression predictors for Child–Pugh C; Table S30. The effects of liver disease etiology on CVA frequency; Table S31. Comorbidities per years; Table S32. Comorbidities per age; Table S33. Sex-Based Distribution of Comorbidities; Table S34. Prevalence of Liver-Related Complications by Age Group; Table S35. Sex-Stratified Prevalence of Major Hepatic Complications; Table S36. K-Means Clustering of Patient Profiles Based on Complications; Table S37. Hospitalization frequency stratification by diagnostic; Table S38. Patient-Level Hospitalization stratification by Age Group; Table S39. Descriptive Statistics of Hospitalization Frequency by Sex and Diagnostic Group; Table S40. Spearman correlation; Table S41. Related-Samples Wilcoxon Signed Rank Test Summary.

## Abbreviations

The following abbreviations are used in this manuscript:

CHC	chronic hepatitis C
ALH	hepatitis associated with alcohol
ALC	cirrhosis associated with alcohol
NALC	non-alcoholic cirrhosis
CLD	chronic liver diseases
MASLD	metabolic dysfunction-associated steatotic liver disease
AH	arterial hypertension
CVA	cerebrovascular accident
HF	heart failure
DM	diabetes mellitus
Ob	obesity
EV	esophageal varices
DB	digestive bleeding
HE	hepatic encephalopathy
HCC	hepatocellular carcinoma
HRS	hepatorenal syndrome
PH	portal hypertension
CIs	confidence intervals
GLM	Generalized Linear Model
EMMs	Estimated Marginal Means
ORs	odds ratios
N	number of patients in full cohort
n	number of patients in subcohort
IQR	interquartile range
Df	degrees of freedom
