# Supplementary material for: A Retrospective Analysis of Hepatic Disease Burden and Progression in a Hospital-Based Romanian Cohort Using Integrated Cross-Sectional and Longitudinal Data (2019–2023)
Source: J Clin Med. 2026 Jan 7;15(2):454. doi: 10.3390/jcm15020454 (PMC12842456; doi:10.3390/jcm15020454)
Supplement: Supplementary file 1 [file jcm-15-00454-s001.zip › jcm-4033101-supplementary.pdf]

Supplementary Material

A Retrospective Analysis of Hepatic Disease Burden and Progression in a Hospital-Based Romanian Cohort Using Integrated Cross-Sectional and Longitudinal Data (2019–2023)

Table S1. Age Groups * Chronic hepatitis C (1=yes, 0=no) * YEAR_min Crosstabulation.

YEAR_min				Chronic hepatitis C (1=yes, 0=no) no	yes	Total
2019	Age Groups	≤30	Count	3	0	3
			Expected Count	1.7	1.3	3.0
			% within Age Groups	100.0%	0.0%	100.0%
		31-40	Count	31	3	34
			Expected Count	19.7	14.3	34.0
			% within Age Groups	91.2%	8.8%	100.0%
		41-50	Count	101	30	131
			Expected Count	75.8	55.2	131.0
			% within Age Groups	77.1%	22.9%	100.0%
		51-60	Count	148	64	212
			Expected Count	122.7	89.3	212.0
			% within Age Groups	69.8%	30.2%	100.0%
		61-70	Count	167	126	293
			Expected Count	169.6	123.4	293.0
			% within Age Groups	57.0%	43.0%	100.0%
		71-80	Count	52	97	149
			Expected Count	86.2	62.8	149.0
			% within Age Groups	34.9%	65.1%	100.0%
		≥81	Count	12	54	66
			Expected Count	38.2	27.8	66.0
			% within Age Groups	18.2%	81.8%	100.0%
	Total		Count	514	374	888
			Expected Count	514.0	374.0	888.0

			% within Age Groups	57.9%	42.1%	100.0%
2020	Age Groups	≤30	Count	2	0	2
			Expected Count	1.1	.9	2.0
			% within Age Groups	100.0%	0.0%	100.0%
		31-40	Count	15	4	19
			Expected Count	10.3	8.7	19.0
			% within Age Groups	78.9%	21.1%	100.0%
		41-50	Count	40	16	56
			Expected Count	30.2	25.8	56.0
			% within Age Groups	71.4%	28.6%	100.0%
		51-60	Count	46	26	72
			Expected Count	38.9	33.1	72.0
			% within Age Groups	63.9%	36.1%	100.0%
		61-70	Count	74	61	135
			Expected Count	72.9	62.1	135.0
			% within Age Groups	54.8%	45.2%	100.0%
		71-80	Count	18	38	56
			Expected Count	30.2	25.8	56.0
			% within Age Groups	32.1%	67.9%	100.0%
		≥81	Count	2	23	25
			Expected Count	13.5	11.5	25.0
			% within Age Groups	8.0%	92.0%	100.0%
	Total		Count	197	168	365
			Expected Count	197.0	168.0	365.0
			% within Age Groups	54.0%	46.0%	100.0%
2021	Age Groups	≤30	Count	2	0	2
			Expected Count	1.2	.8	2.0

			% within Age Groups	100.0%	0.0%	100.0%
		31-40	Count	9	4	13
			Expected Count	8.0	5.0	13.0
			% within Age Groups	69.2%	30.8%	100.0%
		41-50	Count	47	12	59
			Expected Count	36.3	22.7	59.0
			% within Age Groups	79.7%	20.3%	100.0%
		51-60	Count	74	25	99
			Expected Count	60.9	38.1	99.0
			% within Age Groups	74.7%	25.3%	100.0%
		61-70	Count	64	35	99
			Expected Count	60.9	38.1	99.0
			% within Age Groups	64.6%	35.4%	100.0%
		71-80	Count	21	44	65
			Expected Count	40.0	25.0	65.0
			% within Age Groups	32.3%	67.7%	100.0%
		≥81	Count	2	17	19
			Expected Count	11.7	7.3	19.0
			% within Age Groups	10.5%	89.5%	100.0%
	Total		Count	219	137	356
			Expected Count	219.0	137.0	356.0
			% within Age Groups	61.5%	38.5%	100.0%
2022	Age Groups	≤30	Count	1	1	2
			Expected Count	1.3	.7	2.0
			% within Age Groups	50.0%	50.0%	100.0%
		31-40	Count	17	3	20
			Expected Count	13.0	7.0	20.0

			% within Age Groups	85.0%	15.0%	100.0%
		41-50	Count	56	8	64
			Expected Count	41.6	22.4	64.0
			% within Age Groups	87.5%	12.5%	100.0%
		51-60	Count	79	30	109
			Expected Count	70.9	38.1	109.0
			% within Age Groups	72.5%	27.5%	100.0%
		61-70	Count	87	28	115
			Expected Count	74.8	40.2	115.0
			% within Age Groups	75.7%	24.3%	100.0%
		71-80	Count	23	43	66
			Expected Count	42.9	23.1	66.0
			% within Age Groups	34.8%	65.2%	100.0%
		≥81	Count	3	30	33
			Expected Count	21.5	11.5	33.0
			% within Age Groups	9.1%	90.9%	100.0%
	Total		Count	266	143	409
			Expected Count	266.0	143.0	409.0
			% within Age Groups	65.0%	35.0%	100.0%
2023	Age Groups	≤30	Count	1	0	1
			Expected Count	.6	.4	1.0
			% within Age Groups	100.0%	0.0%	100.0%
		31-40	Count	9	5	14
			Expected Count	8.0	6.0	14.0
			% within Age Groups	64.3%	35.7%	100.0%
		41-50	Count	42	6	48
			Expected Count	27.3	20.7	48.0

			% within Age Groups	87.5%	12.5%	100.0%
		51-60	Count	62	22	84
			Expected Count	47.8	36.2	84.0
			% within Age Groups	73.8%	26.2%	100.0%
		61-70	Count	52	38	90
			Expected Count	51.2	38.8	90.0
			% within Age Groups	57.8%	42.2%	100.0%
		71-80	Count	25	52	77
			Expected Count	43.8	33.2	77.0
			% within Age Groups	32.5%	67.5%	100.0%
		≥81	Count	3	24	27
			Expected Count	15.4	11.6	27.0
			% within Age Groups	11.1%	88.9%	100.0%
	Total		Count	194	147	341
			Expected Count	194.0	147.0	341.0
			% within Age Groups	56.9%	43.1%	100.0%
Total	Age Groups	≤30	Count	9	1	10
			Expected Count	5.9	4.1	10.0
			% within Age Groups	90.0%	10.0%	100.0%
		31-40	Count	81	19	100
			Expected Count	58.9	41.1	100.0
			% within Age Groups	81.0%	19.0%	100.0%
		41-50	Count	286	72	358
			Expected Count	210.9	147.1	358.0
			% within Age Groups	79.9%	20.1%	100.0%
		51-60	Count	409	167	576
			Expected Count	339.4	236.6	576.0

		% within Age Groups	71.0%	29.0%	100.0%
	61-70	Count	444	288	732
		Expected Count	431.3	300.7	732.0
		% within Age Groups	60.7%	39.3%	100.0%
	71-80	Count	139	274	413
		Expected Count	243.4	169.6	413.0
		% within Age Groups	33.7%	66.3%	100.0%
	≥81	Count	22	148	170
		Expected Count	100.2	69.8	170.0
		% within Age Groups	12.9%	87.1%	100.0%
Total		Count	1390	969	2359
		Expected Count	1390.0	969.0	2359.0
		% within Age Groups	58.9%	41.1%	100.0%

Table S2. Pearson's chi-square tests—age group and year.

YEAR_min		Value	df	Asymptotic Significance (2-sided)
2019	Pearson Chi-Square	124.911[b]	6	.000
	Likelihood Ratio	132.148	6	.000
	Linear-by-Linear Association	118.859	1	.000
	N of Valid Cases	888		
2020	Pearson Chi-Square	48.245[c]	6	.000
	Likelihood Ratio	52.780	6	.000
	Linear-by-Linear Association	42.700	1	.000
	N of Valid Cases	365		
2021	Pearson Chi-Square	61.805[d]	6	.000
	Likelihood Ratio	63.727	6	.000
	Linear-by-Linear Association	47.557	1	.000
	N of Valid Cases	356		
2022	Pearson Chi-Square	98.134[e]	6	.000
	Likelihood Ratio	100.141	6	.000
	Linear-by-Linear Association	68.243	1	.000
	N of Valid Cases	409		
2023	Pearson Chi-Square	71.041[f]	6	.000
	Likelihood Ratio	76.712	6	.000
	Linear-by-Linear Association	59.624	1	.000
	N of Valid Cases	341		
Total	Pearson Chi-Square	382.238[a]	6	.000
	Likelihood Ratio	398.137	6	.000
	Linear-by-Linear Association	336.717	1	.000

N of Valid Cases	2359		

a. 1 cells (7.1%) have an expected count of less than 5. The minimum expected count is 4.11.

b. 2 cells (14.3%) have an expected count of less than 5. The minimum expected count is 1.26.

c. 2 cells (14.3%) have an expected count of less than 5. The minimum expected count is .92.

d. 2 cells (14.3%) have an expected count of less than 5. The minimum expected count is .77.

e. 2 cells (14.3%) have an expected count of less than 5. The minimum expected count is .70.

f. 2 cells (14.3%) have an expected count of less than 5. The minimum expected count is .43.

Table S3. Symmetric Measures—age group and year.

YEAR_min				Value	Asymptotic Standard Error[a]	Approximate T[b]	Approximate Significance
2019	Ordinal by Ordinal	Gamma	Zero-Order	.518	.040	12.082	.000
	N of Valid Cases			888			
2020	Ordinal by Ordinal	Gamma	Zero-Order	.484	.064	7.144	.000
	N of Valid Cases			365			
2021	Ordinal by Ordinal	Gamma	Zero-Order	.521	.065	7.371	.000
	N of Valid Cases			356			
2022	Ordinal by Ordinal	Gamma	Zero-Order	.571	.059	8.692	.000
	N of Valid Cases			409			
2023	Ordinal by Ordinal	Gamma	Zero-Order	.596	.059	9.225	.000
	N of Valid Cases			341			
Total	Ordinal by Ordinal	Gamma	Zero-Order	.535	.024	20.412	.000

		First-Order Partial	.529			
	N of Valid Cases		2359			

a. Not assuming the null hypothesis.

b. Using the asymptotic standard error assuming the null hypothesis.

Table S4. Age Groups * Alcoholic hepatitis (1=yes, 0=no) * YEAR_min Crosstabulation.

YEAR_min				Alcoholic hepatitis (1=yes, 0=no) no	yes	Total
2019	Age Groups	≤30	Count	1	2	3
			Expected Count	1.6	1.4	3.0
			% within Age Groups	33.3%	66.7%	100.0%
		31-40	Count	7	27	34
			Expected Count	18.3	15.7	34.0
			% within Age Groups	20.6%	79.4%	100.0%
		41-50	Count	41	90	131
			Expected Count	70.5	60.5	131.0
			% within Age Groups	31.3%	68.7%	100.0%
		51-60	Count	86	126	212
			Expected Count	114.1	97.9	212.0
			% within Age Groups	40.6%	59.4%	100.0%
		61-70	Count	165	128	293
			Expected Count	157.7	135.3	293.0
			% within Age Groups	56.3%	43.7%	100.0%
		71-80	Count	119	30	149

			Expected Count	80.2	68.8	149.0
			% within Age Groups	79.9%	20.1%	100.0%
		≥81	Count	59	7	66
			Expected Count	35.5	30.5	66.0
			% within Age Groups	89.4%	10.6%	100.0%
	Total		Count	478	410	888
			Expected Count	478.0	410.0	888.0
			% within Age Groups	53.8%	46.2%	100.0%
2020	Age Groups	≤30	Count	0	2	2
			Expected Count	1.3	.7	2.0
			% within Age Groups	0.0%	100.0%	100.0%
		31-40	Count	6	13	19
			Expected Count	12.8	6.2	19.0
			% within Age Groups	31.6%	68.4%	100.0%
		41-50	Count	27	29	56
			Expected Count	37.6	18.4	56.0
			% within Age Groups	48.2%	51.8%	100.0%
		51-60	Count	41	31	72
			Expected Count	48.3	23.7	72.0
			% within Age Groups	56.9%	43.1%	100.0%
		61-70	Count	102	33	135
			Expected Count	90.6	44.4	135.0
			% within Age Groups	75.6%	24.4%	100.0%
		71-80	Count	44	12	56
			Expected Count	37.6	18.4	56.0
			% within Age Groups	78.6%	21.4%	100.0%
		≥81	Count	25	0	25

			Expected Count	16.8	8.2	25.0
			% within Age Groups	100.0%	0.0%	100.0%
	Total		Count	245	120	365
			Expected Count	245.0	120.0	365.0
			% within Age Groups	67.1%	32.9%	100.0%
2021	Age Groups	≤30	Count	1	1	2
			Expected Count	1.3	.7	2.0
			% within Age Groups	50.0%	50.0%	100.0%
		31-40	Count	7	6	13
			Expected Count	8.3	4.7	13.0
			% within Age Groups	53.8%	46.2%	100.0%
		41-50	Count	27	32	59
			Expected Count	37.8	21.2	59.0
			% within Age Groups	45.8%	54.2%	100.0%
		51-60	Count	50	49	99
			Expected Count	63.4	35.6	99.0
			% within Age Groups	50.5%	49.5%	100.0%
		61-70	Count	69	30	99
			Expected Count	63.4	35.6	99.0
			% within Age Groups	69.7%	30.3%	100.0%
		71-80	Count	56	9	65
			Expected Count	41.6	23.4	65.0
			% within Age Groups	86.2%	13.8%	100.0%
		≥81	Count	18	1	19
			Expected Count	12.2	6.8	19.0
			% within Age Groups	94.7%	5.3%	100.0%
	Total		Count	228	128	356

			Expected Count	228.0	128.0	356.0
			% within Age Groups	64.0%	36.0%	100.0%
2022	Age Groups	≤30	Count	1	1	2
			Expected Count	1.1	.9	2.0
			% within Age Groups	50.0%	50.0%	100.0%
		31-40	Count	6	14	20
			Expected Count	11.3	8.7	20.0
			% within Age Groups	30.0%	70.0%	100.0%
		41-50	Count	20	44	64
			Expected Count	36.3	27.7	64.0
			% within Age Groups	31.3%	68.8%	100.0%
		51-60	Count	46	63	109
			Expected Count	61.8	47.2	109.0
			% within Age Groups	42.2%	57.8%	100.0%
		61-70	Count	74	41	115
			Expected Count	65.2	49.8	115.0
			% within Age Groups	64.3%	35.7%	100.0%
		71-80	Count	54	12	66
			Expected Count	37.4	28.6	66.0
			% within Age Groups	81.8%	18.2%	100.0%
		≥81	Count	31	2	33
			Expected Count	18.7	14.3	33.0
			% within Age Groups	93.9%	6.1%	100.0%
	Total		Count	232	177	409
			Expected Count	232.0	177.0	409.0
			% within Age Groups	56.7%	43.3%	100.0%
2023	Age Groups	≤30	Count	1	0	1

			Expected Count	.7	.3	1.0
			% within Age Groups	100.0%	0.0%	100.0%
		31-40	Count	5	9	14
			Expected Count	9.1	4.9	14.0
			% within Age Groups	35.7%	64.3%	100.0%
		41-50	Count	17	31	48
			Expected Count	31.2	16.8	48.0
			% within Age Groups	35.4%	64.6%	100.0%
		51-60	Count	46	38	84
			Expected Count	54.7	29.3	84.0
			% within Age Groups	54.8%	45.2%	100.0%
		61-70	Count	63	27	90
			Expected Count	58.6	31.4	90.0
			% within Age Groups	70.0%	30.0%	100.0%
		71-80	Count	65	12	77
			Expected Count	50.1	26.9	77.0
			% within Age Groups	84.4%	15.6%	100.0%
		≥81	Count	25	2	27
			Expected Count	17.6	9.4	27.0
			% within Age Groups	92.6%	7.4%	100.0%
	Total		Count	222	119	341
			Expected Count	222.0	119.0	341.0
			% within Age Groups	65.1%	34.9%	100.0%
Total	Age Groups	≤30	Count	4	6	10
			Expected Count	6.0	4.0	10.0
			% within Age Groups	40.0%	60.0%	100.0%
		31-40	Count	31	69	100

			Expected Count	59.6	40.4	100.0
			% within Age Groups	31.0%	69.0%	100.0%
		41-50	Count	132	226	358
			Expected Count	213.2	144.8	358.0
			% within Age Groups	36.9%	63.1%	100.0%
		51-60	Count	269	307	576
			Expected Count	343.1	232.9	576.0
			% within Age Groups	46.7%	53.3%	100.0%
		61-70	Count	473	259	732
			Expected Count	436.0	296.0	732.0
			% within Age Groups	64.6%	35.4%	100.0%
		71-80	Count	338	75	413
			Expected Count	246.0	167.0	413.0
			% within Age Groups	81.8%	18.2%	100.0%
		≥81	Count	158	12	170
			Expected Count	101.3	68.7	170.0
			% within Age Groups	92.9%	7.1%	100.0%
	Total		Count	1405	954	2359
			Expected Count	1405.0	954.0	2359.0
			% within Age Groups	59.6%	40.4%	100.0%

Table S5. Chi-Square Tests—age group and CHC.

YEAR_min		Value	df	Asymptotic Significance (2-sided)
2019	Pearson Chi-Square	132.345[b]	6	.000
	Likelihood Ratio	142.484	6	.000

	Linear-by-Linear Association	125.985	1	.000
	N of Valid Cases	888		
2020	Pearson Chi-Square	47.335[c]	6	.000
	Likelihood Ratio	54.278	6	.000
	Linear-by-Linear Association	44.716	1	.000
	N of Valid Cases	365		
2021	Pearson Chi-Square	40.147[d]	6	.000
	Likelihood Ratio	44.160	6	.000
	Linear-by-Linear Association	34.278	1	.000
	N of Valid Cases	356		
2022	Pearson Chi-Square	70.410[e]	6	.000
	Likelihood Ratio	76.930	6	.000
	Linear-by-Linear Association	65.221	1	.000
	N of Valid Cases	409		
2023	Pearson Chi-Square	51.003[f]	6	.000
	Likelihood Ratio	53.938	6	.000
	Linear-by-Linear Association	46.132	1	.000
	N of Valid Cases	341		
Total	Pearson Chi-Square	323.043[a]	6	.000
	Likelihood Ratio	349.514	6	.000
	Linear-by-Linear Association	310.466	1	.000
	N of Valid Cases	2359		

a. 1 cells (7.1%) have an expected count of less than 5. The minimum expected count is 4.04.

b. 2 cells (14.3%) have an expected count of less than 5. The minimum expected count is 1.39.

c. 2 cells (14.3%) have an expected count of less than 5. The minimum expected count is .66.

d. 3 cells (21.4%) have an expected count of less than 5. The minimum expected count is .72.

e. 2 cells (14.3%) have an expected count of less than 5. The minimum expected count is .87.

f. 3 cells (21.4%) have an expected count of less than 5. The minimum expected count is .35.

Table S6. Symmetric Measures—age group and CHC.

YEAR_min				Value	Asymptotic Standard Error[a]	Approximate T[b]	Approximate Significance
2019	Ordinal by Ordinal	Gamma	Zero-Order	-.537	.038	-13.011	.000
	N of Valid Cases			888			
2020	Ordinal by Ordinal	Gamma	Zero-Order	-.508	.064	-7.025	.000
	N of Valid Cases			365			
2021	Ordinal by Ordinal	Gamma	Zero-Order	-.473	.065	-6.765	.000
	N of Valid Cases			356			
2022	Ordinal by Ordinal	Gamma	Zero-Order	-.571	.054	-9.702	.000
	N of Valid Cases			409			
2023	Ordinal by Ordinal	Gamma	Zero-Order	-.545	.062	-7.798	.000
	N of Valid Cases			341			

Total	Ordinal by Ordinal	Gamma	Zero-Order	-.525	.024	-20.319	.000
			First-Order Partial	-.534			
	N of Valid Cases			2359			

a. Not assuming the null hypothesis.

b. Using the asymptotic standard error assuming the null hypothesis.

Table S7. Age Groups * Non-alcoholic cirrhosis * YEAR_min Crosstabulation.

				Non-alcoholic cirrhosis		
YEAR_min				no	yes	Total
2019	Age Groups	≤30	Count	3	0	3
			Expected Count	2.2	.8	3.0
			% within Age Groups	100.0%	0.0%	100.0%
		31-40	Count	25	9	34
			Expected Count	25.2	8.8	34.0
			% within Age Groups	73.5%	26.5%	100.0%
		41-50	Count	105	26	131
			Expected Count	97.2	33.8	131.0
			% within Age Groups	80.2%	19.8%	100.0%
		51-60	Count	149	63	212
			Expected Count	157.3	54.7	212.0
			% within Age Groups	70.3%	29.7%	100.0%
		61-70	Count	206	87	293
			Expected Count	217.4	75.6	293.0

			% within Age Groups	70.3%	29.7%	100.0%
		71-80	Count	110	39	149
			Expected Count	110.6	38.4	149.0
			% within Age Groups	73.8%	26.2%	100.0%
		≥81	Count	61	5	66
			Expected Count	49.0	17.0	66.0
			% within Age Groups	92.4%	7.6%	100.0%
	Total		Count	659	229	888
			Expected Count	659.0	229.0	888.0
			% within Age Groups	74.2%	25.8%	100.0%
2020	Age Groups	≤30	Count	2	0	2
			Expected Count	1.5	.5	2.0
			% within Age Groups	100.0%	0.0%	100.0%
		31-40	Count	15	4	19
			Expected Count	14.5	4.5	19.0
			% within Age Groups	78.9%	21.1%	100.0%
		41-50	Count	40	16	56
			Expected Count	42.8	13.2	56.0
			% within Age Groups	71.4%	28.6%	100.0%
		51-60	Count	52	20	72
			Expected Count	55.0	17.0	72.0
			% within Age Groups	72.2%	27.8%	100.0%
		61-70	Count	96	39	135
			Expected Count	103.2	31.8	135.0
			% within Age Groups	71.1%	28.9%	100.0%
		71-80	Count	50	6	56
			Expected Count	42.8	13.2	56.0

			% within Age Groups	89.3%	10.7%	100.0%
		≥81	Count	24	1	25
			Expected Count	19.1	5.9	25.0
			% within Age Groups	96.0%	4.0%	100.0%
	Total		Count	279	86	365
			Expected Count	279.0	86.0	365.0
			% within Age Groups	76.4%	23.6%	100.0%
2021	Age Groups	≤30	Count	1	1	2
			Expected Count	1.4	.6	2.0
			% within Age Groups	50.0%	50.0%	100.0%
		31-40	Count	8	5	13
			Expected Count	9.2	3.8	13.0
			% within Age Groups	61.5%	38.5%	100.0%
		41-50	Count	40	19	59
			Expected Count	41.6	17.4	59.0
			% within Age Groups	67.8%	32.2%	100.0%
		51-60	Count	73	26	99
			Expected Count	69.8	29.2	99.0
			% within Age Groups	73.7%	26.3%	100.0%
		61-70	Count	62	37	99
			Expected Count	69.8	29.2	99.0
			% within Age Groups	62.6%	37.4%	100.0%
		71-80	Count	49	16	65
			Expected Count	45.8	19.2	65.0
			% within Age Groups	75.4%	24.6%	100.0%
		≥81	Count	18	1	19
			Expected Count	13.4	5.6	19.0

			% within Age Groups	94.7%	5.3%	100.0%
	Total		Count	251	105	356
			Expected Count	251.0	105.0	356.0
			% within Age Groups	70.5%	29.5%	100.0%
2022	Age Groups	≤30	Count	2	0	2
			Expected Count	1.5	.5	2.0
			% within Age Groups	100.0%	0.0%	100.0%
		31-40	Count	18	2	20
			Expected Count	14.8	5.2	20.0
			% within Age Groups	90.0%	10.0%	100.0%
		41-50	Count	48	16	64
			Expected Count	47.4	16.6	64.0
			% within Age Groups	75.0%	25.0%	100.0%
		51-60	Count	85	24	109
			Expected Count	80.8	28.2	109.0
			% within Age Groups	78.0%	22.0%	100.0%
		61-70	Count	66	49	115
			Expected Count	85.2	29.8	115.0
			% within Age Groups	57.4%	42.6%	100.0%
		71-80	Count	53	13	66
			Expected Count	48.9	17.1	66.0
			% within Age Groups	80.3%	19.7%	100.0%
		≥81	Count	31	2	33
			Expected Count	24.4	8.6	33.0
			% within Age Groups	93.9%	6.1%	100.0%
	Total		Count	303	106	409
			Expected Count	303.0	106.0	409.0

			% within Age Groups	74.1%	25.9%	100.0%
2023	Age Groups	≤30	Count	0	1	1
			Expected Count	.8	.2	1.0
			% within Age Groups	0.0%	100.0%	100.0%
		31-40	Count	13	1	14
			Expected Count	10.8	3.2	14.0
			% within Age Groups	92.9%	7.1%	100.0%
		41-50	Count	37	11	48
			Expected Count	37.2	10.8	48.0
			% within Age Groups	77.1%	22.9%	100.0%
		51-60	Count	61	23	84
			Expected Count	65.0	19.0	84.0
			% within Age Groups	72.6%	27.4%	100.0%
		61-70	Count	66	24	90
			Expected Count	69.7	20.3	90.0
			% within Age Groups	73.3%	26.7%	100.0%
		71-80	Count	61	16	77
			Expected Count	59.6	17.4	77.0
			% within Age Groups	79.2%	20.8%	100.0%
		≥81	Count	26	1	27
			Expected Count	20.9	6.1	27.0
			% within Age Groups	96.3%	3.7%	100.0%
	Total		Count	264	77	341
			Expected Count	264.0	77.0	341.0
			% within Age Groups	77.4%	22.6%	100.0%
Total	Age Groups	≤30	Count	8	2	10
			Expected Count	7.4	2.6	10.0

		% within Age Groups	80.0%	20.0%	100.0%
	31-40	Count	79	21	100
		Expected Count	74.4	25.6	100.0
		% within Age Groups	79.0%	21.0%	100.0%
	41-50	Count	270	88	358
		Expected Count	266.5	91.5	358.0
		% within Age Groups	75.4%	24.6%	100.0%
	51-60	Count	420	156	576
		Expected Count	428.8	147.2	576.0
		% within Age Groups	72.9%	27.1%	100.0%
	61-70	Count	496	236	732
		Expected Count	544.9	187.1	732.0
		% within Age Groups	67.8%	32.2%	100.0%
	71-80	Count	323	90	413
		Expected Count	307.4	105.6	413.0
		% within Age Groups	78.2%	21.8%	100.0%
	≥81	Count	160	10	170
		Expected Count	126.5	43.5	170.0
		% within Age Groups	94.1%	5.9%	100.0%
Total		Count	1756	603	2359
		Expected Count	1756.0	603.0	2359.0
		% within Age Groups	74.4%	25.6%	100.0%

Table S8. Chi-Square Tests—NALC and age group.

YEAR_min		Value	df	Asymptotic Significance (2-sided)
2019	Pearson Chi-Square	18.961[b]	6	.004
	Likelihood Ratio	22.812	6	.001
	Linear-by-Linear Association	.715	1	.398
	N of Valid Cases	888		
2020	Pearson Chi-Square	14.745[c]	6	.022
	Likelihood Ratio	18.072	6	.006
	Linear-by-Linear Association	4.600	1	.032
	N of Valid Cases	365		
2021	Pearson Chi-Square	10.677[d]	6	.099
	Likelihood Ratio	12.344	6	.055
	Linear-by-Linear Association	2.664	1	.103
	N of Valid Cases	356		
2022	Pearson Chi-Square	29.024[e]	6	.000
	Likelihood Ratio	30.663	6	.000
	Linear-by-Linear Association	.000	1	.989
	N of Valid Cases	409		
2023	Pearson Chi-Square	12.953[f]	6	.044
	Likelihood Ratio	15.165	6	.019
	Linear-by-Linear Association	1.558	1	.212
	N of Valid Cases	341		
Total	Pearson Chi-Square	56.983[a]	6	.000
	Likelihood Ratio	67.373	6	.000
	Linear-by-Linear Association	6.395	1	.011
	N of Valid Cases	2359		

a. 1 cells (7.1%) have an expected count of less than 5. The minimum expected count is 2.56.

b. 2 cells (14.3%) have an expected count of less than 5. The minimum expected count is .77.

c. 3 cells (21.4%) have an expected count of less than 5. The minimum expected count is .47.

d. 3 cells (21.4%) have an expected count of less than 5. The minimum expected count is .59.

e. 2 cells (14.3%) have an expected count of less than 5. The minimum expected count is .52.

f. 3 cells (21.4%) have an expected count of less than 5. The minimum expected count is .23.

Table S9. Symmetric Measures—NALC and age group.

YEAR_min				Value	Asymptotic Standard Error[a]	Approximate T[b]	Approximate Significance
2019	Ordinal by Ordinal	Gamma	Zero-Order	-.040	.052	-.766	.444
	N of Valid Cases			888			
2020	Ordinal by Ordinal	Gamma	Zero-Order	-.213	.079	-2.594	.009
	N of Valid Cases			365			
2021	Ordinal by Ordinal	Gamma	Zero-Order	-.111	.081	-1.366	.172
	N of Valid Cases			356			
2022	Ordinal by Ordinal	Gamma	Zero-Order	.016	.073	.215	.830
	N of Valid Cases			409			
2023	Ordinal by Ordinal	Gamma	Zero-Order	-.117	.085	-1.366	.172
	N of Valid Cases			341			

Total	Ordinal by Ordinal	Gamma	Zero-Order	-.080	.032	-2.498	.012
			First-Order Partial	-.063			
	N of Valid Cases			2359			

a. Not assuming the null hypothesis.

b. Using the asymptotic standard error assuming the null hypothesis.

Table S10. Age Groups * Alcoholic cirrhosis (1=yes, 0=no) * YEAR_min Crosstabulation.

YEAR_min				Alcoholic cirrhosis (1=yes, 0=no) no	yes	Total
2019	Age Groups	≤30	Count	2	1	3
			Expected Count	2.6	.4	3.0
			% within Age Groups	66.7%	33.3%	100.0%
		31-40	Count	30	4	34
			Expected Count	29.7	4.3	34.0
			% within Age Groups	88.2%	11.8%	100.0%
		41-50	Count	104	27	131
			Expected Count	114.6	16.4	131.0
			% within Age Groups	79.4%	20.6%	100.0%
		51-60	Count	176	36	212
			Expected Count	185.5	26.5	212.0
			% within Age Groups	83.0%	17.0%	100.0%
		61-70	Count	259	34	293
			Expected Count	256.4	36.6	293.0
			% within Age Groups	88.4%	11.6%	100.0%
		71-80	Count	141	8	149
			Expected Count	130.4	18.6	149.0

			% within Age Groups	94.6%	5.4%	100.0%
		≥81	Count	65	1	66
			Expected Count	57.8	8.3	66.0
			% within Age Groups	98.5%	1.5%	100.0%
	Total		Count	777	111	888
			Expected Count	777.0	111.0	888.0
			% within Age Groups	87.5%	12.5%	100.0%
2020	Age Groups	≤30	Count	2	0	2
			Expected Count	1.8	.2	2.0
			% within Age Groups	100.0%	0.0%	100.0%
		31-40	Count	16	3	19
			Expected Count	17.1	1.9	19.0
			% within Age Groups	84.2%	15.8%	100.0%
		41-50	Count	50	6	56
			Expected Count	50.3	5.7	56.0
			% within Age Groups	89.3%	10.7%	100.0%
		51-60	Count	62	10	72
			Expected Count	64.7	7.3	72.0
			% within Age Groups	86.1%	13.9%	100.0%
		61-70	Count	120	15	135
			Expected Count	121.3	13.7	135.0
			% within Age Groups	88.9%	11.1%	100.0%
		71-80	Count	54	2	56
			Expected Count	50.3	5.7	56.0
			% within Age Groups	96.4%	3.6%	100.0%
		≥81	Count	24	1	25
			Expected Count	22.5	2.5	25.0

			% within Age Groups	96.0%	4.0%	100.0%
	Total		Count	328	37	365
			Expected Count	328.0	37.0	365.0
			% within Age Groups	89.9%	10.1%	100.0%
2021	Age Groups	≤30	Count	2	0	2
			Expected Count	1.8	.2	2.0
			% within Age Groups	100.0%	0.0%	100.0%
		31-40	Count	12	1	13
			Expected Count	11.6	1.4	13.0
			% within Age Groups	92.3%	7.7%	100.0%
		41-50	Count	53	6	59
			Expected Count	52.7	6.3	59.0
			% within Age Groups	89.8%	10.2%	100.0%
		51-60	Count	82	17	99
			Expected Count	88.4	10.6	99.0
			% within Age Groups	82.8%	17.2%	100.0%
		61-70	Count	87	12	99
			Expected Count	88.4	10.6	99.0
			% within Age Groups	87.9%	12.1%	100.0%
		71-80	Count	63	2	65
			Expected Count	58.1	6.9	65.0
			% within Age Groups	96.9%	3.1%	100.0%
		≥81	Count	19	0	19
			Expected Count	17.0	2.0	19.0
			% within Age Groups	100.0%	0.0%	100.0%
	Total		Count	318	38	356
			Expected Count	318.0	38.0	356.0

			% within Age Groups	89.3%	10.7%	100.0%
2022	Age Groups	≤30	Count	2	0	2
			Expected Count	1.8	.2	2.0
			% within Age Groups	100.0%	0.0%	100.0%
		31-40	Count	17	3	20
			Expected Count	18.2	1.8	20.0
			% within Age Groups	85.0%	15.0%	100.0%
		41-50	Count	54	10	64
			Expected Count	58.4	5.6	64.0
			% within Age Groups	84.4%	15.6%	100.0%
		51-60	Count	98	11	109
			Expected Count	99.4	9.6	109.0
			% within Age Groups	89.9%	10.1%	100.0%
		61-70	Count	103	12	115
			Expected Count	104.9	10.1	115.0
			% within Age Groups	89.6%	10.4%	100.0%
		71-80	Count	66	0	66
			Expected Count	60.2	5.8	66.0
			% within Age Groups	100.0%	0.0%	100.0%
		≥81	Count	33	0	33
			Expected Count	30.1	2.9	33.0
			% within Age Groups	100.0%	0.0%	100.0%
	Total		Count	373	36	409
			Expected Count	373.0	36.0	409.0
			% within Age Groups	91.2%	8.8%	100.0%
2023	Age Groups	≤30	Count	1	0	1
			Expected Count	.9	.1	1.0

			% within Age Groups	100.0%	0.0%	100.0%
		31-40	Count	12	2	14
			Expected Count	12.4	1.6	14.0
			% within Age Groups	85.7%	14.3%	100.0%
		41-50	Count	37	11	48
			Expected Count	42.7	5.3	48.0
			% within Age Groups	77.1%	22.9%	100.0%
		51-60	Count	73	11	84
			Expected Count	74.6	9.4	84.0
			% within Age Groups	86.9%	13.1%	100.0%
		61-70	Count	79	11	90
			Expected Count	80.0	10.0	90.0
			% within Age Groups	87.8%	12.2%	100.0%
		71-80	Count	74	3	77
			Expected Count	68.4	8.6	77.0
			% within Age Groups	96.1%	3.9%	100.0%
		≥81	Count	27	0	27
			Expected Count	24.0	3.0	27.0
			% within Age Groups	100.0%	0.0%	100.0%
	Total		Count	303	38	341
			Expected Count	303.0	38.0	341.0
			% within Age Groups	88.9%	11.1%	100.0%
Total	Age Groups	≤30	Count	9	1	10
			Expected Count	8.9	1.1	10.0
			% within Age Groups	90.0%	10.0%	100.0%
		31-40	Count	87	13	100
			Expected Count	89.0	11.0	100.0

			% within Age Groups	87.0%	13.0%	100.0%
		41-50	Count	298	60	358
			Expected Count	318.5	39.5	358.0
			% within Age Groups	83.2%	16.8%	100.0%
		51-60	Count	491	85	576
			Expected Count	512.5	63.5	576.0
			% within Age Groups	85.2%	14.8%	100.0%
		61-70	Count	648	84	732
			Expected Count	651.3	80.7	732.0
			% within Age Groups	88.5%	11.5%	100.0%
		71-80	Count	398	15	413
			Expected Count	367.5	45.5	413.0
			% within Age Groups	96.4%	3.6%	100.0%
		≥81	Count	168	2	170
			Expected Count	151.3	18.7	170.0
			% within Age Groups	98.8%	1.2%	100.0%
	Total		Count	2099	260	2359
			Expected Count	2099.0	260.0	2359.0
			% within Age Groups	89.0%	11.0%	100.0%

Table S11. Chi-Square Tests—ALC and age group.

YEAR_min		Value	df	Asymptotic Significance (2-sided)
2019	Pearson Chi-Square	27.402[b]	6	.000
	Likelihood Ratio	31.162	6	.000
	Linear-by-Linear Association	21.710	1	.000

	N of Valid Cases	888		
2020	Pearson Chi-Square	5.849[c]	6	.440
	Likelihood Ratio	6.928	6	.328
	Linear-by-Linear Association	2.858	1	.091
	N of Valid Cases	365		
2021	Pearson Chi-Square	11.182[d]	6	.083
	Likelihood Ratio	14.188	6	.028
	Linear-by-Linear Association	3.110	1	.078
	N of Valid Cases	356		
2022	Pearson Chi-Square	15.025[e]	6	.020
	Likelihood Ratio	23.076	6	.001
	Linear-by-Linear Association	10.866	1	.001
	N of Valid Cases	409		
2023	Pearson Chi-Square	14.884[f]	6	.021
	Likelihood Ratio	17.800	6	.007
	Linear-by-Linear Association	11.280	1	.001
	N of Valid Cases	341		
Total	Pearson Chi-Square	60.577[a]	6	.000
	Likelihood Ratio	75.122	6	.000
	Linear-by-Linear Association	46.412	1	.000
	N of Valid Cases	2359		

a. 1 cells (7.1%) have an expected count of less than 5. The minimum expected count is 1.10.

b. 3 cells (21.4%) have an expected count of less than 5. The minimum expected count is .38.

c. 4 cells (28.6%) have an expected count of less than 5. The minimum expected count is .20.

d. 4 cells (28.6%) have an expected count of less than 5. The minimum expected count is .21.

e. 4 cells (28.6%) have an expected count of less than 5. The minimum expected count is .18.

f. 4 cells (28.6%) have an expected count of less than 5. The minimum expected count is .11.

Table S12. Symmetric Measures—ALC and age group.

YEAR_min				Value	Asymptotic Standard Error[a]	Approximate T[b]	Approximate Significance
2019	Ordinal by Ordinal	Gamma	Zero-Order	-.353	.061	-5.140	.000
	N of Valid Cases			888			
2020	Ordinal by Ordinal	Gamma	Zero-Order	-.232	.110	-1.980	.048
	N of Valid Cases			365			
2021	Ordinal by Ordinal	Gamma	Zero-Order	-.242	.098	-2.315	.021
	N of Valid Cases			356			
2022	Ordinal by Ordinal	Gamma	Zero-Order	-.420	.091	-3.677	.000
	N of Valid Cases			409			
2023	Ordinal by Ordinal	Gamma	Zero-Order	-.422	.095	-3.687	.000
	N of Valid Cases			341			
Total	Ordinal by Ordinal	Gamma	Zero-Order	-.337	.038	-7.760	.000
			First-Order Partial	-.346			
	N of Valid Cases			2359			

a. Not assuming the null hypothesis.

b. Using the asymptotic standard error assuming the null hypothesis.

Table S13. Distribution of annual hospitalization days (2019-2023) showed extreme positive skew and high kurtosis.

Descriptives

	YEAR_min			Statistic	Std. Error
Days_Hospitalization_per_years_sum	2019	Mean		13.7973	.52724
		95% Confidence Interval for Mean	Lower Bound	12.7625	
			Upper Bound	14.8321	
		5% Trimmed Mean		11.6604	
		Median		9.0000	
		Variance		246.852	
		Std. Deviation		15.71152	
		Minimum		1.00	
		Maximum		147.00	
		Range		146.00	
		Interquartile Range		11.00	
		Skewness		3.582	.082
		Kurtosis		19.400	.164
	2020	Mean		10.5549	.60355
		95% Confidence Interval for Mean	Lower Bound	9.3681	
			Upper Bound	11.7418	
		5% Trimmed Mean		9.2564	
		Median		7.0000	
		Variance		132.595	
		Std. Deviation		11.51498	
		Minimum		1.00	
		Maximum		137.00	
		Range		136.00	
		Interquartile Range		9.00	
		Skewness		5.278	.128

		Kurtosis		47.058	.255
	2021	Mean		10.4719	.48584
		95% Confidence Interval for Mean	Lower Bound	9.5164	
			Upper Bound	11.4274	
		5% Trimmed Mean		9.3789	
		Median		8.0000	
		Variance		84.030	
		Std. Deviation		9.16680	
		Minimum		1.00	
		Maximum		62.00	
		Range		61.00	
		Interquartile Range		8.00	
		Skewness		2.327	.129
		Kurtosis		7.280	.258
	2022	Mean		10.9731	.59585
		95% Confidence Interval for Mean	Lower Bound	9.8018	
			Upper Bound	12.1444	
		5% Trimmed Mean		9.4976	
		Median		8.0000	
		Variance		145.213	
		Std. Deviation		12.05042	
		Minimum		1.00	
		Maximum		164.00	
		Range		163.00	
		Interquartile Range		8.00	
		Skewness		6.184	.121
		Kurtosis		65.999	.241

	2023	Mean		9.0997	.43037
		95% Confidence Interval for Mean	Lower Bound	8.2532	
			Upper Bound	9.9462	
		5% Trimmed Mean		8.1812	
		Median		7.0000	
		Variance		63.161	
		Std. Deviation		7.94737	
		Minimum		1.00	
		Maximum		62.00	
		Range		61.00	
		Interquartile Range		6.00	
		Skewness		2.726	.132
		Kurtosis		11.368	.263

Tests of Normality

		Kolmogorov–Smirnov[a]			Shapiro-Wilk		
	YEAR_min	Statistic	df	Sig.	Statistic	df	Sig.
Days_Hospitalization_per_years_sum	2019	.208	888	.000	.665	888	.000
	2020	.203	364	.000	.617	364	.000
	2021	.192	356	.000	.779	356	.000
	2022	.205	409	.000	.583	409	.000
	2023	.200	341	.000	.761	341	.000

a. Lilliefors Significance Correction.

Table S14. Days of hospitalization per CHC per year .

Descriptives

Chronic hepatitis C (1=yes, 0=no)			Bootstrap[a]

				Statistic	Std. Error	Bias	Std. Error	95% Confidence Interval Lower	95% Confidence Interval Upper
Days_Hospitalization_per_yers_sum	no	Mean		12.0152	.29777	.0019	.2957	11.4601	12.5605
		95% Confidence Interval for Mean	Lower Bound	11.4311					
			Upper Bound	12.5993					
		5% Trimmed Mean		10.5400		.0009	.2579	10.0308	11.0313
		Median		8.0000		.3670	.4812	8.0000	9.0000
		Variance		123.248		.487	9.134	106.037	141.080
		Std. Deviation		11.10173		.01430	.41123	10.29742	11.87769
		Minimum		1.00					
		Maximum		60.82					
		Range		59.82					

		Interquartile Range		10.00		-.35	.52	9.00	10.00
		Skewness		2.303	.066	-.003	.084	2.138	2.472
		Kurtosis		6.073	.131	-.015	.557	5.029	7.245
	yes	Mean		10.3793	.32573	-.0162	.3203	9.7058	11.0227
		95% Confidence Interval for Mean	Lower Bound	9.7401					
			Upper Bound	11.0185					
		5% Trimmed Mean		9.0885		-.0038	.2778	8.5121	9.6376
		Median		7.0000		.1905	.3887	7.0000	8.0000
		Variance		102.813		-.675	9.466	85.053	121.121
		Std. Deviation		10.13967		-.04415	.46797	9.22240	11.00552
		Minimum		1.00					

	Maximum	60.82					
	Range	59.82					
	Interquartile Range	9.00		.04	.66	8.00	10.00
	Skewness	2.270	.079	-.011	.123	2.005	2.502
	Kurtosis	6.489	.157	-.034	.798	4.969	8.117

a. Unless otherwise noted, bootstrap results are based on 1000 bootstrap samples.

Percentiles

					Bootstrap[a]			
		Chronic hepatitis C (1=yes, 0=no)					95% Confidence Interval	
			Percentiles	Percentile	Bias	Std. Error	Lower	Upper
Weighted Average(Definition 1)	Days_Hospitalization_per_yers_sum	no	5	2.0000	.0000	.0000	2.0000	2.0000
			10	3.0000	.0150	.1165	3.0000	3.0000
			25	5.0000	.0517	.2181	5.0000	6.0000
			50	8.0000	.3670	.4812	8.0000	9.0000
			75	15.0000	-.2940	.4934	14.0000	15.0000
			90	25.0000	.0376	.9948	23.0000	27.0000
			95	35.0000	.4122	2.1510	31.0000	40.0000
		yes	5	1.0000	.0000	.0000	1.0000	1.0000
			10	2.0000	-.1672	.3657	1.0000	2.0000
			25	4.0000	-.0395	.2009	3.0000	4.0000
			50	7.0000	.1905	.3887	7.0000	8.0000
			75	13.0000	.0018	.6394	12.0000	14.0000
			90	23.0000	-.4196	.9502	21.0000	25.0000
			95	30.0000	.0920	1.6752	27.0000	34.0000

Tukey's Hinges	Days_Hospitalization_per_yers_sum	no	25	5.0000	.0545	.2266	5.0000	6.0000
			50	8.0000	.3670	.4812	8.0000	9.0000
			75	15.0000	-.3050	.4967	14.0000	15.0000
		yes	25	4.0000	-.0365	.1999	3.0000	4.0000
			50	7.0000	.1905	.3887	7.0000	8.0000
			75	13.0000	-.0120	.6370	12.0000	14.0000

a. Unless otherwise noted, bootstrap results are based on 1000 bootstrap samples.

Descriptives

					Bootstrap[a]			
							95% Confidence Interval	
	YEAR_min		Statistic	Std. Error	Bias	Std. Error	Lower	Upper
Days_Hospitalization_per_yers_sum	Mean		13.2466	.43005	.0050	.4188	12.4587	14.0858
	95% Confidence Interval for Mean	Lower Bound	12.4025					
		Upper Bound	14.0906					
	5% Trimmed Mean		11.6604		.0067	.4045	10.9096	12.4987
	Median		9.0000		.0005	.2394	8.0000	10.0000
	Variance		164.233		.387	12.625	139.322	189.296
	Std. Deviation		12.81535		.00561	.49300	11.80347	13.75849
	Minimum		1.00					
	Maximum		60.82					
	Range		59.82					
	Interquartile Range		11.00		.30	.70	10.00	13.00
	Skewness		1.946	.082	-.003	.089	1.772	2.125
	Kurtosis		3.774	.164	-.006	.498	2.799	4.816
	Mean		10.3930	.49649	-.0087	.4984	9.4322	11.4384
	95% Confidence Interval for Mean	Lower Bound	9.4167					
		Upper Bound	11.3694					

	5% Trimmed Mean		9.3135		.0062	.4479	8.4325	10.2232
	Median		7.0000		.1630	.3900	7.0000	8.0000
	Variance		89.974		-.593	14.074	64.523	119.749
	Std. Deviation		9.48548		-.06052	.74286	8.03260	10.94298
	Minimum		1.00					
	Maximum		60.82					
	Range		59.82					
	Interquartile Range		9.00		-.19	1.03	7.00	11.00
	Skewness		2.277	.128	-.053	.286	1.615	2.738
	Kurtosis		7.285	.255	-.334	1.779	3.023	10.306
	Mean		10.4686	.48486	-.0226	.4906	9.5114	11.4377
	95% Confidence Interval for Mean	Lower Bound	9.5150					
		Upper Bound	11.4222					
	5% Trimmed Mean		9.3789		-.0104	.4299	8.5540	10.2170
	Median		8.0000		-.1895	.4081	7.0000	8.0000
	Variance		83.692		-.791	13.287	58.695	110.945
	Std. Deviation		9.14831		-.07278	.73229	7.66125	10.53304
	Minimum		1.00					
	Maximum		60.82					
	Range		59.82					
	Interquartile Range		8.00		.46	.96	7.00	10.00
	Skewness		2.308	.129	-.041	.258	1.722	2.750
	Kurtosis		7.112	.258	-.239	1.715	3.446	10.554
	Mean		10.6935	.47115	-.0111	.4634	9.7799	11.6107
	95% Confidence Interval for Mean	Lower Bound	9.7673					
		Upper Bound	11.6197					
	5% Trimmed Mean		9.4976		-.0024	.3961	8.7229	10.3102

	Median		8.0000		.0585	.3065	8.0000	9.0000
	Variance		90.791		.167	13.712	63.702	120.061
	Std. Deviation		9.52842		-.01865	.72269	7.98136	10.95722
	Minimum		1.00					
	Maximum		60.82					
	Range		59.82					
	Interquartile Range		8.00		.33	.76	7.00	10.00
	Skewness		2.453	.121	-.034	.218	1.940	2.818
	Kurtosis		7.901	.241	-.208	1.448	4.934	10.637
	Mean		9.0962	.42914	.0014	.4450	8.2284	9.9704
	95% Confidence Interval for Mean	Lower Bound	8.2522					
		Upper Bound	9.9403					
	5% Trimmed Mean		8.1812		.0133	.3727	7.4839	8.9411
	Median		7.0000		.1175	.3662	7.0000	8.0000
	Variance		62.798		-.275	12.238	40.644	88.626
	Std. Deviation		7.92449		-.05538	.77469	6.37529	9.41412
	Minimum		1.00					
	Maximum		60.82					
	Range		59.82					
	Interquartile Range		6.00		.36	.83	5.00	8.00
	Skewness		2.693	.132	-.112	.390	1.679	3.249
	Kurtosis		11.026	.263	-.847	2.882	3.742	15.993

a. Unless otherwise noted, bootstrap results are based on 1000 bootstrap samples.

Percentiles

			Bootstrap[a]			
					95% Confidence Interval	
YEAR_min	Percentiles	Percentile	Bias	Std. Error	Lower	Upper

Weighted Average(Definition 1)	Days_Hospitalization_per_yers_sum	2019	5	2.0000	-.3233	.4584	1.0000	2.0000
			10	2.0000	.2172	.4075	2.0000	3.0000
			25	5.0000	-.0597	.2947	4.0000	5.0000
			50	9.0000	.0005	.2394	8.0000	10.0000
			75	16.0000	.2450	.6936	15.0000	18.0000
			90	29.0000	.3550	1.8788	27.0000	34.0000
			95	43.0000	.0782	1.8768	39.0000	48.0000
		2020	5	1.0000	.0962	.2790	1.0000	2.0000
			10	2.0000	.1141	.3294	2.0000	3.0000
			25	5.0000	-.2970	.4517	4.0000	5.0000
			50	7.0000	.1630	.3900	7.0000	8.0000
			75	14.0000	-.4835	1.0460	11.0000	16.0000
			90	23.0000	-.3696	1.0555	21.0000	25.0975
			95	29.0000	-.6047	1.9560	25.0000	32.4975
		2021	5	2.0000	-.2457	.4214	1.0000	2.0000
			10	3.0000	-.2523	.4365	2.0000	3.0000
			25	5.0000	-.2202	.4304	4.0000	5.0000
			50	8.0000	-.1895	.4081	7.0000	8.0000
			75	13.0000	.2413	.9866	12.0000	15.0000
			90	21.3000	.1621	1.7792	18.0000	27.0000
			95	28.1500	.4234	1.9555	23.8025	33.4975
		2022	5	1.0000	.4027	.4756	1.0000	2.0000
			10	2.0000	.3179	.4650	2.0000	3.0000
			25	5.0000	.0165	.2406	4.7564	6.0000
			50	8.0000	.0585	.3065	8.0000	9.0000
			75	13.0000	.3450	.7744	12.0000	15.0000
			90	21.0000	.4310	1.4164	20.0000	25.0000

			95	30.0000	-.4738	3.0830	25.0000	34.0000
		2023	5	1.0000	.0285	.1584	1.0000	1.6000
			10	2.0000	.0722	.3752	1.0000	3.0000
			25	5.0000	-.3970	.4877	4.0000	5.0000
			50	7.0000	.1175	.3662	7.0000	8.0000
			75	11.0000	-.0332	.8260	10.0000	13.0000
			90	18.8000	-.2357	1.2827	16.0000	21.2975
			95	23.0000	.4618	1.6306	20.9513	27.0000
Tukey's Hinges	Days_Hospitalization_per_yers_sum	2019	25	5.0000	-.0570	.2972	4.0000	5.0000
			50	9.0000	.0005	.2394	8.0000	10.0000
			75	16.0000	.2250	.6855	15.0000	18.0000
		2020	25	5.0000	-.2850	.4461	4.0000	5.0000
			50	7.0000	.1630	.3900	7.0000	8.0000
			75	14.0000	-.5245	1.0506	11.0000	16.0000
		2021	25	5.0000	-.2035	.4225	4.0000	5.0000
			50	8.0000	-.1895	.4081	7.0000	8.0000
			75	13.0000	.2070	.9888	12.0000	15.0000
		2022	25	5.0000	.0220	.2441	5.0000	6.0000
			50	8.0000	.0585	.3065	8.0000	9.0000
			75	13.0000	.3070	.7864	12.0000	15.0000
		2023	25	5.0000	-.3810	.4817	4.0000	5.0000
			50	7.0000	.1175	.3662	7.0000	8.0000
			75	11.0000	-.0680	.8189	10.0000	13.0000

a. Unless otherwise noted, bootstrap results are based on 1000 bootstrap samples.

Table S15. Days of hospitalization per ALH per year.

Descriptives

	Alcoholic hepatitis (1=yes, 0=no)			Statistic	Std. Error	Bootstrap[a] Bias	Std. Error	95% Confidence Interval Lower	Upper
Days_Hospitalization_per_yers_sum	r c	Mean		10.1909	.25659	-.0057	.2542	9.6802	10.6746
		95% Confidence Interval for Mean	Lower Bound	9.6875					
			Upper Bound	10.6942					
		5% Trimmed Mean		8.9725		.0031	.2149	8.5360	9.3930
		Median		7.0000		.2160	.4093	7.0000	8.0000
		Variance		92.505		-.537	7.779	77.386	107.718
		Std. Deviation		9.61797		-.03657	.40615	8.79692	10.37873
		Minimum		1.00					
		Maximum		60.82					
		Range		59.82					
		Interquartile Range		9.00		-.47	.57	7.51	9.00
		Skewness		2.474	.065	-.015	.116	2.219	2.686
		Kurtosis		8.029	.130	-.073	.776	6.457	9.521
	y e s	Mean		13.0403	.38925	.0124	.3809	12.2741	13.7915
		95% Confidence Interval for Mean	Lower Bound	12.2764					
			Upper Bound	13.8042					
		5% Trimmed Mean		11.5291		.0179	.3597	10.8290	12.2473
		Median		9.0000		.0010	.1747	9.0000	9.0000
		Variance		144.546		.301	11.574	122.310	167.372

	Std. Deviation	12.02271		.00290	.48151	11.05939	12.93724
	Minimum	1.00					
	Maximum	60.82					
	Range	59.82					
	Interquartile Range	11.00		-.43	.65	9.75	12.00
	Skewness	2.040	.079	-.004	.092	1.854	2.220
	Kurtosis	4.429	.158	-.013	.535	3.421	5.544

a. Unless otherwise noted, bootstrap results are based on 1000 bootstrap samples.

Percentiles

		Alcoholic hepatitis (1=yes, 0=no)	Percentiles	Percentile	Bootstrap[a]			
							95% Confidence Interval	
					Bias	Std. Error	Lower	Upper
Weighted Average(Definition 1)	Days_Hospitalization_per_yers_sum	no	5	1.0000	.0030	.0547	1.0000	1.0000
			10	2.0000	.0000	.0000	2.0000	2.0000
			25	4.0000	.1567	.3593	4.0000	5.0000
			50	7.0000	.2160	.4093	7.0000	8.0000
			75	13.0000	-.3130	.5235	12.0000	13.9936
			90	21.0000	.4451	.8502	20.0000	23.0000
			95	28.0000	.3560	1.0405	26.0000	30.4462
		yes	5	2.0000	-.0030	.0668	2.0000	2.0000
			10	3.0000	.0619	.2950	3.0000	4.0000
			25	5.0000	.3858	.4831	5.0000	6.0000
			50	9.0000	.0010	.1747	9.0000	9.0000
			75	16.0000	-.0392	.5747	15.0000	17.0000
			90	28.0000	-.2458	1.3366	25.0000	31.0000

			95	41.0000	-.3884	2.4539	35.0000	44.0000
Tukey's Hinges	Days_Hospitalization_per_yers_sum	no	25	4.0000	.1650	.3680	4.0000	5.0000
			50	7.0000	.2160	.4093	7.0000	8.0000
			75	13.0000	-.3220	.5259	12.0000	13.5000
		yes	25	5.0000	.3960	.4852	5.0000	6.0000
			50	9.0000	.0010	.1747	9.0000	9.0000
			75	16.0000	-.0565	.5687	15.0000	17.0000

a. Unless otherwise noted, bootstrap results are based on 1000 bootstrap samples.

Descriptives

						Bootstrap[a]			
								95% Confidence Interval	
	YEAR_min			Statistic	Std. Error	Bias	Std. Error	Lower	Upper
Days_Hospitalization_per_yers_sum	2019	Mean		13.2466	.43005	-.0050	.4345	12.3632	14.1445
		95% Confidence Interval for Mean	Lower Bound	12.4025					
			Upper Bound	14.0906					
		5% Trimmed Mean		11.6604		.0003	.4169	10.8100	12.5417
		Median		9.0000		-.0020	.2618	8.0000	10.0000
		Variance		164.233		-.284	12.995	139.078	190.529
		Std. Deviation		12.81535		-.02116	.50841	11.79315	13.80322
		Minimum		1.00					
		Maximum		60.82					
		Range		59.82					
		Interquartile Range		11.00		.29	.72	10.00	13.00
		Skewness		1.946	.082	-.001	.091	1.768	2.129
		Kurtosis		3.774	.164	.011	.514	2.843	4.874
	2020	Mean		10.3930	.49649	-.0006	.4949	9.4827	11.3992
			Lower Bound	9.4167					

		95% Confidence Interval for Mean	Upper Bound	11.3694					
		5% Trimmed Mean		9.3135		.0152	.4363	8.5379	10.1949
		Median		7.0000		.1560	.3890	7.0000	8.0000
		Variance		89.974		-.465	14.369	64.168	120.097
		Std. Deviation		9.48548		-.05485	.75682	8.01050	10.95888
		Minimum		1.00					
		Maximum		60.82					
		Range		59.82					
		Interquartile Range		9.00		-.14	1.04	7.00	11.00
		Skewness		2.277	.128	-.056	.268	1.657	2.706
		Kurtosis		7.285	.255	-.351	1.670	3.277	10.138
	2021	Mean		10.4686	.48486	.0008	.4915	9.5547	11.4691
		95% Confidence Interval for Mean	Lower Bound	9.5150					
			Upper Bound	11.4222					
		5% Trimmed Mean		9.3789		.0113	.4243	8.5513	10.2067
		Median		8.0000		-.1755	.3809	7.0000	8.0000
		Variance		83.692		-.291	13.785	58.574	114.557
		Std. Deviation		9.14831		-.04702	.75377	7.65339	10.70312
		Minimum		1.00					
		Maximum		60.82					
		Range		59.82					
		Interquartile Range		8.00		.54	1.01	7.00	10.25
		Skewness		2.308	.129	-.057	.254	1.735	2.753
		Kurtosis		7.112	.258	-.353	1.678	3.634	10.216
	2022	Mean		10.6935	.47115	.0072	.4709	9.7861	11.6029
			Lower Bound	9.7673					

		95% Confidence Interval for Mean	Upper Bound	11.6197				
		5% Trimmed Mean		9.4976		.0292	.4052	8.7822	10.3112
		Median		8.0000		.0650	.2964	8.0000	9.0000
		Variance		90.791		-.435	13.887	64.536	117.705
		Std. Deviation		9.52842		-.05125	.73471	8.03342	10.84920
		Minimum		1.00					
		Maximum		60.82					
		Range		59.82					
		Interquartile Range		8.00		.31	.79	7.00	10.00
		Skewness		2.453	.121	-.044	.221	1.966	2.844
		Kurtosis		7.901	.241	-.226	1.467	4.853	10.809
	2023	Mean		9.0962	.42914	.0065	.4384	8.2413	10.0615
		95% Confidence Interval for Mean	Lower Bound	8.2522					
			Upper Bound	9.9403					
		5% Trimmed Mean		8.1812		.0106	.3708	7.4950	8.9907
		Median		7.0000		.1150	.3455	7.0000	8.0000
		Variance		62.798		-.034	12.192	41.666	88.407
		Std. Deviation		7.92449		-.03940	.76783	6.45495	9.40249
		Minimum		1.00					
		Maximum		60.82					
		Range		59.82					
		Interquartile Range		6.00		.38	.85	5.00	8.00
		Skewness		2.693	.132	-.103	.387	1.641	3.244
		Kurtosis		11.026	.263	-.841	2.876	3.870	15.647

a. Unless otherwise noted, bootstrap results are based on 1000 bootstrap samples.

Percentiles

		YEAR_min	Percentiles	Percentile	Bootstrap[a] Bias	Std. Error	95% Confidence Interval Lower	Upper
Weighted Average(Definition 1)	Days_Hospitalization_per_yers_sum	2019	5	2.0000	-.2956	.4461	1.0000	2.0000
			10	2.0000	.2781	.4405	2.0000	3.0000
			25	5.0000	-.0552	.2932	4.0000	5.0000
			50	9.0000	-.0020	.2618	8.0000	10.0000
			75	16.0000	.2332	.7182	15.0000	18.0000
			90	29.0000	.2904	1.8777	27.0000	34.0000
			95	43.0000	-.0142	1.9610	39.0000	47.4487
		2020	5	1.0000	.1006	.2901	1.0000	2.0000
			10	2.0000	.1426	.3633	2.0000	3.0000
			25	5.0000	-.3150	.4589	4.0000	5.0000
			50	7.0000	.1560	.3890	7.0000	8.0000
			75	14.0000	-.4590	1.0398	11.0000	16.0000
			90	23.0000	-.3916	1.0207	21.0000	25.2949
			95	29.0000	-.6050	1.9462	25.0000	32.0000
		2021	5	2.0000	-.2638	.4193	1.0000	2.0000
			10	3.0000	-.2417	.4288	2.0000	3.0000
			25	5.0000	-.2337	.4282	4.0000	5.0000
			50	8.0000	-.1755	.3809	7.0000	8.0000
			75	13.0000	.3047	.9829	11.7500	15.0000
			90	21.3000	.2578	1.8029	18.0000	27.0000
			95	28.1500	.6118	2.1906	23.0000	34.9949
		2022	5	1.0000	.4298	.4812	1.0000	2.0000
			10	2.0000	.3736	.4821	2.0000	3.0000
			25	5.0000	.0265	.2429	5.0000	6.0000

			50	8.0000	.0650	.2964	8.0000	9.0000
			75	13.0000	.3400	.8059	12.0000	15.0000
			90	21.0000	.4709	1.4498	20.0000	25.0000
			95	30.0000	-.5109	3.0958	25.0000	34.0000
		2023	5	1.0000	.0326	.1690	1.0000	1.8987
			10	2.0000	.0927	.3606	1.1000	3.0000
			25	5.0000	-.3908	.4884	4.0000	5.0000
			50	7.0000	.1150	.3455	7.0000	8.0000
			75	11.0000	-.0103	.8246	10.0000	13.0000
			90	18.8000	-.3097	1.2742	16.0000	21.3975
			95	23.0000	.5006	1.6233	20.9025	27.0000
Tukey's Hinges	Days_Hospitalization_per_yers_sum	2019	25	5.0000	-.0485	.2924	4.0000	5.0000
			50	9.0000	-.0020	.2618	8.0000	10.0000
			75	16.0000	.2145	.7168	15.0000	18.0000
		2020	25	5.0000	-.3040	.4569	4.0000	5.0000
			50	7.0000	.1560	.3890	7.0000	8.0000
			75	14.0000	-.5055	1.0459	11.0000	16.0000
		2021	25	5.0000	-.2185	.4227	4.0000	5.0000
			50	8.0000	-.1755	.3809	7.0000	8.0000
			75	13.0000	.2610	.9835	11.5000	15.0000
		2022	25	5.0000	.0320	.2481	5.0000	6.0000
			50	8.0000	.0650	.2964	8.0000	9.0000
			75	13.0000	.3095	.8143	12.0000	15.0000
		2023	25	5.0000	-.3720	.4892	4.0000	5.0000
			50	7.0000	.1150	.3455	7.0000	8.0000
			75	11.0000	-.0380	.8187	10.0000	13.0000

a. Unless otherwise noted, bootstrap results are based on 1000 bootstrap samples.

Table S16. Days of hospitalization per NALC per year.

Descriptives

	Non-alcoholic cirrhosis			Statistic	Std. Error	Bootstrap[a] Bias	Bootstrap[a] Std. Error	Bootstrap[a] 95% Confidence Interval Lower	Bootstrap[a] 95% Confidence Interval Upper
Days_Hospitalization_per_yers_sum	no	Mean		9.6155	.20375	.0013	.2005	9.2100	10.0255
		95% Confidence Interval for Mean	Lower Bound	9.2158					
			Upper Bound	10.0151					
		5% Trimmed Mean		8.6025		.0047	.1752	8.2597	8.9515
		Median		7.0000		.1420	.3471	7.0000	8.0000
		Variance		72.899		-.107	5.690	62.310	84.515
		Std. Deviation		8.53810		-.01277	.33296	7.89366	9.19321
		Minimum		1.00					
		Maximum		60.82					
		Range		59.82					
		Interquartile Range		8.00		-.05	.48	7.00	9.00
		Skewness		2.414	.058	-.015	.137	2.127	2.659
		Kurtosis		8.382	.117	-.099	.939	6.400	10.183
	yes	Mean		16.3747	.58398	.0043	.5903	15.2414	17.5666
		95% Confidence Interval for Mean	Lower Bound	15.2278					
			Upper Bound	17.5216					
		5% Trimmed Mean		14.8294		.0167	.6062	13.6988	16.1021

	Median	11.0000		.2115	.7229	10.0000	13.0000
	Variance	205.644		-.062	16.888	172.072	238.548
	Std. Deviation	14.34028		-.01430	.58998	13.11764	15.44501
	Minimum	1.00					
	Maximum	60.82					
	Range	59.82					
	Interquartile Range	14.00		.66	1.14	12.26	17.00
	Skewness	1.586	.100	-.001	.092	1.406	1.767
	Kurtosis	1.970	.199	.010	.418	1.210	2.863

a. Unless otherwise noted, bootstrap results are based on 1000 bootstrap samples

Percentiles

					Bootstrap[a]			
		Non-alcoholic cirrhosis	Percentiles	Percentile	Bias	Std. Error	95% Confidence Interval Lower	95% Confidence Interval Upper
Weighted Average(Definition 1)	Days_Hospitalization_per_yers_sum	no	5	1.0000	.0018	.0405	1.0000	1.0000
			10	2.0000	.0008	.0253	2.0000	2.0000
			25	4.0000	.0948	.2888	4.0000	5.0000
			50	7.0000	.1420	.3471	7.0000	8.0000
			75	12.0000	.0430	.4242	11.0000	13.0000
			90	20.0000	-.0489	.6850	19.0000	21.0000
			95	26.0000	.2049	.8395	24.1589	28.0000
		yes	5	3.0000	-.1139	.3082	2.0000	3.0000
			10	4.0000	-.0229	.2559	3.0000	4.1975
			25	7.0000	-.2913	.4483	6.0000	7.0000
			50	11.0000	.2115	.7229	10.0000	13.0000
			75	21.0000	.3715	1.1360	19.0000	23.0000
			90	38.6000	-.3816	2.5328	33.6000	43.0000

			95	51.8000	-.0577	3.2144	45.0000	57.0000
Tukey's Hinges	Days_Hospitalization_per_yers_sum	no	25	4.0000	.1000	.2985	4.0000	5.0000
			50	7.0000	.1420	.3471	7.0000	8.0000
			75	12.0000	.0385	.4224	11.0000	13.0000
		yes	25	7.0000	-.2760	.4416	6.0000	7.0000
			50	11.0000	.2115	.7229	10.0000	13.0000
			75	21.0000	.3305	1.1379	19.0000	23.0000

a. Unless otherwise noted, bootstrap results are based on 1000 bootstrap samples.

Descriptives

						Bootstrap[a]			
								95% Confidence Interval	
	YEAR_min			Statistic	Std. Error	Bias	Std. Error	Lower	Upper
Days_Hospitalization_per_yers_sum	2019	Mean		13.2466	.43005	.0123	.4253	12.4333	14.1080
		95% Confidence Interval for Mean	Lower Bound	12.4025					
			Upper Bound	14.0906					
		5% Trimmed Mean		11.6604		.0166	.4083	10.9055	12.5160
		Median		9.0000		.0130	.2375	9.0000	10.0000
		Variance		164.233		.137	13.167	138.655	190.757
		Std. Deviation		12.81535		-.00497	.51469	11.77520	13.81147
		Minimum		1.00					
		Maximum		60.82					
		Range		59.82					
		Interquartile Range		11.00		.29	.71	10.00	13.00
		Skewness		1.946	.082	-.003	.091	1.761	2.119
		Kurtosis		3.774	.164	.001	.507	2.844	4.787

	2020	Mean		10.3930	.49649	.0039	.5136	9.4731	11.4514
		95% Confidence Interval for Mean	Lower Bound	9.4167					
			Upper Bound	11.3694					
		5% Trimmed Mean		9.3135		.0160	.4493	8.4854	10.2233
		Median		7.0000		.1560	.4010	7.0000	8.0000
		Variance		89.974		-.266	14.951	63.329	121.409
		Std. Deviation		9.48548		-.04669	.78647	7.95795	11.01855
		Minimum		1.00					
		Maximum		60.82					
		Range		59.82					
		Interquartile Range		9.00		-.18	1.02	7.00	11.00
		Skewness		2.277	.128	-.056	.274	1.600	2.708
		Kurtosis		7.285	.255	-.370	1.696	3.148	10.012
	2021	Mean		10.4686	.48486	.0257	.4993	9.5061	11.5440
		95% Confidence Interval for Mean	Lower Bound	9.5150					
			Upper Bound	11.4222					
		5% Trimmed Mean		9.3789		.0349	.4382	8.5503	10.3165
		Median		8.0000		-.1330	.3632	7.0000	8.0000
		Variance		83.692		-.084	13.558	58.985	110.820
		Std. Deviation		9.14831		-.03475	.74258	7.68017	10.52713
		Minimum		1.00					
		Maximum		60.82					
		Range		59.82					

		Interquartile Range		8.00		.58	.94	7.00	10.00
		Skewness		2.308	.129	-.060	.259	1.708	2.740
		Kurtosis		7.112	.258	-.374	1.730	3.468	10.370
	2022	Mean		10.6935	.47115	-.0243	.4610	9.7528	11.5852
		95% Confidence Interval for Mean	Lower Bound	9.7673					
			Upper Bound	11.6197					
		5% Trimmed Mean		9.4976		-.0065	.3886	8.7079	10.2546
		Median		8.0000		.0470	.2956	8.0000	9.0000
		Variance		90.791		-.481	13.903	63.832	118.741
		Std. Deviation		9.52842		-.05352	.73222	7.98950	10.89683
		Minimum		1.00					
		Maximum		60.82					
		Range		59.82					
		Interquartile Range		8.00		.28	.76	7.00	10.00
		Skewness		2.453	.121	-.036	.217	1.947	2.807
		Kurtosis		7.901	.241	-.193	1.463	4.852	10.839
	2023	Mean		9.0962	.42914	.0123	.4295	8.3030	9.9701
		95% Confidence Interval for Mean	Lower Bound	8.2522					
			Upper Bound	9.9403					
		5% Trimmed Mean		8.1812		.0199	.3614	7.5268	8.9870
		Median		7.0000		.1240	.3602	7.0000	8.0000
		Variance		62.798		.010	12.297	39.890	89.324
		Std. Deviation		7.92449		-.03776	.77957	6.31585	9.45116

	Minimum	1.00					
	Maximum	60.82					
	Range	59.82					
	Interquartile Range	6.00		.42	.86	5.00	8.00
	Skewness	2.693	.132	-.115	.395	1.632	3.236
	Kurtosis	11.026	.263	-.901	2.885	3.690	15.603

a. Unless otherwise noted, bootstrap results are based on 1000 bootstrap samples.

Percentiles

					Bootstrap[a]			
							95% Confidence Interval	
		YEAR_min	Percentiles	Percentile	Bias	Std. Error	Lower	Upper
Weighted Average(Definition 1)	Days_Hospitalization_per_yers _sum	2019	5	2.0000	-.3156	.4535	1.0000	2.0000
			10	2.0000	.2587	.4298	2.0000	3.0000
			25	5.0000	-.0538	.2748	4.0000	5.0000
			50	9.0000	.0130	.2375	9.0000	10.0000
			75	16.0000	.2320	.7100	15.0000	18.0000
			90	29.0000	.3587	1.8963	27.0000	34.0000
			95	43.0000	.0153	1.9981	39.0000	47.9848
		2020	5	1.0000	.1004	.2901	1.0000	2.0000
			10	2.0000	.1266	.3450	2.0000	3.0000
			25	5.0000	-.2875	.4463	4.0000	5.0000
			50	7.0000	.1560	.4010	7.0000	8.0000
			75	14.0000	-.4682	1.0366	11.0000	16.0000
			90	23.0000	-.3663	1.0643	21.0000	25.6949
			95	29.0000	-.5076	1.9808	25.0000	32.1987
		2021	5	2.0000	-.2714	.4334	1.0000	2.0000
			10	3.0000	-.2576	.4434	2.0000	3.0000

			25	5.0000	-.2235	.4236	4.0000	5.0000
			50	8.0000	-.1330	.3632	7.0000	8.0000
			75	13.0000	.3555	.9712	12.0000	15.0000
			90	21.3000	.2691	1.8299	18.0000	27.0000
			95	28.1500	.6372	2.1509	24.0000	34.7309
		2022	5	1.0000	.4358	.4829	1.0000	2.0000
			10	2.0000	.3301	.4634	2.0000	3.0000
			25	5.0000	.0243	.2565	4.7564	6.0000
			50	8.0000	.0470	.2956	8.0000	9.0000
			75	13.0000	.3078	.7663	12.0000	15.0000
			90	21.0000	.4364	1.4215	20.0000	25.0000
			95	30.0000	-.5175	3.1657	25.0000	34.0000
		2023	5	1.0000	.0268	.1498	1.0000	1.5500
			10	2.0000	.0670	.3729	1.0000	3.0000
			25	5.0000	-.4258	.4913	4.0000	5.0000
			50	7.0000	.1240	.3602	7.0000	8.0000
			75	11.0000	-.0040	.8136	10.0000	13.0000
			90	18.8000	-.2339	1.2618	16.0000	21.2975
			95	23.0000	.4863	1.5980	20.8513	27.0000
Tukey's Hinges	Days_Hospitalization_per_yers_sum	2019	25	5.0000	-.0485	.2720	4.0000	5.0000
			50	9.0000	.0130	.2375	9.0000	10.0000
			75	16.0000	.2120	.7050	15.0000	18.0000
		2020	25	5.0000	-.2725	.4441	4.0000	5.0000
			50	7.0000	.1560	.4010	7.0000	8.0000
			75	14.0000	-.5115	1.0441	11.0000	16.0000
		2021	25	5.0000	-.2090	.4177	4.0000	5.0000
			50	8.0000	-.1330	.3632	7.0000	8.0000

			75	13.0000	.3200	.9732	12.0000	15.0000
		2022	25	5.0000	.0315	.2633	5.0000	6.0000
			50	8.0000	.0470	.2956	8.0000	9.0000
			75	13.0000	.2740	.7701	12.0000	14.9873
		2023	25	5.0000	-.4030	.4882	4.0000	5.0000
			50	7.0000	.1240	.3602	7.0000	8.0000
			75	11.0000	-.0400	.8112	10.0000	13.0000

a. Unless otherwise noted, bootstrap results are based on 1000 bootstrap samples.

Table S17. Days of hospitalization per ALC per year.

Descriptives

	Alcoholic cirrhosis (1=yes, 0=no)			Statistic	Std. Error	Bootstrap[a] Bias	Std. Error	95% Confidence Interval Lower	Upper
Days_Hospitalization_per_yers_sum	no	Mean		10.5610	.22203	-.0059	.2200	10.1227	10.9769
		95% Confidence Interval for Mean	Lower Bound	10.1256					
			Upper Bound	10.9964					
		5% Trimmed Mean		9.2026		.0015	.1876	8.8437	9.5783
		Median		8.0000		-.3565	.4774	7.0000	8.0000
		Variance		103.471		-.398	6.904	89.642	116.294
		Std. Deviation		10.17207		-.02531	.34075	9.46797	10.78397
		Minimum		1.00					
		Maximum		60.82					
		Range		59.82					
		Interquartile Range		8.00		.46	.58	7.50	9.00

				Statistic	Std. Error	Bias	Std. Error	Lower	Upper
		Skewness		2.486	.053	-.006	.083	2.318	2.646
		Kurtosis		7.585	.107	-.008	.576	6.517	8.744
	yes	Mean		17.6581	.80380	.0630	.7866	16.2225	19.2679
		95% Confidence Interval for Mean	Lower Bound	16.0753					
			Upper Bound	19.2409					
		5% Trimmed Mean		16.3120		.0930	.7868	14.9177	17.9652
		Median		14.0000		.4490	.8886	13.0000	16.0000
		Variance		167.986		.881	21.197	129.036	214.472
		Std. Deviation		12.96094		.00826	.81696	11.35939	14.64488
		Minimum		2.00					
		Maximum		60.82					
		Range		58.82					
		Interquartile Range		14.00		.48	1.08	13.00	17.00
		Skewness		1.520	.151	-.015	.127	1.255	1.764
		Kurtosis		2.310	.301	-.040	.593	1.253	3.573

a. Unless otherwise noted, bootstrap results are based on 1000 bootstrap samples.

Percentiles

		Alcoholic cirrhosis (1=yes, 0=no)	Percentiles	Percentile	Bootstrap[a]			
							95% Confidence Interval	
					Bias	Std. Error	Lower	Upper
Weighted Average(Definition 1)	Days_Hospitalization_per_yers_sum	no	5	1.0000	.0261	.1555	1.0000	1.6449
			10	2.0000	.0036	.0570	2.0000	2.0000
			25	5.0000	-.4467	.4939	4.0000	5.0000

			50	8.0000	-.3565	.4774	7.0000	8.0000
			75	13.0000	.0103	.4234	12.0000	14.0000
			90	22.0000	-.0096	.8247	20.0000	23.0000
			95	30.0000	.3825	1.3464	28.0000	34.0000
		yes	5	4.0000	-.3351	.5860	3.0000	5.0000
			10	5.0000	.1015	.4477	4.0000	6.0000
			25	9.0000	-.3850	.6176	7.0064	10.0000
			50	14.0000	.4490	.8886	13.0000	16.0000
			75	23.0000	.0970	1.0504	21.0000	25.0000
			90	34.0000	.2433	3.2093	28.0000	43.0000
			95	49.9000	-1.1535	4.6644	38.5013	56.7949
Tukey's Hinges	Days_Hospitalization_per_yers _sum	no	25	5.0000	-.4385	.4937	4.0000	5.0000
			50	8.0000	-.3565	.4774	7.0000	8.0000
			75	13.0000	.0025	.4254	12.0000	14.0000
		yes	25	9.0000	-.3525	.6184	7.5000	10.0000
			50	14.0000	.4490	.8886	13.0000	16.0000
			75	23.0000	.0435	1.0358	21.0000	25.0000

a. Unless otherwise noted, bootstrap results are based on 1000 bootstrap samples.

Descriptives

						Bootstrap[a]			
								95% Confidence Interval	
	YEAR_min			Statistic	Std. Error	Bias	Std. Error	Lower	Upper
Days_Hospitalization_per_yers _sum	2019	Mean		13.2466	.43005	.0122	.4099	12.4481	14.0331
		95% Confidence Interval for Mean	Lower Bound	12.4025					
			Upper Bound	14.0906					

	5% Trimmed Mean		11.6604		.0180	.3961	10.8920	12.4298
	Median		9.0000		.0020	.2357	8.0000	10.0000
	Variance		164.233		-.138	12.536	139.283	188.722
	Std. Deviation		12.81535		-.01475	.49005	11.80184	13.73763
	Minimum		1.00					
	Maximum		60.82					
	Range		59.82					
	Interquartile Range		11.00		.30	.70	10.00	13.00
	Skewness		1.946	.082	-.005	.087	1.774	2.120
	Kurtosis		3.774	.164	-.006	.479	2.851	4.820
2020	Mean		10.3930	.49649	-.0048	.4916	9.4370	11.3285
	95% Confidence Interval for Mean	Lower Bound	9.4167					
		Upper Bound	11.3694					
	5% Trimmed Mean		9.3135		.0071	.4386	8.4797	10.1841
	Median		7.0000		.1450	.3836	7.0000	8.0000
	Variance		89.974		-.395	14.052	62.776	117.829
	Std. Deviation		9.48548		-.05017	.74484	7.92316	10.85489
	Minimum		1.00					
	Maximum		60.82					
	Range		59.82					
	Interquartile Range		9.00		-.22	1.02	7.00	11.00
	Skewness		2.277	.128	-.042	.270	1.617	2.699
	Kurtosis		7.285	.255	-.264	1.701	3.245	10.239
2021	Mean		10.4686	.48486	-.0194	.4669	9.6153	11.3806

		95% Confidence Interval for Mean	Lower Bound	9.5150					
			Upper Bound	11.4222					
		5% Trimmed Mean		9.3789		-.0076	.4103	8.5930	10.1649
		Median		8.0000		-.1690	.3874	7.0000	8.0000
		Variance		83.692		-.580	13.040	59.552	109.508
		Std. Deviation		9.14831		-.05987	.71596	7.71703	10.46459
		Minimum		1.00					
		Maximum		60.82					
		Range		59.82					
		Interquartile Range		8.00		.46	.96	7.00	10.00
		Skewness		2.308	.129	-.049	.260	1.729	2.786
		Kurtosis		7.112	.258	-.294	1.708	3.538	10.604
	2022	Mean		10.6935	.47115	.0082	.4727	9.8085	11.6586
		95% Confidence Interval for Mean	Lower Bound	9.7673					
			Upper Bound	11.6197					
		5% Trimmed Mean		9.4976		.0248	.4061	8.7445	10.3145
		Median		8.0000		.0660	.3150	8.0000	9.0000
		Variance		90.791		-.062	14.051	65.316	120.904
		Std. Deviation		9.52842		-.03165	.73545	8.08184	10.99562
		Minimum		1.00					
		Maximum		60.82					
		Range		59.82					
		Interquartile Range		8.00		.33	.79	7.00	10.00

		Skewness		2.453	.121	-.042	.210	1.999	2.810
		Kurtosis		7.901	.241	-.226	1.399	5.057	10.639
	2023	Mean		9.0962	.42914	-.0067	.4254	8.2794	9.9899
		95% Confidence Interval for Mean	Lower Bound	8.2522					
			Upper Bound	9.9403					
		5% Trimmed Mean		8.1812		.0063	.3613	7.5080	8.9256
		Median		7.0000		.0995	.3293	7.0000	8.0000
		Variance		62.798		-.409	11.726	42.292	87.882
		Std. Deviation		7.92449		-.06050	.73916	6.50320	9.37457
		Minimum		1.00					
		Maximum		60.82					
		Range		59.82					
		Interquartile Range		6.00		.36	.83	5.00	8.00
		Skewness		2.693	.132	-.105	.377	1.721	3.219
		Kurtosis		11.026	.263	-.798	2.777	4.135	15.579

a. Unless otherwise noted, bootstrap results are based on 1000 bootstrap samples.

Percentiles

					Bootstrap[a]			
							95% Confidence Interval	
		YEAR_min	Percentiles	Percentile	Bias	Std. Error	Lower	Upper
Weighted Average(Definition 1)	Days_Hospitalization_per_yers_sum	2019	5	2.0000	-.2944	.4475	1.0000	2.0000
			10	2.0000	.2396	.4179	2.0000	3.0000
			25	5.0000	-.0485	.2600	4.0000	5.0000

			50	9.0000	.0020	.2357	8.0000	10.0000
			75	16.0000	.2527	.7038	15.0000	18.0000
			90	29.0000	.3088	1.8225	27.0000	34.0000
			95	43.0000	.0764	1.8338	39.5051	46.9987
		2020	5	1.0000	.1028	.2958	1.0000	2.0000
			10	2.0000	.1317	.3559	2.0000	3.0000
			25	5.0000	-.2917	.4475	4.0000	5.0000
			50	7.0000	.1450	.3836	7.0000	8.0000
			75	14.0000	-.5125	1.0150	11.0064	15.7436
			90	23.0000	-.4190	1.0705	21.0000	25.0000
			95	29.0000	-.5515	2.0277	25.0000	32.3500
		2021	5	2.0000	-.2706	.4328	1.0000	2.0000
			10	3.0000	-.2769	.4440	2.0000	3.0000
			25	5.0000	-.2300	.4215	4.0000	5.0000
			50	8.0000	-.1690	.3874	7.0000	8.0000
			75	13.0000	.2345	.9628	11.5064	15.0000
			90	21.3000	.2264	1.7274	18.0000	26.5949
			95	28.1500	.5526	2.1534	24.0000	35.0000
		2022	5	1.0000	.4140	.4754	1.0000	2.0000
			10	2.0000	.3557	.4835	2.0000	3.0000
			25	5.0000	.0243	.2675	4.5064	6.0000
			50	8.0000	.0660	.3150	8.0000	9.0000
			75	13.0000	.3557	.7973	12.0000	15.0000
			90	21.0000	.4632	1.4392	20.0000	25.0000
			95	30.0000	-.4649	3.1117	25.0000	34.0000
		2023	5	1.0000	.0326	.1690	1.0000	1.9987
			10	2.0000	.0600	.3877	1.0000	3.0000

			25	5.0000	-.3872	.4872	4.0000	5.0000
			50	7.0000	.0995	.3293	7.0000	8.0000
			75	11.0000	-.0285	.7839	10.0000	13.0000
			90	18.8000	-.3025	1.2325	16.0000	21.1000
			95	23.0000	.4241	1.5167	20.9025	27.0000
Tukey's Hinges	Days_Hospitalization_per_yers_sum	2019	25	5.0000	-.0445	.2595	4.0000	5.0000
			50	9.0000	.0020	.2357	8.0000	10.0000
			75	16.0000	.2315	.7019	15.0000	18.0000
		2020	25	5.0000	-.2730	.4384	4.0000	5.0000
			50	7.0000	.1450	.3836	7.0000	8.0000
			75	14.0000	-.5560	1.0234	11.0000	15.4873
		2021	25	5.0000	-.2180	.4161	4.0000	5.0000
			50	8.0000	-.1690	.3874	7.0000	8.0000
			75	13.0000	.1940	.9630	11.0127	15.0000
		2022	25	5.0000	.0300	.2668	5.0000	6.0000
			50	8.0000	.0660	.3150	8.0000	9.0000
			75	13.0000	.3205	.8063	12.0000	15.0000
		2023	25	5.0000	-.3725	.4840	4.0000	5.0000
			50	7.0000	.0995	.3293	7.0000	8.0000
			75	11.0000	-.0630	.7727	10.0000	13.0000

a. Unless otherwise noted, bootstrap results are based on 1000 bootstrap samples.

Table S18. Generalized Linear Model (Gamma distribution, log link).

Days_Hospitalization_per_yers_sum

		Frequency	Percent	Valid Percent	Cumulative Percent
Valid	1.00	126	5.3	5.3	5.3

	2.00	128	5.4	5.4	10.8
	3.00	129	5.5	5.5	16.2
	4.00	158	6.7	6.7	22.9
	5.00	175	7.4	7.4	30.4
	6.00	190	8.1	8.1	38.4
	7.00	187	7.9	7.9	46.4
	8.00	171	7.2	7.3	53.6
	9.00	152	6.4	6.4	60.1
	10.00	110	4.7	4.7	64.7
	11.00	71	3.0	3.0	67.7
	12.00	64	2.7	2.7	70.4
	13.00	59	2.5	2.5	72.9
	14.00	66	2.8	2.8	75.7
	15.00	65	2.8	2.8	78.5
	16.00	52	2.2	2.2	80.7
	17.00	39	1.7	1.7	82.4
	18.00	37	1.6	1.6	83.9
	19.00	26	1.1	1.1	85.0
	20.00	31	1.3	1.3	86.3
	21.00	28	1.2	1.2	87.5
	22.00	25	1.1	1.1	88.6
	23.00	30	1.3	1.3	89.9
	24.00	14	.6	.6	90.5
	25.00	16	.7	.7	91.1
	26.00	18	.8	.8	91.9
	27.00	18	.8	.8	92.7
	28.00	18	.8	.8	93.4

	29.00	10	.4	.4	93.9
	30.00	11	.5	.5	94.3
	31.00	8	.3	.3	94.7
	32.00	5	.2	.2	94.9
	33.00	7	.3	.3	95.2
	34.00	9	.4	.4	95.5
	35.00	6	.3	.3	95.8
	36.00	4	.2	.2	96.0
	37.00	5	.2	.2	96.2
	38.00	4	.2	.2	96.4
	39.00	2	.1	.1	96.4
	40.00	5	.2	.2	96.6
	41.00	8	.3	.3	97.0
	42.00	3	.1	.1	97.1
	43.00	5	.2	.2	97.3
	44.00	5	.2	.2	97.5
	45.00	4	.2	.2	97.7
	46.00	2	.1	.1	97.8
	47.00	1	.0	.0	97.8
	48.00	3	.1	.1	98.0
	49.00	2	.1	.1	98.0
	50.00	3	.1	.1	98.2
	51.00	4	.2	.2	98.3
	52.00	2	.1	.1	98.4
	53.00	3	.1	.1	98.6
	54.00	2	.1	.1	98.6
	55.00	1	.0	.0	98.7

	56.00	1	.0	.0	98.7
	57.00	4	.2	.2	98.9
	58.00	1	.0	.0	98.9
	60.00	2	.1	.1	99.0
	62.00	2	.1	.1	99.1
	64.00	1	.0	.0	99.2
	65.00	1	.0	.0	99.2
	66.00	2	.1	.1	99.3
	68.00	1	.0	.0	99.3
	69.00	1	.0	.0	99.4
	70.00	1	.0	.0	99.4
	72.00	2	.1	.1	99.5
	73.00	1	.0	.0	99.5
	81.00	1	.0	.0	99.6
	94.00	1	.0	.0	99.6
	95.00	1	.0	.0	99.7
	100.00	1	.0	.0	99.7
	105.00	1	.0	.0	99.7
	106.00	1	.0	.0	99.8
	135.00	1	.0	.0	99.8
	137.00	1	.0	.0	99.9
	141.00	1	.0	.0	99.9
	147.00	1	.0	.0	100.0
	164.00	1	.0	.0	100.0
	Total	2358	100.0	100.0	
Missing	System	1	.0		
Total		2359	100.0		

Case Processing Summary

	N	Percent
Included	2358	100.0%
Excluded	1	0.0%
Total	2359	100.0%

Categorical Variable Information

			N	Percent
Factor	SEX_first	FEMALE	848	36.0%
		MALE	1510	64.0%
		Total	2358	100.0%
	Chronic hepatitis C (1=yes, 0=no)	no	1389	58.9%
		yes	969	41.1%
		Total	2358	100.0%
	Alcoholic hepatitis (1=yes, 0=no)	no	1405	59.6%
		yes	953	40.4%
		Total	2358	100.0%
	Non-alcoholic cirrhosis	no	1755	74.4%
		yes	603	25.6%
		Total	2358	100.0%
	Alcoholic cirrhosis (1=yes, 0=no)	no	2098	89.0%
		yes	260	11.0%
		Total	2358	100.0%

Continuous Variable Information

		N	Minimum	Maximum	Mean	Std. Deviation
Dependent Variable	Days_Hospitalization_per_years_sum	2358	1.00	60.82	11.3463	10.74621
Covariate	AGE_min	2358	25	95	61.71	12.501

Child–Pugh	2358	0	3	1.47	.744

Goodness of Fit[a]

	Value	df	Value/df
Deviance	1412.947	2350	.601
Scaled Deviance	2568.397	2350	
Pearson Chi-Square	1672.857	2350	.712
Scaled Pearson Chi-Square	3040.848	2350	
Log Likelihood[b]	-7704.087		
Akaike's Information Criterion (AIC)	15426.174		
Finite Sample Corrected AIC (AICC)	15426.250		
Bayesian Information Criterion (BIC)	15478.064		
Consistent AIC (CAIC)	15487.064		

Dependent Variable: Days_Hospitalization_per_yers_sum.
Model: (Intercept), Chronic hepatitis C (1=yes, 0=no), Alcoholic hepatitis (1=yes, 0=no), Non-alcoholic cirrhosis, Alcoholic cirrhosis (1=yes, 0=no), Child–Pugh , SEX_first, AGE_min[a]

a. Information criteria are in smaller-is-better form.

b. The full log likelihood function is displayed and used in computing information criteria.

Omnibus Test[a]

Likelihood Ratio Chi-Square	df	Sig.
559.374	7	.000

Dependent Variable:
Days_Hospitalization_per_yers_sum
Model: (Intercept), Chronic hepatitis C (1=yes, 0=no), Alcoholic hepatitis (1=yes, 0=no), Non-alcoholic cirrhosis, Alcoholic cirrhosis (1=yes, 0=no), Child–Pugh , SEX_first, AGE_min[a]

a. Compares the fitted model against the intercept-only model.

Tests of Model Effects

Source	Type III Wald Chi-Square	df	Sig.
(Intercept)	777.544	1	.000
Chronic hepatitis C (1=yes, 0=no)	162.394	1	.000
Alcoholic hepatitis (1=yes, 0=no)	220.100	1	.000
Non-alcoholic cirrhosis	381.241	1	.000
Alcoholic cirrhosis (1=yes, 0=no)	144.333	1	.000
Child–Pugh	.112	1	.738
SEX_first	1.021	1	.312
AGE_min	8.776	1	.003

Dependent Variable: Days_Hospitalization_per_yers_sum
Model: (Intercept), Chronic hepatitis C (1=yes, 0=no), Alcoholic hepatitis (1=yes, 0=no), Non-alcoholic cirrhosis, Alcoholic cirrhosis (1=yes, 0=no), Child–Pugh , SEX_first, AGE_min

Parameter Estimates

Parameter	B	Std. Error	95% Wald Confidence Interval		Hypothesis Test		
			Lower	Upper	Wald Chi-Square	df	Sig.
(Intercept)	4.245	.1405	3.970	4.521	912.367	1	.000
[Chronic hepatitis C (1=yes, 0=no)=.00]	-.784	.0615	-.904	-.663	162.394	1	.000
[Chronic hepatitis C (1=yes, 0=no)=1.00]	0[a]	.	.	.	.	.	.
[Alcoholic hepatitis (1=yes, 0=no)=.00]	-.750	.0506	-.850	-.651	220.100	1	.000
[Alcoholic hepatitis (1=yes, 0=no)=1.00]	0[a]	.	.	.	.	.	.
[Non-alcoholic cirrhosis=.00]	-.940	.0481	-1.034	-.846	381.241	1	.000
[Non-alcoholic cirrhosis=1.00]	0[a]	.	.	.	.	.	.
[Alcoholic cirrhosis (1=yes, 0=no)=.00]	-.626	.0521	-.728	-.524	144.333	1	.000
[Alcoholic cirrhosis (1=yes, 0=no)=1.00]	0[a]	.	.	.	.	.	.
Child–Pugh	.007	.0224	-.036	.051	.112	1	.738
[SEX_first=1.00]	.040	.0398	-.038	.118	1.021	1	.312
[SEX_first=2.00]	0[a]	.	.	.	.	.	.
AGE_min	.004	.0014	.001	.007	8.776	1	.003
(Scale)	.550[b]	.0148	.522	.580			

Dependent Variable: Days_Hospitalization_per_yers_sum

Model: (Intercept), Chronic hepatitis C (1=yes, 0=no), Alcoholic hepatitis (1=yes, 0=no), Non-alcoholic cirrhosis, Alcoholic cirrhosis (1=yes, 0=no), Child–Pugh , SEX_first, AGE_min

a. Set to zero because this parameter is redundant.

b. Maximum likelihood estimate.

Table S19. Sex-Based CHC Patterns.

Crosstab

YEAR_min				Chronic hepatitis C (1=yes, 0=no) no	Chronic hepatitis C (1=yes, 0=no) yes	Total
2019	SEX_first	FEMALE	Count	77	258	335
			% within SEX_first	23.0%	77.0%	100.0%
			% within Chronic hepatitis C (1=yes, 0=no)	15.0%	69.0%	37.7%
			% of Total	8.7%	29.1%	37.7%
		MALE	Count	437	116	553
			% within SEX_first	79.0%	21.0%	100.0%
			% within Chronic hepatitis C (1=yes, 0=no)	85.0%	31.0%	62.3%
			% of Total	49.2%	13.1%	62.3%
	Total		Count	514	374	888
			% within SEX_first	57.9%	42.1%	100.0%
			% within Chronic hepatitis C (1=yes, 0=no)	100.0%	100.0%	100.0%
			% of Total	57.9%	42.1%	100.0%
2020	SEX_first	FEMALE	Count	30	117	147
			% within SEX_first	20.4%	79.6%	100.0%
			% within Chronic hepatitis C (1=yes, 0=no)	15.2%	69.6%	40.3%
			% of Total	8.2%	32.1%	40.3%
		MALE	Count	167	51	218
			% within SEX_first	76.6%	23.4%	100.0%

			% within Chronic hepatitis C (1=yes, 0=no)	84.8%	30.4%	59.7%
			% of Total	45.8%	14.0%	59.7%
	Total		Count	197	168	365
			% within SEX_first	54.0%	46.0%	100.0%
			% within Chronic hepatitis C (1=yes, 0=no)	100.0%	100.0%	100.0%
			% of Total	54.0%	46.0%	100.0%
2021	SEX_first	FEMALE	Count	21	82	103
			% within SEX_first	20.4%	79.6%	100.0%
			% within Chronic hepatitis C (1=yes, 0=no)	9.6%	59.9%	28.9%
			% of Total	5.9%	23.0%	28.9%
		MALE	Count	198	55	253
			% within SEX_first	78.3%	21.7%	100.0%
			% within Chronic hepatitis C (1=yes, 0=no)	90.4%	40.1%	71.1%
			% of Total	55.6%	15.4%	71.1%
	Total		Count	219	137	356
			% within SEX_first	61.5%	38.5%	100.0%
			% within Chronic hepatitis C (1=yes, 0=no)	100.0%	100.0%	100.0%
			% of Total	61.5%	38.5%	100.0%
2022	SEX_first	FEMALE	Count	44	95	139
			% within SEX_first	31.7%	68.3%	100.0%
			% within Chronic hepatitis C (1=yes, 0=no)	16.5%	66.4%	34.0%

			% of Total	10.8%	23.2%	34.0%
		MALE	Count	222	48	270
			% within SEX_first	82.2%	17.8%	100.0%
			% within Chronic hepatitis C (1=yes, 0=no)	83.5%	33.6%	66.0%
			% of Total	54.3%	11.7%	66.0%
	Total		Count	266	143	409
			% within SEX_first	65.0%	35.0%	100.0%
			% within Chronic hepatitis C (1=yes, 0=no)	100.0%	100.0%	100.0%
			% of Total	65.0%	35.0%	100.0%
2023	SEX_first	FEMALE	Count	29	96	125
			% within SEX_first	23.2%	76.8%	100.0%
			% within Chronic hepatitis C (1=yes, 0=no)	14.9%	65.3%	36.7%
			% of Total	8.5%	28.2%	36.7%
		MALE	Count	165	51	216
			% within SEX_first	76.4%	23.6%	100.0%
			% within Chronic hepatitis C (1=yes, 0=no)	85.1%	34.7%	63.3%
			% of Total	48.4%	15.0%	63.3%
	Total		Count	194	147	341
			% within SEX_first	56.9%	43.1%	100.0%
			% within Chronic hepatitis C (1=yes, 0=no)	100.0%	100.0%	100.0%
			% of Total	56.9%	43.1%	100.0%
Total	SEX_first	FEMALE	Count	201	648	849

			% within SEX_first	23.7%	76.3%	100.0%
			% within Chronic hepatitis C (1=yes, 0=no)	14.5%	66.9%	36.0%
			% of Total	8.5%	27.5%	36.0%
		MALE	Count	1189	321	1510
			% within SEX_first	78.7%	21.3%	100.0%
			% within Chronic hepatitis C (1=yes, 0=no)	85.5%	33.1%	64.0%
			% of Total	50.4%	13.6%	64.0%
	Total		Count	1390	969	2359
			% within SEX_first	58.9%	41.1%	100.0%
			% within Chronic hepatitis C (1=yes, 0=no)	100.0%	100.0%	100.0%
			% of Total	58.9%	41.1%	100.0%

Chi-Square Tests

YEAR_min		Value	df	Asymptotic Significance (2-sided)	Exact Sig. (2-sided)	Exact Sig. (1-sided)
2019	Pearson Chi-Square	268.732[c]	1	.000		
	Continuity Correction[b]	266.439	1	.000		
	Likelihood Ratio	279.579	1	.000		
	Fisher's Exact Test				.000	.000
	Linear-by-Linear Association	268.430	1	.000		
	N of Valid Cases	888				
2020	Pearson Chi-Square	111.615[d]	1	.000		
	Continuity Correction[b]	109.364	1	.000		
	Likelihood Ratio	117.741	1	.000		

	Fisher's Exact Test				.000	.000
	Linear-by-Linear Association	111.309	1	.000		
	N of Valid Cases	365				
2021	Pearson Chi-Square	103.559[e]	1	.000		
	Continuity Correction[b]	101.129	1	.000		
	Likelihood Ratio	105.346	1	.000		
	Fisher's Exact Test				.000	.000
	Linear-by-Linear Association	103.268	1	.000		
	N of Valid Cases	356				
2022	Pearson Chi-Square	103.188[f]	1	.000		
	Continuity Correction[b]	100.976	1	.000		
	Likelihood Ratio	103.163	1	.000		
	Fisher's Exact Test				.000	.000
	Linear-by-Linear Association	102.935	1	.000		
	N of Valid Cases	409				
2023	Pearson Chi-Square	91.336[g]	1	.000		
	Continuity Correction[b]	89.180	1	.000		
	Likelihood Ratio	94.695	1	.000		
	Fisher's Exact Test				.000	.000
	Linear-by-Linear Association	91.068	1	.000		
	N of Valid Cases	341				
Total	Pearson Chi-Square	680.854[a]	1	.000		
	Continuity Correction[b]	678.581	1	.000		
	Likelihood Ratio	702.986	1	.000		
	Fisher's Exact Test				.000	.000
	Linear-by-Linear Association	680.565	1	.000		
	N of Valid Cases	2359				

a. 0 cells (0.0%) have an expected count of less than 5. The minimum expected count is 348.74.

b. Computed only for a 2x2 table.

c. 0 cells (0.0%) have an expected count of less than 5. The minimum expected count is 141.09.

d. 0 cells (0.0%) have an expected count of less than 5. The minimum expected count is 67.66.

e. 0 cells (0.0%) have an expected count of less than 5. The minimum expected count is 39.64.

f. 0 cells (0.0%) have an expected count of less than 5. The minimum expected count is 48.60.

g. 0 cells (0.0%) have an expected count of less than 5. The minimum expected count is 53.89.

Table S20. Sex-Based ALH Patterns.

Crosstab

YEAR_min				Alcoholic hepatitis (1=yes, 0=no): no	Alcoholic hepatitis (1=yes, 0=no): yes	Total
2019	SEX_first	FEMALE	Count	274	61	335
			% within SEX_first	81.8%	18.2%	100.0%
			% within Alcoholic hepatitis (1=yes, 0=no)	57.3%	14.9%	37.7%
			% of Total	30.9%	6.9%	37.7%
		MALE	Count	204	349	553
			% within SEX_first	36.9%	63.1%	100.0%
			% within Alcoholic hepatitis (1=yes, 0=no)	42.7%	85.1%	62.3%
			% of Total	23.0%	39.3%	62.3%
	Total		Count	478	410	888
			% within SEX_first	53.8%	46.2%	100.0%
			% within Alcoholic hepatitis (1=yes, 0=no)	100.0%	100.0%	100.0%
			% of Total	53.8%	46.2%	100.0%

2020	SEX_first	FEMALE	Count	137	10	147
			% within SEX_first	93.2%	6.8%	100.0%
			% within Alcoholic hepatitis (1=yes, 0=no)	55.9%	8.3%	40.3%
			% of Total	37.5%	2.7%	40.3%
		MALE	Count	108	110	218
			% within SEX_first	49.5%	50.5%	100.0%
			% within Alcoholic hepatitis (1=yes, 0=no)	44.1%	91.7%	59.7%
			% of Total	29.6%	30.1%	59.7%
	Total		Count	245	120	365
			% within SEX_first	67.1%	32.9%	100.0%
			% within Alcoholic hepatitis (1=yes, 0=no)	100.0%	100.0%	100.0%
			% of Total	67.1%	32.9%	100.0%
2021	SEX_first	FEMALE	Count	96	7	103
			% within SEX_first	93.2%	6.8%	100.0%
			% within Alcoholic hepatitis (1=yes, 0=no)	42.1%	5.5%	28.9%
			% of Total	27.0%	2.0%	28.9%
		MALE	Count	132	121	253
			% within SEX_first	52.2%	47.8%	100.0%
			% within Alcoholic hepatitis (1=yes, 0=no)	57.9%	94.5%	71.1%
			% of Total	37.1%	34.0%	71.1%
	Total		Count	228	128	356
			% within SEX_first	64.0%	36.0%	100.0%

			% within Alcoholic hepatitis (1=yes, 0=no)	100.0%	100.0%	100.0%
			% of Total	64.0%	36.0%	100.0%
2022	SEX_first	FEMALE	Count	117	22	139
			% within SEX_first	84.2%	15.8%	100.0%
			% within Alcoholic hepatitis (1=yes, 0=no)	50.4%	12.4%	34.0%
			% of Total	28.6%	5.4%	34.0%
		MALE	Count	115	155	270
			% within SEX_first	42.6%	57.4%	100.0%
			% within Alcoholic hepatitis (1=yes, 0=no)	49.6%	87.6%	66.0%
			% of Total	28.1%	37.9%	66.0%
	Total		Count	232	177	409
			% within SEX_first	56.7%	43.3%	100.0%
			% within Alcoholic hepatitis (1=yes, 0=no)	100.0%	100.0%	100.0%
			% of Total	56.7%	43.3%	100.0%
2023	SEX_first	FEMALE	Count	116	9	125
			% within SEX_first	92.8%	7.2%	100.0%
			% within Alcoholic hepatitis (1=yes, 0=no)	52.3%	7.6%	36.7%
			% of Total	34.0%	2.6%	36.7%
		MALE	Count	106	110	216
			% within SEX_first	49.1%	50.9%	100.0%
			% within Alcoholic hepatitis (1=yes, 0=no)	47.7%	92.4%	63.3%

			% of Total	31.1%	32.3%	63.3%
	Total		Count	222	119	341
			% within SEX_first	65.1%	34.9%	100.0%
			% within Alcoholic hepatitis (1=yes, 0=no)	100.0%	100.0%	100.0%
			% of Total	65.1%	34.9%	100.0%
Total	SEX_first	FEMALE	Count	740	109	849
			% within SEX_first	87.2%	12.8%	100.0%
			% within Alcoholic hepatitis (1=yes, 0=no)	52.7%	11.4%	36.0%
			% of Total	31.4%	4.6%	36.0%
		MALE	Count	665	845	1510
			% within SEX_first	44.0%	56.0%	100.0%
			% within Alcoholic hepatitis (1=yes, 0=no)	47.3%	88.6%	64.0%
			% of Total	28.2%	35.8%	64.0%
	Total		Count	1405	954	2359
			% within SEX_first	59.6%	40.4%	100.0%
			% within Alcoholic hepatitis (1=yes, 0=no)	100.0%	100.0%	100.0%
			% of Total	59.6%	40.4%	100.0%

Chi-Square Tests

YEAR_min		Value	df	Asymptotic Significance (2-sided)	Exact Sig. (2-sided)	Exact Sig. (1-sided)
2019	Pearson Chi-Square	169.235[c]	1	.000		
	Continuity Correction[b]	167.433	1	.000		

	Likelihood Ratio	179.718	1	.000		
	Fisher's Exact Test				.000	.000
	Linear-by-Linear Association	169.044	1	.000		
	N of Valid Cases	888				
2020	Pearson Chi-Square	75.824[d]	1	.000		
	Continuity Correction[b]	73.859	1	.000		
	Likelihood Ratio	87.056	1	.000		
	Fisher's Exact Test				.000	.000
	Linear-by-Linear Association	75.616	1	.000		
	N of Valid Cases	365				
2021	Pearson Chi-Square	53.514[e]	1	.000		
	Continuity Correction[b]	51.747	1	.000		
	Likelihood Ratio	63.639	1	.000		
	Fisher's Exact Test				.000	.000
	Linear-by-Linear Association	53.363	1	.000		
	N of Valid Cases	356				
2022	Pearson Chi-Square	64.627[f]	1	.000		
	Continuity Correction[b]	62.944	1	.000		
	Likelihood Ratio	69.795	1	.000		
	Fisher's Exact Test				.000	.000
	Linear-by-Linear Association	64.469	1	.000		
	N of Valid Cases	409				
2023	Pearson Chi-Square	66.634[g]	1	.000		
	Continuity Correction[b]	64.723	1	.000		
	Likelihood Ratio	77.063	1	.000		
	Fisher's Exact Test				.000	.000
	Linear-by-Linear Association	66.439	1	.000		

	N of Valid Cases	341				
Total	Pearson Chi-Square	419.545[a]	1	.000		
	Continuity Correction[b]	417.756	1	.000		
	Likelihood Ratio	460.859	1	.000		
	Fisher's Exact Test				.000	.000
	Linear-by-Linear Association	419.367	1	.000		
	N of Valid Cases	2359				

a. 0 cells (0.0%) have an expected count of less than 5. The minimum expected count is 343.34.

b. Computed only for a 2x2 table.

c. 0 cells (0.0%) have an expected count of less than 5. The minimum expected count is 154.67.

d. 0 cells (0.0%) have an expected count of less than 5. The minimum expected count is 48.33.

e. 0 cells (0.0%) have an expected count of less than 5. The minimum expected count is 37.03.

f. 0 cells (0.0%) have an expected count of less than 5. The minimum expected count is 60.15.

g. 0 cells (0.0%) have an expected count of less than 5. The minimum expected count is 43.62.

Table S21. Sex-Based NALC Patterns.

Crosstab

YEAR_min				Non-alcoholic cirrhosis no	Non-alcoholic cirrhosis yes	Total
2019	SEX_first	FEMALE	Count	275	60	335
			% within SEX_first	82.1%	17.9%	100.0%
			% within Non-alcoholic cirrhosis	41.7%	26.2%	37.7%
			% of Total	31.0%	6.8%	37.7%
		MALE	Count	384	169	553
			% within SEX_first	69.4%	30.6%	100.0%

			% within Non-alcoholic cirrhosis	58.3%	73.8%	62.3%
			% of Total	43.2%	19.0%	62.3%
	Total		Count	659	229	888
			% within SEX_first	74.2%	25.8%	100.0%
			% within Non-alcoholic cirrhosis	100.0%	100.0%	100.0%
			% of Total	74.2%	25.8%	100.0%
2020	SEX_first	FEMALE	Count	122	25	147
			% within SEX_first	83.0%	17.0%	100.0%
			% within Non-alcoholic cirrhosis	43.7%	29.1%	40.3%
			% of Total	33.4%	6.8%	40.3%
		MALE	Count	157	61	218
			% within SEX_first	72.0%	28.0%	100.0%
			% within Non-alcoholic cirrhosis	56.3%	70.9%	59.7%
			% of Total	43.0%	16.7%	59.7%
	Total		Count	279	86	365
			% within SEX_first	76.4%	23.6%	100.0%
			% within Non-alcoholic cirrhosis	100.0%	100.0%	100.0%
			% of Total	76.4%	23.6%	100.0%
2021	SEX_first	FEMALE	Count	83	20	103
			% within SEX_first	80.6%	19.4%	100.0%
			% within Non-alcoholic cirrhosis	33.1%	19.0%	28.9%

			% of Total	23.3%	5.6%	28.9%
		MALE	Count	168	85	253
			% within SEX_first	66.4%	33.6%	100.0%
			% within Non-alcoholic cirrhosis	66.9%	81.0%	71.1%
			% of Total	47.2%	23.9%	71.1%
	Total		Count	251	105	356
			% within SEX_first	70.5%	29.5%	100.0%
			% within Non-alcoholic cirrhosis	100.0%	100.0%	100.0%
			% of Total	70.5%	29.5%	100.0%
2022	SEX_first	FEMALE	Count	109	30	139
			% within SEX_first	78.4%	21.6%	100.0%
			% within Non-alcoholic cirrhosis	36.0%	28.3%	34.0%
			% of Total	26.7%	7.3%	34.0%
		MALE	Count	194	76	270
			% within SEX_first	71.9%	28.1%	100.0%
			% within Non-alcoholic cirrhosis	64.0%	71.7%	66.0%
			% of Total	47.4%	18.6%	66.0%
	Total		Count	303	106	409
			% within SEX_first	74.1%	25.9%	100.0%
			% within Non-alcoholic cirrhosis	100.0%	100.0%	100.0%
			% of Total	74.1%	25.9%	100.0%
2023	SEX_first	FEMALE	Count	103	22	125

			% within SEX_first	82.4%	17.6%	100.0%
			% within Non-alcoholic cirrhosis	39.0%	28.6%	36.7%
			% of Total	30.2%	6.5%	36.7%
		MALE	Count	161	55	216
			% within SEX_first	74.5%	25.5%	100.0%
			% within Non-alcoholic cirrhosis	61.0%	71.4%	63.3%
			% of Total	47.2%	16.1%	63.3%
	Total		Count	264	77	341
			% within SEX_first	77.4%	22.6%	100.0%
			% within Non-alcoholic cirrhosis	100.0%	100.0%	100.0%
			% of Total	77.4%	22.6%	100.0%
Total	SEX_first	FEMALE	Count	692	157	849
			% within SEX_first	81.5%	18.5%	100.0%
			% within Non-alcoholic cirrhosis	39.4%	26.0%	36.0%
			% of Total	29.3%	6.7%	36.0%
		MALE	Count	1064	446	1510
			% within SEX_first	70.5%	29.5%	100.0%
			% within Non-alcoholic cirrhosis	60.6%	74.0%	64.0%
			% of Total	45.1%	18.9%	64.0%
	Total		Count	1756	603	2359
			% within SEX_first	74.4%	25.6%	100.0%

		% within Non-alcoholic cirrhosis	100.0%	100.0%	100.0%
		% of Total	74.4%	25.6%	100.0%

Chi-Square Tests

YEAR_min		Value	df	Asymptotic Significance (2-sided)	Exact Sig. (2-sided)	Exact Sig. (1-sided)
2019	Pearson Chi-Square	17.444[c]	1	.000		
	Continuity Correction[b]	16.790	1	.000		
	Likelihood Ratio	18.087	1	.000		
	Fisher's Exact Test				.000	.000
	Linear-by-Linear Association	17.425	1	.000		
	N of Valid Cases	888				
2020	Pearson Chi-Square	5.872[d]	1	.015		
	Continuity Correction[b]	5.278	1	.022		
	Likelihood Ratio	6.047	1	.014		
	Fisher's Exact Test				.017	.010
	Linear-by-Linear Association	5.856	1	.016		
	N of Valid Cases	365				
2021	Pearson Chi-Square	7.077[e]	1	.008		
	Continuity Correction[b]	6.412	1	.011		
	Likelihood Ratio	7.452	1	.006		
	Fisher's Exact Test				.007	.005
	Linear-by-Linear Association	7.057	1	.008		
	N of Valid Cases	356				
2022	Pearson Chi-Square	2.060[f]	1	.151		
	Continuity Correction[b]	1.732	1	.188		

	Likelihood Ratio	2.102	1	.147		
	Fisher's Exact Test				.190	.093
	Linear-by-Linear Association	2.055	1	.152		
	N of Valid Cases	409				
2023	Pearson Chi-Square	2.800[g]	1	.094		
	Continuity Correction[b]	2.369	1	.124		
	Likelihood Ratio	2.877	1	.090		
	Fisher's Exact Test				.107	.061
	Linear-by-Linear Association	2.792	1	.095		
	N of Valid Cases	341				
Total	Pearson Chi-Square	34.836[a]	1	.000		
	Continuity Correction[b]	34.258	1	.000		
	Likelihood Ratio	36.058	1	.000		
	Fisher's Exact Test				.000	.000
	Linear-by-Linear Association	34.821	1	.000		
	N of Valid Cases	2359				

a. 0 cells (0.0%) have an expected count of less than 5. The minimum expected count is 217.02.

b. Computed only for a 2x2 table.

c. 0 cells (0.0%) have an expected count of less than 5. The minimum expected count is 86.39.

d. 0 cells (0.0%) have an expected count of less than 5. The minimum expected count is 34.64.

e. 0 cells (0.0%) have an expected count of less than 5. The minimum expected count is 30.38.

f. 0 cells (0.0%) have an expected count of less than 5. The minimum expected count is 36.02.

g. 0 cells (0.0%) have an expected count of less than 5. The minimum expected count is 28.23.

Table S22. Sex-Based ALC Patterns.

Crosstab

YEAR_min	Alcoholic cirrhosis (1=yes, 0=no)	Total

				no	yes	
2019	SEX_first	FEMALE	Count	312	23	335
			% within SEX_first	93.1%	6.9%	100.0%
			% within Alcoholic cirrhosis (1=yes, 0=no)	40.2%	20.7%	37.7%
			% of Total	35.1%	2.6%	37.7%
		MALE	Count	465	88	553
			% within SEX_first	84.1%	15.9%	100.0%
			% within Alcoholic cirrhosis (1=yes, 0=no)	59.8%	79.3%	62.3%
			% of Total	52.4%	9.9%	62.3%
	Total		Count	777	111	888
			% within SEX_first	87.5%	12.5%	100.0%
			% within Alcoholic cirrhosis (1=yes, 0=no)	100.0%	100.0%	100.0%
			% of Total	87.5%	12.5%	100.0%
2020	SEX_first	FEMALE	Count	139	8	147
			% within SEX_first	94.6%	5.4%	100.0%
			% within Alcoholic cirrhosis (1=yes, 0=no)	42.4%	21.6%	40.3%
			% of Total	38.1%	2.2%	40.3%
		MALE	Count	189	29	218
			% within SEX_first	86.7%	13.3%	100.0%
			% within Alcoholic cirrhosis (1=yes, 0=no)	57.6%	78.4%	59.7%
			% of Total	51.8%	7.9%	59.7%
	Total		Count	328	37	365

			% within SEX_first	89.9%	10.1%	100.0%
			% within Alcoholic cirrhosis (1=yes, 0=no)	100.0%	100.0%	100.0%
			% of Total	89.9%	10.1%	100.0%
2021	SEX_first	FEMALE	Count	101	2	103
			% within SEX_first	98.1%	1.9%	100.0%
			% within Alcoholic cirrhosis (1=yes, 0=no)	31.8%	5.3%	28.9%
			% of Total	28.4%	0.6%	28.9%
		MALE	Count	217	36	253
			% within SEX_first	85.8%	14.2%	100.0%
			% within Alcoholic cirrhosis (1=yes, 0=no)	68.2%	94.7%	71.1%
			% of Total	61.0%	10.1%	71.1%
	Total		Count	318	38	356
			% within SEX_first	89.3%	10.7%	100.0%
			% within Alcoholic cirrhosis (1=yes, 0=no)	100.0%	100.0%	100.0%
			% of Total	89.3%	10.7%	100.0%
2022	SEX_first	FEMALE	Count	135	4	139
			% within SEX_first	97.1%	2.9%	100.0%
			% within Alcoholic cirrhosis (1=yes, 0=no)	36.2%	11.1%	34.0%
			% of Total	33.0%	1.0%	34.0%
		MALE	Count	238	32	270
			% within SEX_first	88.1%	11.9%	100.0%

			% within Alcoholic cirrhosis (1=yes, 0=no)	63.8%	88.9%	66.0%
			% of Total	58.2%	7.8%	66.0%
	Total		Count	373	36	409
			% within SEX_first	91.2%	8.8%	100.0%
			% within Alcoholic cirrhosis (1=yes, 0=no)	100.0%	100.0%	100.0%
			% of Total	91.2%	8.8%	100.0%
2023	SEX_first	FEMALE	Count	122	3	125
			% within SEX_first	97.6%	2.4%	100.0%
			% within Alcoholic cirrhosis (1=yes, 0=no)	40.3%	7.9%	36.7%
			% of Total	35.8%	0.9%	36.7%
		MALE	Count	181	35	216
			% within SEX_first	83.8%	16.2%	100.0%
			% within Alcoholic cirrhosis (1=yes, 0=no)	59.7%	92.1%	63.3%
			% of Total	53.1%	10.3%	63.3%
	Total		Count	303	38	341
			% within SEX_first	88.9%	11.1%	100.0%
			% within Alcoholic cirrhosis (1=yes, 0=no)	100.0%	100.0%	100.0%
			% of Total	88.9%	11.1%	100.0%
Total	SEX_first	FEMALE	Count	809	40	849
			% within SEX_first	95.3%	4.7%	100.0%
			% within Alcoholic cirrhosis (1=yes, 0=no)	38.5%	15.4%	36.0%

		% of Total	34.3%	1.7%	36.0%
	MALE	Count	1290	220	1510
		% within SEX_first	85.4%	14.6%	100.0%
		% within Alcoholic cirrhosis (1=yes, 0=no)	61.5%	84.6%	64.0%
		% of Total	54.7%	9.3%	64.0%
Total		Count	2099	260	2359
		% within SEX_first	89.0%	11.0%	100.0%
		% within Alcoholic cirrhosis (1=yes, 0=no)	100.0%	100.0%	100.0%
		% of Total	89.0%	11.0%	100.0%

Chi-Square Tests

YEAR_min		Value	df	Asymptotic Significance (2-sided)	Exact Sig. (2-sided)	Exact Sig. (1-sided)
2019	Pearson Chi-Square	15.613[c]	1	.000		
	Continuity Correction[b]	14.797	1	.000		
	Likelihood Ratio	16.863	1	.000		
	Fisher's Exact Test				.000	.000
	Linear-by-Linear Association	15.596	1	.000		
	N of Valid Cases	888				
2020	Pearson Chi-Square	5.955[d]	1	.015		
	Continuity Correction[b]	5.124	1	.024		
	Likelihood Ratio	6.412	1	.011		
	Fisher's Exact Test				.020	.010
	Linear-by-Linear Association	5.939	1	.015		
	N of Valid Cases	365				

2021	Pearson Chi-Square	11.591[e]	1	.001		
	Continuity Correction[b]	10.338	1	.001		
	Likelihood Ratio	15.096	1	.000		
	Fisher's Exact Test				.000	.000
	Linear-by-Linear Association	11.559	1	.001		
	N of Valid Cases	356				
2022	Pearson Chi-Square	9.206[f]	1	.002		
	Continuity Correction[b]	8.122	1	.004		
	Likelihood Ratio	10.899	1	.001		
	Fisher's Exact Test				.002	.001
	Linear-by-Linear Association	9.184	1	.002		
	N of Valid Cases	409				
2023	Pearson Chi-Square	15.236[g]	1	.000		
	Continuity Correction[b]	13.874	1	.000		
	Likelihood Ratio	18.670	1	.000		
	Fisher's Exact Test				.000	.000
	Linear-by-Linear Association	15.192	1	.000		
	N of Valid Cases	341				
Total	Pearson Chi-Square	53.854[a]	1	.000		
	Continuity Correction[b]	52.853	1	.000		
	Likelihood Ratio	60.681	1	.000		
	Fisher's Exact Test				.000	.000
	Linear-by-Linear Association	53.831	1	.000		
	N of Valid Cases	2359				

a. 0 cells (0.0%) have an expected count of less than 5. The minimum expected count is 93.57.

b. Computed only for a 2x2 table.

c. 0 cells (0.0%) have an expected count of less than 5. The minimum expected count is 41.88.

d. 0 cells (0.0%) have an expected count of less than 5. The minimum expected count is 14.90.

e. 0 cells (0.0%) have an expected count of less than 5. The minimum expected count is 10.99.

f. 0 cells (0.0%) have an expected count of less than 5. The minimum expected count is 12.23.

g. 0 cells (0.0%) have an expected count of less than 5. The minimum expected count is 13.93.

Table S23. Distribution of Child–Pugh score by CHC.

Crosstab

			SEX_first		
			FEMALE	MALE	Total
Child–Pugh	0	Count	0	1	1
		% within Child–Pugh	0.0%	100.0%	100.0%
		% within SEX_first	0.0%	0.1%	0.0%
		% of Total	0.0%	0.0%	0.0%
	A	Count	701	893	1594
		% within Child–Pugh	44.0%	56.0%	100.0%
		% within SEX_first	82.7%	59.1%	67.6%
		% of Total	29.7%	37.9%	67.6%
	B	Count	74	331	405
		% within Child–Pugh	18.3%	81.7%	100.0%
		% within SEX_first	8.7%	21.9%	17.2%
		% of Total	3.1%	14.0%	17.2%
	C	Count	73	285	358
		% within Child–Pugh	20.4%	79.6%	100.0%
		% within SEX_first	8.6%	18.9%	15.2%
		% of Total	3.1%	12.1%	15.2%
Total		Count	848	1510	2358
		% within Child–Pugh	36.0%	64.0%	100.0%

	% within SEX_first	100.0%	100.0%	100.0%
	% of Total	36.0%	64.0%	100.0%

Chi-Square Tests

	Value	df	Asymptotic Significance (2-sided)
Pearson Chi-Square	137.756[a]	3	.000
Likelihood Ratio	146.656	3	.000
Linear-by-Linear Association	111.032	1	.000
N of Valid Cases	2358		

a. 2 cells (25.0%) have an expected count of less than 5. The minimum expected count is .36.

Crosstab

			YEAR_min					
			2019	2020	2021	2022	2023	Total
Child–Pugh	0	Count	1	0	0	0	0	1
		% within Child–Pugh	100.0%	0.0%	0.0%	0.0%	0.0%	100.0%
		% within YEAR_min	0.1%	0.0%	0.0%	0.0%	0.0%	0.0%
		% of Total	0.0%	0.0%	0.0%	0.0%	0.0%	0.0%
	A	Count	638	251	226	249	230	1594
		% within Child–Pugh	40.0%	15.7%	14.2%	15.6%	14.4%	100.0%
		% within YEAR_min	71.9%	68.8%	63.5%	60.9%	67.4%	67.6%
		% of Total	27.1%	10.6%	9.6%	10.6%	9.8%	67.6%
	B	Count	141	55	63	90	56	405
		% within Child–Pugh	34.8%	13.6%	15.6%	22.2%	13.8%	100.0%
		% within YEAR_min	15.9%	15.1%	17.7%	22.0%	16.4%	17.2%
		% of Total	6.0%	2.3%	2.7%	3.8%	2.4%	17.2%

	C	Count	107	59	67	70	55	358
		% within Child–Pugh	29.9%	16.5%	18.7%	19.6%	15.4%	100.0%
		% within YEAR_min	12.1%	16.2%	18.8%	17.1%	16.1%	15.2%
		% of Total	4.5%	2.5%	2.8%	3.0%	2.3%	15.2%
Total		Count	887	365	356	409	341	2358
		% within Child–Pugh	37.6%	15.5%	15.1%	17.3%	14.5%	100.0%
		% within YEAR_min	100.0%	100.0%	100.0%	100.0%	100.0%	100.0%
		% of Total	37.6%	15.5%	15.1%	17.3%	14.5%	100.0%

Chi-Square Tests

	Value	df	Asymptotic Significance (2-sided)
Pearson Chi-Square	25.553[a]	12	.012
Likelihood Ratio	25.684	12	.012
Linear-by-Linear Association	11.050	1	.001
N of Valid Cases	2358		

a. 5 cells (25.0%) have an expected count of less than 5. The minimum expected count is .14.

Crosstab

			Age Groups							
			≤30	31-40	41-50	51-60	61-70	71-80	≥81	Total
Child–Pugh	0	Count	1	0	0	0	0	0	0	1
		% within Child–Pugh	100.0%	0.0%	0.0%	0.0%	0.0%	0.0%	0.0%	100.0%
		% within Age Groups	10.0%	0.0%	0.0%	0.0%	0.0%	0.0%	0.0%	0.0%
		% of Total	0.0%	0.0%	0.0%	0.0%	0.0%	0.0%	0.0%	0.0%
	A	Count	4	61	208	352	494	324	151	1594
		% within Child–Pugh	0.3%	3.8%	13.0%	22.1%	31.0%	20.3%	9.5%	100.0%

		% within Age Groups	40.0%	61.6%	58.1%	61.1%	67.5%	78.5%	88.8%	67.6%
		% of Total	0.2%	2.6%	8.8%	14.9%	20.9%	13.7%	6.4%	67.6%
	B	Count	3	18	83	106	135	45	15	405
		% within Child–Pugh	0.7%	4.4%	20.5%	26.2%	33.3%	11.1%	3.7%	100.0%
		% within Age Groups	30.0%	18.2%	23.2%	18.4%	18.4%	10.9%	8.8%	17.2%
		% of Total	0.1%	0.8%	3.5%	4.5%	5.7%	1.9%	0.6%	17.2%
	C	Count	2	20	67	118	103	44	4	358
		% within Child–Pugh	0.6%	5.6%	18.7%	33.0%	28.8%	12.3%	1.1%	100.0%
		% within Age Groups	20.0%	20.2%	18.7%	20.5%	14.1%	10.7%	2.4%	15.2%
		% of Total	0.1%	0.8%	2.8%	5.0%	4.4%	1.9%	0.2%	15.2%
Total		Count	10	99	358	576	732	413	170	2358
		% within Child–Pugh	0.4%	4.2%	15.2%	24.4%	31.0%	17.5%	7.2%	100.0%
		% within Age Groups	100.0%	100.0%	100.0%	100.0%	100.0%	100.0%	100.0%	100.0%
		% of Total	0.4%	4.2%	15.2%	24.4%	31.0%	17.5%	7.2%	100.0%

Chi-Square Tests

	Value	df	Asymptotic Significance (2-sided)
Pearson Chi-Square	329.472[a]	18	.000
Likelihood Ratio	116.278	18	.000
Linear-by-Linear Association	67.716	1	.000
N of Valid Cases	2358		

a. 9 cells (32.1%) have an expected count of less than 5. The minimum expected count is .00.

Crosstab

	Chronic hepatitis C (1=yes, 0=no)		Total
	no	yes	

Child–Pugh	0	Count	1	0	1
		% within Child–Pugh	100.0%	0.0%	100.0%
		% within Chronic hepatitis C (1=yes, 0=no)	0.1%	0.0%	0.0%
		% of Total	0.0%	0.0%	0.0%
	A	Count	708	886	1594
		% within Child–Pugh	44.4%	55.6%	100.0%
		% within Chronic hepatitis C (1=yes, 0=no)	51.0%	91.4%	67.6%
		% of Total	30.0%	37.6%	67.6%
	B	Count	363	42	405
		% within Child–Pugh	89.6%	10.4%	100.0%
		% within Chronic hepatitis C (1=yes, 0=no)	26.1%	4.3%	17.2%
		% of Total	15.4%	1.8%	17.2%
	C	Count	317	41	358
		% within Child–Pugh	88.5%	11.5%	100.0%
		% within Chronic hepatitis C (1=yes, 0=no)	22.8%	4.2%	15.2%
		% of Total	13.4%	1.7%	15.2%
Total		Count	1389	969	2358
		% within Child–Pugh	58.9%	41.1%	100.0%
		% within Chronic hepatitis C (1=yes, 0=no)	100.0%	100.0%	100.0%
		% of Total	58.9%	41.1%	100.0%

Chi-Square Tests

	Value	df	Asymptotic Significance (2-sided)
Pearson Chi-Square	426.813[a]	3	.000
Likelihood Ratio	479.185	3	.000
Linear-by-Linear Association	357.489	1	.000
N of Valid Cases	2358		

a. 2 cells (25.0%) have an expected count of less than 5. The minimum expected count is .41.

Table S24. Distribution of Child–Pugh score by diagnostic ALH.

Crosstab

			SEX_first		
			FEMALE	MALE	Total
Child–Pugh	0	Count	0	1	1
		% within Child–Pugh	0.0%	100.0%	100.0%
		% within SEX_first	0.0%	0.1%	0.0%
		% of Total	0.0%	0.0%	0.0%
	A	Count	702	893	1595
		% within Child–Pugh	44.0%	56.0%	100.0%
		% within SEX_first	82.7%	59.1%	67.6%
		% of Total	29.8%	37.9%	67.6%
	B	Count	74	331	405
		% within Child–Pugh	18.3%	81.7%	100.0%
		% within SEX_first	8.7%	21.9%	17.2%
		% of Total	3.1%	14.0%	17.2%
	C	Count	73	285	358

	% within Child–Pugh	20.4%	79.6%	100.0%
	% within SEX_first	8.6%	18.9%	15.2%
	% of Total	3.1%	12.1%	15.2%
Total	Count	849	1510	2359
	% within Child–Pugh	36.0%	64.0%	100.0%
	% within SEX_first	100.0%	100.0%	100.0%
	% of Total	36.0%	64.0%	100.0%

Chi-Square Tests

	Value	df	Asymptotic Significance (2-sided)
Pearson Chi-Square	138.128[a]	3	.000
Likelihood Ratio	147.058	3	.000
Linear-by-Linear Association	111.345	1	.000
N of Valid Cases	2359		

a. 2 cells (25.0%) have an expected count of less than 5. The minimum expected count is .36.

Crosstab

			YEAR_min					
			2019	2020	2021	2022	2023	Total
Child–Pugh	0	Count	1	0	0	0	0	1
		% within Child–Pugh	100.0%	0.0%	0.0%	0.0%	0.0%	100.0%
		% within YEAR_min	0.1%	0.0%	0.0%	0.0%	0.0%	0.0%
		% of Total	0.0%	0.0%	0.0%	0.0%	0.0%	0.0%
	A	Count	639	251	226	249	230	1595
		% within Child–Pugh	40.1%	15.7%	14.2%	15.6%	14.4%	100.0%
		% within YEAR_min	72.0%	68.8%	63.5%	60.9%	67.4%	67.6%

		% of Total	27.1%	10.6%	9.6%	10.6%	9.7%	67.6%
	B	Count	141	55	63	90	56	405
		% within Child–Pugh	34.8%	13.6%	15.6%	22.2%	13.8%	100.0%
		% within YEAR_min	15.9%	15.1%	17.7%	22.0%	16.4%	17.2%
		% of Total	6.0%	2.3%	2.7%	3.8%	2.4%	17.2%
	C	Count	107	59	67	70	55	358
		% within Child–Pugh	29.9%	16.5%	18.7%	19.6%	15.4%	100.0%
		% within YEAR_min	12.0%	16.2%	18.8%	17.1%	16.1%	15.2%
		% of Total	4.5%	2.5%	2.8%	3.0%	2.3%	15.2%
Total		Count	888	365	356	409	341	2359
		% within Child–Pugh	37.6%	15.5%	15.1%	17.3%	14.5%	100.0%
		% within YEAR_min	100.0%	100.0%	100.0%	100.0%	100.0%	100.0%
		% of Total	37.6%	15.5%	15.1%	17.3%	14.5%	100.0%

Chi-Square Tests

	Value	df	Asymptotic Significance (2-sided)
Pearson Chi-Square	25.677[a]	12	.012
Likelihood Ratio	25.808	12	.011
Linear-by-Linear Association	11.139	1	.001
N of Valid Cases	2359		

a. 5 cells (25.0%) have an expected count of less than 5. The minimum expected count is .14.

Crosstab

			Age Groups							
			≤30	31-40	41-50	51-60	61-70	71-80	≥81	Total
Child–Pugh	0	Count	1	0	0	0	0	0	0	1

		% within Child–Pugh	100.0%	0.0%	0.0%	0.0%	0.0%	0.0%	0.0%	100.0%
		% within Age Groups	10.0%	0.0%	0.0%	0.0%	0.0%	0.0%	0.0%	0.0%
		% of Total	0.0%	0.0%	0.0%	0.0%	0.0%	0.0%	0.0%	0.0%
	A	Count	4	62	208	352	494	324	151	1595
		% within Child–Pugh	0.3%	3.9%	13.0%	22.1%	31.0%	20.3%	9.5%	100.0%
		% within Age Groups	40.0%	62.0%	58.1%	61.1%	67.5%	78.5%	88.8%	67.6%
		% of Total	0.2%	2.6%	8.8%	14.9%	20.9%	13.7%	6.4%	67.6%
	B	Count	3	18	83	106	135	45	15	405
		% within Child–Pugh	0.7%	4.4%	20.5%	26.2%	33.3%	11.1%	3.7%	100.0%
		% within Age Groups	30.0%	18.0%	23.2%	18.4%	18.4%	10.9%	8.8%	17.2%
		% of Total	0.1%	0.8%	3.5%	4.5%	5.7%	1.9%	0.6%	17.2%
	C	Count	2	20	67	118	103	44	4	358
		% within Child–Pugh	0.6%	5.6%	18.7%	33.0%	28.8%	12.3%	1.1%	100.0%
		% within Age Groups	20.0%	20.0%	18.7%	20.5%	14.1%	10.7%	2.4%	15.2%
		% of Total	0.1%	0.8%	2.8%	5.0%	4.4%	1.9%	0.2%	15.2%
Total		Count	10	100	358	576	732	413	170	2359
		% within Child–Pugh	0.4%	4.2%	15.2%	24.4%	31.0%	17.5%	7.2%	100.0%
		% within Age Groups	100.0%	100.0%	100.0%	100.0%	100.0%	100.0%	100.0%	100.0%
		% of Total	0.4%	4.2%	15.2%	24.4%	31.0%	17.5%	7.2%	100.0%

Chi-Square Tests

	Value	df	Asymptotic Significance (2-sided)
Pearson Chi-Square	329.401[a]	18	.000
Likelihood Ratio	116.099	18	.000
Linear-by-Linear Association	67.166	1	.000
N of Valid Cases	2359		

a. 9 cells (32.1%) have an expected count of less than 5. The minimum expected count is .00.

Crosstab

			Alcoholic hepatitis (1=yes, 0=no)		
			no	yes	Total
Child–Pugh	0	Count	0	1	1
		% within Child–Pugh	0.0%	100.0%	100.0%
		% within Alcoholic hepatitis (1=yes, 0=no)	0.0%	0.1%	0.0%
		% of Total	0.0%	0.0%	0.0%
	A	Count	1104	491	1595
		% within Child–Pugh	69.2%	30.8%	100.0%
		% within Alcoholic hepatitis (1=yes, 0=no)	78.6%	51.5%	67.6%
		% of Total	46.8%	20.8%	67.6%
	B	Count	165	240	405
		% within Child–Pugh	40.7%	59.3%	100.0%
		% within Alcoholic hepatitis (1=yes, 0=no)	11.7%	25.2%	17.2%
		% of Total	7.0%	10.2%	17.2%
	C	Count	136	222	358
		% within Child–Pugh	38.0%	62.0%	100.0%
		% within Alcoholic hepatitis (1=yes, 0=no)	9.7%	23.3%	15.2%
		% of Total	5.8%	9.4%	15.2%
Total		Count	1405	954	2359
		% within Child–Pugh	59.6%	40.4%	100.0%

	% within Alcoholic hepatitis (1=yes, 0=no)	100.0%	100.0%	100.0%
	% of Total	59.6%	40.4%	100.0%

Chi-Square Tests

	Value	df	Asymptotic Significance (2-sided)
Pearson Chi-Square	191.932[a]	3	.000
Likelihood Ratio	191.226	3	.000
Linear-by-Linear Association	168.156	1	.000
N of Valid Cases	2359		

a. 2 cells (25.0%) have an expected count of less than 5. The minimum expected count is .40.

Table S25. Distribution of Child–Pugh score by diagnostic NALC.

Crosstab

			SEX_first		
			FEMALE	MALE	Total
Child–Pugh	0	Count	0	1	1
		% within Child–Pugh	0.0%	100.0%	100.0%
		% within SEX_first	0.0%	0.1%	0.0%
		% of Total	0.0%	0.0%	0.0%
	A	Count	702	893	1595
		% within Child–Pugh	44.0%	56.0%	100.0%
		% within SEX_first	82.7%	59.1%	67.6%
		% of Total	29.8%	37.9%	67.6%

	B	Count	74	331	405
		% within Child–Pugh	18.3%	81.7%	100.0%
		% within SEX_first	8.7%	21.9%	17.2%
		% of Total	3.1%	14.0%	17.2%
	C	Count	73	285	358
		% within Child–Pugh	20.4%	79.6%	100.0%
		% within SEX_first	8.6%	18.9%	15.2%
		% of Total	3.1%	12.1%	15.2%
Total		Count	849	1510	2359
		% within Child–Pugh	36.0%	64.0%	100.0%
		% within SEX_first	100.0%	100.0%	100.0%
		% of Total	36.0%	64.0%	100.0%

Chi-Square Tests

	Value	df	Asymptotic Significance (2-sided)
Pearson Chi-Square	138.128[a]	3	.000
Likelihood Ratio	147.058	3	.000
Linear-by-Linear Association	111.345	1	.000
N of Valid Cases	2359		

a. 2 cells (25.0%) have an expected count of less than 5. The minimum expected count is .36.

Crosstab

			YEAR_min					
			2019	2020	2021	2022	2023	Total
Child–Pugh	0	Count	1	0	0	0	0	1
		% within Child–Pugh	100.0%	0.0%	0.0%	0.0%	0.0%	100.0%

		% within YEAR_min	0.1%	0.0%	0.0%	0.0%	0.0%	0.0%
		% of Total	0.0%	0.0%	0.0%	0.0%	0.0%	0.0%
	A	Count	639	251	226	249	230	1595
		% within Child–Pugh	40.1%	15.7%	14.2%	15.6%	14.4%	100.0%
		% within YEAR_min	72.0%	68.8%	63.5%	60.9%	67.4%	67.6%
		% of Total	27.1%	10.6%	9.6%	10.6%	9.7%	67.6%
	B	Count	141	55	63	90	56	405
		% within Child–Pugh	34.8%	13.6%	15.6%	22.2%	13.8%	100.0%
		% within YEAR_min	15.9%	15.1%	17.7%	22.0%	16.4%	17.2%
		% of Total	6.0%	2.3%	2.7%	3.8%	2.4%	17.2%
	C	Count	107	59	67	70	55	358
		% within Child–Pugh	29.9%	16.5%	18.7%	19.6%	15.4%	100.0%
		% within YEAR_min	12.0%	16.2%	18.8%	17.1%	16.1%	15.2%
		% of Total	4.5%	2.5%	2.8%	3.0%	2.3%	15.2%
Total		Count	888	365	356	409	341	2359
		% within Child–Pugh	37.6%	15.5%	15.1%	17.3%	14.5%	100.0%
		% within YEAR_min	100.0%	100.0%	100.0%	100.0%	100.0%	100.0%
		% of Total	37.6%	15.5%	15.1%	17.3%	14.5%	100.0%

Chi-Square Tests

	Value	df	Asymptotic Significance (2-sided)
Pearson Chi-Square	25.677[a]	12	.012
Likelihood Ratio	25.808	12	.011
Linear-by-Linear Association	11.139	1	.001
N of Valid Cases	2359		

a. 5 cells (25.0%) have an expected count of less than 5. The minimum expected count is .14.

Crosstab

			Age Groups							
			≤30	31-40	41-50	51-60	61-70	71-80	≥81	Total
Child–Pugh	0	Count	1	0	0	0	0	0	0	1
		% within Child–Pugh	100.0%	0.0%	0.0%	0.0%	0.0%	0.0%	0.0%	100.0%
		% within Age Groups	10.0%	0.0%	0.0%	0.0%	0.0%	0.0%	0.0%	0.0%
		% of Total	0.0%	0.0%	0.0%	0.0%	0.0%	0.0%	0.0%	0.0%
	A	Count	4	62	208	352	494	324	151	1595
		% within Child–Pugh	0.3%	3.9%	13.0%	22.1%	31.0%	20.3%	9.5%	100.0%
		% within Age Groups	40.0%	62.0%	58.1%	61.1%	67.5%	78.5%	88.8%	67.6%
		% of Total	0.2%	2.6%	8.8%	14.9%	20.9%	13.7%	6.4%	67.6%
	B	Count	3	18	83	106	135	45	15	405
		% within Child–Pugh	0.7%	4.4%	20.5%	26.2%	33.3%	11.1%	3.7%	100.0%
		% within Age Groups	30.0%	18.0%	23.2%	18.4%	18.4%	10.9%	8.8%	17.2%
		% of Total	0.1%	0.8%	3.5%	4.5%	5.7%	1.9%	0.6%	17.2%
	C	Count	2	20	67	118	103	44	4	358
		% within Child–Pugh	0.6%	5.6%	18.7%	33.0%	28.8%	12.3%	1.1%	100.0%
		% within Age Groups	20.0%	20.0%	18.7%	20.5%	14.1%	10.7%	2.4%	15.2%
		% of Total	0.1%	0.8%	2.8%	5.0%	4.4%	1.9%	0.2%	15.2%
Total		Count	10	100	358	576	732	413	170	2359
		% within Child–Pugh	0.4%	4.2%	15.2%	24.4%	31.0%	17.5%	7.2%	100.0%
		% within Age Groups	100.0%	100.0%	100.0%	100.0%	100.0%	100.0%	100.0%	100.0%
		% of Total	0.4%	4.2%	15.2%	24.4%	31.0%	17.5%	7.2%	100.0%

Chi-Square Tests

	Value	df	Asymptotic Significance (2-sided)
Pearson Chi-Square	329.401[a]	18	.000
Likelihood Ratio	116.099	18	.000
Linear-by-Linear Association	67.166	1	.000
N of Valid Cases	2359		

a. 9 cells (32.1%) have an expected count of less than 5. The minimum expected count is .00.

Crosstab

			Non-alcoholic cirrhosis		
			no	yes	Total
Child–Pugh	0	Count	1	0	1
		% within Child–Pugh	100.0%	0.0%	100.0%
		% within Non-alcoholic cirrhosis	0.1%	0.0%	0.0%
		% of Total	0.0%	0.0%	0.0%
	A	Count	1277	318	1595
		% within Child–Pugh	80.1%	19.9%	100.0%
		% within Non-alcoholic cirrhosis	72.7%	52.7%	67.6%
		% of Total	54.1%	13.5%	67.6%
	B	Count	252	153	405
		% within Child–Pugh	62.2%	37.8%	100.0%
		% within Non-alcoholic cirrhosis	14.4%	25.4%	17.2%
		% of Total	10.7%	6.5%	17.2%
	C	Count	226	132	358

		% within Child–Pugh	63.1%	36.9%	100.0%
		% within Non-alcoholic cirrhosis	12.9%	21.9%	15.2%
		% of Total	9.6%	5.6%	15.2%
Total		Count	1756	603	2359
		% within Child–Pugh	74.4%	25.6%	100.0%
		% within Non-alcoholic cirrhosis	100.0%	100.0%	100.0%
		% of Total	74.4%	25.6%	100.0%

Chi-Square Tests

	Value	df	Asymptotic Significance (2-sided)
Pearson Chi-Square	82.691[a]	3	.000
Likelihood Ratio	79.986	3	.000
Linear-by-Linear Association	68.709	1	.000
N of Valid Cases	2359		

a. 2 cells (25.0%) have an expected count of less than 5. The minimum expected count is .26.

Table S26. Distribution of Child–Pugh score by ALC.

Crosstab

			SEX_first		
			FEMALE	MALE	Total
Child–Pugh	0	Count	0	1	1
		% within Child–Pugh	0.0%	100.0%	100.0%
		% within SEX_first	0.0%	0.1%	0.0%
		% of Total	0.0%	0.0%	0.0%

	A	Count	702	893	1595
		% within Child–Pugh	44.0%	56.0%	100.0%
		% within SEX_first	82.7%	59.1%	67.6%
		% of Total	29.8%	37.9%	67.6%
	B	Count	74	331	405
		% within Child–Pugh	18.3%	81.7%	100.0%
		% within SEX_first	8.7%	21.9%	17.2%
		% of Total	3.1%	14.0%	17.2%
	C	Count	73	285	358
		% within Child–Pugh	20.4%	79.6%	100.0%
		% within SEX_first	8.6%	18.9%	15.2%
		% of Total	3.1%	12.1%	15.2%
Total		Count	849	1510	2359
		% within Child–Pugh	36.0%	64.0%	100.0%
		% within SEX_first	100.0%	100.0%	100.0%
		% of Total	36.0%	64.0%	100.0%

Chi-Square Tests

	Value	df	Asymptotic Significance (2-sided)
Pearson Chi-Square	138.128[a]	3	.000
Likelihood Ratio	147.058	3	.000
Linear-by-Linear Association	111.345	1	.000
N of Valid Cases	2359		

a. 2 cells (25.0%) have an expected count of less than 5. The minimum expected count is .36.

Crosstab

			YEAR_min					
			2019	2020	2021	2022	2023	Total
Child–Pugh	0	Count	1	0	0	0	0	1
		% within Child–Pugh	100.0%	0.0%	0.0%	0.0%	0.0%	100.0%
		% within YEAR_min	0.1%	0.0%	0.0%	0.0%	0.0%	0.0%
		% of Total	0.0%	0.0%	0.0%	0.0%	0.0%	0.0%
	A	Count	639	251	226	249	230	1595
		% within Child–Pugh	40.1%	15.7%	14.2%	15.6%	14.4%	100.0%
		% within YEAR_min	72.0%	68.8%	63.5%	60.9%	67.4%	67.6%
		% of Total	27.1%	10.6%	9.6%	10.6%	9.7%	67.6%
	B	Count	141	55	63	90	56	405
		% within Child–Pugh	34.8%	13.6%	15.6%	22.2%	13.8%	100.0%
		% within YEAR_min	15.9%	15.1%	17.7%	22.0%	16.4%	17.2%
		% of Total	6.0%	2.3%	2.7%	3.8%	2.4%	17.2%
	C	Count	107	59	67	70	55	358
		% within Child–Pugh	29.9%	16.5%	18.7%	19.6%	15.4%	100.0%
		% within YEAR_min	12.0%	16.2%	18.8%	17.1%	16.1%	15.2%
		% of Total	4.5%	2.5%	2.8%	3.0%	2.3%	15.2%
Total		Count	888	365	356	409	341	2359
		% within Child–Pugh	37.6%	15.5%	15.1%	17.3%	14.5%	100.0%
		% within YEAR_min	100.0%	100.0%	100.0%	100.0%	100.0%	100.0%
		% of Total	37.6%	15.5%	15.1%	17.3%	14.5%	100.0%

Chi-Square Tests

	Value	df	Asymptotic Significance (2-sided)
Pearson Chi-Square	25.677[a]	12	.012

Likelihood Ratio	25.808	12	.011
Linear-by-Linear Association	11.139	1	.001
N of Valid Cases	2359		

a. 5 cells (25.0%) have an expected count of less than 5. The minimum expected count is .14.

Crosstab

			Age Groups							
			≤30	31-40	41-50	51-60	61-70	71-80	≥81	Total
Child–Pugh	0	Count	1	0	0	0	0	0	0	1
		% within Child–Pugh	100.0%	0.0%	0.0%	0.0%	0.0%	0.0%	0.0%	100.0%
		% within Age Groups	10.0%	0.0%	0.0%	0.0%	0.0%	0.0%	0.0%	0.0%
		% of Total	0.0%	0.0%	0.0%	0.0%	0.0%	0.0%	0.0%	0.0%
	A	Count	4	62	208	352	494	324	151	1595
		% within Child–Pugh	0.3%	3.9%	13.0%	22.1%	31.0%	20.3%	9.5%	100.0%
		% within Age Groups	40.0%	62.0%	58.1%	61.1%	67.5%	78.5%	88.8%	67.6%
		% of Total	0.2%	2.6%	8.8%	14.9%	20.9%	13.7%	6.4%	67.6%
	B	Count	3	18	83	106	135	45	15	405
		% within Child–Pugh	0.7%	4.4%	20.5%	26.2%	33.3%	11.1%	3.7%	100.0%
		% within Age Groups	30.0%	18.0%	23.2%	18.4%	18.4%	10.9%	8.8%	17.2%
		% of Total	0.1%	0.8%	3.5%	4.5%	5.7%	1.9%	0.6%	17.2%
	C	Count	2	20	67	118	103	44	4	358
		% within Child–Pugh	0.6%	5.6%	18.7%	33.0%	28.8%	12.3%	1.1%	100.0%
		% within Age Groups	20.0%	20.0%	18.7%	20.5%	14.1%	10.7%	2.4%	15.2%
		% of Total	0.1%	0.8%	2.8%	5.0%	4.4%	1.9%	0.2%	15.2%
Total		Count	10	100	358	576	732	413	170	2359
		% within Child–Pugh	0.4%	4.2%	15.2%	24.4%	31.0%	17.5%	7.2%	100.0%
		% within Age Groups	100.0%	100.0%	100.0%	100.0%	100.0%	100.0%	100.0%	100.0%

	% of Total	0.4%	4.2%	15.2%	24.4%	31.0%	17.5%	7.2%	100.0%

Chi-Square Tests

	Value	df	Asymptotic Significance (2-sided)
Pearson Chi-Square	329.401[a]	18	.000
Likelihood Ratio	116.099	18	.000
Linear-by-Linear Association	67.166	1	.000
N of Valid Cases	2359		

a. 9 cells (32.1%) have an expected count of less than 5. The minimum expected count is .00.

Crosstab

			Alcoholic cirrhosis (1=yes, 0=no)		
			no	yes	Total
Child–Pugh	0	Count	1	0	1
		% within Child–Pugh	100.0%	0.0%	100.0%
		% within Alcoholic cirrhosis (1=yes, 0=no)	0.0%	0.0%	0.0%
		% of Total	0.0%	0.0%	0.0%
	A	Count	1463	132	1595
		% within Child–Pugh	91.7%	8.3%	100.0%
		% within Alcoholic cirrhosis (1=yes, 0=no)	69.7%	50.8%	67.6%
		% of Total	62.0%	5.6%	67.6%
	B	Count	341	64	405
		% within Child–Pugh	84.2%	15.8%	100.0%

		% within Alcoholic cirrhosis (1=yes, 0=no)	16.2%	24.6%	17.2%
		% of Total	14.5%	2.7%	17.2%
	C	Count	294	64	358
		% within Child–Pugh	82.1%	17.9%	100.0%
		% within Alcoholic cirrhosis (1=yes, 0=no)	14.0%	24.6%	15.2%
		% of Total	12.5%	2.7%	15.2%
Total		Count	2099	260	2359
		% within Child–Pugh	89.0%	11.0%	100.0%
		% within Alcoholic cirrhosis (1=yes, 0=no)	100.0%	100.0%	100.0%
		% of Total	89.0%	11.0%	100.0%

Chi-Square Tests

	Value	df	Asymptotic Significance (2-sided)
Pearson Chi-Square	38.981[a]	3	.000
Likelihood Ratio	36.740	3	.000
Linear-by-Linear Association	36.675	1	.000
N of Valid Cases	2359		

a. 2 cells (25.0%) have an expected count of less than 5. The minimum expected count is .11.

Table S27. Logistic regression predictors for Child–Pugh A**.**

Categorical Variables Codings

	Frequency	Parameter coding

			(1)	(2)	(3)	(4)	(5)	(6)
Age Groups	≤30	10	1.000	.000	.000	.000	.000	.000
	31-40	100	.000	1.000	.000	.000	.000	.000
	41-50	358	.000	.000	1.000	.000	.000	.000
	51-60	576	.000	.000	.000	1.000	.000	.000
	61-70	732	.000	.000	.000	.000	1.000	.000
	71-80	413	.000	.000	.000	.000	.000	1.000
	≥81	170	.000	.000	.000	.000	.000	.000
YEAR_min	2019	888	1.000	.000	.000	.000		
	2020	365	.000	1.000	.000	.000		
	2021	356	.000	.000	1.000	.000		
	2022	409	.000	.000	.000	1.000		
	2023	341	.000	.000	.000	.000		
Chronic hepatitis C (1=yes, 0=no)	no	1390	1.000					
	yes	969	.000					
Alcoholic hepatitis (1=yes, 0=no)	no	1405	1.000					
	yes	954	.000					
Non-alcoholic cirrhosis	no	1756	1.000					
	yes	603	.000					
Alcoholic cirrhosis (1=yes, 0=no)	no	2099	1.000					
	yes	260	.000					
Cerebrovascular Accident	negative	2223	1.000					
	positive	136	.000					
Heart failure	negative	1914	1.000					
	positive	445	.000					
DIABETES MELLITUS	negative	1977	1.000					
	positive	382	.000					

OBESITY_max	negative	2215	1.000					
	positive	144	.000					
Portal hypertension	negative	1077	1.000					
	positive	1282	.000					
Hepatorenal syndrome	negative	2131	1.000					
	positive	228	.000					
Hepatocellular Carcinoma	negative	2210	1.000					
	positive	149	.000					
ENCEPHALOPATHY_max	negative	2078	1.000					
	positive	281	.000					
Esophageal varices	negative	1802	1.000					
	positive	557	.000					
DIGESTIVE BLEEDING	negative	2139	1.000					
	positive	220	.000					
SEX_first	FEMALE	849	.000					
	MALE	1510	1.000					

Classification Table[a,b]

Observed			Predicted		
			ChildPugh_A		Percentage Correct
			.00	1.00	
Step 0	ChildPugh_A	.00	0	763	.0
		1.00	0	1596	100.0
	Overall Percentage				67.7

a. Constant is included in the model.

b. The cut value is .500

Variables in the Equation

	B	S.E.	Wald	df	Sig.	Exp(B)

Step 0	Constant	.738	.044	281.151	1	.000	2.092

Variables not in the Equation

			Score	df	Sig.
Step 0	Variables	SEX_first(1)	136.918	1	.000
		YEAR_min	19.540	4	.001
		YEAR_min(1)	12.692	1	.000
		YEAR_min(2)	.244	1	.622
		YEAR_min(3)	3.336	1	.068
		YEAR_min(4)	10.380	1	.001
		Days_Hospitalization_per_yers_sum	8.734	1	.003
		Chronic hepatitis C (1=yes, 0=no)(1)	424.924	1	.000
		Alcoholic hepatitis (1=yes, 0=no)(1)	189.347	1	.000
		Non-alcoholic cirrhosis(1)	82.400	1	.000
		Alcoholic cirrhosis (1=yes, 0=no)(1)	38.078	1	.000
		Cerebrovascular Accident (1)	11.538	1	.001
		Heart failure(1)	63.680	1	.000
		DIABETES MELLITUS(1)	3.024	1	.082
		OBESITY_max(1)	11.662	1	.001
		Esophageal varices(1)	100.728	1	.000
		DIGESTIVE BLEEDING(1)	15.299	1	.000
		ENCEPHALOPATHY_max(1)	62.347	1	.000

		Hepatocellular Carcinoma (1)	.334	1	.563
		Hepatorenal syndrome(1)	41.513	1	.000
		Portal hypertension(1)	385.999	1	.000
		Age Groups	85.909	6	.000
		Age Groups(1)	1.431	1	.232
		Age Groups(2)	1.526	1	.217
		Age Groups(3)	17.609	1	.000
		Age Groups(4)	14.917	1	.000
		Age Groups(5)	.014	1	.906
		Age Groups(6)	26.659	1	.000
	Overall Statistics		555.500	26	.000

Omnibus Tests of Model Coefficients

		Chi-square	df	Sig.
Step 1	Step	634.110	26	.000
	Block	634.110	26	.000
	Model	634.110	26	.000

Model Summary

Step	-2 Log likelihood	Cox & Snell R Square	Nagelkerke R Square
1	2335.573[a]	.236	.329

a. Estimation terminated at iteration number 5 because parameter estimates changed by less than .001.

Hosmer and Lemeshow Test

Step	Chi-square	df	Sig.
1	54.843	8	.000

Contingency Table for Hosmer and Lemeshow Test

	ChildPugh_A = .00	ChildPugh_A = 1.00	Total

		Observed	Expected	Observed	Expected	
Step 1	1	150	160.642	86	75.358	236
	2	150	139.039	86	96.961	236
	3	118	125.322	118	110.678	236
	4	102	113.560	134	122.440	236
	5	88	92.606	148	143.394	236
	6	81	58.725	155	177.275	236
	7	58	33.725	178	202.275	236
	8	7	16.440	229	219.560	236
	9	7	13.159	229	222.841	236
	10	2	9.781	233	225.219	235

Classification Table[a]

			Predicted		
			ChildPugh_A		Percentage
	Observed		.00	1.00	Correct
Step 1	ChildPugh_A	.00	451	312	59.1
		1.00	311	1285	80.5
	Overall Percentage				73.6

a. The cut value is .500

Variables in the Equation

		B	S.E.	Wald	df	Sig.	Exp(B)	95% C.I.for EXP(B)	
								Lower	Upper
Step 1[a]	SEX_first(1)	-.063	.138	.208	1	.649	.939	.716	1.231
	YEAR_min			7.218	4	.125			
	YEAR_min(1)	.294	.164	3.229	1	.072	1.342	.974	1.849
	YEAR_min(2)	.093	.190	.240	1	.624	1.098	.756	1.593
	YEAR_min(3)	.027	.188	.020	1	.887	1.027	.711	1.484

	YEAR_min(4)	-.056	.180	.095	1	.757	.946	.664	1.347
	Days_Hospitalization_per_yers_sum	.007	.006	1.534	1	.216	1.007	.996	1.018
	Chronic hepatitis C (1=yes, 0=no)(1)	-1.753	.218	64.834	1	.000	.173	.113	.266
	Alcoholic hepatitis (1=yes, 0=no)(1)	-.224	.169	1.740	1	.187	.800	.574	1.115
	Non-alcoholic cirrhosis(1)	-.161	.165	.954	1	.329	.851	.616	1.176
	Alcoholic cirrhosis (1=yes, 0=no)(1)	-.027	.156	.029	1	.864	.974	.717	1.322
	Cerebrovascular Accident (1)	-.280	.269	1.083	1	.298	.756	.446	1.281
	Heart failure(1)	-.312	.176	3.146	1	.076	.732	.519	1.033
	DIABETES MELLITUS(1)	.290	.155	3.506	1	.061	1.336	.987	1.810
	OBESITY_max(1)	-.101	.259	.151	1	.697	.904	.545	1.502
	Esophageal varices(1)	.393	.124	10.066	1	.002	1.481	1.162	1.888
	DIGESTIVE BLEEDING(1)	.235	.176	1.773	1	.183	1.265	.895	1.787
	ENCEPHALOPATHY_max(1)	.483	.149	10.509	1	.001	1.620	1.210	2.169
	Hepatocellular Carcinoma (1)	-.006	.213	.001	1	.977	.994	.654	1.510
	Hepatorenal syndrome(1)	.327	.160	4.202	1	.040	1.387	1.014	1.897
	Portal hypertension(1)	1.124	.133	71.805	1	.000	3.078	2.373	3.993
	Age Groups			1.493	6	.960			
	Age Groups(1)	-.144	.736	.038	1	.845	.866	.204	3.667
	Age Groups(2)	.193	.363	.284	1	.594	1.213	.596	2.470
	Age Groups(3)	.062	.308	.040	1	.841	1.064	.581	1.947
	Age Groups(4)	-.030	.297	.010	1	.919	.970	.543	1.736
	Age Groups(5)	.087	.293	.088	1	.767	1.091	.614	1.937
	Age Groups(6)	.097	.304	.101	1	.751	1.101	.607	1.999

	Constant	.802	.680	1.392	1	.238	2.229		

a. Variable(s) entered on step 1: SEX_first, YEAR_min, Days_Hospitalization_per_yers_sum, Chronic hepatitis C (1=yes, 0=no), Alcoholic hepatitis (1=yes, 0=no), Non-alcoholic cirrhosis, Alcoholic cirrhosis (1=yes, 0=no), Cerebrovascular Accident , Heart failure, DIABETES MELLITUS, OBESITY_max, Esophageal varices, DIGESTIVE BLEEDING, ENCEPHALOPATHY_max, Hepatocellular Carcinoma , Hepatorenal syndrome, Portal hypertension, Age Groups.

Step number: 1

Observed Groups and Predicted Probabilities

```
    200 +                                                                    +
        I                                                           11    I
        I                                                           11    I
F       I                                                           11    I
R   150 +                                                             11    +
E       I                                                            111   I
Q       I                                                            111   I
U       I                                                            111   I
E   100 +                                                              111   +
N       I                                                            11111   I
C       I                                                            11111   I
Y       I                          1                                 11111   I
     50 +                         11111                                11111   +
        I                    1111011 11101 11  11           1   1 1         111111   I
        I                1   111100000000100000111111111          1 11111111  1      1111111   I
        I                0110000010000000000000000000000010011111 10101010001001111111111111111  I
Predicted ---------+---------+---------+---------+---------+---------+---------+---------+---------+----------
  Prob:   0       .1       .2       .3       .4       .5       .6       .7       .8       .9       1
  Group:  00000000000000000000000000000000000000000000000000111111111111111111111111111111111111111111111111111
```

Predicted Probability is of Membership for 1.00.
The Cut Value is .50.
Symbols: 0 - .00
1 - 1.00
Each Symbol Represents 12.5 Cases.

Table S28. Logistic regression predictors for Child–Pugh B.

Categorical Variables Codings

		Frequency	Parameter coding (1)	(2)	(3)	(4)	(5)	(6)
Age Groups	≤30	10	1.000	.000	.000	.000	.000	.000
	31-40	100	.000	1.000	.000	.000	.000	.000
	41-50	358	.000	.000	1.000	.000	.000	.000
	51-60	576	.000	.000	.000	1.000	.000	.000
	61-70	732	.000	.000	.000	.000	1.000	.000
	71-80	413	.000	.000	.000	.000	.000	1.000
	≥81	170	.000	.000	.000	.000	.000	.000
YEAR_min	2019	888	1.000	.000	.000	.000		
	2020	365	.000	1.000	.000	.000		
	2021	356	.000	.000	1.000	.000		
	2022	409	.000	.000	.000	1.000		
	2023	341	.000	.000	.000	.000		
Chronic hepatitis C (1=yes, 0=no)	no	1390	1.000					
	yes	969	.000					
Alcoholic hepatitis (1=yes, 0=no)	no	1405	1.000					
	yes	954	.000					
Non-alcoholic cirrhosis	no	1756	1.000					
	yes	603	.000					
Alcoholic cirrhosis (1=yes, 0=no)	no	2099	1.000					
	yes	260	.000					
Cerebrovascular Accident	negative	2223	1.000					
	positive	136	.000					

Heart failure	negative	1914	1.000						
	positive	445	.000						
DIABETES MELLITUS	negative	1977	1.000						
	positive	382	.000						
OBESITY_max	negative	2215	1.000						
	positive	144	.000						
Portal hypertension	negative	1077	1.000						
	positive	1282	.000						
Hepatorenal syndrome	negative	2131	1.000						
	positive	228	.000						
Hepatocellular Carcinoma	negative	2210	1.000						
	positive	149	.000						
ENCEPHALOPATHY_max	negative	2078	1.000						
	positive	281	.000						
Esophageal varices	negative	1802	1.000						
	positive	557	.000						
DIGESTIVE BLEEDING	negative	2139	1.000						
	positive	220	.000						
SEX_first	FEMALE	849	.000						
	MALE	1510	1.000						

Classification Table[a,b]

Observed			Predicted: ChildPugh_B .00	Predicted: ChildPugh_B 1.00	Predicted: Percentage Correct
Step 0	ChildPugh_B	.00	1954	0	100.0
		1.00	405	0	.0
	Overall Percentage				82.8

a. Constant is included in the model.

b. The cut value is .500.

Variables in the Equation

		B	S.E.	Wald	df	Sig.	Exp(B)
Step 0	Constant	-1.574	.055	830.848	1	.000	.207

Variables not in the Equation

			Score	df	Sig.
Step 0	Variables	SEX_first(1)	66.630	1	.000
		YEAR_min	9.102	4	.059
		YEAR_min(1)	1.666	1	.197
		YEAR_min(2)	1.339	1	.247
		YEAR_min(3)	.082	1	.774
		YEAR_min(4)	8.139	1	.004
		Days_Hospitalization_per_years_sum	2.401	1	.121
		Chronic hepatitis C (1=yes, 0=no)(1)	190.473	1	.000
		Alcoholic hepatitis (1=yes, 0=no)(1)	71.888	1	.000
		Non-alcoholic cirrhosis(1)	38.348	1	.000
		Alcoholic cirrhosis (1=yes, 0=no)(1)	11.396	1	.001
		Cerebrovascular Accident (1)	5.876	1	.015
		Heart failure(1)	23.046	1	.000
		DIABETES MELLITUS(1)	.461	1	.497
		OBESITY_max(1)	7.147	1	.008
		Esophageal varices(1)	58.259	1	.000
		DIGESTIVE BLEEDING(1)	3.689	1	.055

	ENCEPHALOPATHY_max(1)	18.847	1	.000
	Hepatocellular Carcinoma (1)	.126	1	.723
	Hepatorenal syndrome(1)	.805	1	.370
	Portal hypertension(1)	150.446	1	.000
	Age Groups	31.521	6	.000
	Age Groups(1)	1.163	1	.281
	Age Groups(2)	.051	1	.822
	Age Groups(3)	10.742	1	.001
	Age Groups(4)	.817	1	.366
	Age Groups(5)	1.212	1	.271
	Age Groups(6)	13.851	1	.000
Overall Statistics		252.289	26	.000

Omnibus Tests of Model Coefficients

		Chi-square	df	Sig.
Step 1	Step	287.309	26	.000
	Block	287.309	26	.000
	Model	287.309	26	.000

Model Summary

Step	-2 Log likelihood	Cox & Snell R Square	Nagelkerke R Square
1	1876.104[a]	.115	.191

a. Estimation terminated at iteration number 6 because parameter estimates changed by less than .001.

Hosmer and Lemeshow Test

Step	Chi-square	df	Sig.

1	12.477	8	.131

Contingency Table for Hosmer and Lemeshow Test

		ChildPugh_B = .00		ChildPugh_B = 1.00		
		Observed	Expected	Observed	Expected	Total
Step 1	1	231	230.417	5	5.583	236
	2	234	228.685	2	7.315	236
	3	231	226.381	5	9.619	236
	4	215	220.280	21	15.720	236
	5	198	204.671	38	31.329	236
	6	186	189.775	50	46.225	236
	7	179	178.821	57	57.179	236
	8	165	171.343	71	64.657	236
	9	169	161.730	67	74.270	236
	10	146	141.897	89	93.103	235

Classification Table[a]

			Predicted		
			ChildPugh_B		Percentage
	Observed		.00	1.00	Correct
Step 1	ChildPugh_B	.00	1951	3	99.8
		1.00	405	0	.0
	Overall Percentage				82.7

a. The cut value is .500.

Variables in the Equation

		B	S.E.	Wald	df	Sig.	Exp(B)	95% C.I.for EXP(B)	
								Lower	Upper
Step 1[a]	SEX_first(1)	.192	.164	1.376	1	.241	1.211	.879	1.669
	YEAR_min			3.098	4	.541			

	YEAR_min(1)	-.067	.189	.125	1	.724	.935	.646	1.355
	YEAR_min(2)	-.176	.223	.628	1	.428	.838	.542	1.297
	YEAR_min(3)	-.122	.217	.314	1	.575	.885	.579	1.355
	YEAR_min(4)	.139	.204	.467	1	.494	1.149	.771	1.713
	Days_Hospitalization_per_yers_sum	-.002	.006	.089	1	.765	.998	.985	1.011
	Chronic hepatitis C (1=yes, 0=no)(1)	1.797	.273	43.286	1	.000	6.031	3.531	10.301
	Alcoholic hepatitis (1=yes, 0=no)(1)	.319	.194	2.721	1	.099	1.376	.942	2.011
	Non-alcoholic cirrhosis(1)	.163	.191	.730	1	.393	1.177	.810	1.710
	Alcoholic cirrhosis (1=yes, 0=no)(1)	.172	.177	.951	1	.329	1.188	.840	1.680
	Cerebrovascular Accident (1)	.357	.328	1.189	1	.276	1.429	.752	2.716
	Heart failure(1)	.146	.208	.497	1	.481	1.158	.771	1.739
	DIABETES MELLITUS(1)	-.302	.174	3.017	1	.082	.739	.525	1.040
	OBESITY_max(1)	.345	.326	1.124	1	.289	1.412	.746	2.673
	Esophageal varices(1)	-.484	.137	12.563	1	.000	.616	.471	.805
	DIGESTIVE BLEEDING(1)	-.028	.197	.020	1	.887	.972	.660	1.431
	ENCEPHALOPATHY_max(1)	-.184	.163	1.269	1	.260	.832	.605	1.146
	Hepatocellular Carcinoma (1)	.076	.248	.093	1	.760	1.079	.663	1.754
	Hepatorenal syndrome(1)	.328	.191	2.948	1	.086	1.388	.955	2.019
	Portal hypertension(1)	-.772	.164	22.106	1	.000	.462	.335	.638
	Age Groups			5.210	6	.517			
	Age Groups(1)	-.264	.782	.114	1	.736	.768	.166	3.554
	Age Groups(2)	-.775	.417	3.449	1	.063	.461	.203	1.044
	Age Groups(3)	-.487	.343	2.022	1	.155	.614	.314	1.202

	Age Groups(4)	-.615	.332	3.426	1	.064	.541	.282	1.037
	Age Groups(5)	-.453	.325	1.942	1	.163	.636	.336	1.202
	Age Groups(6)	-.590	.343	2.951	1	.086	.554	.283	1.087
	Constant	-3.023	.799	14.332	1	.000	.049		

a. Variable(s) entered on step 1: SEX_first, YEAR_min, Days_Hospitalization_per_yers_sum, Chronic hepatitis C (1=yes, 0=no), Alcoholic hepatitis (1=yes, 0=no), Non-alcoholic cirrhosis, Alcoholic cirrhosis (1=yes, 0=no), Cerebrovascular Accident , Heart failure, DIABETES MELLITUS, OBESITY_max, Esophageal varices, DIGESTIVE BLEEDING, ENCEPHALOPATHY_max, Hepatocellular Carcinoma , Hepatorenal syndrome, Portal hypertension, Age Groups.

Step number: 1

Observed Groups and Predicted Probabilities

```
    320 +                                                        +
        I                                                        I
        I  0                                                     I
F       I  00                                                    I
R   240 +  00                                                    +
E       I  00                                                    I
Q       I  00                                                    I
U       I  00                                                    I
E   160 +  00                                                    +
N       I  001                                                   I
C       I  000                                                   I
Y       I  0001                                                  I
     80 +  0000          11111                                   +
        I  0000        0 00000 0                                 I
        I 000000001 01000 001000000000000  11                    I
        I 000000000000000000000000000000000000 001  1            I
Predicted ---------+---------+---------+---------+---------+---------+---------+---------+---------+----------
  Prob:   0       .1       .2       .3       .4       .5       .6       .7       .8       .9       1
  Group:  000000000000000000000000000000000000000000000000001111111111111111111111111111111111111111111111111
```

Predicted Probability is of Membership for 1.00
The Cut Value is .50.

Symbols: 0 - .00
1 - 1.00
Each Symbol Represents 20 Cases.

Table S29. Logistic regression predictors for Child–Pugh C.

Categorical Variables Codings

			Parameter coding					
		Frequency	(1)	(2)	(3)	(4)	(5)	(6)
Age Groups	≤30	10	1.000	.000	.000	.000	.000	.000
	31-40	100	.000	1.000	.000	.000	.000	.000
	41-50	358	.000	.000	1.000	.000	.000	.000
	51-60	576	.000	.000	.000	1.000	.000	.000
	61-70	732	.000	.000	.000	.000	1.000	.000
	71-80	413	.000	.000	.000	.000	.000	1.000
	≥81	170	.000	.000	.000	.000	.000	.000
YEAR_min	2019	888	1.000	.000	.000	.000		
	2020	365	.000	1.000	.000	.000		
	2021	356	.000	.000	1.000	.000		
	2022	409	.000	.000	.000	1.000		
	2023	341	.000	.000	.000	.000		
Chronic hepatitis C (1=yes, 0=no)	no	1390	1.000					
	yes	969	.000					
Alcoholic hepatitis (1=yes, 0=no)	no	1405	1.000					
	yes	954	.000					
Non-alcoholic cirrhosis	no	1756	1.000					
	yes	603	.000					
Alcoholic cirrhosis (1=yes, 0=no)	no	2099	1.000					
	yes	260	.000					

Cerebrovascular Accident	negative	2223	1.000
	positive	136	.000
Heart failure	negative	1914	1.000
	positive	445	.000
DIABETES MELLITUS	negative	1977	1.000
	positive	382	.000
OBESITY_max	negative	2215	1.000
	positive	144	.000
Portal hypertension	negative	1077	1.000
	positive	1282	.000
Hepatorenal syndrome	negative	2131	1.000
	positive	228	.000
Hepatocellular Carcinoma	negative	2210	1.000
	positive	149	.000
ENCEPHALOPATHY_max	negative	2078	1.000
	positive	281	.000
Esophageal varices	negative	1802	1.000
	positive	557	.000
DIGESTIVE BLEEDING	negative	2139	1.000
	positive	220	.000
SEX_first	FEMALE	849	.000
	MALE	1510	1.000

Classification Table[a,b]

			Predicted		
			ChildPugh_C		Percentage
	Observed		.00	1.00	Correct
Step 0	ChildPugh_C	.00	2001	0	100.0

	1.00	358	0	.0
	Overall Percentage			84.8

a. Constant is included in the model.

b. The cut value is .500.

Variables in the Equation

		B	S.E.	Wald	df	Sig.	Exp(B)
Step 0	Constant	-1.721	.057	899.286	1	.000	.179

Variables not in the Equation

			Score	df	Sig.
Step 0	Variables	SEX_first(1)	44.577	1	.000
		YEAR_min	12.128	4	.016
		YEAR_min(1)	10.813	1	.001
		YEAR_min(2)	.328	1	.567
		YEAR_min(3)	4.326	1	.038
		YEAR_min(4)	1.445	1	.229
		Days_Hospitalization_per_yers_sum	4.948	1	.026
		Chronic hepatitis C (1=yes, 0=no)(1)	153.029	1	.000
		Alcoholic hepatitis (1=yes, 0=no)(1)	81.528	1	.000
		Non-alcoholic cirrhosis(1)	28.372	1	.000
		Alcoholic cirrhosis (1=yes, 0=no)(1)	20.226	1	.000
		Cerebrovascular Accident (1)	3.537	1	.060
		Heart failure(1)	28.716	1	.000
		DIABETES MELLITUS(1)	2.413	1	.120

		OBESITY_max(1)	2.698	1	.100
		Esophageal varices(1)	25.634	1	.000
		DIGESTIVE BLEEDING(1)	9.493	1	.002
		ENCEPHALOPATHY_max(1)	32.855	1	.000
		Hepatocellular Carcinoma (1)	.145	1	.704
		Hepatorenal syndrome(1)	55.613	1	.000
		Portal hypertension(1)	161.897	1	.000
		Age Groups	47.059	6	.000
		Age Groups(1)	.182	1	.670
		Age Groups(2)	1.888	1	.169
		Age Groups(3)	4.107	1	.043
		Age Groups(4)	16.693	1	.000
		Age Groups(5)	1.006	1	.316
		Age Groups(6)	7.953	1	.005
	Overall Statistics		248.294	26	.000

Omnibus Tests of Model Coefficients

		Chi-square	df	Sig.
Step 1	Step	278.433	26	.000
	Block	278.433	26	.000
	Model	278.433	26	.000

Model Summary

Step	-2 Log likelihood	Cox & Snell R Square	Nagelkerke R Square
1	1730.249[a]	.111	.194

a. Estimation terminated at iteration number 7 because parameter estimates changed by less than .001.

Hosmer and Lemeshow Test

Step	Chi-square	df	Sig.
1	27.518	8	.001

Contingency Table for Hosmer and Lemeshow Test

		ChildPugh_C = .00		ChildPugh_C = 1.00		
		Observed	Expected	Observed	Expected	Total
Step 1	1	235	232.279	1	3.721	236
	2	234	229.676	2	6.324	236
	3	233	227.385	3	8.615	236
	4	220	222.069	16	13.931	236
	5	195	211.761	41	24.239	236
	6	197	197.729	39	38.271	236
	7	189	187.362	47	48.638	236
	8	171	179.960	65	56.040	236
	9	169	168.644	67	67.356	236
	10	158	144.136	77	90.864	235

Classification Table[a]

			Predicted		
			ChildPugh_C		Percentage
	Observed		.00	1.00	Correct
Step 1	ChildPugh_C	.00	1998	3	99.9
		1.00	347	11	3.1
	Overall Percentage				85.2

a. The cut value is .500

Variables in the Equation

	B	S.E.	Wald	df	Sig.	Exp(B)	95% C.I.for EXP(B)

								Lower	Upper
Step 1[a]	SEX_first(1)	-.115	.170	.454	1	.500	.892	.639	1.244
	YEAR_min			8.304	4	.081			
	YEAR_min(1)	-.360	.199	3.271	1	.071	.698	.473	1.031
	YEAR_min(2)	.063	.222	.081	1	.776	1.065	.690	1.646
	YEAR_min(3)	.097	.218	.198	1	.657	1.102	.719	1.688
	YEAR_min(4)	-.064	.214	.090	1	.764	.938	.617	1.425
	Days_Hospitalization_per_yers_sum	-.007	.007	1.154	1	.283	.993	.979	1.006
	Chronic hepatitis C (1=yes, 0=no)(1)	1.115	.274	16.607	1	.000	3.050	1.784	5.215
	Alcoholic hepatitis (1=yes, 0=no)(1)	-.045	.199	.051	1	.822	.956	.647	1.413
	Non-alcoholic cirrhosis(1)	.042	.194	.047	1	.829	1.043	.713	1.526
	Alcoholic cirrhosis (1=yes, 0=no)(1)	-.128	.179	.509	1	.476	.880	.619	1.251
	Cerebrovascular Accident (1)	.071	.335	.046	1	.831	1.074	.558	2.069
	Heart failure(1)	.331	.230	2.072	1	.150	1.392	.887	2.185
	DIABETES MELLITUS(1)	-.076	.188	.165	1	.685	.926	.641	1.340
	OBESITY_max(1)	-.199	.313	.404	1	.525	.819	.443	1.514
	Esophageal varices(1)	-.010	.147	.005	1	.943	.990	.742	1.319
	DIGESTIVE BLEEDING(1)	-.302	.201	2.264	1	.132	.739	.499	1.096
	ENCEPHALOPATHY_max(1)	-.426	.165	6.666	1	.010	.653	.473	.903
	Hepatocellular Carcinoma (1)	-.090	.263	.117	1	.733	.914	.546	1.530
	Hepatorenal syndrome(1)	-.691	.170	16.480	1	.000	.501	.359	.699
	Portal hypertension(1)	-1.055	.183	33.325	1	.000	.348	.244	.498
	Age Groups			7.727	6	.259			

	Age Groups(1)	.804	.974	.681	1	.409	2.234	.331	15.070
	Age Groups(2)	.917	.592	2.398	1	.121	2.502	.784	7.985
	Age Groups(3)	.799	.550	2.112	1	.146	2.222	.757	6.525
	Age Groups(4)	1.063	.539	3.889	1	.049	2.895	1.006	8.326
	Age Groups(5)	.723	.538	1.804	1	.179	2.061	.717	5.920
	Age Groups(6)	.868	.547	2.515	1	.113	2.383	.815	6.967
	Constant	-1.465	.906	2.617	1	.106	.231		

a. Variable(s) entered on step 1: SEX_first, YEAR_min, Days_Hospitalization_per_yers_sum, Chronic hepatitis C (1=yes, 0=no), Alcoholic hepatitis (1=yes, 0=no), Non-alcoholic cirrhosis, Alcoholic cirrhosis (1=yes, 0=no), Cerebrovascular Accident , Heart failure, DIABETES MELLITUS, OBESITY_max, Esophageal varices, DIGESTIVE BLEEDING, ENCEPHALOPATHY_max, Hepatocellular Carcinoma , Hepatorenal syndrome, Portal hypertension, Age Groups.

Step number: 1

Observed Groups and Predicted Probabilities

```
    320 +                                                            +
        I                                                            I
        I                                                            I
F       I  0                                                         I
R   240 +  00                                                        +
E       I  00                                                        I
Q       I  00                                                        I
U       I  00                                                        I
E   160 +  00                                                        +
N       I 000                                                        I
C       I 0001                                                       I
Y       I 0000          11                                           I
     80 + 0000          011                                          +
        I 0000 0100     001 100111                                   I
        I00000000000011 1000000000000001                             I
        I0000000000000000000000000000000000000010010  1   1          I
Predicted ---------+---------+---------+---------+---------+---------+---------+---------+---------+----------
  Prob:   0       .1       .2       .3       .4       .5       .6       .7       .8       .9        1
```

Group: 00000000000000000000000000000000000000000000000000111111111111111111111111111111111111111111111111111

Predicted Probability is of Membership for 1.00.
The Cut Value is .50.
Symbols: 0 - .00
1 - 1.00
Each Symbol Represents 20 Cases.

Table S30. The effects of liver disease etiology on CVA frequency.

Descriptives

Number_Hospitalizations

	N	Mean	Std. Deviation	Std. Error	95% Confidence Interval for Mean		Minimum	Maximum
					Lower Bound	Upper Bound		
CHC	1622	3.99	5.388	.134	3.73	4.26	1	31
ALH	1295	2.86	2.722	.076	2.71	3.01	1	17
NALC	1129	4.92	4.482	.133	4.66	5.18	1	19
ALC	294	3.10	2.364	.138	2.82	3.37	1	15
Total	4340	3.83	4.392	.067	3.70	3.97	1	31

Tests of Homogeneity of Variances

		Levene Statistic	df1	df2	Sig.
Number_Hospitalizations	Based on Mean	78.020	3	4336	.000
	Based on Median	37.244	3	4336	.000
	Based on Median and with adjusted df	37.244	3	3246.272	.000
	Based on trimmed mean	55.543	3	4336	.000

ANOVA

Number_Hospitalizations

	Sum of Squares	df	Mean Square	F	Sig.
Between Groups	2767.606	3	922.535	49.424	.000
Within Groups	80934.941	4336	18.666		

Total	83702.546	4339			

Multiple Comparisons

Dependent Variable: Number_Hospitalizations

Tukey HSD

(I) DIAGNOSTIC	(J) DIAGNOSTIC	Mean Difference (I-J)	Std. Error	Sig.	95% Confidence Interval Lower Bound	Upper Bound
CHC	ALH	1.135*	.161	.000	.72	1.55
	NALC	-.927*	.167	.000	-1.36	-.50
	ALC	.898*	.274	.006	.19	1.60
ALH	CHC	-1.135*	.161	.000	-1.55	-.72
	NALC	-2.062*	.176	.000	-2.51	-1.61
	ALC	-.237	.279	.830	-.95	.48
NALC	CHC	.927*	.167	.000	.50	1.36
	ALH	2.062*	.176	.000	1.61	2.51
	ALC	1.825*	.283	.000	1.10	2.55
ALC	CHC	-.898*	.274	.006	-1.60	-.19
	ALH	.237	.279	.830	-.48	.95
	NALC	-1.825*	.283	.000	-2.55	-1.10

*. The mean difference is significant at the 0.05 level.

Number_Hospitalizations

Tukey HSD[a,b]

DIAGNOSTIC	N	Subset for alpha = 0.05 1	2	3
ALH	1295	2.86		
ALC	294	3.10		
CHC	1622		3.99	
NALC	1129			4.92

Sig.		.731	1.000	1.000

Means for groups in homogeneous subsets are displayed.

a. Uses Harmonic Mean Sample Size = 704.744.

b. The group sizes are unequal. The harmonic mean of the group sizes is used. Type I error levels are not guaranteed.

Table S31. Comorbidities per years.

Crosstab

			YEAR_min					
			2019	2020	2021	2022	2023	Total
Cerebrovascular Accident	negative	Count	827	347	341	393	315	2223
		% within Cerebrovascular Accident	37.2%	15.6%	15.3%	17.7%	14.2%	100.0%
		% within YEAR_min	93.1%	95.1%	95.8%	96.1%	92.4%	94.2%
		% of Total	35.1%	14.7%	14.5%	16.7%	13.4%	94.2%
	positive	Count	61	18	15	16	26	136
		% within Cerebrovascular Accident	44.9%	13.2%	11.0%	11.8%	19.1%	100.0%
		% within YEAR_min	6.9%	4.9%	4.2%	3.9%	7.6%	5.8%
		% of Total	2.6%	0.8%	0.6%	0.7%	1.1%	5.8%
Total		Count	888	365	356	409	341	2359
		% within Cerebrovascular Accident	37.6%	15.5%	15.1%	17.3%	14.5%	100.0%
		% within YEAR_min	100.0%	100.0%	100.0%	100.0%	100.0%	100.0%
		% of Total	37.6%	15.5%	15.1%	17.3%	14.5%	100.0%

Chi-Square Tests

	Value	df	Asymptotic Significance (2-sided)
Pearson Chi-Square	8.793[a]	4	.066
Likelihood Ratio	8.984	4	.061
Linear-by-Linear Association	.463	1	.496
N of Valid Cases	2359		

a. 0 cells (.0%) have an expected count of less than 5. The minimum expected count is 19.66.

Crosstab

			YEAR_min					Total
			2019	2020	2021	2022	2023	
Heart failure	negative	Count	695	296	301	321	301	1914
		% within Heart failure	36.3%	15.5%	15.7%	16.8%	15.7%	100.0%
		% within YEAR_min	78.3%	81.1%	84.6%	78.5%	88.3%	81.1%
		% of Total	29.5%	12.5%	12.8%	13.6%	12.8%	81.1%
	positive	Count	193	69	55	88	40	445
		% within Heart failure	43.4%	15.5%	12.4%	19.8%	9.0%	100.0%
		% within YEAR_min	21.7%	18.9%	15.4%	21.5%	11.7%	18.9%
		% of Total	8.2%	2.9%	2.3%	3.7%	1.7%	18.9%
Total		Count	888	365	356	409	341	2359
		% within Heart failure	37.6%	15.5%	15.1%	17.3%	14.5%	100.0%
		% within YEAR_min	100.0%	100.0%	100.0%	100.0%	100.0%	100.0%
		% of Total	37.6%	15.5%	15.1%	17.3%	14.5%	100.0%

Chi-Square Tests

	Value	df	Asymptotic Significance (2-sided)
Pearson Chi-Square	20.710[a]	4	.000
Likelihood Ratio	22.000	4	.000
Linear-by-Linear Association	9.889	1	.002
N of Valid Cases	2359		

a. 0 cells (.0%) have an expected count of less than 5. The minimum expected count is 64.33.

Crosstab

			YEAR_min					Total
			2019	2020	2021	2022	2023	
DIABETES MELLITUS	negative	Count	735	298	302	351	291	1977
		% within DIABETES MELLITUS	37.2%	15.1%	15.3%	17.8%	14.7%	100.0%
		% within YEAR_min	82.8%	81.6%	84.8%	85.8%	85.3%	83.8%
		% of Total	31.2%	12.6%	12.8%	14.9%	12.3%	83.8%
	positive	Count	153	67	54	58	50	382
		% within DIABETES MELLITUS	40.1%	17.5%	14.1%	15.2%	13.1%	100.0%
		% within YEAR_min	17.2%	18.4%	15.2%	14.2%	14.7%	16.2%
		% of Total	6.5%	2.8%	2.3%	2.5%	2.1%	16.2%
Total		Count	888	365	356	409	341	2359
		% within DIABETES MELLITUS	37.6%	15.5%	15.1%	17.3%	14.5%	100.0%
		% within YEAR_min	100.0%	100.0%	100.0%	100.0%	100.0%	100.0%
		% of Total	37.6%	15.5%	15.1%	17.3%	14.5%	100.0%

Chi-Square Tests

	Value	df	Asymptotic Significance (2-sided)
Pearson Chi-Square	4.046[a]	4	.400
Likelihood Ratio	4.056	4	.398
Linear-by-Linear Association	2.852	1	.091
N of Valid Cases	2359		

a. 0 cells (.0%) have an expected count of less than 5. The minimum expected count is 55.22.

Crosstab

			YEAR_min					
			2019	2020	2021	2022	2023	Total
OBESITY_max	negative	Count	821	342	341	393	318	2215
		% within OBESITY_max	37.1%	15.4%	15.4%	17.7%	14.4%	100.0%
		% within YEAR_min	92.5%	93.7%	95.8%	96.1%	93.3%	93.9%
		% of Total	34.8%	14.5%	14.5%	16.7%	13.5%	93.9%
	positive	Count	67	23	15	16	23	144
		% within OBESITY_max	46.5%	16.0%	10.4%	11.1%	16.0%	100.0%
		% within YEAR_min	7.5%	6.3%	4.2%	3.9%	6.7%	6.1%
		% of Total	2.8%	1.0%	0.6%	0.7%	1.0%	6.1%
Total		Count	888	365	356	409	341	2359
		% within OBESITY_max	37.6%	15.5%	15.1%	17.3%	14.5%	100.0%
		% within YEAR_min	100.0%	100.0%	100.0%	100.0%	100.0%	100.0%
		% of Total	37.6%	15.5%	15.1%	17.3%	14.5%	100.0%

Chi-Square Tests

	Value	df	Asymptotic Significance (2-sided)
Pearson Chi-Square	9.135[a]	4	.058
Likelihood Ratio	9.644	4	.047
Linear-by-Linear Association	3.189	1	.074
N of Valid Cases	2359		

a. 0 cells (.0%) have an expected count of less than 5. The minimum expected count is 20.82.

Table S32. Comorbidites per age.

Case Processing Summary

	Cases					
	Valid		Missing		Total	
	N	Percent	N	Percent	N	Percent
Cerebrovascular Accident * Age Groups	2359	100.0%	0	0.0%	2359	100.0%
Heart failure * Age Groups	2359	100.0%	0	0.0%	2359	100.0%
DIABETES MELLITUS * Age Groups	2359[a]	100.0%	0	0.0%	2359	100.0%
OBESITY_max * Age Groups	2359[a]	100.0%	0	0.0%	2359	100.0%

a. Number of valid cases is different from the total count in the crosstabulation table because the cell counts have been rounded.

Crosstab

			Age Groups							
			≤30	31-40	41-50	51-60	61-70	71-80	≥81	Total
Cerebrovascular Accident	negative	Count	10	99	353	548	686	380	147	[illegible]

		% within Cerebrovascular Accident	0.4%	4.5%	15.9%	24.7%	30.9%	17.1%	6.6%	[illegible]
		% within Age Groups	100.0%	99.0%	98.6%	95.1%	93.7%	92.0%	86.5%	[illegible]
		% of Total	0.4%	4.2%	15.0%	23.2%	29.1%	16.1%	6.2%	[illegible]
	positive	Count	0	1	5	28	46	33	23	[illegible]
		% within Cerebrovascular Accident	0.0%	0.7%	3.7%	20.6%	33.8%	24.3%	16.9%	[illegible]
		% within Age Groups	0.0%	1.0%	1.4%	4.9%	6.3%	8.0%	13.5%	[illegible]
		% of Total	0.0%	0.0%	0.2%	1.2%	1.9%	1.4%	1.0%	[illegible]
Total		Count	10	100	358	576	732	413	170	[illegible]
		% within Cerebrovascular Accident	0.4%	4.2%	15.2%	24.4%	31.0%	17.5%	7.2%	[illegible]
		% within Age Groups	100.0%	100.0%	100.0%	100.0%	100.0%	100.0%	100.0%	[illegible]
		% of Total	0.4%	4.2%	15.2%	24.4%	31.0%	17.5%	7.2%	[illegible]

Chi-Square Tests

	Value	df	Asymptotic Significance (2-sided)
Pearson Chi-Square	41.224[a]	6	.000
Likelihood Ratio	43.873	6	.000
Linear-by-Linear Association	37.741	1	.000
N of Valid Cases	2359		

a. 1 cells (7.1%) have an expected count of less than 5. The minimum expected count is .58.

Crosstab

	Age Groups	Total

			≤30	31-40	41-50	51-60	61-70	71-80	≥81	
Heart failure	negative	Count	10	98	344	498	593	290	81	1914
		% within Heart failure	0.5%	5.1%	18.0%	26.0%	31.0%	15.2%	4.2%	100.0%
		% within Age Groups	100.0%	98.0%	96.1%	86.5%	81.0%	70.2%	47.6%	81.1%
		% of Total	0.4%	4.2%	14.6%	21.1%	25.1%	12.3%	3.4%	81.1%
	positive	Count	0	2	14	78	139	123	89	445
		% within Heart failure	0.0%	0.4%	3.1%	17.5%	31.2%	27.6%	20.0%	100.0%
		% within Age Groups	0.0%	2.0%	3.9%	13.5%	19.0%	29.8%	52.4%	18.9%
		% of Total	0.0%	0.1%	0.6%	3.3%	5.9%	5.2%	3.8%	18.9%
Total		Count	10	100	358	576	732	413	170	2359
		% within Heart failure	0.4%	4.2%	15.2%	24.4%	31.0%	17.5%	7.2%	100.0%
		% within Age Groups	100.0%	100.0%	100.0%	100.0%	100.0%	100.0%	100.0%	100.0%
		% of Total	0.4%	4.2%	15.2%	24.4%	31.0%	17.5%	7.2%	100.0%

Chi-Square Tests

	Value	df	Asymptotic Significance (2-sided)
Pearson Chi-Square	240.610[a]	6	.000
Likelihood Ratio	240.085	6	.000
Linear-by-Linear Association	214.476	1	.000
N of Valid Cases	2359		

a. 1 cells (7.1%) have an expected count of less than 5. The minimum expected count is 1.89.

Crosstab

		Age Groups							Total
		≤30	31-40	41-50	1-60	61-70	71-80	≥81	
DIABETES MELLITUS	Count	9	97	321	507	598	308	137	1977

	negative	% within DIABETES MELLITUS	0.5%	4.9%	16.2%	25.6%	30.2%	15.6%	6.9%	100.0%
		% within Age Groups	90.0%	97.0%	89.7%	88.0%	81.7%	74.6%	80.6%	83.8%
		% of Total	0.4%	4.1%	13.6%	21.5%	25.3%	13.1%	5.8%	83.8%
	positive	Count	1	3	37	69	134	105	33	382
		% within DIABETES MELLITUS	0.3%	0.8%	9.7%	18.1%	35.1%	27.5%	8.6%	100.0%
		% within Age Groups	10.0%	3.0%	10.3%	12.0%	18.3%	25.4%	19.4%	16.2%
		% of Total	0.0%	0.1%	1.6%	2.9%	5.7%	4.5%	1.4%	16.2%
Total		Count	10	100	358	576	732	413	170	2359
		% within DIABETES MELLITUS	0.4%	4.2%	15.2%	24.4%	31.0%	17.5%	7.2%	100.0%
		% within Age Groups	100.0%	100.0%	100.0%	100.0%	100.0%	100.0%	100.0%	100.0%
		% of Total	0.4%	4.2%	15.2%	24.4%	31.0%	17.5%	7.2%	100.0%

Chi-Square Tests

	Value	df	Asymptotic Significance (2-sided)
Pearson Chi-Square	59.333[a]	6	.000
Likelihood Ratio	63.274	6	.000
Linear-by-Linear Association	47.565	1	.000
N of Valid Cases	2359		

a. 1 cells (7.1%) have an expected count of less than 5. The minimum expected count is 1.62.

Crosstab

			Age Groups							Total
			≤30	31-40	41-50	51-60	61-70	71-80	≥81	
OBESITY_max	negative	Count	10	98	343	544	671	392	157	2215

		% within OBESITY_max	0.5%	4.4%	15.5%	24.6%	30.3%	17.7%	7.1%	100.0%
		% within Age Groups	100.0%	98.0%	95.8%	94.4%	91.7%	94.9%	92.4%	93.9%
		% of Total	0.4%	4.2%	14.5%	23.1%	28.4%	16.6%	6.7%	93.9%
	positive	Count	0	2	15	32	61	21	13	144
		% within OBESITY_max	0.0%	1.4%	10.4%	22.2%	42.4%	14.6%	9.0%	100.0%
		% within Age Groups	0.0%	2.0%	4.2%	5.6%	8.3%	5.1%	7.6%	6.1%
		% of Total	0.0%	0.1%	0.6%	1.4%	2.6%	0.9%	0.6%	6.1%
Total		Count	10	100	358	576	732	413	170	2359
		% within OBESITY_max	0.4%	4.2%	15.2%	24.4%	31.0%	17.5%	7.2%	100.0%
		% within Age Groups	100.0%	100.0%	100.0%	100.0%	100.0%	100.0%	100.0%	100.0%
		% of Total	0.4%	4.2%	15.2%	24.4%	31.0%	17.5%	7.2%	100.0%

Chi-Square Tests

	Value	df	Asymptotic Significance (2-sided)
Pearson Chi-Square	13.981[a]	6	.030
Likelihood Ratio	15.230	6	.019
Linear-by-Linear Association	4.852	1	.028
N of Valid Cases	2359		

a. 1 cells (7.1%) have an expected count of less than 5. The minimum expected count is .61.

Table S33. Sex-Based Distribution of Comorbidities.

Case Processing Summary

Cases					
Valid		Missing		Total	
N	Percent	N	Percent	N	Percent

Cerebrovascular Accident * SEX_first	2359	100.0%	0	0.0%	2359	100.0%
Heart failure * SEX_first	2359	100.0%	0	0.0%	2359	100.0%
DIABETES MELLITUS * SEX_first	2359[a]	100.0%	0	0.0%	2359	100.0%
OBESITY_max * SEX_first	2359[a]	100.0%	0	0.0%	2359	100.0%

a. Number of valid cases is different from the total count in the crosstabulation table because the cell counts have been rounded.

Crosstab

			SEX_first		
			FEMALE	MALE	Total
Cerebrovascular Accident	negative	Count	778	1445	2223
		% within Cerebrovascular Accident	35.0%	65.0%	100.0%
		% within SEX_first	91.6%	95.7%	94.2%
		% of Total	33.0%	61.3%	94.2%
	positive	Count	71	65	136
		% within Cerebrovascular Accident	52.2%	47.8%	100.0%
		% within SEX_first	8.4%	4.3%	5.8%
		% of Total	3.0%	2.8%	5.8%
Total		Count	849	1510	2359
		% within Cerebrovascular Accident	36.0%	64.0%	100.0%
		% within SEX_first	100.0%	100.0%	100.0%
		% of Total	36.0%	64.0%	100.0%

Chi-Square Tests

	Value	df	Asymptotic Significance (2-sided)	Exact Sig. (2-sided)	Exact Sig. (1-sided)
Pearson Chi-Square	16.474[a]	1	.000		
Continuity Correction[b]	15.735	1	.000		
Likelihood Ratio	15.794	1	.000		
Fisher's Exact Test				.000	.000
Linear-by-Linear Association	16.467	1	.000		
N of Valid Cases	2359				

a. 0 cells (.0%) have an expected count of less than 5. The minimum expected count is 48.95.

b. Computed only for a 2x2 table.

Crosstab

			SEX_first		
			FEMALE	MALE	Total
Heart failure	negative	Count	610	1304	1914
		% within Heart failure	31.9%	68.1%	100.0%
		% within SEX_first	71.8%	86.4%	81.1%
		% of Total	25.9%	55.3%	81.1%
	positive	Count	239	206	445
		% within Heart failure	53.7%	46.3%	100.0%
		% within SEX_first	28.2%	13.6%	18.9%
		% of Total	10.1%	8.7%	18.9%
Total		Count	849	1510	2359
		% within Heart failure	36.0%	64.0%	100.0%
		% within SEX_first	100.0%	100.0%	100.0%
		% of Total	36.0%	64.0%	100.0%

Chi-Square Tests

	Value	df	Asymptotic Significance (2-sided)	Exact Sig. (2-sided)	Exact Sig. (1-sided)
Pearson Chi-Square	74.739[a]	1	.000		
Continuity Correction[b]	73.794	1	.000		
Likelihood Ratio	72.196	1	.000		
Fisher's Exact Test				.000	.000
Linear-by-Linear Association	74.708	1	.000		
N of Valid Cases	2359				

a. 0 cells (.0%) have an expected count of less than 5. The minimum expected count is 160.15.

b. Computed only for a 2x2 table.

Crosstab

			SEX_first		Total
			FEMALE	MALE	
DIABETES MELLITUS	negative	Count	679	1298	1977
		% within DIABETES MELLITUS	34.3%	65.7%	100.0%
		% within SEX_first	80.0%	86.0%	83.8%
		% of Total	28.8%	55.0%	83.8%
	positive	Count	170	212	382
		% within DIABETES MELLITUS	44.5%	55.5%	100.0%
		% within SEX_first	20.0%	14.0%	16.2%
		% of Total	7.2%	9.0%	16.2%
Total		Count	849	1510	2359
		% within DIABETES MELLITUS	36.0%	64.0%	100.0%
		% within SEX_first	100.0%	100.0%	100.0%

% of Total	36.0%	64.0%	100.0%

Chi-Square Tests

	Value	df	Asymptotic Significance (2-sided)	Exact Sig. (2-sided)	Exact Sig. (1-sided)
Pearson Chi-Square	14.338[a]	1	.000		
Continuity Correction[b]	13.901	1	.000		
Likelihood Ratio	14.017	1	.000		
Fisher's Exact Test				.000	.000
Linear-by-Linear Association	14.332	1	.000		
N of Valid Cases	2359				

a. 0 cells (.0%) have an expected count of less than 5. The minimum expected count is 137.48.

b. Computed only for a 2x2 table.

Crosstab

			SEX_first		
			FEMALE	MALE	Total
OBESITY_max	negative	Count	773	1442	2215
		% within OBESITY_max	34.9%	65.1%	100.0%
		% within SEX_first	91.0%	95.5%	93.9%
		% of Total	32.8%	61.1%	93.9%
	positive	Count	76	68	144
		% within OBESITY_max	52.8%	47.2%	100.0%
		% within SEX_first	9.0%	4.5%	6.1%
		% of Total	3.2%	2.9%	6.1%
Total		Count	849	1510	2359
		% within OBESITY_max	36.0%	64.0%	100.0%
		% within SEX_first	100.0%	100.0%	100.0%

	% of Total	36.0%	64.0%	100.0%

Chi-Square Tests

	Value	df	Asymptotic Significance (2-sided)	Exact Sig. (2-sided)	Exact Sig. (1-sided)
Pearson Chi-Square	18.762[a]	1	.000		
Continuity Correction[b]	17.994	1	.000		
Likelihood Ratio	17.976	1	.000		
Fisher's Exact Test				.000	.000
Linear-by-Linear Association	18.754	1	.000		
N of Valid Cases	2359				

a. 0 cells (.0%) have an expected count of less than 5. The minimum expected count is 51.83.

b. Computed only for a 2x2 table.

Table S34. Prevalence of Liver-Related Complications by Age Group.

Case Processing Summary

	Cases					
	Valid		Missing		Total	
	N	Percent	N	Percent	N	Percent
ASCITES_max * Age Groups	2359	100.0%	0	0.0%	2359	100.0%
Esophageal varices * Age Groups	2359	100.0%	0	0.0%	2359	100.0%
DIGESTIVE BLEEDING * Age Groups	2359[a]	100.0%	0	0.0%	2359	100.0%
ENCEPHALOPATHY_max * Age Groups	2359[a]	100.0%	0	0.0%	2359	100.0%

Hepatocellular Carcinoma * Age Groups	2359[a]	100.0%	0	0.0%	2359	100.0%
Hepatorenal syndrome * Age Groups	2359[a]	100.0%	0	0.0%	2359	100.0%
Portal hypertension * Age Groups	2359[a]	100.0%	0	0.0%	2359	100.0%

a. Number of valid cases is different from the total count in the crosstabulation table because the cell counts have been rounded.

Crosstab

			Age Groups							
			≤30	31-40	41-50	51-60	61-70	71-80	≥81	Total
ASCITES_max	negative	Count	7	74	215	353	462	320	151	1582
		% within ASCITES_max	0.4%	4.7%	13.6%	22.3%	29.2%	20.2%	9.5%	100.0%
		% within Age Groups	70.0%	74.0%	60.1%	61.3%	63.1%	77.5%	88.8%	67.1%
		% of Total	0.3%	3.1%	9.1%	15.0%	19.6%	13.6%	6.4%	67.1%
	positive	Count	3	26	143	223	270	93	19	777
		% within ASCITES_max	0.4%	3.3%	18.4%	28.7%	34.7%	12.0%	2.4%	100.0%
		% within Age Groups	30.0%	26.0%	39.9%	38.7%	36.9%	22.5%	11.2%	32.9%
		% of Total	0.1%	1.1%	6.1%	9.5%	11.4%	3.9%	0.8%	32.9%
Total		Count	10	100	358	576	732	413	170	2359
		% within ASCITES_max	0.4%	4.2%	15.2%	24.4%	31.0%	17.5%	7.2%	100.0%
		% within Age Groups	100.0%	100.0%	100.0%	100.0%	100.0%	100.0%	100.0%	100.0%
		% of Total	0.4%	4.2%	15.2%	24.4%	31.0%	17.5%	7.2%	100.0%

Chi-Square Tests

	Value	df	Asymptotic Significance (2-sided)
Pearson Chi-Square	80.787[a]	6	.000

Likelihood Ratio	89.059	6	.000
Linear-by-Linear Association	37.603	1	.000
N of Valid Cases	2359		

a. 1 cells (7.1%) have an expected count of less than 5. The minimum expected count is 3.29.

Crosstab

			Age Groups							
			≤30	31-40	41-50	51-60	61-70	71-80	≥81	Total
Esophageal varices	negative	Count	7	74	246	422	563	336	154	1802
		% within Esophageal varices	0.4%	4.1%	13.7%	23.4%	31.2%	18.6%	8.5%	100.0%
		% within Age Groups	70.0%	74.0%	68.7%	73.3%	76.9%	81.4%	90.6%	76.4%
		% of Total	0.3%	3.1%	10.4%	17.9%	23.9%	14.2%	6.5%	76.4%
	positive	Count	3	26	112	154	169	77	16	557
		% within Esophageal varices	0.5%	4.7%	20.1%	27.6%	30.3%	13.8%	2.9%	100.0%
		% within Age Groups	30.0%	26.0%	31.3%	26.7%	23.1%	18.6%	9.4%	23.6%
		% of Total	0.1%	1.1%	4.7%	6.5%	7.2%	3.3%	0.7%	23.6%
Total		Count	10	100	358	576	732	413	170	2359
		% within Esophageal varices	0.4%	4.2%	15.2%	24.4%	31.0%	17.5%	7.2%	100.0%
		% within Age Groups	100.0%	100.0%	100.0%	100.0%	100.0%	100.0%	100.0%	100.0%
		% of Total	0.4%	4.2%	15.2%	24.4%	31.0%	17.5%	7.2%	100.0%

Chi-Square Tests

	Value	df	Asymptotic Significance (2-sided)
Pearson Chi-Square	40.114[a]	6	.000
Likelihood Ratio	43.654	6	.000

Linear-by-Linear Association	33.161	1	.000
N of Valid Cases	2359		

a. 1 cells (7.1%) have an expected count of less than 5. The minimum expected count is 2.36.

Crosstab

			Age Groups							
			≤30	31-40	41-50	51-60	61-70	71-80	≥81	Total
DIGESTIVE BLEEDING	negative	Count	10	93	312	522	659	380	163	2139
		% within DIGESTIVE BLEEDING	0.5%	4.3%	14.6%	24.4%	30.8%	17.8%	7.6%	100.0%
		% within Age Groups	100.0%	93.0%	87.2%	90.6%	90.0%	92.0%	95.9%	90.7%
		% of Total	0.4%	3.9%	13.2%	22.1%	27.9%	16.1%	6.9%	90.7%
	positive	Count	0	7	46	54	73	33	7	220
		% within DIGESTIVE BLEEDING	0.0%	3.2%	20.9%	24.5%	33.2%	15.0%	3.2%	100.0%
		% within Age Groups	0.0%	7.0%	12.8%	9.4%	10.0%	8.0%	4.1%	9.3%
		% of Total	0.0%	0.3%	1.9%	2.3%	3.1%	1.4%	0.3%	9.3%
Total		Count	10	100	358	576	732	413	170	2359
		% within DIGESTIVE BLEEDING	0.4%	4.2%	15.2%	24.4%	31.0%	17.5%	7.2%	100.0%
		% within Age Groups	100.0%	100.0%	100.0%	100.0%	100.0%	100.0%	100.0%	100.0%
		% of Total	0.4%	4.2%	15.2%	24.4%	31.0%	17.5%	7.2%	100.0%

Chi-Square Tests

	Value	df	Asymptotic Significance (2-sided)
Pearson Chi-Square	13.612[a]	6	.034

Likelihood Ratio	15.437	6	.017
Linear-by-Linear Association	4.458	1	.035
N of Valid Cases	2359		

a. 1 cells (7.1%) have an expected count of less than 5. The minimum expected count is .93.

Crosstab

			Age Groups							
			≤30	31-40	41-50	51-60	61-70	71-80	≥81	Total
ENCEPHALOPATHY_max	negative	Count	10	86	313	500	647	362	160	[illegible]
		% within ENCEPHALOPATHY_max	0.5%	4.1%	15.1%	24.1%	31.1%	17.4%	7.7%	[illegible]
		% within Age Groups	100.0%	86.0%	87.4%	86.8%	88.4%	87.7%	94.1%	[illegible]
		% of Total	0.4%	3.6%	13.3%	21.2%	27.4%	15.3%	6.8%	[illegible]
	positive	Count	0	14	45	76	85	51	10	[illegible]
		% within ENCEPHALOPATHY_max	0.0%	5.0%	16.0%	27.0%	30.2%	18.1%	3.6%	[illegible]
		% within Age Groups	0.0%	14.0%	12.6%	13.2%	11.6%	12.3%	5.9%	[illegible]
		% of Total	0.0%	0.6%	1.9%	3.2%	3.6%	2.2%	0.4%	[illegible]
Total		Count	10	100	358	576	732	413	170	[illegible]
		% within ENCEPHALOPATHY_max	0.4%	4.2%	15.2%	24.4%	31.0%	17.5%	7.2%	[illegible]
		% within Age Groups	100.0%	100.0%	100.0%	100.0%	100.0%	100.0%	100.0%	[illegible]
		% of Total	0.4%	4.2%	15.2%	24.4%	31.0%	17.5%	7.2%	[illegible]

Chi-Square Tests

	Value	df	Asymptotic Significance (2-sided)

Pearson Chi-Square	8.846[a]	6	.182
Likelihood Ratio	11.167	6	.083
Linear-by-Linear Association	2.592	1	.107
N of Valid Cases	2359		

a. 1 cells (7.1%) have an expected count of less than 5. The minimum expected count is 1.19.

Crosstab

			Age Groups							
			≤30	31-40	41-50	51-60	61-70	71-80	≥81	Total
Hepatocellular Carcinoma	negative	Count	10	97	338	539	683	381	162	[illegible]
		% within Hepatocellular Carcinoma	0.5%	4.4%	15.3%	24.4%	30.9%	17.2%	7.3%	[illegible]
		% within Age Groups	100.0%	97.0%	94.4%	93.6%	93.3%	92.3%	95.3%	[illegible]
		% of Total	0.4%	4.1%	14.3%	22.8%	29.0%	16.2%	6.9%	[illegible]
	positive	Count	0	3	20	37	49	32	8	[illegible]
		% within Hepatocellular Carcinoma	0.0%	2.0%	13.4%	24.8%	32.9%	21.5%	5.4%	[illegible]
		% within Age Groups	0.0%	3.0%	5.6%	6.4%	6.7%	7.7%	4.7%	[illegible]
		% of Total	0.0%	0.1%	0.8%	1.6%	2.1%	1.4%	0.3%	[illegible]
Total		Count	10	100	358	576	732	413	170	[illegible]
		% within Hepatocellular Carcinoma	0.4%	4.2%	15.2%	24.4%	31.0%	17.5%	7.2%	[illegible]
		% within Age Groups	100.0%	100.0%	100.0%	100.0%	100.0%	100.0%	100.0%	[illegible]
		% of Total	0.4%	4.2%	15.2%	24.4%	31.0%	17.5%	7.2%	[illegible]

Chi-Square Tests

	Value	df	Asymptotic Significance (2-sided)
Pearson Chi-Square	5.219[a]	6	.516
Likelihood Ratio	6.259	6	.395
Linear-by-Linear Association	1.353	1	.245
N of Valid Cases	2359		

a. 1 cells (7.1%) have an expected count of less than 5. The minimum expected count is .63.

Crosstab

			Age Groups							
			≤30	31-40	41-50	51-60	61-70	71-80	≥81	Total
Hepatorenal syndrome	negative	Count	9	88	327	506	658	377	166	2131
		% within Hepatorenal syndrome	0.4%	4.1%	15.3%	23.7%	30.9%	17.7%	7.8%	100.0%
		% within Age Groups	90.0%	88.0%	91.3%	87.8%	89.9%	91.3%	97.6%	90.3%
		% of Total	0.4%	3.7%	13.9%	21.4%	27.9%	16.0%	7.0%	90.3%
	positive	Count	1	12	31	70	74	36	4	228
		% within Hepatorenal syndrome	0.4%	5.3%	13.6%	30.7%	32.5%	15.8%	1.8%	100.0%
		% within Age Groups	10.0%	12.0%	8.7%	12.2%	10.1%	8.7%	2.4%	9.7%
		% of Total	0.0%	0.5%	1.3%	3.0%	3.1%	1.5%	0.2%	9.7%
Total		Count	10	100	358	576	732	413	170	2359
		% within Hepatorenal syndrome	0.4%	4.2%	15.2%	24.4%	31.0%	17.5%	7.2%	100.0%
		% within Age Groups	100.0%	100.0%	100.0%	100.0%	100.0%	100.0%	100.0%	100.0%
		% of Total	0.4%	4.2%	15.2%	24.4%	31.0%	17.5%	7.2%	100.0%

Chi-Square Tests

	Value	df	Asymptotic Significance (2-sided)
Pearson Chi-Square	16.125[a]	6	
Likelihood Ratio	19.960	6	
Linear-by-Linear Association	5.533	1	
N of Valid Cases	2359		

a. 1 cells (7.1%) have an expected count of less than 5. The minimum expected count is .97.

Crosstab

			Age Groups							
			≤30	31-40	41-50	51-60	61-70	71-80	≥81	Total
Portal hypertension	negative	Count	2	31	100	215	329	260	140	1077
		% within Portal hypertension	0.2%	2.9%	9.3%	20.0%	30.5%	24.1%	13.0%	100.0%
		% within Age Groups	20.0%	31.0%	27.9%	37.3%	44.9%	63.0%	82.4%	45.7%
		% of Total	0.1%	1.3%	4.2%	9.1%	13.9%	11.0%	5.9%	45.7%
	positive	Count	8	69	258	361	403	153	30	1282
		% within Portal hypertension	0.6%	5.4%	20.1%	28.2%	31.4%	11.9%	2.3%	100.0%
		% within Age Groups	80.0%	69.0%	72.1%	62.7%	55.1%	37.0%	17.6%	54.3%
		% of Total	0.3%	2.9%	10.9%	15.3%	17.1%	6.5%	1.3%	54.3%
Total		Count	10	100	358	576	732	413	170	2359
		% within Portal hypertension	0.4%	4.2%	15.2%	24.4%	31.0%	17.5%	7.2%	100.0%
		% within Age Groups	100.0%	100.0%	100.0%	100.0%	100.0%	100.0%	100.0%	100.0%
		% of Total	0.4%	4.2%	15.2%	24.4%	31.0%	17.5%	7.2%	100.0%

Chi-Square Tests

	Value	df	Asymptotic Significance (2-sided)
Pearson Chi-Square	214.966[a]	6	.000

Likelihood Ratio	223.198	6	.000
Linear-by-Linear Association	190.099	1	.000
N of Valid Cases	2359		

a. 1 cells (7.1%) have an expected count of less than 5. The minimum expected count is 4.57.

Table S35. Sex-Stratified Prevalence of Major Hepatic Complications.

Crosstab

			SEX_first		
			FEMALE	MALE	Total
ASCITES_max	negative	Count	650	932	1582
		% within ASCITES_max	41.1%	58.9%	100.0%
		% within SEX_first	76.6%	61.7%	67.1%
		% of Total	27.6%	39.5%	67.1%
	positive	Count	199	578	777
		% within ASCITES_max	25.6%	74.4%	100.0%
		% within SEX_first	23.4%	38.3%	32.9%
		% of Total	8.4%	24.5%	32.9%
Total		Count	849	1510	2359
		% within ASCITES_max	36.0%	64.0%	100.0%
		% within SEX_first	100.0%	100.0%	100.0%
		% of Total	36.0%	64.0%	100.0%

Chi-Square Tests

	Value	df	Asymptotic Significance (2-sided)	Exact Sig. (2-sided)	Exact Sig. (1-sided)
Pearson Chi-Square	54.173[a]	1	.000		

Continuity Correction[b]	53.503	1	.000		
Likelihood Ratio	55.819	1	.000		
Fisher's Exact Test				.000	.000
Linear-by-Linear Association	54.150	1	.000		
N of Valid Cases	2359				

a. 0 cells (.0%) have an expected count of less than 5. The minimum expected count is 279.64.

b. Computed only for a 2x2 table.

Crosstab

			SEX_first		
			FEMALE	MALE	Total
Esophageal varices	negative	Count	693	1109	1802
		% within Esophageal varices	38.5%	61.5%	100.0%
		% within SEX_first	81.6%	73.4%	76.4%
		% of Total	29.4%	47.0%	76.4%
	positive	Count	156	401	557
		% within Esophageal varices	28.0%	72.0%	100.0%
		% within SEX_first	18.4%	26.6%	23.6%
		% of Total	6.6%	17.0%	23.6%
Total		Count	849	1510	2359
		% within Esophageal varices	36.0%	64.0%	100.0%
		% within SEX_first	100.0%	100.0%	100.0%
		% of Total	36.0%	64.0%	100.0%

Chi-Square Tests

	Value	df	Asymptotic Significance (2-sided)	Exact Sig. (2-sided)	Exact Sig. (1-sided)
Pearson Chi-Square	20.169[a]	1	.000		

Continuity Correction[b]	19.718	1	.000		
Likelihood Ratio	20.730	1	.000		
Fisher's Exact Test				.000	.000
Linear-by-Linear Association	20.161	1	.000		
N of Valid Cases	2359				

a. 0 cells (.0%) have an expected count of less than 5. The minimum expected count is 200.46.

b. Computed only for a 2x2 table.

Crosstab

			SEX_first		Total
			FEMALE	MALE	
DIGESTIVE BLEEDING	negative	Count	779	1360	2139
		% within DIGESTIVE BLEEDING	36.4%	63.6%	100.0%
		% within SEX_first	91.8%	90.1%	90.7%
		% of Total	33.0%	57.7%	90.7%
	positive	Count	70	150	220
		% within DIGESTIVE BLEEDING	31.8%	68.2%	100.0%
		% within SEX_first	8.2%	9.9%	9.3%
		% of Total	3.0%	6.4%	9.3%
Total		Count	849	1510	2359
		% within DIGESTIVE BLEEDING	36.0%	64.0%	100.0%
		% within SEX_first	100.0%	100.0%	100.0%
		% of Total	36.0%	64.0%	100.0%

Chi-Square Tests

	Value	df	Asymptotic Significance (2-sided)	Exact Sig. (2-sided)	Exact Sig. (1-sided)
Pearson Chi-Square	1.833[a]	1	.176		
Continuity Correction[b]	1.639	1	.201		
Likelihood Ratio	1.864	1	.172		
Fisher's Exact Test				.185	.100
Linear-by-Linear Association	1.832	1	.176		
N of Valid Cases	2359				

a. 0 cells (.0%) have an expected count of less than 5. The minimum expected count is 79.18.

b. Computed only for a 2x2 table.

Crosstab

			SEX_first		
			FEMALE	MALE	Total
ENCEPHALOPATHY_max	negative	Count	789	1289	2078
		% within ENCEPHALOPATHY_max	38.0%	62.0%	100.0%
		% within SEX_first	92.9%	85.4%	88.1%
		% of Total	33.4%	54.6%	88.1%
	positive	Count	60	221	281
		% within ENCEPHALOPATHY_max	21.4%	78.6%	100.0%
		% within SEX_first	7.1%	14.6%	11.9%
		% of Total	2.5%	9.4%	11.9%
Total		Count	849	1510	2359
		% within ENCEPHALOPATHY_max	36.0%	64.0%	100.0%
		% within SEX_first	100.0%	100.0%	100.0%

	% of Total	36.0%	64.0%	100.0%

Chi-Square Tests

	Value	df	Asymptotic Significance (2-sided)	Exact Sig. (2-sided)	Exact Sig. (1-sided)
Pearson Chi-Square	29.668[a]	1	.000		
Continuity Correction[b]	28.952	1	.000		
Likelihood Ratio	31.882	1	.000		
Fisher's Exact Test				.000	.000
Linear-by-Linear Association	29.656	1	.000		
N of Valid Cases	2359				

a. 0 cells (.0%) have an expected count of less than 5. The minimum expected count is 101.13.

b. Computed only for a 2x2 table.

Crosstab

			SEX_first		
			FEMALE	MALE	Total
Hepatocellular Carcinoma	negative	Count	805	1405	2210
		% within Hepatocellular Carcinoma	36.4%	63.6%	100.0%
		% within SEX_first	94.8%	93.0%	93.7%
		% of Total	34.1%	59.6%	93.7%
	positive	Count	44	105	149
		% within Hepatocellular Carcinoma	29.5%	70.5%	100.0%
		% within SEX_first	5.2%	7.0%	6.3%
		% of Total	1.9%	4.5%	6.3%
Total		Count	849	1510	2359

	% within Hepatocellular Carcinoma	36.0%	64.0%	100.0%
	% within SEX_first	100.0%	100.0%	100.0%
	% of Total	36.0%	64.0%	100.0%

Chi-Square Tests

	Value	df	Asymptotic Significance (2-sided)	Exact Sig. (2-sided)	Exact Sig. (1-sided)
Pearson Chi-Square	2.881[a]	1	.090		
Continuity Correction[b]	2.589	1	.108		
Likelihood Ratio	2.963	1	.085		
Fisher's Exact Test				.094	.052
Linear-by-Linear Association	2.880	1	.090		
N of Valid Cases	2359				

a. 0 cells (.0%) have an expected count of less than 5. The minimum expected count is 53.62.

b. Computed only for a 2x2 table.

Crosstab

			SEX_first		
			FEMALE	MALE	Total
Hepatorenal syndrome	negative	Count	798	1333	2131
		% within Hepatorenal syndrome	37.4%	62.6%	100.0%
		% within SEX_first	94.0%	88.3%	90.3%
		% of Total	33.8%	56.5%	90.3%
	positive	Count	51	177	228
		% within Hepatorenal syndrome	22.4%	77.6%	100.0%
		% within SEX_first	6.0%	11.7%	9.7%

		% of Total	2.2%	7.5%	9.7%
Total		Count	849	1510	2359
		% within Hepatorenal syndrome	36.0%	64.0%	100.0%
		% within SEX_first	100.0%	100.0%	100.0%
		% of Total	36.0%	64.0%	100.0%

Chi-Square Tests

	Value	df	Asymptotic Significance (2-sided)	Exact Sig. (2-sided)	Exact Sig. (1-sided)
Pearson Chi-Square	20.328[a]	1	.000		
Continuity Correction[b]	19.679	1	.000		
Likelihood Ratio	21.742	1	.000		
Fisher's Exact Test				.000	.000
Linear-by-Linear Association	20.319	1	.000		
N of Valid Cases	2359				

a. 0 cells (.0%) have an expected count of less than 5. The minimum expected count is 82.06.

b. Computed only for a 2x2 table.

Crosstab

			SEX_first		
			FEMALE	MALE	Total
Portal hypertension	negative	Count	576	501	1077
		% within Portal hypertension	53.5%	46.5%	100.0%
		% within SEX_first	67.8%	33.2%	45.7%
		% of Total	24.4%	21.2%	45.7%
	positive	Count	273	1009	1282

	% within Portal hypertension	21.3%	78.7%	100.0%
	% within SEX_first	32.2%	66.8%	54.3%
	% of Total	11.6%	42.8%	54.3%
Total	Count	849	1510	2359
	% within Portal hypertension	36.0%	64.0%	100.0%
	% within SEX_first	100.0%	100.0%	100.0%
	% of Total	36.0%	64.0%	100.0%

Chi-Square Tests

	Value	df	Asymptotic Significance (2-sided)	Exact Sig. (2-sided)	Exact Sig. (1-sided)
Pearson Chi-Square	263.214[a]	1	.000		
Continuity Correction[b]	261.819	1	.000		
Likelihood Ratio	267.004	1	.000		
Fisher's Exact Test				.000	.000
Linear-by-Linear Association	263.103	1	.000		
N of Valid Cases	2359				

a. 0 cells (.0%) have an expected count of less than 5. The minimum expected count is 387.61.

b. Computed only for a 2x2 table.

Table S36. K-Means Clustering of Patient Profiles Based on Complications.

Initial Cluster Centers

	Cluster				
	1	2	3	4	5
Cerebrovascular Accident	1	0	1	0	0

Heart failure	1	1	0	0	0
DIABETES MELLITUS	1	0	0	0	1
OBESITY_max	1	0	0	0	0
ASCITES_max	0	1	0	1	1
Esophageal varices	0	1	1	0	1
DIGESTIVE BLEEDING	0	1	0	0	0
ENCEPHALOPATHY_max	0	1	1	0	0
Hepatocellular Carcinoma	0	0	0	0	1
Hepatorenal syndrome	0	1	0	1	0
Portal hypertension	0	1	1	0	1

Iteration History[a]

	Change in Cluster Centers				
Iteration	1	2	3	4	5
1	1.295	1.174	1.332	1.067	1.042
2	.056	.229	.234	.128	.225
3	.119	.131	.037	.450	.449
4	.028	.073	.104	.008	.137
5	.018	.012	.003	.017	.011
6	.000	.000	.000	.000	.000

a. Convergence achieved due to no or small change in cluster centers. The maximum absolute coordinate change for any center is .000. The current iteration is 6. The minimum distance between initial centers is 2.236.

Cluster Membership

Case Number	Cluster	Distance
1	1	.626
2	4	.155
3	4	.992

4	5	1.293
5	5	.400
6	4	.155
7	5	.400
8	3	1.352
9	2	1.283
10	3	.964
11	3	.362
12	5	.400
13	1	1.321
14	3	.362
15	4	.948
16	5	1.241
17	4	.916
18	1	1.116
19	4	.155
20	1	1.060
21	4	.155
22	1	1.025
23	1	.592
24	2	.940
25	4	.916
26	1	.626
27	4	.155
28	1	1.025
29	1	.626
30	1	.922

31	1	1.060
32	4	.155
33	2	.940
34	5	.875
35	4	.155
36	4	.155
37	3	1.360
38	4	.155
39	1	.626
40	1	.922
41	1	1.118
42	4	.155
43	4	.916
44	1	.626
45	4	.155
46	1	.626
47	2	1.354
48	5	.400
49	3	1.514
50	4	.948
51	1	.626
52	1	.626
53	1	.592
54	5	1.204
55	1	.626
56	5	.400
57	3	.980

58	1	.626
59	4	.953
60	1	.626
61	1	1.045
62	1	.626
63	1	.626
64	4	.953
65	3	.362
66	3	.964
67	1	.626
68	3	.772
69	2	1.754
70	5	.400
71	1	1.116
72	4	.155
73	3	.958
74	5	1.157
75	1	1.158
76	1	.626
77	4	1.356
78	5	1.204
79	3	1.286
80	5	.400
81	1	.626
82	4	.969
83	5	.967
84	1	.626

85	3	1.009
86	1	.922
87	5	.751
88	3	1.484
89	1	1.321
90	2	1.116
91	5	1.358
92	1	.626
93	1	1.724
94	5	1.023
95	3	.362
96	1	.626
97	3	.772
98	5	1.241
99	3	.958
100	3	.362
101	1	1.040
102	3	.908
103	1	1.426
104	1	1.470
105	5	1.293
106	4	.155
107	5	1.212
108	2	1.403
109	4	.155
110	4	.155
111	3	.362

112	3	1.181
113	1	.922
114	5	1.316
115	1	1.737
116	1	.592
117	1	1.468
118	1	.592
119	1	1.134
120	4	.969
121	3	1.477
122	1	.592
123	4	1.332
124	4	.155
125	2	1.577
126	4	.155
127	2	1.386
128	2	1.317
129	3	.772
130	2	.940
131	4	.916
132	1	.626
133	2	1.336
134	5	1.204
135	5	.400
136	1	1.245
137	1	1.454
138	2	1.613

139	2	1.089
140	1	.626
141	1	1.100
142	3	1.286
143	5	.400
144	3	1.269
145	4	.916
146	4	.951
147	5	1.669
148	1	1.118
149	4	.948
150	5	.967
151	5	.751
152	5	.400
153	4	.155
154	5	1.157
155	4	.155
156	1	1.025
157	1	.626
158	4	.155
159	4	.155
160	3	1.176
161	3	1.269
162	1	.922
163	4	.155
164	3	.908
165	1	1.104

166	5	.979
167	4	.992
168	3	1.310
169	1	.922
170	1	1.045
171	3	.362
172	3	.362
173	4	.916
174	5	.400
175	1	.626
176	1	1.060
177	1	1.025
178	3	.908
179	5	1.023
180	5	.751
181	5	.400
182	1	.922
183	3	.964
184	5	.979
185	4	.953
186	3	.362
187	3	.362
188	3	.362
189	1	1.060
190	4	.155
191	3	.908
192	1	1.045

193	4	.155
194	5	.400
195	5	.400
196	5	1.529
197	1	.922
198	4	.979
199	1	.626
200	5	1.204
201	5	.875
202	3	.772
203	1	1.045
204	4	.155
205	5	.875
206	3	.362
207	1	1.025
208	3	.362
209	5	1.250
210	2	1.283
211	4	.953
212	5	.979
213	1	1.308
214	4	.155
215	4	.155
216	3	1.441
217	4	.155
218	4	.155
219	3	1.009

220	5	.875
221	4	.155
222	1	.626
223	5	1.358
224	5	1.534
225	1	1.116
226	4	.155
227	4	.953
228	2	1.388
229	5	.751
230	3	.362
231	1	.592
232	1	.922
233	3	.362
234	2	1.055
235	4	.155
236	2	1.075
237	5	.875
238	5	.967
239	3	.958
240	3	.362
241	1	.592
242	1	.626
243	4	.155
244	1	.922
245	3	.958
246	3	1.741

247	1	.592
248	3	.772
249	3	.362
250	5	1.491
251	2	1.336
252	5	.875
253	1	.626
254	5	.400
255	4	.953
256	4	.155
257	4	.155
258	2	.628
259	1	1.400
260	5	.400
261	2	1.089
262	1	1.442
263	1	1.683
264	1	.592
265	3	.362
266	4	.155
267	3	.964
268	4	.916
269	1	.592
270	5	.979
271	2	1.597
272	4	.155
273	3	.772

274	4	1.313
275	4	.155
276	4	.969
277	3	1.022
278	3	1.009
279	3	1.477
280	4	.155
281	3	1.176
282	5	.751
283	5	.979
284	1	.626
285	1	1.741
286	3	1.181
287	4	.155
288	2	1.089
289	1	1.045
290	4	.155
291	3	.908
292	4	.155
293	1	1.454
294	5	1.059
295	3	.362
296	3	.362
297	2	.940
298	3	.964
299	2	1.603
300	4	.975

301	5	.400
302	4	1.349
303	3	.908
304	3	.772
305	3	.362
306	5	.967
307	1	.626
308	1	1.118
309	2	1.863
310	1	1.258
311	2	1.089
312	5	.400
313	1	.592
314	5	.400
315	4	.948
316	5	.751
317	5	.751
318	5	.751
319	5	.875
320	5	1.285
321	4	.155
322	3	1.656
323	4	.155
324	5	.400
325	1	.626
326	5	.400
327	3	.362

328	5	1.212
329	4	.155
330	1	.922
331	5	1.316
332	3	.772
333	5	.400
334	3	1.020
335	3	.362
336	5	.979
337	2	1.089
338	4	.953
339	1	1.487
340	2	.628
341	5	.751
342	1	.626
343	4	.155
344	4	.155
345	2	1.881
346	3	.362
347	4	.155
348	3	1.317
349	3	1.136
350	3	1.020
351	3	.362
352	5	.400
353	4	.975
354	1	1.100

355	1	1.160
356	3	.362
357	5	.979
358	5	1.082
359	5	.875
360	3	1.020
361	3	.980
362	5	.400
363	1	1.045
364	5	.400
365	5	.400
366	1	.922
367	5	.967
368	5	.400
369	3	1.218
370	2	1.664
371	3	.362
372	3	.362
373	5	.751
374	5	.400
375	4	.916
376	4	.969
377	5	.751
378	2	1.168
379	4	1.334
380	5	1.082
381	4	.953

382	4	.155
383	5	.979
384	3	.362
385	3	.362
386	3	.362
387	4	.155
388	4	.155
389	1	.626
390	3	.362
391	2	1.047
392	5	1.250
393	3	1.484
394	5	.400
395	1	1.045
396	3	1.176
397	3	.362
398	1	1.116
399	3	.362
400	3	.772
401	3	.964
402	5	.751
403	3	1.368
404	3	.958
405	3	.980
406	1	.592
407	4	.155
408	5	1.316

409	4	.155
410	4	.155
411	3	.362
412	5	1.316
413	4	.951
414	4	.155
415	5	.751
416	3	.964
417	1	.592
418	1	.922
419	3	.362
420	5	.400
421	5	1.618
422	2	1.283
423	5	.875
424	1	.592
425	5	.400
426	5	.400
427	4	.916
428	2	.628
429	3	1.344
430	2	1.055
431	5	.400
432	4	.948
433	3	1.357
434	3	1.136
435	4	.155

436	4	.948
437	3	.772
438	3	.362
439	1	.626
440	3	.362
441	1	.922
442	3	.362
443	5	1.204
444	1	1.142
445	3	1.136
446	3	.362
447	1	1.395
448	4	.975
449	2	1.150
450	2	1.089
451	2	1.089
452	5	.967
453	3	1.368
454	1	1.397
455	3	.772
456	4	.155
457	4	.992
458	3	.362
459	5	1.434
460	5	.751
461	3	.362
462	3	1.445

463	1	.626
464	1	.626
465	3	1.136
466	3	1.218
467	2	1.055
468	5	1.241
469	1	.626
470	3	.980
471	2	.628
472	5	.751
473	5	.751
474	3	1.009
475	4	.155
476	2	1.854
477	3	.362
478	1	1.116
479	5	.400
480	2	1.654
481	5	.751
482	5	.875
483	5	.751
484	5	.979
485	2	1.374
486	5	.400
487	2	1.403
488	5	.751
489	5	.400

490	5	.400
491	4	.155
492	1	1.636
493	4	.155
494	3	1.022
495	4	.155
496	1	.626
497	3	.362
498	3	.362
499	3	.980
500	3	.362
501	3	.772
502	5	1.582
503	3	1.136
504	3	.362
505	5	.751
506	3	.362
507	2	.803
508	2	1.628
509	5	.400
510	2	1.089
511	5	1.241
512	3	.362
513	1	.922
514	5	1.358
515	4	.155
516	5	1.212

517	5	.751
518	4	.916
519	4	.953
520	5	.751
521	2	1.309
522	4	.953
523	5	.400
524	5	1.061
525	5	.400
526	3	.964
527	4	.155
528	4	.155
529	4	.155
530	3	.362
531	3	.908
532	5	.400
533	2	1.302
534	4	.916
535	5	.875
536	5	1.212
537	2	1.584
538	5	1.358
539	1	1.635
540	5	.751
541	5	.400
542	5	.400
543	1	1.631

544	5	.875
545	5	.400
546	3	.362
547	5	.400
548	5	.875
549	4	.155
550	2	1.406
551	5	.400
552	4	.155
553	3	.772
554	2	1.861
555	4	.916
556	2	.997
557	4	1.623
558	1	.922
559	4	.155
560	3	.772
561	5	.400
562	3	.362
563	4	1.313
564	1	.922
565	3	1.136
566	3	1.218
567	4	.155
568	4	.155
569	5	1.434
570	4	.155

571	3	.908
572	3	.772
573	5	.979
574	4	.948
575	3	.362
576	4	.951
577	4	.948
578	5	.979
579	3	.362
580	1	1.045
581	5	.400
582	3	1.286
583	5	.751
584	5	1.846
585	5	.400
586	3	.362
587	4	.155
588	3	1.136
589	5	.979
590	3	.772
591	3	.362
592	3	.958
593	3	.362
594	2	1.568
595	1	1.040
596	3	.908
597	5	1.529

598	3	.362
599	3	.362
600	2	.997
601	1	1.724
602	4	.155
603	3	.362
604	5	1.082
605	1	1.635
606	5	.875
607	4	1.332
608	3	.958
609	3	.362
610	4	.979
611	1	1.045
612	5	1.204
613	5	1.250
614	5	.400
615	3	.362
616	3	.772
617	3	.362
618	1	.626
619	4	.979
620	3	.964
621	5	.875
622	5	.979
623	3	.772
624	3	.362

625	3	.964
626	5	.400
627	3	.964
628	5	.400
629	3	.362
630	3	.362
631	3	1.511
632	3	1.310
633	5	.400
634	5	1.023
635	4	.155
636	4	.951
637	3	.362
638	3	.362
639	5	.400
640	2	1.838
641	3	.908
642	1	.592
643	5	.400
644	5	.400
645	4	.155
646	5	.400
647	4	.155
648	3	.362
649	5	.400
650	4	.155
651	3	.362

652	4	.155
653	1	.922
654	5	.875
655	5	1.293
656	4	.155
657	1	.922
658	5	.751
659	1	.592
660	5	.967
661	3	.362
662	2	1.055
663	3	1.771
664	4	1.349
665	2	1.402
666	3	1.511
667	2	1.347
668	3	1.352
669	3	1.176
670	2	1.331
671	4	.155
672	3	.772
673	3	1.705
674	3	1.374
675	4	1.313
676	2	1.347
677	5	1.358
678	3	1.286

679	5	1.157
680	2	.628
681	3	.908
682	3	.980
683	4	.992
684	2	1.089
685	5	1.082
686	1	1.045
687	3	.362
688	3	.362
689	5	1.534
690	5	.751
691	3	.362
692	3	.362
693	1	.626
694	2	1.075
695	5	.751
696	5	.400
697	4	.155
698	3	1.022
699	3	.964
700	5	1.023
701	5	1.922
702	5	.751
703	5	.751
704	4	.155
705	2	1.841

706	4	.155
707	5	1.023
708	4	.155
709	2	1.504
710	5	.400
711	5	.751
712	5	1.157
713	1	1.675
714	5	.400
715	3	.362
716	5	.400
717	5	.751
718	3	.362
719	4	.975
720	5	.400
721	5	1.617
722	5	.400
723	5	.400
724	5	1.497
725	4	1.313
726	5	.400
727	1	1.100
728	5	.400
729	5	.400
730	4	.155
731	5	.400
732	5	.400

733	5	1.212
734	1	.922
735	2	1.641
736	3	.772
737	4	.155
738	4	.155
739	3	.908
740	2	1.089
741	4	.155
742	4	.155
743	5	1.023
744	5	.400
745	1	1.104
746	3	.362
747	1	1.142
748	5	.400
749	2	1.065
750	2	1.116
751	5	.979
752	5	.751
753	3	1.009
754	3	.362
755	5	.751
756	3	.772
757	4	1.354
758	1	1.931
759	1	1.703

760	4	.155
761	3	.362
762	4	.916
763	5	1.082
764	3	.772
765	3	.362
766	5	1.947
767	3	.772
768	4	.969
769	5	.400
770	5	1.250
771	3	1.695
772	4	.155
773	1	.922
774	5	.400
775	5	.400
776	4	.916
777	5	.751
778	3	.362
779	5	.400
780	2	1.089
781	4	.155
782	3	1.009
783	3	.362
784	4	.969
785	3	.362
786	5	1.212

787	2	1.099
788	2	1.861
789	5	.875
790	2	1.414
791	2	1.137
792	3	1.181
793	4	.155
794	4	.155
795	4	.975
796	5	.400
797	4	.975
798	5	.400
799	5	.875
800	5	.400
801	3	.958
802	1	.922
803	3	.362
804	2	.803
805	2	1.075
806	5	1.212
807	5	1.285
808	5	.751
809	5	.875
810	4	.992
811	5	.751
812	3	1.592
813	5	.400

814	1	1.321
815	3	.362
816	3	1.589
817	2	1.089
818	2	.628
819	1	1.060
820	1	.626
821	3	1.484
822	3	.362
823	3	.772
824	3	.362
825	2	1.336
826	4	.916
827	5	.400
828	4	.155
829	1	1.563
830	4	.953
831	1	1.060
832	3	1.009
833	3	1.269
834	2	.997
835	5	.400
836	5	1.212
837	5	.979
838	3	1.009
839	5	.400
840	5	.400

841	1	1.134
842	3	.772
843	2	1.601
844	3	.964
845	3	.362
846	4	.155
847	5	1.241
848	5	.400
849	2	1.344
850	4	.155
851	3	.362
852	3	1.050
853	3	.362
854	4	.155
855	1	1.116
856	2	1.055
857	5	1.702
858	3	1.369
859	1	1.397
860	2	1.065
861	3	.362
862	2	1.601
863	3	1.136
864	1	1.465
865	3	1.286
866	1	.922
867	3	.362

868	5	.400
869	3	.362
870	2	1.089
871	3	.362
872	2	1.089
873	4	.951
874	3	.362
875	3	.362
876	4	.155
877	2	.628
878	3	.362
879	4	.951
880	4	.975
881	5	.751
882	4	.155
883	5	1.250
884	2	.628
885	5	1.285
886	2	.940
887	5	1.349
888	4	.969
889	3	.362
890	2	1.780
891	3	.772
892	4	.155
893	1	1.160
894	5	.751

895	5	.400
896	3	.362
897	5	.400
898	3	.362
899	3	.362
900	5	.875
901	4	.155
902	2	1.089
903	5	.400
904	2	.628
905	1	1.614
906	1	.922
907	4	.155
908	5	.751
909	3	1.456
910	5	1.842
911	3	.964
912	5	.875
913	5	.875
914	3	.772
915	2	1.309
916	1	.922
917	4	.155
918	3	.362
919	3	1.352
920	4	.155
921	4	.155

922	5	.400
923	5	.875
924	2	1.035
925	5	.400
926	3	.362
927	3	.362
928	5	.400
929	3	1.136
930	3	.772
931	4	.948
932	2	.628
933	4	.975
934	4	.916
935	5	.400
936	5	.400
937	3	.362
938	5	1.241
939	1	.626
940	4	.155
941	2	.940
942	3	.964
943	4	.155
944	1	1.060
945	3	.362
946	2	.628
947	3	.964
948	1	1.045

949	5	.400
950	4	.155
951	2	1.283
952	3	1.340
953	3	1.592
954	5	1.558
955	4	.979
956	2	1.249
957	3	1.136
958	3	.362
959	4	.155
960	3	1.368
961	3	.958
962	3	.362
963	2	1.075
964	2	1.089
965	5	1.082
966	3	.772
967	5	.400
968	4	1.383
969	3	.908
970	2	1.065
971	3	1.584
972	4	.992
973	5	.400
974	4	.951
975	5	.751

976	3	.362
977	4	.951
978	4	.155
979	4	.953
980	3	.362
981	5	.400
982	2	.628
983	4	.155
984	3	1.022
985	5	.400
986	5	.875
987	3	.362
988	4	.155
989	5	1.846
990	2	1.302
991	3	.772
992	5	.967
993	2	1.283
994	4	.951
995	2	.628
996	4	.155
997	3	.980
998	2	1.515
999	5	1.815
1000	3	.362
1001	5	.400
1002	5	.751

1003	5	.400
1004	4	.155
1005	1	1.116
1006	2	1.283
1007	3	.362
1008	5	1.082
1009	4	.155
1010	2	1.336
1011	4	.951
1012	3	1.176
1013	2	.628
1014	2	1.336
1015	2	1.302
1016	5	.979
1017	4	.155
1018	4	.155
1019	3	.362
1020	3	.958
1021	5	.875
1022	4	.155
1023	3	.362
1024	5	.967
1025	3	.362
1026	3	.362
1027	3	.362
1028	5	.400
1029	3	.362

1030	3	.362
1031	4	.155
1032	5	.400
1033	3	.362
1034	4	.155
1035	5	.400
1036	3	.362
1037	5	.400
1038	5	.400
1039	3	.362
1040	3	.772
1041	3	.362
1042	2	.628
1043	5	.400
1044	5	.400
1045	4	.155
1046	3	.362
1047	5	.400
1048	1	.922
1049	3	.362
1050	1	.922
1051	3	.362
1052	5	.400
1053	5	.400
1054	4	.155
1055	2	.997
1056	4	.155

1057	5	.979
1058	4	.155
1059	5	1.241
1060	3	1.368
1061	1	.626
1062	3	1.369
1063	3	.362
1064	3	.772
1065	2	.628
1066	4	.992
1067	5	1.293
1068	3	.964
1069	4	.155
1070	2	.628
1071	4	.155
1072	4	1.335
1073	4	.155
1074	5	.875
1075	4	.979
1076	5	.400
1077	2	1.612
1078	5	1.082
1079	2	.803
1080	3	.362
1081	4	.155
1082	1	1.160
1083	2	.628

1084	3	.362
1085	5	.400
1086	3	.362
1087	3	.362
1088	3	1.476
1089	3	.772
1090	2	1.055
1091	2	1.395
1092	3	1.635
1093	4	.155
1094	5	.400
1095	4	1.313
1096	1	1.258
1097	5	.751
1098	5	.400
1099	3	1.274
1100	3	.362
1101	3	.772
1102	5	.751
1103	5	.751
1104	3	.772
1105	5	.751
1106	4	.916
1107	2	1.577
1108	5	.400
1109	5	1.023
1110	3	.362

1111	3	.908
1112	4	.155
1113	3	.964
1114	3	.772
1115	3	.362
1116	5	.979
1117	3	.980
1118	3	.362
1119	4	.951
1120	3	1.136
1121	3	1.589
1122	5	.400
1123	3	.362
1124	1	.626
1125	3	.908
1126	5	.875
1127	3	.908
1128	2	1.047
1129	3	1.176
1130	3	.362
1131	3	.958
1132	5	.400
1133	3	.362
1134	5	.400
1135	3	.362
1136	5	.400
1137	5	.400

1138	3	.772
1139	5	.400
1140	4	.953
1141	5	.751
1142	5	1.204
1143	5	.400
1144	3	.958
1145	3	.362
1146	5	.400
1147	3	.772
1148	4	.155
1149	5	.979
1150	1	.626
1151	3	.362
1152	2	.997
1153	3	.958
1154	4	.155
1155	3	.362
1156	2	.803
1157	5	.751
1158	3	.772
1159	5	.400
1160	4	.155
1161	3	.362
1162	5	.751
1163	5	.400
1164	3	.362

1165	5	.751
1166	5	.400
1167	2	.940
1168	3	1.136
1169	3	.362
1170	4	.155
1171	3	.362
1172	3	.362
1173	1	1.623
1174	1	1.308
1175	4	.948
1176	3	.908
1177	1	.592
1178	3	1.009
1179	3	.362
1180	5	.751
1181	2	1.055
1182	1	.922
1183	1	1.040
1184	3	.362
1185	5	.979
1186	2	1.344
1187	3	1.136
1188	2	1.089
1189	2	1.089
1190	2	1.377
1191	3	.964

1192	4	.155
1193	4	.979
1194	3	.362
1195	3	.362
1196	5	1.349
1197	4	.155
1198	4	.155
1199	2	.628
1200	5	.751
1201	2	1.089
1202	4	.155
1203	3	.362
1204	5	.400
1205	3	1.136
1206	4	.155
1207	2	1.249
1208	3	.362
1209	3	.362
1210	1	.626
1211	2	.997
1212	5	.967
1213	2	1.355
1214	2	.803
1215	3	1.176
1216	5	.400
1217	2	.628
1218	3	1.360

1219	4	.155
1220	2	.628
1221	4	.155
1222	3	1.784
1223	3	.362
1224	4	.951
1225	4	1.355
1226	1	1.258
1227	4	.155
1228	3	.362
1229	5	.875
1230	4	.155
1231	2	1.283
1232	2	1.089
1233	3	.772
1234	1	1.311
1235	2	.940
1236	3	.362
1237	1	.922
1238	5	.400
1239	5	.400
1240	3	1.136
1241	3	.362
1242	5	.751
1243	4	.155
1244	3	.362
1245	5	.400

1246	3	.772
1247	5	.875
1248	5	.751
1249	2	.940
1250	4	.951
1251	4	.953
1252	5	1.235
1253	5	.751
1254	3	.362
1255	5	.400
1256	2	1.089
1257	4	.155
1258	5	.979
1259	2	1.437
1260	3	1.758
1261	4	.155
1262	4	.155
1263	3	.362
1264	4	.916
1265	5	.400
1266	3	.362
1267	3	1.340
1268	5	.400
1269	4	.155
1270	3	.964
1271	3	.362
1272	2	1.543

1273	5	.400
1274	5	.979
1275	3	.772
1276	5	1.023
1277	1	1.328
1278	2	.628
1279	3	.362
1280	4	.155
1281	5	.751
1282	4	.948
1283	2	.628
1284	3	.362
1285	3	.964
1286	4	.155
1287	5	.400
1288	5	.400
1289	3	1.009
1290	3	.362
1291	3	.964
1292	4	.155
1293	4	.155
1294	3	1.269
1295	5	.751
1296	4	.916
1297	5	1.061
1298	5	.751
1299	4	.155

1300	5	.400
1301	4	.951
1302	1	.592
1303	3	1.340
1304	3	.362
1305	3	.362
1306	5	.400
1307	3	.772
1308	3	.772
1309	2	.628
1310	4	.155
1311	3	.362
1312	3	.362
1313	3	.362
1314	5	.875
1315	3	.362
1316	5	.875
1317	3	.772
1318	3	.772
1319	3	.958
1320	2	.628
1321	3	1.009
1322	1	1.451
1323	4	.155
1324	5	1.023
1325	2	1.089
1326	3	.958

1327	3	.362
1328	4	.979
1329	3	.362
1330	5	.751
1331	3	.362
1332	2	1.338
1333	3	.362
1334	2	1.089
1335	5	.400
1336	5	.751
1337	5	.400
1338	3	1.050
1339	3	.772
1340	5	.400
1341	2	1.365
1342	4	.155
1343	2	1.065
1344	3	.362
1345	4	.916
1346	2	1.055
1347	4	.979
1348	5	1.082
1349	2	1.317
1350	5	.751
1351	3	.362
1352	3	.362
1353	2	1.055

1354	5	.875
1355	4	.155
1356	5	.400
1357	5	.400
1358	3	.362
1359	3	.908
1360	5	1.250
1361	5	.400
1362	4	.951
1363	3	.362
1364	5	.751
1365	2	.628
1366	5	.751
1367	3	1.728
1368	4	.979
1369	5	.967
1370	3	.362
1371	3	.362
1372	4	.155
1373	2	1.075
1374	3	1.009
1375	5	.875
1376	3	.908
1377	5	.967
1378	4	.155
1379	5	.400
1380	3	.362

1381	3	.362
1382	5	.400
1383	5	.751
1384	4	.951
1385	4	.155
1386	3	1.310
1387	2	1.309
1388	3	1.274
1389	3	.362
1390	1	.922
1391	5	1.212
1392	2	1.089
1393	5	.751
1394	3	.362
1395	3	.362
1396	2	.628
1397	5	.751
1398	3	.772
1399	5	1.082
1400	5	.400
1401	3	1.514
1402	4	.992
1403	5	.875
1404	3	.362
1405	4	.948
1406	3	.362
1407	5	.751

1408	5	.875
1409	2	1.089
1410	5	.751
1411	4	.155
1412	5	.400
1413	5	.400
1414	5	.751
1415	3	.362
1416	3	1.009
1417	3	.362
1418	4	.155
1419	1	.922
1420	3	.964
1421	5	.400
1422	5	1.316
1423	3	1.022
1424	4	.155
1425	4	.155
1426	2	1.116
1427	4	.155
1428	3	.362
1429	3	.362
1430	3	.772
1431	3	.362
1432	3	.362
1433	3	.362
1434	3	1.136

1435	4	.916
1436	4	.916
1437	3	.362
1438	3	.362
1439	4	.155
1440	3	1.009
1441	3	.362
1442	3	.362
1443	3	.772
1444	3	.362
1445	2	1.055
1446	3	.362
1447	4	.155
1448	4	.155
1449	4	.155
1450	3	.362
1451	2	1.336
1452	3	.772
1453	3	.362
1454	3	.908
1455	4	.951
1456	4	.916
1457	3	.908
1458	3	1.731
1459	3	.362
1460	5	.979
1461	3	.964

1462	5	.751
1463	3	.772
1464	4	.155
1465	4	.155
1466	3	.772
1467	5	.400
1468	1	.626
1469	3	.362
1470	5	.751
1471	3	.362
1472	4	.916
1473	5	.400
1474	2	.940
1475	5	.400
1476	4	.155
1477	4	.155
1478	3	1.050
1479	3	.772
1480	3	.772
1481	5	.751
1482	3	.772
1483	2	1.065
1484	4	.155
1485	3	.362
1486	4	.155
1487	5	.400
1488	3	.362

1489	5	.400
1490	4	.916
1491	4	.155
1492	5	.751
1493	3	.964
1494	5	1.082
1495	4	.979
1496	4	.951
1497	3	.772
1498	4	.155
1499	5	.751
1500	3	.964
1501	3	.362
1502	4	.916
1503	3	1.176
1504	3	.362
1505	3	.362
1506	1	.626
1507	1	.626
1508	4	.953
1509	4	.155
1510	4	.155
1511	1	.626
1512	4	.155
1513	1	.626
1514	4	.155
1515	4	.969

1516	1	.626
1517	1	.626
1518	1	.626
1519	5	.400
1520	1	.592
1521	4	.155
1522	4	.155
1523	4	.969
1524	4	.948
1525	1	1.025
1526	1	.626
1527	1	.626
1528	1	.626
1529	3	1.601
1530	1	.592
1531	4	.155
1532	1	1.045
1533	4	.155
1534	1	1.335
1535	4	.155
1536	1	1.134
1537	2	1.532
1538	1	1.690
1539	1	1.025
1540	3	.772
1541	1	1.311
1542	5	1.023

1543	1	1.410
1544	1	.592
1545	1	1.430
1546	3	1.741
1547	4	.155
1548	1	.626
1549	4	.155
1550	1	1.245
1551	4	.155
1552	4	.155
1553	3	1.286
1554	1	.626
1555	1	1.045
1556	4	.155
1557	1	.626
1558	1	.626
1559	4	.155
1560	4	.155
1561	1	.626
1562	1	1.025
1563	4	.948
1564	1	.592
1565	4	.975
1566	1	.626
1567	4	.155
1568	4	.155
1569	1	.626

1570	5	.400
1571	4	.155
1572	5	1.204
1573	1	.592
1574	4	.155
1575	1	.626
1576	4	.155
1577	1	1.060
1578	1	.626
1579	1	1.134
1580	1	.592
1581	1	.626
1582	4	.155
1583	1	.626
1584	3	1.286
1585	4	.969
1586	1	.626
1587	1	2.149
1588	1	1.025
1589	3	1.009
1590	3	1.909
1591	4	.155
1592	1	.626
1593	1	.626
1594	1	.626
1595	1	.626
1596	4	.951

1597	4	.951
1598	4	1.366
1599	1	.922
1600	1	.592
1601	4	.155
1602	1	.626
1603	5	.400
1604	2	1.035
1605	1	.922
1606	4	.155
1607	1	.626
1608	1	1.737
1609	4	.155
1610	1	.922
1611	4	.155
1612	1	.592
1613	4	.969
1614	4	.155
1615	5	1.241
1616	5	.400
1617	1	.626
1618	4	.155
1619	1	.922
1620	1	1.158
1621	1	.922
1622	1	1.245
1623	1	1.397

1624	1	1.045
1625	2	1.801
1626	1	1.040
1627	4	.155
1628	1	1.474
1629	4	1.346
1630	4	.155
1631	4	.969
1632	4	.155
1633	4	.155
1634	3	1.286
1635	4	.975
1636	1	1.045
1637	2	.997
1638	1	1.025
1639	5	.400
1640	4	.951
1641	3	1.218
1642	1	.592
1643	1	.626
1644	1	.922
1645	3	1.195
1646	1	.922
1647	3	1.742
1648	3	.362
1649	1	1.483
1650	5	.875

1651	4	.155
1652	1	1.060
1653	1	.592
1654	1	.592
1655	4	.155
1656	2	1.283
1657	3	.772
1658	1	.626
1659	1	.592
1660	1	1.045
1661	4	.155
1662	1	.922
1663	4	.155
1664	1	.626
1665	3	1.218
1666	1	1.045
1667	1	1.045
1668	1	1.045
1669	4	.155
1670	4	.155
1671	1	.922
1672	4	.155
1673	2	1.249
1674	1	.626
1675	4	.155
1676	1	1.116
1677	3	1.705

1678	1	1.122
1679	1	1.045
1680	1	.626
1681	4	.155
1682	1	.922
1683	1	1.116
1684	4	.155
1685	1	.592
1686	4	.155
1687	4	.155
1688	4	.155
1689	2	1.283
1690	1	.592
1691	5	.875
1692	5	1.349
1693	1	.922
1694	1	.626
1695	1	.592
1696	4	.155
1697	4	.155
1698	4	.155
1699	4	.979
1700	1	.626
1701	3	.772
1702	4	.155
1703	4	.155
1704	5	.400

1705	1	1.122
1706	5	1.349
1707	5	.400
1708	4	.155
1709	4	.155
1710	3	1.529
1711	4	.953
1712	1	1.408
1713	1	.626
1714	1	1.258
1715	4	.969
1716	5	.400
1717	1	.922
1718	2	2.021
1719	3	1.635
1720	4	.155
1721	4	.155
1722	1	.922
1723	4	1.324
1724	4	.951
1725	3	1.734
1726	1	.626
1727	4	.979
1728	4	.992
1729	4	.155
1730	2	1.579
1731	2	1.302

1732	4	.155
1733	4	.155
1734	2	.628
1735	5	1.212
1736	4	1.366
1737	1	1.503
1738	4	.155
1739	3	.772
1740	4	.155
1741	1	1.335
1742	1	.922
1743	4	.979
1744	4	.155
1745	3	.362
1746	1	.626
1747	1	.626
1748	1	.626
1749	1	.626
1750	1	.626
1751	1	.592
1752	1	.626
1753	2	1.661
1754	2	1.055
1755	1	1.741
1756	5	.875
1757	1	.626
1758	4	.155

1759	4	.155
1760	1	.626
1761	4	.969
1762	4	.155
1763	4	.155
1764	3	.362
1765	4	.155
1766	1	1.025
1767	4	.155
1768	5	1.316
1769	1	.592
1770	5	1.212
1771	2	1.065
1772	3	.908
1773	1	.626
1774	4	.155
1775	4	.155
1776	3	.362
1777	3	1.529
1778	1	1.258
1779	1	1.158
1780	3	.964
1781	4	.948
1782	4	.155
1783	4	.155
1784	1	1.040
1785	2	.628

1786	1	1.025
1787	3	.958
1788	1	1.321
1789	3	.362
1790	4	.948
1791	4	.953
1792	1	1.620
1793	1	1.456
1794	4	.916
1795	1	1.245
1796	4	.155
1797	1	.592
1798	4	.155
1799	2	1.055
1800	1	1.258
1801	1	.626
1802	1	1.487
1803	3	.362
1804	5	1.204
1805	3	1.176
1806	4	.155
1807	3	1.705
1808	3	.958
1809	1	.922
1810	4	.155
1811	2	1.089
1812	3	.362

1813	1	1.116
1814	4	.155
1815	4	.155
1816	3	.772
1817	1	.592
1818	5	1.285
1819	4	.155
1820	4	.155
1821	4	.155
1822	5	.979
1823	4	.948
1824	1	.626
1825	2	1.283
1826	1	1.116
1827	2	1.035
1828	2	1.259
1829	3	.362
1830	4	.155
1831	4	.975
1832	1	.922
1833	1	1.060
1834	4	.948
1835	5	.875
1836	1	.592
1837	1	.922
1838	4	1.350
1839	4	.155

1840	2	.628
1841	4	.155
1842	5	.400
1843	4	.155
1844	5	.400
1845	5	.751
1846	4	.155
1847	5	1.241
1848	2	1.780
1849	4	.155
1850	1	.626
1851	4	.155
1852	1	.592
1853	1	1.483
1854	4	1.357
1855	1	.626
1856	1	1.104
1857	4	.155
1858	4	.155
1859	1	1.040
1860	4	.155
1861	1	.922
1862	3	.362
1863	4	.969
1864	1	.922
1865	4	.975
1866	1	.592

1867	4	.155
1868	1	1.245
1869	1	.922
1870	4	.155
1871	4	.155
1872	4	1.334
1873	5	.751
1874	5	.400
1875	4	1.313
1876	2	1.568
1877	4	.155
1878	5	.967
1879	1	.626
1880	4	.155
1881	1	.626
1882	4	.155
1883	1	.922
1884	1	.626
1885	1	1.580
1886	1	1.025
1887	4	.155
1888	1	.626
1889	1	1.040
1890	1	.922
1891	1	1.040
1892	4	.155
1893	1	.922

1894	3	.362
1895	5	.400
1896	2	.628
1897	1	.592
1898	2	.997
1899	1	.922
1900	4	.155
1901	1	1.040
1902	4	.951
1903	1	1.116
1904	4	.155
1905	1	1.124
1906	4	.155
1907	1	.922
1908	2	1.579
1909	4	.975
1910	1	1.258
1911	1	1.045
1912	1	1.245
1913	4	.953
1914	1	.922
1915	4	.155
1916	4	.948
1917	5	.967
1918	1	.922
1919	4	.916
1920	4	.916

1921	1	.626
1922	1	.626
1923	4	.155
1924	3	.362
1925	4	.155
1926	2	1.335
1927	1	.626
1928	3	.980
1929	5	1.082
1930	1	1.335
1931	2	1.717
1932	2	.628
1933	4	.155
1934	1	1.406
1935	5	1.241
1936	1	.626
1937	5	.875
1938	4	.969
1939	5	.751
1940	1	.626
1941	1	.626
1942	1	.592
1943	4	.155
1944	2	1.055
1945	1	.626
1946	3	1.176
1947	1	1.570

1948	4	.155
1949	4	1.313
1950	1	1.060
1951	4	.155
1952	4	.155
1953	5	1.763
1954	4	.155
1955	1	1.158
1956	1	.922
1957	1	.922
1958	5	.979
1959	4	.155
1960	4	.916
1961	2	1.717
1962	5	.967
1963	2	1.346
1964	5	.875
1965	4	.155
1966	4	.155
1967	1	.592
1968	1	1.321
1969	5	.400
1970	1	1.258
1971	4	.975
1972	4	.948
1973	3	.908
1974	4	.155

1975	1	.626
1976	2	1.302
1977	4	.155
1978	1	.626
1979	1	.626
1980	4	.953
1981	4	.155
1982	4	.155
1983	5	.400
1984	3	1.269
1985	2	.628
1986	4	.155
1987	3	.362
1988	1	.922
1989	3	.772
1990	5	.400
1991	1	1.122
1992	4	.969
1993	5	1.212
1994	5	.400
1995	4	.155
1996	4	.953
1997	4	.916
1998	1	1.040
1999	5	.400
2000	2	.997
2001	4	.953

2002	4	.155
2003	5	.400
2004	4	.155
2005	2	1.055
2006	1	1.258
2007	5	.400
2008	4	.155
2009	1	.592
2010	4	1.346
2011	5	.400
2012	5	.400
2013	3	.362
2014	5	1.394
2015	4	.155
2016	5	1.241
2017	4	.969
2018	4	.155
2019	5	1.418
2020	1	.922
2021	5	.979
2022	1	1.040
2023	4	.916
2024	2	1.532
2025	1	1.552
2026	4	.155
2027	5	1.250
2028	4	.948

2029	3	1.181
2030	5	.751
2031	4	.916
2032	4	.155
2033	4	.155
2034	4	.155
2035	4	.155
2036	4	.916
2037	3	.964
2038	1	.922
2039	2	1.055
2040	1	.626
2041	1	1.350
2042	3	1.020
2043	4	.948
2044	1	.626
2045	4	.948
2046	4	.155
2047	5	.751
2048	2	1.283
2049	1	.626
2050	5	1.082
2051	4	.975
2052	5	.751
2053	4	.155
2054	1	1.636
2055	2	1.246

2056	4	.155
2057	5	.400
2058	1	.626
2059	5	1.082
2060	5	.751
2061	4	.948
2062	1	1.060
2063	4	.155
2064	3	.362
2065	1	1.258
2066	4	.155
2067	5	.400
2068	2	.628
2069	3	1.218
2070	4	.155
2071	4	.975
2072	5	.751
2073	2	1.055
2074	2	1.099
2075	4	.155
2076	1	.922
2077	1	1.134
2078	1	1.350
2079	4	.155
2080	1	1.442
2081	3	1.369
2082	4	1.332

2083	5	.751
2084	1	.626
2085	4	.969
2086	3	.362
2087	4	.155
2088	4	.155
2089	4	.155
2090	4	.155
2091	4	.155
2092	4	.155
2093	4	.948
2094	4	.948
2095	1	1.060
2096	3	1.195
2097	4	.155
2098	4	.979
2099	3	.362
2100	4	.155
2101	1	.592
2102	4	.948
2103	5	.751
2104	3	.362
2105	1	1.060
2106	5	1.554
2107	1	.592
2108	5	.400
2109	5	.400

2110	5	.751
2111	5	.400
2112	3	.362
2113	2	.997
2114	1	.592
2115	1	.626
2116	2	.940
2117	4	.155
2118	5	.400
2119	2	1.089
2120	3	1.009
2121	1	.626
2122	1	1.025
2123	2	.628
2124	3	1.369
2125	3	.772
2126	4	.155
2127	5	.979
2128	1	1.045
2129	5	.979
2130	1	.922
2131	4	.155
2132	5	.400
2133	4	.155
2134	4	.155
2135	4	.155
2136	4	.916

2137	4	.155
2138	4	.975
2139	4	.155
2140	4	.155
2141	4	.916
2142	4	.948
2143	1	1.636
2144	5	.400
2145	4	.951
2146	1	.592
2147	1	.626
2148	4	.155
2149	2	1.099
2150	5	1.204
2151	4	.155
2152	4	.155
2153	2	1.089
2154	3	.772
2155	5	1.618
2156	1	1.045
2157	3	1.269
2158	5	.967
2159	3	.772
2160	4	1.313
2161	4	.155
2162	3	1.195
2163	2	1.365

2164	4	.916
2165	5	.751
2166	4	.155
2167	5	1.841
2168	3	.772
2169	1	1.060
2170	5	.400
2171	1	.922
2172	5	.751
2173	4	.155
2174	1	.626
2175	5	1.204
2176	4	.155
2177	1	1.040
2178	5	.400
2179	3	.772
2180	5	.400
2181	5	.400
2182	4	.155
2183	5	.400
2184	1	1.342
2185	5	.751
2186	1	1.060
2187	4	.155
2188	5	.400
2189	4	.155
2190	1	.922

2191	3	.980
2192	2	1.336
2193	5	.875
2194	5	1.212
2195	1	.626
2196	4	.155
2197	4	.155
2198	4	.155
2199	4	.916
2200	1	1.100
2201	1	1.350
2202	4	.155
2203	2	1.249
2204	3	.772
2205	2	1.089
2206	4	.155
2207	5	.751
2208	4	.155
2209	1	1.497
2210	1	.626
2211	4	1.309
2212	1	1.040
2213	5	1.315
2214	5	.400
2215	4	.155
2216	1	.626
2217	3	1.020

2218	3	1.136
2219	4	.155
2220	4	.155
2221	4	.155
2222	4	.155
2223	4	.155
2224	2	.628
2225	3	.958
2226	2	1.395
2227	4	.155
2228	4	.155
2229	4	.155
2230	3	.964
2231	5	.400
2232	4	.155
2233	4	.155
2234	5	1.250
2235	4	.155
2236	4	.155
2237	5	1.316
2238	4	.155
2239	1	.626
2240	3	.908
2241	4	.155
2242	5	.400
2243	3	.362
2244	5	.751

2245	2	.997
2246	4	.155
2247	4	.155
2248	2	1.246
2249	4	.155
2250	4	.155
2251	2	1.470
2252	5	.967
2253	3	.362
2254	4	.155
2255	5	1.212
2256	5	1.082
2257	2	.628
2258	3	.772
2259	4	.155
2260	5	.751
2261	3	.362
2262	1	1.712
2263	4	1.312
2264	5	1.212
2265	2	1.089
2266	4	.155
2267	4	.155
2268	5	.400
2269	5	.400
2270	4	.948
2271	4	.155

2272	1	1.045
2273	3	.772
2274	4	.951
2275	3	.362
2276	3	.362
2277	1	1.258
2278	3	.772
2279	1	.626
2280	4	.948
2281	5	1.082
2282	3	.362
2283	5	.751
2284	5	.400
2285	3	1.759
2286	4	1.355
2287	4	.155
2288	5	1.023
2289	3	.772
2290	3	.772
2291	5	.400
2292	4	.155
2293	2	.997
2294	4	.155
2295	4	.953
2296	5	.400
2297	4	.155
2298	4	.155

2299	5	.400
2300	5	.751
2301	3	.362
2302	5	1.491
2303	2	.803
2304	4	.975
2305	3	.772
2306	4	.155
2307	4	.155
2308	4	.948
2309	4	.155
2310	5	.400
2311	4	.155
2312	5	.400
2313	1	1.258
2314	5	1.434
2315	2	1.317
2316	4	.155
2317	5	.751
2318	3	.362
2319	4	.155
2320	4	.155
2321	4	.155
2322	4	.979
2323	4	.155
2324	3	.772
2325	3	.772

2326	3	.362
2327	5	1.250
2328	3	.362
2329	1	.922
2330	4	.155
2331	3	.964
2332	4	.155
2333	5	.751
2334	1	.922
2335	4	1.312
2336	4	.155
2337	5	.751
2338	3	1.136
2339	4	.916
2340	1	.922
2341	5	1.440
2342	3	1.176
2343	4	.155
2344	4	.155
2345	3	.362
2346	4	.916
2347	4	.155
2348	1	.626
2349	3	.980
2350	1	.922
2351	1	.626
2352	3	.362

2353	5	.400
2354	4	.155
2355	4	.155
2356	5	.400
2357	4	.155
2358	4	.948
2359	5	.400

Final Cluster Centers

	Cluster				
	1	2	3	4	5
Cerebrovascular Accident	0	0	0	0	0
Heart failure	1	0	0	0	0
DIABETES MELLITUS	1	0	0	0	0
OBESITY_max	0	0	0	0	0
ASCITES_max	0	1	0	0	1
Esophageal varices	0	1	0	0	0
DIGESTIVE BLEEDING	0	1	0	0	0
ENCEPHALOPATHY_max	0	0	0	0	0
Hepatocellular Carcinoma	0	0	0	0	0
Hepatorenal syndrome	0	0	0	0	0
Portal hypertension	0	1	1	0	1

Distances between Final Cluster Centers

Cluster	1	2	3	4	5
1		1.730	1.262	.925	1.573
2	1.730		1.248	1.614	1.034
3	1.262	1.248		1.047	1.009
4	.925	1.614	1.047		1.361

5	1.573	1.034	1.009	1.361	

ANOVA

	Cluster		Error			
	Mean Square	df	Mean Square	df	F	Sig.
Cerebrovascular Accident	1.221	4	.052	2354	23.308	.000
Heart failure	43.852	4	.079	2354	556.043	.000
DIABETES MELLITUS	18.869	4	.104	2354	181.549	.000
OBESITY_max	.858	4	.056	2354	15.328	.000
ASCITES_max	100.940	4	.050	2354	2025.484	.000
Esophageal varices	32.724	4	.125	2354	261.490	.000
DIGESTIVE BLEEDING	29.985	4	.034	2354	887.395	.000
ENCEPHALOPATHY_max	5.212	4	.096	2354	54.128	.000
Hepatocellular Carcinoma	.077	4	.059	2354	1.293	.270
Hepatorenal syndrome	3.443	4	.082	2354	42.176	.000
Portal hypertension	128.757	4	.030	2354	4313.505	.000

The F tests should be used only for descriptive purposes because the clusters have been chosen to maximize the differences among cases in different clusters. The observed significance levels are not corrected for this and thus cannot be interpreted as tests of the hypothesis that the cluster means are equal.

Number of Cases in each Cluster

Cluster	1	440.000
	2	235.000
	3	554.000
	4	607.000
	5	523.000
Valid		2359.000
Missing		.000

Table S37. Hospitalization frequency stratification by diagnostic.

Descriptives

	Chronic hepatitis C (1=yes, 0=no)			Statistic	Std. Error	Bootstrap[a] Bias	Bootstrap[a] Std. Error	Bootstrap[a] 95% Confidence Interval Lower	Bootstrap[a] 95% Confidence Interval Upper
Number_Hospitalizations	no	Mean		3.74	.070	.00	.07	3.60	3.87
		95% Confidence Interval for Mean	Lower Bound	3.60					
			Upper Bound	3.88					
		5% Trimmed Mean		3.25		.00	.06	3.13	3.37
		Median		2.00		.05	.23	2.00	3.00
		Variance		13.465		-.011	.610	12.232	14.677
		Std. Deviation		3.669		-.002	.083	3.497	3.831
		Minimum		1					
		Maximum		19					
		Range		18					
		Interquartile Range		4		0	0	3	4
		Skewness		1.992	.047	-.002	.055	1.886	2.107

		Kurtosis		3.912	.094	-.005	.304	3.336	4.531
	yes	Mean		3.99	.134	-.01	.14	3.71	4.27
		95% Confidence Interval for Mean	Lower Bound	3.73					
			Upper Bound	4.26					
		5% Trimmed Mean		3.07		.00	.10	2.87	3.30
		Median		2.00		.00	.00	2.00	2.00
		Variance		29.027		-.176	2.815	23.716	35.025
		Std. Deviation		5.388		-.023	.262	4.870	5.918
		Minimum		1					
		Maximum		31					
		Range		30					
		Interquartile Range		4		0	0	3	4
		Skewness		3.195	.061	.002	.119	2.977	3.438
		Kurtosis		11.567	.121	.083	1.102	9.571	13.864

a. Unless otherwise noted, bootstrap results are based on 1000 bootstrap samples.

Percentiles

			Bootstrap[a]		
					95% Confidence Interval
Chronic hepatitis C (1=yes, 0=no)	Percentiles	Percentile	Bias	Std. Error	

							Lower
Weighted Average(Definition 1)	Number_Hospitalizations	no	5	1.00	.00	.00	1.00
			10	1.00	.00	.00	1.00
			25	1.00	.00	.00	1.00
			50	2.00	.05	.23	2.00
			75	5.00	-.20	.40	4.00

			90	9.00	-.02	.16	8.40
			95	12.00	.02	.20	12.00
		yes	5	1.00	.00	.00	1.00
			10	1.00	.00	.00	1.00
			25	1.00	.00	.00	1.00
			50	2.00	.00	.00	2.00

			75	5.00	-.28	.45	4.00
			90	9.00	-.37	.50	8.00
			95	16.00	-1.05	1.73	10.00
Tukey's Hinges	Number_Hospitalizations	no	25	1.00	.00	.00	1.00
			50	2.00	.05	.23	2.00
			75	5.00	-.21	.40	4.00

	yes	25	1.00	.00	.00	1.00
		50	2.00	.00	.00	2.00
		75	5.00	-.28	.45	4.00

a. Unless otherwise noted, bootstrap results are based on 1000 bootstrap samples.

Extreme Values

	Chronic hepatitis C (1=yes, 0=no)			Case Number	YEAR	Value
Number_Hospitalizations	no	Highest	1	1402	2020	19
			2	1403	2020	19
			3	1404	2020	19
			4	1405	2020	19
			5	1406	2020	19[a]
		Lowest	1	4340	2023	1
			2	4334	2020	1
			3	4327	2019	1
			4	4326	2021	1
			5	4323	2021	1[b]
	yes	Highest	1	2864	2019	31

		2	2865	2019	31
		3	2866	2019	31
		4	2867	2019	31
		5	2868	2019	31[c]
	Lowest	1	4339	2023	1
		2	4338	2023	1
		3	4337	2020	1
		4	4336	2020	1
		5	4335	2020	1[b]

a. Only a partial list of cases with the value 19 are shown in the table of upper extremes.

b. Only a partial list of cases with the value 1 are shown in the table of lower extremes.

c. Only a partial list of cases with the value 31 are shown in the table of upper extremes.

Descriptives

	Alcoholic hepatitis (1=yes, 0=no)			Statistic	Std. Error	Bootstrap[a] Bias	Bootstrap[a] Std. Error	Bootstrap[a] 95% Confidence Interval Lower	Bootstrap[a] 95% Confidence Interval Upper
Number_Hospitalizations	no	Mean		4.25	.088	-.01	.09	4.06	4.43
		95% Confidence Interval for Mean	Lower Bound	4.08					
			Upper Bound	4.42					
		5% Trimmed Mean		3.53		.00	.08	3.38	3.68
		Median		2.00		.19	.39	2.00	3.00
		Variance		23.768		-.097	1.567	20.508	26.853
		Std. Deviation		4.875		-.013	.161	4.529	5.182
		Minimum		1					

		Maximum		31					
		Range		30					
		Interquartile Range		4		0	0	4	5
		Skewness		2.754	.044	-.006	.089	2.573	2.920
		Kurtosis		9.555	.089	-.028	.649	8.302	10.841
	yes	Mean		2.86	.076	.00	.07	2.71	3.00
		95% Confidence Interval for Mean	Lower Bound	2.71					
			Upper Bound	3.01					
		5% Trimmed Mean		2.48		.00	.07	2.34	2.62
		Median		2.00		.00	.00	2.00	2.00
		Variance		7.411		-.005	.535	6.334	8.504
		Std. Deviation		2.722		-.003	.098	2.517	2.916
		Minimum		1					
		Maximum		17					
		Range		16					
		Interquartile Range		2		0	0	2	3
		Skewness		2.164	.068	-.001	.109	1.953	2.392
		Kurtosis		5.082	.136	-.005	.716	3.705	6.566

a. Unless otherwise noted, bootstrap results are based on 1000 bootstrap samples.

Percentiles

		Alcoholic hepatitis (1=yes, 0=no)	Percentiles	Percentile	Bootstrap[a] Bias	Std. Error	95% Confidence Interval Lower	Upper
Weighted Average(Definition 1)	Number_Hospitalizations	no	5	1.00	.00	.00	1.00	1.00
			10	1.00	.00	.00	1.00	1.00

			25	1.00	.00	.00	1.00	1.00
			50	2.00	.19	.39	2.00	3.00
			75	5.00	.09	.28	5.00	6.00
			90	10.00	-.32	.46	9.00	10.00
			95	15.00	-.18	.53	14.00	16.00
		yes	5	1.00	.00	.00	1.00	1.00
			10	1.00	.00	.00	1.00	1.00
			25	1.00	.00	.00	1.00	1.00
			50	2.00	.00	.00	2.00	2.00
			75	3.00	.44	.49	3.00	4.00
			90	6.40	.08	.50	6.00	7.00
			95	9.00	.12	.35	9.00	10.00
Tukey's Hinges	Number_Hospitalizations	no	25	1.00	.00	.00	1.00	1.00
			50	2.00	.19	.39	2.00	3.00
			75	5.00	.09	.28	5.00	6.00
		yes	25	1.00	.00	.00	1.00	1.00
			50	2.00	.00	.00	2.00	2.00
			75	3.00	.44	.49	3.00	4.00

a. Unless otherwise noted, bootstrap results are based on 1000 bootstrap samples.

Extreme Values

	Alcoholic hepatitis (1=yes, 0=no)			Case Number	YEAR	Value
Number_Hospitalizations	no	Highest	1	2864	2019	31
			2	2865	2019	31
			3	2866	2019	31
			4	2867	2019	31
			5	2868	2019	31[a]
		Lowest	1	4340	2023	1

			2	4339	2023	1
			3	4338	2023	1
			4	4337	2020	1
			5	4336	2020	1[b]
	yes	Highest	1	1616	2019	17
			2	1617	2020	17
			3	1618	2020	17
			4	1647	2019	17
			5	615	2019	15[c]
		Lowest	1	4327	2019	1
			2	4326	2021	1
			3	4323	2021	1
			4	4322	2022	1
			5	4319	2022	1[b]

a. Only a partial list of cases with the value 31 are shown in the table of upper extremes.

b. Only a partial list of cases with the value 1 are shown in the table of lower extremes.

c. Only a partial list of cases with the value 15 are shown in the table of upper extremes.

Descriptives

						Bootstrap[a]			
					Std.		Std.	95% Confidence Interval	
	Non-alcoholic cirrhosis			Statistic	Error	Bias	Error	Lower	Upper
Number_Hospitalizations	no	Mean		3.45	.076	-.01	.08	3.30	3.61
		95% Confidence Interval for Mean	Lower Bound	3.30					
			Upper Bound	3.60					
		5% Trimmed Mean		2.78		.00	.05	2.68	2.88
		Median		2.00		.00	.00	2.00	2.00
		Variance		18.458		-.093	1.491	15.662	21.611

		Std. Deviation		4.296		-.014	.174	3.957	4.649
		Minimum		1					
		Maximum		31					
		Range		30					
		Interquartile Range		3		0	0	3	3
		Skewness		3.658	.043	-.007	.109	3.436	3.868
		Kurtosis		17.316	.086	-.006	1.060	15.275	19.561
	yes	Mean		4.92	.133	.00	.13	4.68	5.18
		95% Confidence Interval for Mean	Lower Bound	4.66					
			Upper Bound	5.18					
		5% Trimmed Mean		4.44		.00	.14	4.19	4.71
		Median		3.00		.00	.00	3.00	3.00
		Variance		20.084		-.031	1.089	17.993	22.285
		Std. Deviation		4.482		-.005	.122	4.242	4.721
		Minimum		1					
		Maximum		19					
		Range		18					
		Interquartile Range		5		0	0	4	5
		Skewness		1.485	.073	-.003	.066	1.357	1.617
		Kurtosis		1.461	.145	-.001	.266	.974	2.003

a. Unless otherwise noted, bootstrap results are based on 1000 bootstrap samples.

Percentiles

		Non-alcoholic cirrhosis	Percentiles	Percentile	Bootstrap[a] Bias	Std. Error	95% Confidence Interval Lower	Upper
Weighted Average(Definition 1)	Number_Hospitalizations	no	5	1.00	.00	.00	1.00	1.00
			10	1.00	.00	.00	1.00	1.00

			25	1.00	.00	.00	1.00	1.00
			50	2.00	.00	.00	2.00	2.00
			75	4.00	.00	.00	4.00	4.00
			90	8.00	-.10	.30	7.00	8.00
			95	10.00	.05	.26	10.00	11.00
		yes	5	1.00	.00	.00	1.00	1.00
			10	1.00	.00	.00	1.00	1.00
			25	2.00	-.01	.08	2.00	2.00
			50	3.00	.00	.00	3.00	3.00
			75	6.50	.00	.50	6.00	7.00
			90	12.00	.04	.52	11.00	13.00
			95	15.00	.43	.82	15.00	17.00
Tukey's Hinges	Number_Hospitalizations	no	25	1.00	.00	.00	1.00	1.00
			50	2.00	.00	.00	2.00	2.00
			75	4.00	.00	.00	4.00	4.00
		yes	25	2.00	-.01	.08	2.00	2.00
			50	3.00	.00	.00	3.00	3.00
			75	6.00	.49	.49	6.00	7.00

a. Unless otherwise noted, bootstrap results are based on 1000 bootstrap samples.

Extreme Values

	Non-alcoholic cirrhosis			Case Number	YEAR	Value
Number_Hospitalizations	no	Highest	1	2864	2019	31
			2	2865	2019	31
			3	2866	2019	31
			4	2867	2019	31
			5	2868	2019	31[a]
		Lowest	1	4340	2023	1

			2	4339	2023	1
			3	4338	2023	1
			4	4337	2020	1
			5	4336	2020	1[b]
	yes	Highest	1	1402	2020	19
			2	1403	2020	19
			3	1404	2020	19
			4	1405	2020	19
			5	1406	2020	19[c]
		Lowest	1	4334	2020	1
			2	4318	2023	1
			3	4316	2021	1
			4	4274	2022	1
			5	4272	2021	1[b]

a. Only a partial list of cases with the value 31 are shown in the table of upper extremes.

b. Only a partial list of cases with the value 1 are shown in the table of lower extremes.

c. Only a partial list of cases with the value 19 are shown in the table of upper extremes.

Descriptives

	Alcoholic cirrhosis (1=yes, 0=no)			Statistic	Std. Error	Bootstrap[a] Bias	Bootstrap[a] Std. Error	95% Confidence Interval Lower	95% Confidence Interval Upper
Number_Hospitalizations	no	Mean		3.89	.071	.00	.07	3.74	4.02
		95% Confidence Interval for Mean	Lower Bound	3.75					
			Upper Bound	4.03					
		5% Trimmed Mean		3.21		.00	.06	3.10	3.32

		Median		2.00		.00	.00	2.00	2.00
		Variance		20.245		-.071	1.210	17.732	22.637
		Std. Deviation		4.499		-.010	.135	4.211	4.758
		Minimum		1					
		Maximum		31					
		Range		30					
		Interquartile Range		4		0	0	3	4
		Skewness		2.908	.038	-.008	.093	2.709	3.083
		Kurtosis		11.110	.077	-.051	.713	9.683	12.542
	yes	Mean		3.10	.138	-.01	.14	2.82	3.36
		95% Confidence Interval for Mean	Lower Bound	2.82					
			Upper Bound	3.37					
		5% Trimmed Mean		2.79		.00	.13	2.55	3.05
		Median		2.00		.24	.42	2.00	3.00
		Variance		5.588		-.060	.847	4.000	7.338
		Std. Deviation		2.364		-.020	.180	2.000	2.709
		Minimum		1					
		Maximum		15					
		Range		14					
		Interquartile Range		2		0	0	2	3
		Skewness		2.025	.142	-.028	.210	1.599	2.422
		Kurtosis		4.994	.283	-.104	1.328	2.657	7.811

a. Unless otherwise noted, bootstrap results are based on 1000 bootstrap samples.

Percentiles

Percentiles	Percentile	Bootstrap[a]

		Alcoholic cirrhosis (1=yes, 0=no)			Bias	Std. Error	95% Confidence Interval	
							Lower	Upper
Weighted Average(Definition 1)	Number_Hospitalizations	no	5	1.00	.00	.00	1.00	1.00
			10	1.00	.00	.00	1.00	1.00
			25	1.00	.00	.00	1.00	1.00
			50	2.00	.00	.00	2.00	2.00
			75	5.00	-.04	.19	4.00	5.00
			90	9.00	.00	.11	9.00	9.00
			95	13.00	-.01	.79	12.00	14.00
		yes	5	1.00	.00	.00	1.00	1.00
			10	1.00	.00	.00	1.00	1.00
			25	2.00	-.44	.49	1.00	2.00
			50	2.00	.24	.42	2.00	3.00
			75	4.00	-.03	.17	3.00	4.00
			90	6.00	-.07	.63	5.00	7.00
			95	9.00	-.86	1.05	7.00	10.00
Tukey's Hinges	Number_Hospitalizations	no	25	1.00	.00	.00	1.00	1.00
			50	2.00	.00	.00	2.00	2.00
			75	5.00	-.04	.19	4.00	5.00
		yes	25	2.00	-.42	.49	1.00	2.00
			50	2.00	.24	.42	2.00	3.00
			75	4.00	-.03	.18	3.00	4.00

a. Unless otherwise noted, bootstrap results are based on 1000 bootstrap samples.

Extreme Values

Alcoholic cirrhosis (1=yes, 0=no)	Case Number	YEAR	Value

Number_Hospitalizations	no	Highest	1	2864	2019	31
			2	2865	2019	31
			3	2866	2019	31
			4	2867	2019	31
			5	2868	2019	31[a]
		Lowest	1	4339	2023	1
			2	4338	2023	1
			3	4337	2020	1
			4	4336	2020	1
			5	4335	2020	1[b]
	yes	Highest	1	2004	2019	15
			2	818	2020	12
			3	819	2020	12
			4	820	2020	12
			5	821	2020	12[c]
		Lowest	1	4340	2023	1
			2	4253	2023	1
			3	4112	2020	1
			4	4096	2023	1
			5	4066	2019	1[b]

a. Only a partial list of cases with the value 31 are shown in the table of upper extremes.

b. Only a partial list of cases with the value 1 are shown in the table of lower extremes.

c. Only a partial list of cases with the value 12 are shown in the table of upper extremes.

Table S38. Patient-Level Hospitalization stratification by Age Group.

Descriptives

	AGE_GROUP			Statistic	Std. Error	Bootstrap[a] Bias	Bootstrap[a] Std. Error	Bootstrap[a] 95% Confidence Interval Lower	Bootstrap[a] 95% Confidence Interval Upper
Number_Hospitalizations	under or equal 30	Mean		2.12	.256	.00	.25	1.67	2.63
		95% Confidence Interval for Mean	Lower Bound	1.58					
			Upper Bound	2.66					
		5% Trimmed Mean		2.08		.01	.28	1.60	2.64
		Median		2.00		.01	.43	1.00	3.00
		Variance		1.110		-.084	.282	.497	1.590
		Std. Deviation		1.054		-.051	.142	.705	1.261
		Minimum		1					
		Maximum		4					
		Range		3					
		Interquartile Range		2		0	1	1	3
		Skewness		.466	.550	-.062	.433	-.439	1.266
		Kurtosis		-.931	1.063	.155	.857	-1.860	1.393
	31-40	Mean		2.77	.184	.00	.19	2.43	3.15
		95% Confidence Interval for Mean	Lower Bound	2.40					
			Upper Bound	3.13					
		5% Trimmed Mean		2.52		.01	.20	2.16	2.95
		Median		2.00		-.07	.27	1.00	2.00
		Variance		5.327		-.052	.741	3.809	6.725
		Std. Deviation		2.308		-.017	.163	1.952	2.593

		Minimum		1					
		Maximum		9					
		Range		8					
		Interquartile Range		3		0	0	2	3
		Skewness		1.404	.193	-.006	.173	1.061	1.761
		Kurtosis		1.062	.384	.039	.658	-.020	2.611
	41-50	Mean		3.24	.104	.00	.11	3.04	3.45
		95% Confidence Interval for Mean	Lower Bound	3.03					
			Upper Bound	3.44					
		5% Trimmed Mean		2.97		.00	.11	2.76	3.20
		Median		2.00		.07	.26	2.00	3.00
		Variance		7.016		-.031	.515	5.974	7.990
		Std. Deviation		2.649		-.008	.098	2.444	2.827
		Minimum		1					
		Maximum		15					
		Range		14					
		Interquartile Range		3		0	0	3	3
		Skewness		1.408	.096	.000	.099	1.218	1.604
		Kurtosis		1.301	.192	.012	.470	.510	2.285
	51-60	Mean		4.38	.135	.01	.13	4.14	4.66
		95% Confidence Interval for Mean	Lower Bound	4.12					
			Upper Bound	4.65					
		5% Trimmed Mean		3.84		.01	.15	3.57	4.14

		Median		3.00		-.21	.40	2.00	3.00
		Variance		20.718		.029	1.243	18.341	23.049
		Std. Deviation		4.552		.001	.136	4.283	4.801
		Minimum		1					
		Maximum		19					
		Range		18					
		Interquartile Range		5		0	0	4	5
		Skewness		1.715	.072	-.005	.074	1.572	1.868
		Kurtosis		2.097	.145	-.010	.342	1.486	2.827
	61-70	Mean		3.66	.093	.00	.09	3.47	3.85
		95% Confidence Interval for Mean	Lower Bound	3.47					
			Upper Bound	3.84					
		5% Trimmed Mean		3.21		.00	.09	3.02	3.39
		Median		2.00		.01	.10	2.00	2.00
		Variance		12.022		-.032	.756	10.618	13.574
		Std. Deviation		3.467		-.006	.109	3.259	3.684
		Minimum		1					
		Maximum		19					
		Range		18					
		Interquartile Range		4		0	0	3	4
		Skewness		1.843	.066	-.005	.077	1.682	1.995
		Kurtosis		3.313	.131	-.017	.409	2.545	4.196
	71-80	Mean		3.33	.135	.00	.13	3.05	3.59
		95% Confidence Interval for Mean	Lower Bound	3.06					

		Upper Bound	3.59					
	5% Trimmed Mean		2.81		.00	.11	2.59	3.03
	Median		2.00		.00	.00	2.00	2.00
	Variance		13.320		-.005	1.679	10.196	16.911
	Std. Deviation		3.650		-.008	.230	3.193	4.112
	Minimum		1					
	Maximum		22					
	Range		21					
	Interquartile Range		3		0	0	2	3
	Skewness		2.895	.090	-.016	.164	2.552	3.215
	Kurtosis		10.520	.180	-.070	1.181	8.433	13.098
over or egual 81	Mean		6.04	.611	.04	.61	4.93	7.28
	95% Confidence Interval for Mean	Lower Bound	4.83					
		Upper Bound	7.24					
	5% Trimmed Mean		4.93		.04	.68	3.70	6.31
	Median		1.00		.15	.35	1.00	2.00
	Variance		98.923		.263	11.938	75.664	123.343
	Std. Deviation		9.946		-.005	.604	8.699	11.106
	Minimum		1					
	Maximum		31					
	Range		30					
	Interquartile Range		3		0	1	1	6
	Skewness		1.929	.150	.005	.225	1.524	2.415
	Kurtosis		2.005	.298	.080	.956	.532	4.215

a. Unless otherwise noted, bootstrap results are based on 1000 bootstrap samples.

Percentiles

					Bootstrap[a]			
							95% Confidence Interval	
		AGE_GROUP	Percentiles	Percentile	Bias	Std. Error	Lower	Upper
Weighted Average(Definition 1)	Number_ Hospitalizations	under or equal 30	5	1.00	.00	.06	1.00	1.00
			10	1.00	.01	.08	1.00	1.00
			25	1.00	.17	.34	1.00	2.00
			50	2.00	.01	.43	1.00	3.00
			75	3.00	-.08	.46	2.00	4.00
			90	4.00	-.38[b]	.44[b]	3.00[b]	4.00[b]
			95	.	1.80E+308[c]	.23[c]	3.00[c]	4.00[c]
		31-40	5	1.00	.00	.00	1.00	1.00
			10	1.00	.00	.00	1.00	1.00
			25	1.00	.00	.00	1.00	1.00
			50	2.00	-.07	.27	1.00	2.00
			75	4.00	-.30	.48	3.00	4.00
			90	7.00	-.33	.64	5.00	7.60
			95	9.00	-.67	.91	7.00	9.00
		41-50	5	1.00	.00	.00	1.00	1.00
			10	1.00	.00	.00	1.00	1.00
			25	1.00	.00	.00	1.00	1.00
			50	2.00	.07	.26	2.00	3.00
			75	4.00	.02	.12	4.00	4.00
			90	7.00	.54	.68	7.00	9.00
			95	10.00	-.42	.48	9.00	10.00
		51-60	5	1.00	.00	.00	1.00	1.00

		10	1.00	.00	.00	1.00	1.00
		25	1.00	.00	.00	1.00	1.00
		50	3.00	-.21	.40	2.00	3.00
		75	6.00	-.29	.45	5.00	6.00
		90	12.00	-.19	.84	10.00	13.00
		95	17.00	-.20	.59	15.00	17.00
	61-70	5	1.00	.00	.00	1.00	1.00
		10	1.00	.00	.00	1.00	1.00
		25	1.00	.00	.00	1.00	1.00
		50	2.00	.01	.10	2.00	2.00
		75	5.00	-.03	.18	4.00	5.00
		90	8.00	.14	.34	8.00	9.00
		95	12.00	-.47	.86	10.00	12.00
	71-80	5	1.00	.00	.00	1.00	1.00
		10	1.00	.00	.00	1.00	1.00
		25	1.00	.00	.00	1.00	1.00
		50	2.00	.00	.00	2.00	2.00
		75	4.00	-.17	.38	3.00	4.00
		90	8.00	.05	.58	7.00	9.00
		95	10.00	.02	.20	10.00	10.00
	over or egual 81	5	1.00	.00	.00	1.00	1.00
		10	1.00	.00	.00	1.00	1.00
		25	1.00	.00	.00	1.00	1.00
		50	1.00	.15	.35	1.00	2.00
		75	4.00	.26	1.42	2.00	7.00
		90	31.00	-1.51	3.50	22.00	31.00
		95	31.00	.00	.00	31.00	31.00

Tukey's Hinges	Number_ Hospitalizations	under or equal 30	25	1.00	.22	.39	1.00	2.00
			50	2.00	.01	.43	1.00	3.00
			75	3.00	-.17	.47	2.00	4.00
		31-40	25	1.00	.00	.00	1.00	1.00
			50	2.00	-.07	.27	1.00	2.00
			75	4.00	-.33	.49	3.00	4.00
		41-50	25	1.00	.00	.00	1.00	1.00
			50	2.00	.07	.26	2.00	3.00
			75	4.00	.01	.12	4.00	4.00
		51-60	25	1.00	.00	.00	1.00	1.00
			50	3.00	-.21	.40	2.00	3.00
			75	6.00	-.30	.46	5.00	6.00
		61-70	25	1.00	.00	.00	1.00	1.00
			50	2.00	.01	.10	2.00	2.00
			75	5.00	-.04	.19	4.00	5.00
		71-80	25	1.00	.00	.00	1.00	1.00
			50	2.00	.00	.00	2.00	2.00
			75	4.00	-.19	.39	3.00	4.00
		over or egual 81	25	1.00	.00	.00	1.00	1.00
			50	1.00	.15	.35	1.00	2.00
			75	4.00	.19	1.41	2.00	7.00

a. Unless otherwise noted, bootstrap results are based on 1000 bootstrap samples.

b. Based on 977 samples.

c. Based on 259 samples.

Extreme Values[m]

	AGE_GROUP			Case Number	YEAR	Value
Number_Hospitalizations	under or equal 30	Highest	1	4301	2023	4

			2	4302	2023	4
			3	2751	2019	3
			4	2752	2019	3
			5	2753	2019	3[a]
		Lowest	1	4319	2023	1
			2	2750	2021	1
			3	2747	2019	1
			4	2746	2022	1
			5	2745	2021	1[b]
	31-40	Highest	1	2629	2020	9
			2	2630	2020	9
			3	2631	2020	9
			4	2632	2020	9
			5	2633	2020	9[c]
		Lowest	1	4329	2023	1
			2	4328	2019	1
			3	4318	2021	1
			4	4317	2021	1
			5	4316	2023	1[b]
	41-50	Highest	1	2005	2019	15
			2	2025	2019	11
			3	2026	2020	11
			4	2027	2020	11
			5	2028	2020	11[d]
		Lowest	1	4327	2021	1
			2	4326	2021	1
			3	4293	2021	1

			4	4290	2022	1
			5	4289	2019	1[b]
	51-60	Highest	1	1402	2020	19
			2	1403	2020	19
			3	1404	2020	19
			4	1405	2020	19
			5	1406	2020	19[e]
		Lowest	1	4341	2023	1
			2	4340	2023	1
			3	4339	2023	1
			4	4338	2020	1
			5	4325	2021	1[b]
	61-70	Highest	1	1417	2023	19
			2	1418	2023	19
			3	1419	2023	19
			4	1420	2023	19
			5	3427	2019	16[f]
		Lowest	1	4337	2020	1
			2	4336	2020	1
			3	4335	2020	1
			4	4322	2023	1
			5	3937	2023	1[b]
	71-80	Highest	1	2931	2019	22
			2	2932	2019	22
			3	2933	2019	22
			4	2934	2019	22
			5	2935	2019	22[g]

		Lowest	1	4334	2020	1
			2	4333	2019	1
			3	4321	2022	1
			4	4320	2022	1
			5	3501	2023	1[b]
	over or egual 81	Highest	1	2865	2019	31
			2	2866	2019	31
			3	2867	2019	31
			4	2868	2019	31
			5	2869	2019	31[h]
		Lowest	1	4332	2020	1
			2	4331	2019	1
			3	4330	2019	1
			4	3018	2023	1
			5	3008	2023	1[b]

a. Only a partial list of cases with the value 3 are shown in the table of upper extremes.

b. Only a partial list of cases with the value 1 are shown in the table of lower extremes.

c. Only a partial list of cases with the value 9 are shown in the table of upper extremes.

d. Only a partial list of cases with the value 11 are shown in the table of upper extremes.

e. Only a partial list of cases with the value 19 are shown in the table of upper extremes.

f. Only a partial list of cases with the value 16 are shown in the table of upper extremes.

g. Only a partial list of cases with the value 22 are shown in the table of upper extremes.

h. Only a partial list of cases with the value 31 are shown in the table of upper extremes.

m. For one or more split files, the requested number of extreme values exceeds the number of data points. A smaller number of extremes is displayed.

Table S39. Descriptive Statistics of Hospitalization Frequency by Sex and Diagnostic Group.

Descriptives

	SEX			Statistic	Std. Error	Bootstrap[a] Bias	Std. Error	95% Confidence Interval Lower	Upper
Number_Hospitalizations	FEMALE	Mean		4.19	.134	.00	.14	3.92	4.47
		95% Confidence Interval for Mean	Lower Bound	3.93					
			Upper Bound	4.46					
		5% Trimmed Mean		3.31		.00	.10	3.11	3.52
		Median		2.00		.01	.08	2.00	2.00
		Variance		28.546		-.077	2.692	23.288	34.080
		Std. Deviation		5.343		-.013	.252	4.826	5.838
		Minimum		1					
		Maximum		31					
		Range		30					
		Interquartile Range		4		0	0	4	4
		Skewness		3.183	.062	-.002	.113	2.969	3.412
		Kurtosis		11.715	.123	.046	1.078	9.715	14.031
	MALE	Mean		3.63	.071	.00	.07	3.49	3.77
		95% Confidence Interval for Mean	Lower Bound	3.49					
			Upper Bound	3.77					
		5% Trimmed Mean		3.11		.00	.07	2.97	3.24
		Median		2.00		.00	.00	2.00	2.00
		Variance		13.896		-.012	.651	12.640	15.174
		Std. Deviation		3.728		-.003	.087	3.555	3.895

	Minimum	1					
	Maximum	19					
	Range	18					
	Interquartile Range	3		0	0	3	4
	Skewness	2.081	.047	.000	.056	1.977	2.197
	Kurtosis	4.267	.093	.009	.323	3.689	4.927

a. Unless otherwise noted, bootstrap results are based on 1000 bootstrap samples.

Percentiles

					Bootstrap[a]			
							95% Confidence Interval	
		SEX	Percentiles	Percentile	Bias	Std. Error	Lower	Upper
Weighted Average(Definition 1)	Number_Hospitalizations	FEMALE	5	1.00	.00	.00	1.00	1.00
			10	1.00	.00	.00	1.00	1.00
			25	1.00	.00	.00	1.00	1.00
			50	2.00	.01	.08	2.00	2.00
			75	5.00	-.02	.13	5.00	5.00
			90	9.00	-.09	.38	8.00	10.00
			95	14.00	-.37	1.31	12.00	16.00
		MALE	5	1.00	.00	.00	1.00	1.00
			10	1.00	.00	.00	1.00	1.00
			25	1.00	.00	.00	1.00	1.00
			50	2.00	.00	.00	2.00	2.00
			75	4.00	.29	.45	4.00	5.00
			90	9.00	-.11	.32	8.00	9.00
			95	12.00	.05	.57	11.00	13.00
Tukey's Hinges	Number_Hospitalizations	FEMALE	25	1.00	.00	.00	1.00	1.00
			50	2.00	.01	.08	2.00	2.00

			75	5.00	-.02	.13	5.00	5.00
		MALE	25	1.00	.00	.00	1.00	1.00
			50	2.00	.00	.00	2.00	2.00
			75	4.00	.29	.45	4.00	5.00

a. Unless otherwise noted, bootstrap results are based on 1000 bootstrap samples.

Extreme Values

	SEX			Case Number	YEAR	Value
Number_Hospitalizations	FEMALE	Highest	1	2865	2019	31
			2	2866	2019	31
			3	2867	2019	31
			4	2868	2019	31
			5	2869	2019	31[a]
		Lowest	1	4340	2023	1
			2	4339	2023	1
			3	4338	2020	1
			4	4337	2020	1
			5	4336	2020	1[b]
	MALE	Highest	1	1402	2020	19
			2	1403	2020	19
			3	1404	2020	19
			4	1405	2020	19
			5	1406	2020	19[c]
		Lowest	1	4341	2023	1
			2	4329	2023	1
			3	4328	2019	1
			4	4327	2021	1
			5	4326	2021	1[b]

a. Only a partial list of cases with the value 31 are shown in the table of upper extremes.

b. Only a partial list of cases with the value 1 are shown in the table of lower extremes.

c. Only a partial list of cases with the value 19 are shown in the table of upper extremes.

Table S40. Spearman correlation.

Correlations

					Number_Hospitalizations	AGE_GROUP	SEX	Chronic hepatitis C (1=yes, 0=no)	Alcoholic hepatitis (1=yes, 0=no)	Non-alcoholic cyrrhosis	Alcoholic cirrhosis (1=yes, 0=no)	Arterial hypertension	Cerebrovascular Accident	Heart failure	DIABETES MELLITUS	OBESITY	Child-Pugh	ASCITES	Esophageal varices	DIGESTIVE BLEEDING	ENCEPHALOPATHY	Hepatocellular Carcinoma	Hepatorenal syndrome	Portal hypertension
Spearman's rho	Number_Hospitalizations	Correlation Coefficient			1.000	-.048**	-.021	-.066**	-.133**	.206**	.011	-.122**	-.092**	-.072**	-.038*	-.034*	.079**	.206**	.091**	-.001	-.001	-.009	.028	.176**
		Sig. (2-tailed)			.	.002	.168	<.001	<.001	<.001	.475	<.001	<.001	<.001	.012	.026	<.001	<.001	<.001	.926	.962	.564	.061	<.001
		N			4339	4339	4339	4339	4339	4339	4339	4339	4339	4339	4339	4339	4339	4339	4339	4339	4339	4339	4339	4339
		Bootstrap[c]	Bias		.000	-.001	.000	-.001	.000	.001	.000	.000	-.001	-.001	.000	.000	.000	.000	-.001	-.001	-.001	.001	.000	.001
			Std. Error		.000	.015	.015	.015	.014	.015	.013	.015	.013	.015	.015	.016	.015	.015	.015	.014	.016	.016	.015	.015
			95% Confidence Interval	Lower	1.000	-.078	-.053	-.099	-.161	.176	-.014	-.154	-.117	-.104	-.068	-.064	.049	.177	.060	-.029	-.033	-.039	-.001	.149
				Upper	1.000	-.019	.008	-.038	-.107	.236	.038	-.091	-.064	-.043	-.009	-.001	.110	.236	.119	.027	.032	.022	.058	.205
	AGE_GROUP	Correlation Coefficient			-.048**	1.000	-.316**	.347**	-.299**	-.006	-.115**	.286**	.102**	.268**	.128**	.036*	-.172**	-.109**	-.072**	-.039*	-.008	.025	-.021	-.226**
		Sig. (2-tailed)			.002	.	<.001	<.001	<.001	.712	<.001	<.001	<.001	<.001	<.001	.019	<.001	<.001	<.001	.011	.593	.100	.165	<.001
		N			4339	4339	4339	4339	4339	4339	4339	4339	4339	4339	4339	4339	4339	4339	4339	4339	4339	4339	4339	4339
		Bootstrap[c]	Bias		-.001	.000	.000	.000	.000	.001	-.001	.000	.000	.000	.000	.000	-.001	.000	.000	.000	.000	.000	.000	.000
			Std. Error		.015	.000	.015	.014	.013	.014	.013	.014	.013	.014	.014	.013	.014	.014	.015	.016	.015	.015	.014	.015
			95% Confidence Interval	Lower	-.078	1.000	-.346	.320	-.326	-.033	-.141	.258	.075	.240	.102	.009	-.201	-.135	-.101	-.070	-.036	-.004	-.049	-.257
				Upper	-.019	1.000	-.289	.375	-.272	.021	-.090	.313	.128	.295	.154	.061	-.145	-.081	-.043	-.008	.020	.054	.006	-.197
	SEX	Correlation Coefficient			-.021	-.316**	1.000	-.521**	.331**	.160**	.120**	-.210**	-.063**	-.164**	-.088**	-.061**	.220**	.160**	.048**	.013	.100**	.042**	.076**	.303**
		Sig. (2-tailed)			.168	<.001	.	<.001	<.001	<.001	<.001	<.001	<.001	<.001	<.001	<.001	<.001	<.001	.002	.379	<.001	.006	<.001	<.001
		N			4339	4339	4339	4339	4339	4339	4339	4339	4339	4339	4339	4339	4339	4339	4339	4339	4339	4339	4339	4339
		Bootstrap[c]	Bias		.000	.000	.000	.000	.000	.000	.000	.000	.000	.000	.001	.000	.000	-.001	.001	.000	.000	.000	.000	.000
			Std. Error		.015	.015	.000	.014	.012	.014	.012	.016	.016	.016	.016	.016	.014	.015	.015	.015	.013	.014	.014	.015
			95% Confidence Interval	Lower	-.053	-.346	1.000	-.548	.306	.132	.096	-.241	-.095	-.198	-.119	-.092	.194	.129	.019	-.019	.074	.013	.047	.274
				Upper	.008	-.289	1.000	-.493	.355	.188	.143	-.179	-.031	-.134	-.056	-.030	.248	.190	.077	.043	.126	.070	.100	.332
	Chronic hepatitis C (1=yes, 0=no)	Correlation Coefficient			-.066**	.347**	-.521**	1.000	-.504**	-.458**	-.208**	.369**	.072**	.281**	.160**	.100**	-.404**	-.339**	-.113**	-.006	-.132**	.007	-.150**	-.543**
		Sig. (2-tailed)			<.001	<.001	<.001	.	<.001	<.001	<.001	<.001	<.001	<.001	<.001	<.001	<.001	<.001	<.001	.693	<.001	.623	<.001	.000
		N			4339	4339	4339	4339	4339	4339	4339	4339	4339	4339	4339	4339	4339	4339	4339	4339	4339	4339	4339	4339
		Bootstrap[c]	Bias		-.001	.000	.000	.000	.000	.000	.000	.001	.000	.001	.000	.000	.000	.000	.001	.000	.000	.001	.000	-.001
			Std. Error		.015	.014	.014	.000	.008	.008	.007	.015	.016	.015	.015	.015	.012	.013	.014	.015	.013	.015	.011	.013
			95% Confidence Interval	Lower	-.099	.320	-.548	1.000	-.519	-.473	-.221	.339	.040	.251	.127	.071	-.426	-.363	-.139	-.038	-.155	-.022	-.171	-.569
				Upper	-.038	.375	-.493	1.000	-.488	-.443	-.195	.399	.102	.311	.190	.131	-.380	-.312	-.084	.024	-.107	.038	-.129	-.518
	Alcoholic hepatitis (1=yes, 0=no)	Correlation Coefficient			-.133**	-.299**	.331**	-.504**	1.000	-.387**	-.176**	-.160**	-.041**	-.168**	-.087**	-.069**	.185**	-.131**	.075**	.005	.066**	-.008	.073**	.275**
		Sig. (2-tailed)			<.001	<.001	<.001	<.001	.	<.001	<.001	<.001	.006	<.001	<.001	<.001	<.001	<.001	<.001	.750	<.001	.599	<.001	<.001
		N			4339	4339	4339	4339	4339	4339	4339	4339	4339	4339	4339	4339	4339	4339	4339	4339	4339	4339	4339	4339
		Bootstrap[c]	Bias		.000	.000	.000	.000	.000	.000	.000	-.001	.000	.000	.001	.001	.000	.000	.001	.001	.000	-.001	.000	.000
			Std. Error		.014	.013	.012	.008	.000	.007	.006	.013	.013	.011	.013	.012	.015	.015	.016	.015	.017	.014	.017	.014
			95% Confidence Interval	Lower	-.161	-.326	.306	-.519	1.000	-.402	-.188	-.186	-.069	-.190	-.112	-.092	.157	-.161	.043	-.022	.034	-.035	.040	.248
				Upper	-.107	-.272	.355	-.488	1.000	-.373	-.165	-.134	-.015	-.146	-.059	-.043	.216	-.103	.106	.035	.099	.021	.106	.301
	Non-alcoholic cyrrhosis	Correlation Coefficient			.206**	-.006	.160**	-.458**	-.387**	1.000	-.160**	-.188**	-.027	-.101**	-.057**	-.015	.202**	.420**	.061**	.013	.081**	.028	.105**	.321**
		Sig. (2-tailed)			<.001	.712	<.001	<.001	<.001	.	<.001	<.001	.079	<.001	<.001	.316	<.001	<.001	<.001	.384	<.001	.063	<.001	<.001
		N			4339	4339	4339	4339	4339	4339	4339	4339	4339	4339	4339	4339	4339	4339	4339	4339	4339	4339	4339	4339
		Bootstrap[c]	Bias		.001	.001	.000	.000	.000	.000	.000	.000	.000	-.001	-.001	-.001	.000	.001	-.001	-.001	-.001	.000	.000	.000
			Std. Error		.015	.014	.014	.008	.007	.000	.005	.011	.014	.013	.014	.015	.016	.015	.016	.016	.017	.016	.017	.013
			95% Confidence Interval	Lower	.176	-.033	.132	-.473	-.402	1.000	-.170	-.209	-.052	-.126	-.086	-.044	.170	.391	.024	-.018	.047	-.001	.069	.295
				Upper	.236	.021	.188	-.443	-.373	1.000	-.150	-.166	.002	-.076	-.031	.014	.233	.451	.089	.043	.114	.059	.138	.347
	Alcoholic cirrhosis (1=yes, 0=no)	Correlation Coefficient			.011	-.115**	.120**	-.208**	-.176**	-.160**	1.000	-.092**	-.016	-.059**	-.051**	-.040**	.088**	.159**	-.025	-.020	-.007	-.049**	-.027	-.016
		Sig. (2-tailed)			.475	<.001	<.001	<.001	<.001	<.001	.	<.001	.287	<.001	<.001	.008	<.001	<.001	.101	.180	.637	.001	.079	.296
		N			4339	4339	4339	4339	4339	4339	4339	4339	4339	4339	4339	4339	4339	4339	4339	4339	4339	4339	4339	4339
		Bootstrap[c]	Bias		.000	-.001	.000	.000	.000	.000	.000	.000	.000	.000	.000	.000	-.001	.000	.000	.000	.001	.000	.000	.000
			Std. Error		.013	.013	.012	.007	.006	.005	.000	.009	.013	.011	.012	.009	.016	.016	.014	.014	.015	.007	.012	.015
			95% Confidence Interval	Lower	-.014	-.141	.096	-.221	-.188	-.170	1.000	-.110	-.040	-.080	-.073	-.057	.057	.128	-.052	-.045	-.034	-.061	-.049	-.044
				Upper	.038	-.090	.143	-.195	-.165	-.150	1.000	-.072	.015	-.038	-.025	-.021	.120	.190	.002	.009	.022	-.035	-.001	.015
	Arterial hypertension	Correlation Coefficient			-.122**	.286**	-.210**	.369**	-.160**	-.188**	-.092**	1.000	.186**	.560**	.250**	.230**	-.212**	-.268**	-.098**	-.047**	-.031*	-.014	-.079**	-.309**
		Sig. (2-tailed)			<.001	<.001	<.001	<.001	<.001	<.001	<.001	.	<.001	.000	<.001	<.001	<.001	<.001	<.001	.002	.043	.343	<.001	<.001
		N			4339	4339	4339	4339	4339	4339	4339	4339	4339	4339	4339	4339	4339	4339	4339	4339	4339	4339	4339	4339
		Bootstrap[c]	Bias		.000	.000	.000	.001	-.001	.000	.000	.000	-.001	.000	.000	.000	.000	.000	.001	.000	.001	.000	.000	.000
			Std. Error		.015	.014	.016	.015	.013	.011	.009	.000	.020	.017	.018	.020	.012	.010	.013	.013	.014	.014	.010	.014
			95% Confidence Interval	Lower	-.154	.258	-.241	.339	-.186	-.209	-.110	1.000	.146	.526	.213	.191	-.236	-.288	-.121	-.070	-.055	-.042	-.099	-.336
				Upper	-.091	.313	-.179	.399	-.134	-.166	-.072	1.000	.223	.593	.285	.270	-.187	-.247	-.073	-.022	-.003	.013	-.058	-.283
	Cerebrovascular Accident	Correlation Coefficient			-.092**	.102**	-.063**	.072**	-.041**	-.027	-.016	.186**	1.000	.208**	.086**	.039*	-.065**	-.087**	-.044**	-.004	.029	-.004	-.004	-.089**
		Sig. (2-tailed)			<.001	<.001	<.001	<.001	.006	.079	.287	<.001	.	<.001	<.001	.011	<.001	<.001	.004	.767	.059	.784	.776	<.001
		N			4339	4339	4339	4339	4339	4339	4339	4339	4339	4339	4339	4339	4339	4339	4339	4339	4339	4339	4339	4339
		Bootstrap[c]	Bias		-.001	.000	.000	.000	.000	.000	.000	-.001	.000	.001	.000	-.001	.001	.001	.000	.000	.000	.000	.000	.000
			Std. Error		.013	.013	.016	.016	.013	.014	.013	.020	.000	.022	.019	.021	.013	.011	.012	.014	.017	.015	.014	.014
			95% Confidence Interval	Lower	-.117	.075	-.095	.040	-.069	-.052	-.040	.146	1.000	.166	.048	.002	-.088	-.106	-.065	-.031	-.003	-.030	-.033	-.117
				Upper	-.064	.128	-.031	.102	-.015	.002	.015	.223	1.000	.252	.125	.084	-.038	-.063	-.019	.027	.065	.026	.026	-.060
	Heart failure	Correlation Coefficient			-.072**	.268**	-.164**	.281**	-.168**	-.101**	-.059**	.560**	.208**	1.000	.223**	.160**	-.154**	-.187**	-.076**	-.028	-.010	-.001	-.054**	-.238**
		Sig. (2-tailed)			<.001	<.001	<.001	<.001	<.001	<.001	<.001	.000	<.001	.	<.001	<.001	<.001	<.001	<.001	.065	.491	.971	<.001	<.001
		N			4339	4339	4339	4339	4339	4339	4339	4339	4339	4339	4339	4339	4339	4339	4339	4339	4339	4339	4339	4339
		Bootstrap[c]	Bias		-.001	.000	.000	.001	.000	-.001	.000	.000	.001	.000	-.001	.001	.000	.000	.000	-.001	.000	.000	.000	.000
			Std. Error		.015	.014	.016	.015	.011	.013	.011	.017	.022	.000	.019	.021	.013	.012	.013	.014	.015	.015	.012	.014
			95% Confidence Interval	Lower	-.104	.240	-.198	.251	-.190	-.126	-.080	.526	.166	1.000	.186	.121	-.181	-.209	-.101	-.053	-.038	-.029	-.076	-.265
				Upper	-.043	.295	-.134	.311	-.146	-.076	-.038	.593	.252	1.000	.261	.202	-.129	-.162	-.052	.000	.020	.030	-.031	-.211
	DIABETES MELLITUS	Correlation Coefficient			-.038*	.128**	-.088**	.160**	-.087**	-.057**	-.051**	.250**	.086**	.223**	1.000	.125**	-.059**	-.110**	-.052**	-.030*	.000	.009	-.009	-.126**
		Sig. (2-tailed)			.012	<.001	<.001	<.001	<.001	<.001	<.001	<.001	<.001	<.001	.	<.001	<.001	<.001	<.001	.046	.983	.551	.556	<.001
		N			4339	4339	4339	4339	4339	4339	4339	4339	4339	4339	4339	4339	4339	4339	4339	4339	4339	4339	4339	4339
		Bootstrap[c]	Bias		.000	.000	.001	.000	.001	-.001	.000	.000	.000	-.001	.000	.000	-.001	.000	.001	.001	.000	.001	.000	.000
			Std. Error		.015	.014	.016	.015	.013	.014	.012	.018	.019	.019	.000	.021	.014	.014	.014	.013	.015	.015	.015	.015
			95% Confidence Interval	Lower	-.068	.102	-.119	.127	-.112	-.086	-.073	.213	.048	.186	1.000	.085	-.086	-.136	-.079	-.055	-.030	-.020	-.038	-.155
				Upper	-.009	.154	-.056	.190	-.059	-.031	-.025	.285	.125	.261	1.000	.168	-.031	-.082	-.025	-.004	.030	.043	.020	-.097
	OBESITY	Correlation Coefficient			-.034*	.036*	-.061**	.100**	-.069**	-.015	-.040**	.230**	.039*	.160**	.125**	1.000	-.044**	-.074**	-.004	-.006	.020	-.025	-.002	-.087**
		Sig. (2-tailed)			.026	.019	<.001	<.001	<.001	.316	.008	<.001	.011	<.001	<.001	.	.004	<.001	.802	.697	.178	.107	.876	<.001
		N			4339	4339	4339	4339	4339	4339	4339	4339	4339	4339	4339	4339	4339	4339	4339	4339	4339	4339	4339	4339
		Bootstrap[c]	Bias		.000	.000	.000	.000	.001	-.001	.000	.000	-.001	.001	.000	.000	.000	.000	.001	.000	.000	.000	.000	.000
			Std. Error		.016	.013	.016	.015	.012	.015	.009	.020	.021	.021	.021	.000	.014	.012	.015	.014	.017	.011	.015	.014
			95% Confidence Interval	Lower	-.064	.009	-.092	.071	-.092	-.044	-.057	.191	.002	.121	.085	1.000	-.069	-.097	-.032	-.030	-.011	-.043	-.031	-.114
				Upper	-.001	.061	-.030	.131	-.043	.014	-.021	.270	.084	.202	.168	1.000	-.017	-.049	.026	.024	.056	-.001	.031	-.058
	Child-Pugh	Correlation Coefficient			.079**	-.172**	.220**	-.404**	.185**	.202**	.088**	-.212**	-.065**	-.154**	-.059**	-.044**	1.000	.294**	.143**	.027	.122**	-.007	.135**	.394**
		Sig. (2-tailed)			<.001	<.001	<.001	<.001	<.001	<.001	<.001	<.001	<.001	<.001	<.001	.004	.	<.001	<.001	.081	<.001	.642	<.001	<.001
		N			4339	4339	4339	4339	4339	4339	4339	4339	4339	4339	4339	4339	4339	4339	4339	4339	4339	4339	4339	4339
		Bootstrap[c]	Bias		.000	-.001	.000	.000	.000	.000	-.001	.000	.001	.000	-.001	.000	.000	.000	.000	.000	.000	-.001	.000	.001
			Std. Error		.015	.014	.014	.012	.015	.016	.016	.012	.013	.013	.014	.014	.000	.015	.016	.015	.016	.015	.017	.013
			95% Confidence Interval	Lower	.049	-.201	.194	-.426	.157	.170	.057	-.236	-.088	-.181	-.086	-.069	1.000	.264	.112	-.004	.088	-.036	.103	.369
				Upper	.110	-.145	.248	-.380	.216	.233	.120	-.187	-.038	-.129	-.031	-.017	1.000	.322	.173	.057	.154	.022	.169	.420
	ASCITES	Correlation Coefficient			.206**	-.109**	.160**	-.339**	-.131**	.420**	.159**	-.268**	-.087**	-.187**	-.110**	-.074**	.294**	1.000	.084**	.050**	.027	.031*	.122**	.380**
		Sig. (2-tailed)			<.001	<.001	<.001	<.001	<.001	<.001	<.001	<.001	<.001	<.001	<.001	<.001	<.001	.	<.001	<.001	.072	.045	<.001	<.001
		N			4339	4339	4339	4339	4339	4339	4339	4339	4339	4339	4339	4339	4339	4339	4339	4339	4339	4339	4339	4339
		Bootstrap[c]	Bias		.000	.000	-.001	.000	.000	.001	.000	.000	.001	.000	.000	.000	.000	.000	.000	.000	.000	.000	.001	.001
			Std. Error		.015	.014	.015	.013	.015	.015	.016	.010	.011	.012	.014	.012	.015	.000	.016	.016	.016	.016	.016	.014
			95% Confidence Interval	Lower	.177	-.135	.129	-.363	-.161	.391	.128	-.288	-.106	-.209	-.136	-.097	.264	1.000	.052	.017	-.005	-.001	.089	.353
				Upper	.236	-.081	.190	-.312	-.103	.451	.190	-.247	-.063	-.162	-.082	-.049	.322	1.000	.116	.081	.061	.062	.152	.410
	Esophageal varices	Correlation Coefficient			.091**	-.072**	.048**	-.113**	.075**	.061**	-.025	-.098**	-.044**	-.076**	-.052**	-.004	.143**	.084**	1.000	.258**	.126**	.016	-.030*	.208**
		Sig. (2-tailed)			<.001	<.001	.002	<.001	<.001	<.001	.101	<.001	.004	<.001	<.001	.802	<.001	<.001	.	<.001	<.001	.285	.049	<.001
		N			4339	4339	4339	4339	4339	4339	4339	4339	4339	4339	4339	4339	4339	4339	4339	4339	4339	4339	4339	4339
		Bootstrap[c]	Bias		-.001	.000	.001	.001	.001	-.001	.000	.001	.000	.000	.001	.001	.000	.000	.000	.001	.000	.000	.000	-.001
			Std. Error		.015	.015	.015	.014	.016	.016	.014	.013	.012	.013	.014	.015	.016	.016	.000	.020	.018	.016	.014	.013
			95% Confidence Interval	Lower	.060	-.101	.019	-.139	.043	.024	-.052	-.121	-.065	-.101	-.079	-.032	.112	.052	1.000	.220	.089	-.014	-.057	.181
				Upper	.119	-.043	.077	-.084	.106	.089	.002	-.073	-.019	-.052	-.025	.026	.173	.116	1.000	.299	.161	.050	-.002	.232
	DIGESTIVE BLEEDING	Correlation Coefficient			-.001	-.039*	.013	-.006	.005	.013	-.020	-.047**	-.004	-.028	-.030*	-.006	.027	.050**	.258**	1.000	.018	-.015	-.010	.045**
		Sig. (2-tailed)			.926	.011	.379	.693	.750	.384	.180	.002	.767	.065	.046	.697	.081	<.001	<.001	.	.226	.308	.502	.003
		N			4339	4339	4339	4339	4339	4339	4339	4339	4339	4339	4339	4339	4339	4339	4339	4339	4339	4339	4339	4339
		Bootstrap[c]	Bias		-.001	.000	.000	.000	.001	-.001	.000	.000	.000	-.001	.001	.000	.000	.000	.001	.000	.000	.000	.000	.000
			Std. Error		.014	.016	.015	.015	.015	.016	.014	.013	.014	.014	.013	.014	.015	.016	.020	.000	.017	.013	.014	.014
			95% Confidence Interval	Lower	-.029	-.070	-.019	-.038	-.022	-.018	-.045	-.070	-.031	-.053	-.055	-.030	-.004	.017	.220	1.000	-.014	-.039	-.035	.017
				Upper	.027	-.008	.043	.024	.035	.043	.009	-.022	.027	.000	-.004	.024	.057	.081	.299	1.000	.051	.011	.019	.073
	ENCEPHALOPATHY	Correlation Coefficient			-.001	-.008	.100**	-.132**	.066**	.081**	-.007	-.031*	.029	-.010	.000	.020	.122**	.027	.126**	.018	1.000	-.021	.003	.112**
		Sig. (2-tailed)			.962	.593	<.001	<.001	<.001	<.001	.637	.043	.059	.491	.983	.178	<.001	.072	<.001	.226	.	.163	.862	<.001
		N			4339	4339	4339	4339	4339	4339	4339	4339	4339	4339	4339	4339	4339	4339	4339	4339	4339	4339	4339	4339
		Bootstrap[c]	Bias		-.001	.000	.000	.000	.000	-.001	.001	.001	.000	.000	.000	.000	.000	.000	.000	.000	.000	.000	.001	.000
			Std. Error		.016	.015	.013	.013	.017	.017	.015	.014	.017	.015	.015	.017	.016	.016	.018	.017	.000	.013	.016	.014
			95% Confidence Interval	Lower	-.033	-.036	.074	-.155	.034	.047	-.034	-.055	-.003	-.038	-.030	-.011	.088	-.005	.089	-.014	1.000	-.044	-.027	.084
				Upper	.032	.020	.126	-.107	.099	.114	.022	-.003	.065	.020	.030	.056	.154	.061	.161	.051	1.000	.004	.035	.140
	Hepatocellular Carcinoma	Correlation Coefficient			-.009	.025	.042**	.007	-.008	.028	-.049**	-.014	-.004	-.001	.009	-.025	-.007	.031*	.016	-.015	-.021	1.000	-.016	-.015
		Sig. (2-tailed)			.564	.100	.006	.623	.599	.063	.001	.343	.784	.971	.551	.107	.642	.045	.285	.308	.163	.	.292	.329
		N			4339	4339	4339	4339	4339	4339	4339	4339	4339	4339	4339	4339	4339	4339	4339	4339	4339	4339	4339	4339
		Bootstrap[c]	Bias		.001	.000	.000	.001	-.001	.000	.000	.000	.000	.000	.001	.000	-.001	.000	.000	.000	.000	.000	-.001	.000
			Std. Error		.016	.015	.014	.015	.014	.016	.007	.014	.015	.015	.015	.011	.015	.016	.016	.013	.013	.000	.013	.016
			95% Confidence Interval	Lower	-.039	-.004	.013	-.022	-.035	-.001	-.061	-.042	-.030	-.029	-.020	-.043	-.036	-.001	-.014	-.039	-.044	1.000	-.041	-.046
				Upper	.022	.054	.070	.038	.021	.059	-.035	.013	.026	.030	.043	-.001	.022	.062	.050	.011	.004	1.000	.010	.015
	Hepatorenal syndrome	Correlation Coefficient			.028	-.021	.076**	-.150**	.073**	.105**	-.027	-.079**	-.004	-.054**	-.009	-.002	.135**	.122**	-.030*	-.010	.003	-.016	1.000	.111**
		Sig. (2-tailed)			.061	.165	<.001	<.001	<.001	<.001	.079	<.001	.776	<.001	.556	.876	<.001	<.001	.049	.502	.862	.292	.	<.001
		N			4339	4339	4339	4339	4339	4339	4339	4339	4339	4339	4339	4339	4339	4339	4339	4339	4339	4339	4339	4339
		Bootstrap[c]	Bias		.000	.000	.000	.000	.000	.000	.000	.000	.000	.000	.000	.000	.000	.001	.000	.000	.001	-.001	.000	.000
			Std. Error		.015	.014	.014	.011	.017	.017	.012	.010	.014	.012	.015	.015	.017	.016	.014	.014	.016	.013	.000	.014
			95% Confidence Interval	Lower	-.001	-.049	.047	-.171	.040	.069	-.049	-.099	-.033	-.076	-.038	-.031	.103	.089	-.057	-.035	-.027	-.041	1.000	.083
				Upper	.058	.006	.100	-.129	.106	.138	-.001	-.058	.026	-.031	.020	.031	.169	.152	-.002	.019	.035	.010	1.000	.138
	Portal hypertension	Correlation Coefficient			.176**	-.226**	.303**	-.543**	.275**	.321**	-.016	-.309**	-.089**	-.238**	-.126**	-.087**	.394**	.380**	.208**	.045**	.112**	-.015	.111**	1.000
		Sig. (2-tailed)			<.001	<.001	<.001	.000	<.001	<.001	.296	<.001	<.001	<.001	<.001	<.001	<.001	<.001	<.001	.003	<.001	.329	<.001	.
		N			4339	4339	4339	4339	4339	4339	4339	4339	4339	4339	4339	4339	4339	4339	4339	4339	4339	4339	4339	4339
		Bootstrap[c]	Bias		.001	.000	.000	-.001	.000	.000	.000	.000	.000	.000	.000	.000	.001	.001	-.001	.000	.000	.000	.000	.000
			Std. Error		.015	.015	.015	.013	.014	.013	.015	.014	.014	.014	.015	.014	.013	.014	.013	.014	.014	.016	.014	.000
			95% Confidence Interval	Lower	.149	-.257	.274	-.569	.248	.295	-.044	-.336	-.117	-.265	-.155	-.114	.369	.353	.181	.017	.084	-.046	.083	1.000
				Upper	.205	-.197	.332	-.518	.301	.347	.015	-.283	-.060	-.211	-.097	-.058	.420	.410	.232	.073	.140	.015	.138	1.000

**. Correlation is significant at the 0.01 level (2-tailed).

Confidence Intervals of Spearman's rho

	Spearman's rho	Significance(2-tailed)	95% Confidence Intervals (2-tailed)[a,b] Lower	Upper
Number_Hospitalizations - AGE_GROUP	-.048	.002	-.078	-.017
Number_Hospitalizations - SEX	-.021	.168	-.052	.010
Number_Hospitalizations - Chronic hepatitis C (1=yes, 0=no)	-.066	.000	-.097	-.036
Number_Hospitalizations - Alcoholic hepatitis (1=yes, 0=no)	-.133	.000	-.163	-.103
Number_Hospitalizations - Non-alcoholic cirrhosis	.206	.000	.176	.235
Number_Hospitalizations - Alcoholic cirrhosis (1=yes, 0=no)	.011	.475	-.020	.041
Number_Hospitalizations - Arterial hypertension	-.122	.000	-.152	-.091
Number_Hospitalizations - Cerebrovascular Accident	-.092	.000	-.122	-.062
Number_Hospitalizations - Heart failure	-.072	.000	-.103	-.042
Number_Hospitalizations - DIABETES MELLITUS	-.038	.012	-.069	-.007

Number_Hospitalizations - OBESITY	-.034	.026	-.064	-.003
Number_Hospitalizations - Child–Pugh	.079	.000	.049	.110
Number_Hospitalizations - ASCITES	.206	.000	.176	.235
Number_Hospitalizations - Esophageal varices	.091	.000	.060	.121
Number_Hospitalizations - DIGESTIVE BLEEDING	-.001	.926	-.032	.029
Number_Hospitalizations - ENCEPHALOPATHY	-.001	.962	-.031	.030
Number_Hospitalizations - Hepatocellular Carcinoma	-.009	.564	-.039	.022
Number_Hospitalizations - Hepatorenal syndrome	.028	.061	-.002	.059
Number_Hospitalizations - Portal hypertension	.176	.000	.146	.205
AGE_GROUP - SEX	-.316	.000	-.344	-.289
AGE_GROUP - Chronic hepatitis C (1=yes, 0=no)	.347	.000	.320	.374
AGE_GROUP - Alcoholic hepatitis (1=yes, 0=no)	-.299	.000	-.326	-.271
AGE_GROUP - Non-alcoholic cirrhosis	-.006	.712	-.036	.025
AGE_GROUP - Alcoholic cirrhosis (1=yes, 0=no)	-.115	.000	-.145	-.084

AGE_GROUP - Arterial hypertension	.286	.000	.258	.314
AGE_GROUP - Cerebrovascular Accident	.102	.000	.072	.132
AGE_GROUP - Heart failure	.268	.000	.239	.296
AGE_GROUP - DIABETES MELLITUS	.128	.000	.098	.158
AGE_GROUP - OBESITY	.036	.019	.005	.066
AGE_GROUP - Child–Pugh	-.172	.000	-.201	-.142
AGE_GROUP - ASCITES	-.109	.000	-.139	-.078
AGE_GROUP - Esophageal varices	-.072	.000	-.103	-.042
AGE_GROUP - DIGESTIVE BLEEDING	-.039	.011	-.069	-.008
AGE_GROUP - ENCEPHALOPATHY	-.008	.593	-.039	.023
AGE_GROUP - Hepatocellular Carcinoma	.025	.100	-.006	.056
AGE_GROUP - Hepatorenal syndrome	-.021	.165	-.052	.010
AGE_GROUP - Portal hypertension	-.226	.000	-.255	-.197
SEX - Chronic hepatitis C (1=yes, 0=no)	-.521	.000	-.543	-.498
SEX - Alcoholic hepatitis (1=yes, 0=no)	.331	.000	.304	.358
SEX - Non-alcoholic cirrhosis	.160	.000	.130	.189

SEX - Alcoholic cirrhosis (1=yes, 0=no)	.120	.000	.090	.150
SEX - Arterial hypertension	-.210	.000	-.239	-.181
SEX - Cerebrovascular Accident	-.063	.000	-.093	-.032
SEX - Heart failure	-.164	.000	-.194	-.134
SEX - DIABETES MELLITUS	-.088	.000	-.118	-.057
SEX - OBESITY	-.061	.000	-.091	-.030
SEX - Child–Pugh	.220	.000	.190	.249
SEX - ASCITES	.160	.000	.130	.189
SEX - Esophageal varices	.048	.002	.017	.078
SEX - DIGESTIVE BLEEDING	.013	.379	-.017	.044
SEX - ENCEPHALOPATHY	.100	.000	.070	.130
SEX - Hepatocellular Carcinoma	.042	.006	.011	.072
SEX - Hepatorenal syndrome	.076	.000	.045	.106
SEX - Portal hypertension	.303	.000	.275	.330
Chronic hepatitis C (1=yes, 0=no) - Alcoholic hepatitis (1=yes, 0=no)	-.504	.000	-.526	-.480
Chronic hepatitis C (1=yes, 0=no) - Non-alcoholic cirrhosis	-.458	.000	-.482	-.434
Chronic hepatitis C (1=yes, 0=no) - Alcoholic cirrhosis (1=yes, 0=no)	-.208	.000	-.237	-.179

Chronic hepatitis C (1=yes, 0=no) - Arterial hypertension	.369	.000	.343	.396
Chronic hepatitis C (1=yes, 0=no) - Cerebrovascular Accident	.072	.000	.041	.102
Chronic hepatitis C (1=yes, 0=no) - Heart failure	.281	.000	.252	.309
Chronic hepatitis C (1=yes, 0=no) - DIABETES MELLITUS	.160	.000	.130	.190
Chronic hepatitis C (1=yes, 0=no) - OBESITY	.100	.000	.070	.130
Chronic hepatitis C (1=yes, 0=no) - Child–Pugh	-.404	.000	-.429	-.378
Chronic hepatitis C (1=yes, 0=no) - ASCITES	-.339	.000	-.366	-.312
Chronic hepatitis C (1=yes, 0=no) - Esophageal varices	-.113	.000	-.143	-.083
Chronic hepatitis C (1=yes, 0=no) - DIGESTIVE BLEEDING	-.006	.693	-.037	.025
Chronic hepatitis C (1=yes, 0=no) - ENCEPHALOPATHY	-.132	.000	-.162	-.102
Chronic hepatitis C (1=yes, 0=no) - Hepatocellular Carcinoma	.007	.623	-.023	.038

Chronic hepatitis C (1=yes, 0=no) - Hepatorenal syndrome	-.150	.000	-.180	-.120
Chronic hepatitis C (1=yes, 0=no) - Portal hypertension	-.543	.000	-.564	-.521
Alcoholic hepatitis (1=yes, 0=no) - Non-alcoholic cirrhosis	-.387	.000	-.412	-.360
Alcoholic hepatitis (1=yes, 0=no) - Alcoholic cirrhosis (1=yes, 0=no)	-.176	.000	-.205	-.146
Alcoholic hepatitis (1=yes, 0=no) - Arterial hypertension	-.160	.000	-.189	-.130
Alcoholic hepatitis (1=yes, 0=no) - Cerebrovascular Accident	-.041	.006	-.072	-.011
Alcoholic hepatitis (1=yes, 0=no) - Heart failure	-.168	.000	-.197	-.138
Alcoholic hepatitis (1=yes, 0=no) - DIABETES MELLITUS	-.087	.000	-.117	-.056
Alcoholic hepatitis (1=yes, 0=no) - OBESITY	-.069	.000	-.100	-.039
Alcoholic hepatitis (1=yes, 0=no) - Child–Pugh	.185	.000	.155	.215
Alcoholic hepatitis (1=yes, 0=no) - ASCITES	-.131	.000	-.161	-.101

Alcoholic hepatitis (1=yes, 0=no) - Esophageal varices	.075	.000	.044	.105
Alcoholic hepatitis (1=yes, 0=no) - DIGESTIVE BLEEDING	.005	.750	-.026	.035
Alcoholic hepatitis (1=yes, 0=no) - ENCEPHALOPATHY	.066	.000	.036	.097
Alcoholic hepatitis (1=yes, 0=no) - Hepatocellular Carcinoma	-.008	.599	-.039	.023
Alcoholic hepatitis (1=yes, 0=no) - Hepatorenal syndrome	.073	.000	.042	.103
Alcoholic hepatitis (1=yes, 0=no) - Portal hypertension	.275	.000	.246	.303
Non-alcoholic cirrhosis - Alcoholic cirrhosis (1=yes, 0=no)	-.160	.000	-.190	-.130
Non-alcoholic cirrhosis - Arterial hypertension	-.188	.000	-.217	-.158
Non-alcoholic cirrhosis - Cerebrovascular Accident	-.027	.079	-.057	.004
Non-alcoholic cirrhosis - Heart failure	-.101	.000	-.131	-.070
Non-alcoholic cirrhosis - DIABETES MELLITUS	-.057	.000	-.087	-.026

Non-alcoholic cirrhosis - OBESITY	-.015	.316	-.046	.015
Non-alcoholic cirrhosis - Child–Pugh	.202	.000	.172	.231
Non-alcoholic cirrhosis - ASCITES	.420	.000	.394	.444
Non-alcoholic cirrhosis - Esophageal varices	.061	.000	.030	.091
Non-alcoholic cirrhosis - DIGESTIVE BLEEDING	.013	.384	-.017	.044
Non-alcoholic cirrhosis - ENCEPHALOPATHY	.081	.000	.050	.111
Non-alcoholic cirrhosis - Hepatocellular Carcinoma	.028	.063	-.002	.059
Non-alcoholic cirrhosis - Hepatorenal syndrome	.105	.000	.075	.135
Non-alcoholic cirrhosis - Portal hypertension	.321	.000	.294	.349
Alcoholic cirrhosis (1=yes, 0=no) - Arterial hypertension	-.092	.000	-.122	-.062
Alcoholic cirrhosis (1=yes, 0=no) - Cerebrovascular Accident	-.016	.287	-.047	.014
Alcoholic cirrhosis (1=yes, 0=no) - Heart failure	-.059	.000	-.090	-.029
Alcoholic cirrhosis (1=yes, 0=no) - DIABETES MELLITUS	-.051	.001	-.081	-.020

Alcoholic cirrhosis (1=yes, 0=no) - OBESITY	-.040	.008	-.071	-.010
Alcoholic cirrhosis (1=yes, 0=no) - Child–Pugh	.088	.000	.058	.119
Alcoholic cirrhosis (1=yes, 0=no) - ASCITES	.159	.000	.129	.189
Alcoholic cirrhosis (1=yes, 0=no) - Esophageal varices	-.025	.101	-.055	.006
Alcoholic cirrhosis (1=yes, 0=no) - DIGESTIVE BLEEDING	-.020	.180	-.051	.010
Alcoholic cirrhosis (1=yes, 0=no) - ENCEPHALOPATHY	-.007	.637	-.038	.023
Alcoholic cirrhosis (1=yes, 0=no) - Hepatocellular Carcinoma	-.049	.001	-.080	-.018
Alcoholic cirrhosis (1=yes, 0=no) - Hepatorenal syndrome	-.027	.079	-.057	.004
Alcoholic cirrhosis (1=yes, 0=no) - Portal hypertension	-.016	.296	-.046	.015
Arterial hypertension - Cerebrovascular Accident	.186	.000	.156	.215
Arterial hypertension - Heart failure	.560	.000	.539	.581
Arterial hypertension - DIABETES MELLITUS	.250	.000	.221	.278

Arterial hypertension - OBESITY	.230	.000	.200	.258
Arterial hypertension - Child–Pugh	-.212	.000	-.241	-.183
Arterial hypertension - ASCITES	-.268	.000	-.296	-.239
Arterial hypertension - Esophageal varices	-.098	.000	-.128	-.068
Arterial hypertension - DIGESTIVE BLEEDING	-.047	.002	-.077	-.016
Arterial hypertension - ENCEPHALOPATHY	-.031	.043	-.061	.000
Arterial hypertension - Hepatocellular Carcinoma	-.014	.343	-.045	.016
Arterial hypertension - Hepatorenal syndrome	-.079	.000	-.109	-.049
Arterial hypertension - Portal hypertension	-.309	.000	-.337	-.281
Cerebrovascular Accident - Heart failure	.208	.000	.178	.237
Cerebrovascular Accident - DIABETES MELLITUS	.086	.000	.056	.116
Cerebrovascular Accident - OBESITY	.039	.011	.008	.069
Cerebrovascular Accident - Child–Pugh	-.065	.000	-.096	-.035
Cerebrovascular Accident - ASCITES	-.087	.000	-.117	-.056

Cerebrovascular Accident - Esophageal varices	-.044	.004	-.074	-.013
Cerebrovascular Accident - DIGESTIVE BLEEDING	-.004	.767	-.035	.026
Cerebrovascular Accident - ENCEPHALOPATHY	.029	.059	-.002	.059
Cerebrovascular Accident - Hepatocellular Carcinoma	-.004	.784	-.035	.026
Cerebrovascular Accident - Hepatorenal syndrome	-.004	.776	-.035	.026
Cerebrovascular Accident - Portal hypertension	-.089	.000	-.120	-.059
Heart failure - DIABETES MELLITUS	.223	.000	.194	.252
Heart failure - OBESITY	.160	.000	.130	.190
Heart failure - Child–Pugh	-.154	.000	-.184	-.124
Heart failure - ASCITES	-.187	.000	-.216	-.157
Heart failure - Esophageal varices	-.076	.000	-.107	-.046
Heart failure - DIGESTIVE BLEEDING	-.028	.065	-.059	.003
Heart failure - ENCEPHALOPATHY	-.010	.491	-.041	.020
Heart failure - Hepatocellular Carcinoma	-.001	.971	-.031	.030
Heart failure - Hepatorenal syndrome	-.054	.000	-.084	-.023

Heart failure - Portal hypertension	-.238	.000	-.267	-.209
DIABETES MELLITUS - OBESITY	.125	.000	.095	.155
DIABETES MELLITUS - Child–Pugh	-.059	.000	-.089	-.028
DIABETES MELLITUS - ASCITES	-.110	.000	-.140	-.079
DIABETES MELLITUS - Esophageal varices	-.052	.001	-.083	-.022
DIABETES MELLITUS - DIGESTIVE BLEEDING	-.030	.046	-.061	.000
DIABETES MELLITUS - ENCEPHALOPATHY	.000	.983	-.030	.031
DIABETES MELLITUS - Hepatocellular Carcinoma	.009	.551	-.022	.040
DIABETES MELLITUS - Hepatorenal syndrome	-.009	.556	-.040	.022
DIABETES MELLITUS - Portal hypertension	-.126	.000	-.156	-.096
OBESITY - Child–Pugh	-.044	.004	-.075	-.014
OBESITY - ASCITES	-.074	.000	-.104	-.043
OBESITY - Esophageal varices	-.004	.802	-.034	.027
OBESITY - DIGESTIVE BLEEDING	-.006	.697	-.037	.025
OBESITY - ENCEPHALOPATHY	.020	.178	-.010	.051

OBESITY - Hepatocellular Carcinoma	-.025	.107	-.055	.006
OBESITY - Hepatorenal syndrome	-.002	.876	-.033	.028
OBESITY - Portal hypertension	-.087	.000	-.117	-.056
Child–Pugh - ASCITES	.294	.000	.265	.321
Child–Pugh - Esophageal varices	.143	.000	.113	.173
Child–Pugh - DIGESTIVE BLEEDING	.027	.081	-.004	.057
Child–Pugh - ENCEPHALOPATHY	.122	.000	.092	.152
Child–Pugh - Hepatocellular Carcinoma	-.007	.642	-.038	.024
Child–Pugh - Hepatorenal syndrome	.135	.000	.105	.165
Child–Pugh - Portal hypertension	.394	.000	.368	.420
ASCITES - Esophageal varices	.084	.000	.054	.115
ASCITES - DIGESTIVE BLEEDING	.050	.001	.020	.081
ASCITES - ENCEPHALOPATHY	.027	.072	-.003	.058
ASCITES - Hepatocellular Carcinoma	.031	.045	.000	.061

ASCITES - Hepatorenal syndrome	.122	.000	.091	.152
ASCITES - Portal hypertension	.380	.000	.354	.406
Esophageal varices - DIGESTIVE BLEEDING	.258	.000	.229	.286
Esophageal varices - ENCEPHALOPATHY	.126	.000	.095	.156
Esophageal varices - Hepatocellular Carcinoma	.016	.285	-.014	.047
Esophageal varices - Hepatorenal syndrome	-.030	.049	-.060	.001
Esophageal varices - Portal hypertension	.208	.000	.178	.237
DIGESTIVE BLEEDING - ENCEPHALOPATHY	.018	.226	-.012	.049
DIGESTIVE BLEEDING - Hepatocellular Carcinoma	-.015	.308	-.046	.015
DIGESTIVE BLEEDING - Hepatorenal syndrome	-.010	.502	-.041	.020
DIGESTIVE BLEEDING - Portal hypertension	.045	.003	.015	.076
ENCEPHALOPATHY - Hepatocellular Carcinoma	-.021	.163	-.052	.009
ENCEPHALOPATHY - Hepatorenal syndrome	.003	.862	-.028	.033
ENCEPHALOPATHY - Portal hypertension	.112	.000	.081	.142

Hepatocellular Carcinoma - Hepatorenal syndrome	-.016	.292	-.047	.015
Hepatocellular Carcinoma - Portal hypertension	-.015	.329	-.045	.016
Hepatorenal syndrome - Portal hypertension	.111	.000	.081	.141

a. Estimation is based on Fisher's r-to-z transformation.

b. Estimation of standard error is based on the formula proposed by Fieller, Hartley, and Pearson.

Table S41. Related-Samples Wilcoxon Signed Rank Test Summary.

Related-Samples Wilcoxon Signed Rank Test Summary

Total N	2358
Test Statistic	40110.000
Standard Error	1989.944
Standardized Test Statistic	2.818
Asymptotic Sig.(2-sided test)	.005

Hypothesis Test Summary

	Null Hypothesis	Test	Sig.[a,b]	Decision
1	The median of differences between Child–Pugh and Child–Pugh equals 0.	Related-Samples Wilcoxon Signed Rank Test	.005	Reject the null hypothesis.

a. The significance level is .050.

b. Asymptotic significance is displayed.

Related-Samples Wilcoxon Signed Rank Test Summary

Total N	2358
Test Statistic	40110.000
Standard Error	1989.944

Standardized Test Statistic	2.818
Asymptotic Sig.(2-sided test)	.005
